# Supplementary material for: DOT1L regulates chromatin reorganization and gene expression during sperm differentiation
Source: EMBO Rep. 2023 Apr 26;24(6):e56316. doi: 10.15252/embr.202256316 (PMC10240200; doi:10.15252/embr.202256316)
Supplement: Supplementary file 1 — Appendix [file EMBR-24-e56316-s006.pdf]

Table of contents

Appendix Figure S1 2
Appendix Figure S2 8
Appendix Figure S3 10
Appendix Figure S4 13
Appendix Figure S5 18
Appendix Table S1 21
Appendix Table S2 22
References 23

Appendix Figure S1

1A

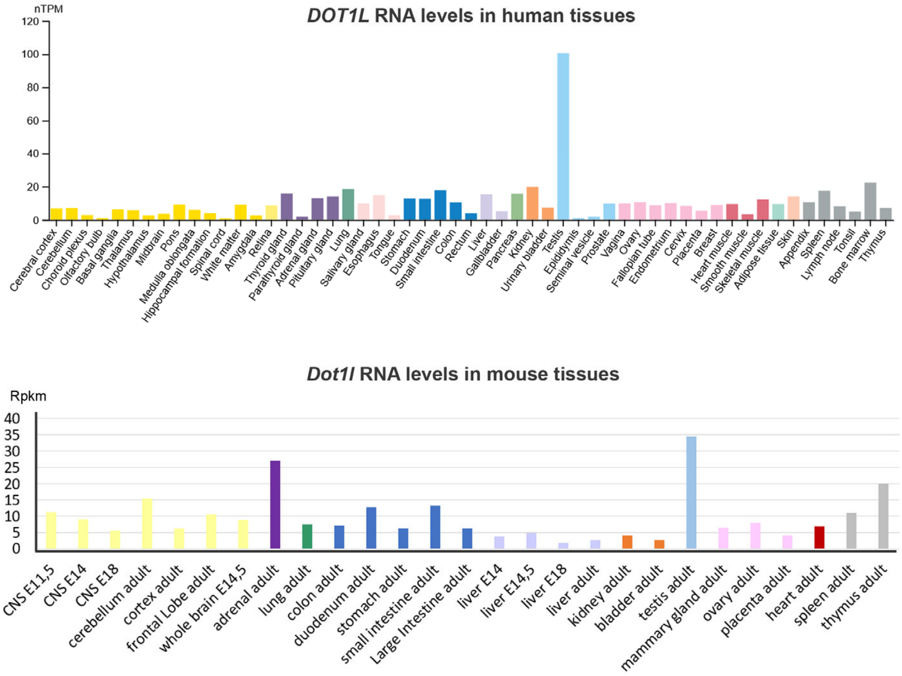

1B

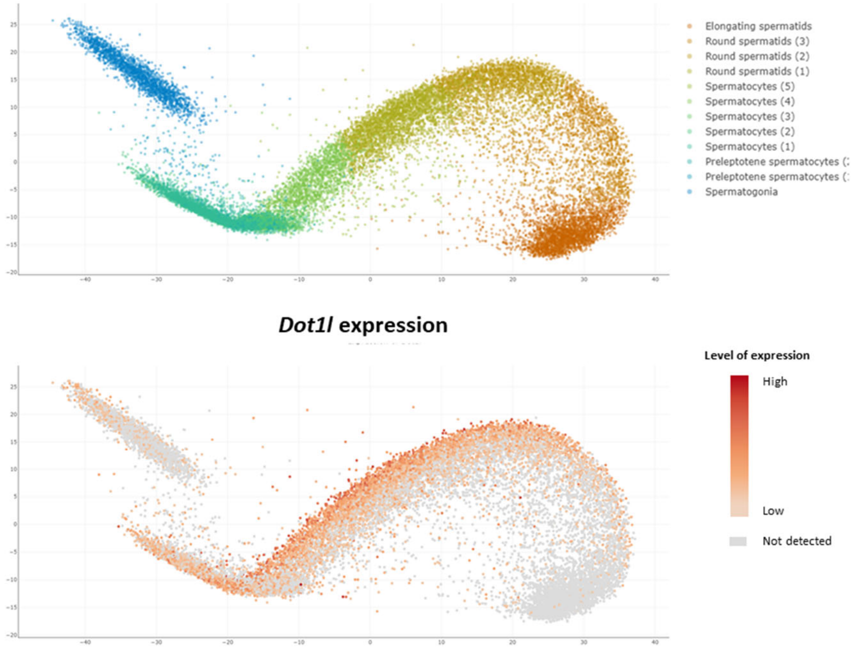

1C

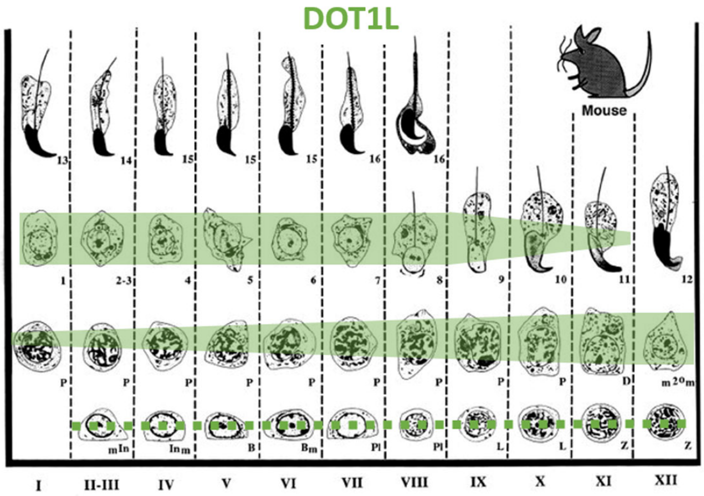
DOT1L
Mouse
I
II-III
IV
V
VI
VII
VIII
IX
X
XI
XII

1D

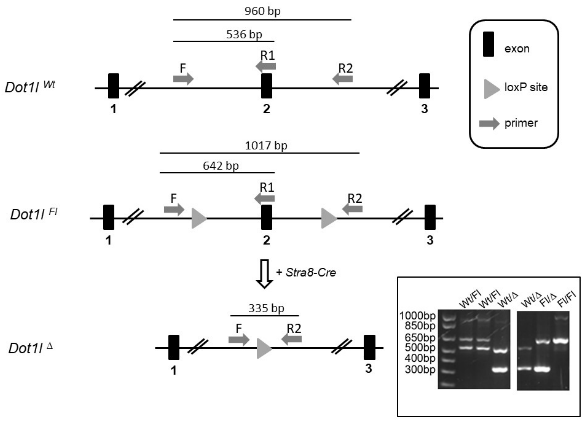
960 bp
536 bp
F
R1
R2
Dot1l Wt
1
2
3
exon
loxP site
primer
1017 bp
642 bp
Dot1l Fl
+ Stra8-Cre
335 bp
Dot1l Δ
1000bp
850bp
650bp
500bp
400bp
300bp
Wt/Fl
Wt/Fl
Wt/Δ
Wt/Δ
Fl/Δ
Fl/Fl

1E

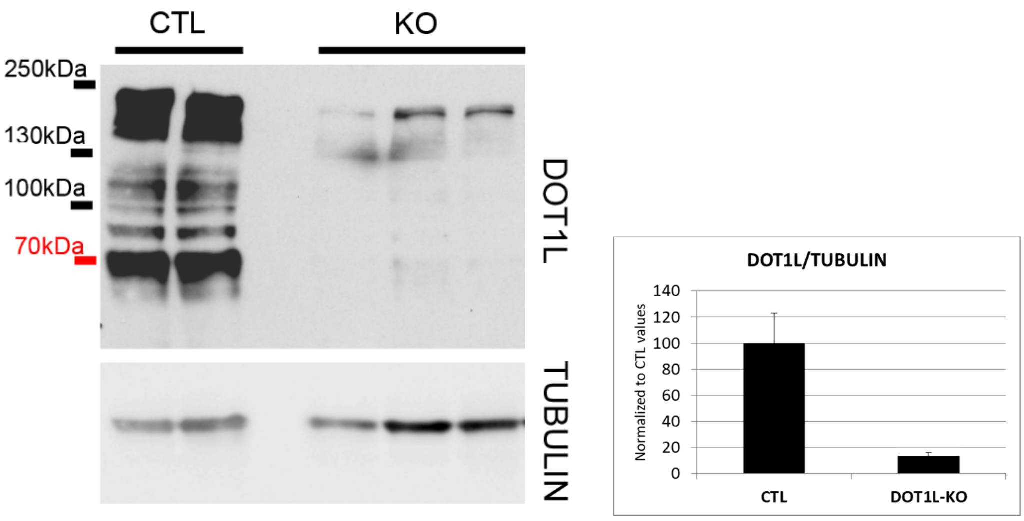
CTL
KO
250kDa
130kDa
100kDa
70kDa
DOT1L
TUBULIN
DOT1L/TUBULIN
Normalized to CTL values
140
120
100
80
60
40
20
0
CTL
DOT1L-KO

1F

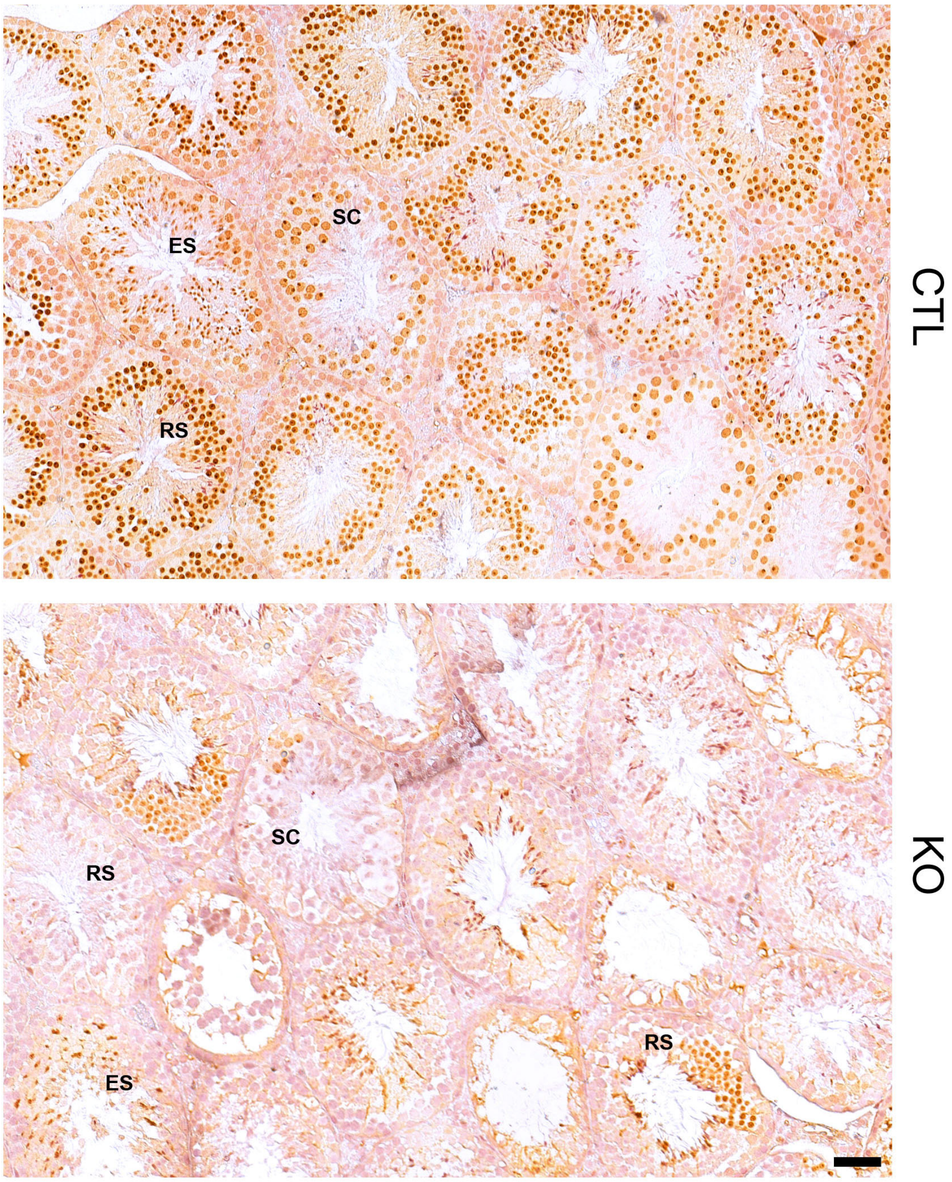

1G

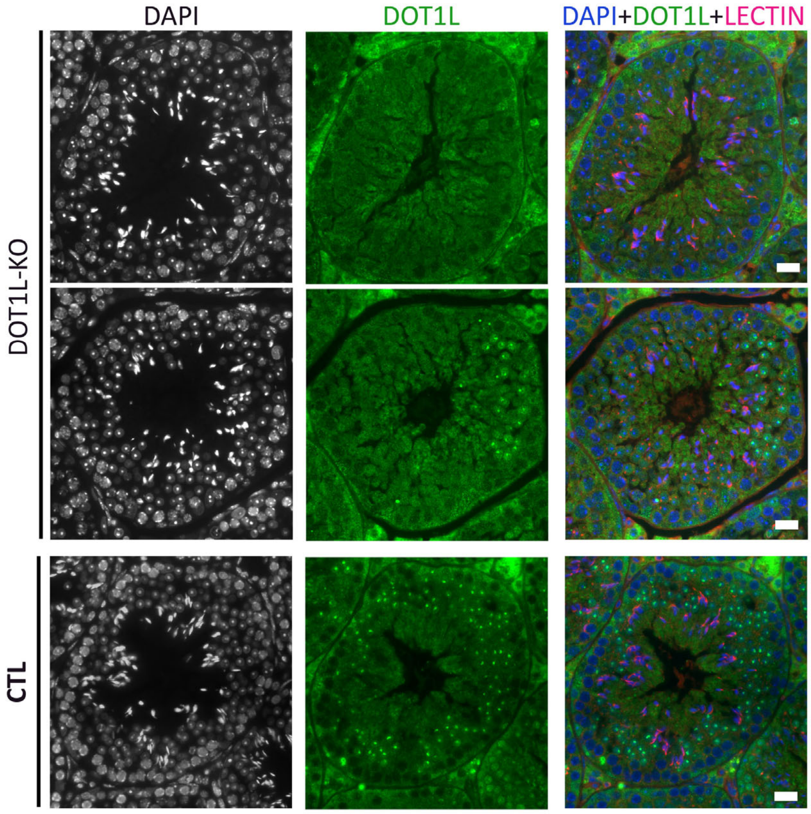
DAPI
DOT1L
DAPI+DOT1L+LECTIN
DOT1L-KO
CTL

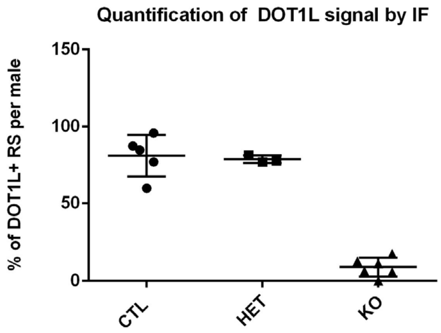
Quantification of DOT1L signal by IF
% of DOT1L+ RS per male
150
100
50
0
CTL
HET
KO

1H

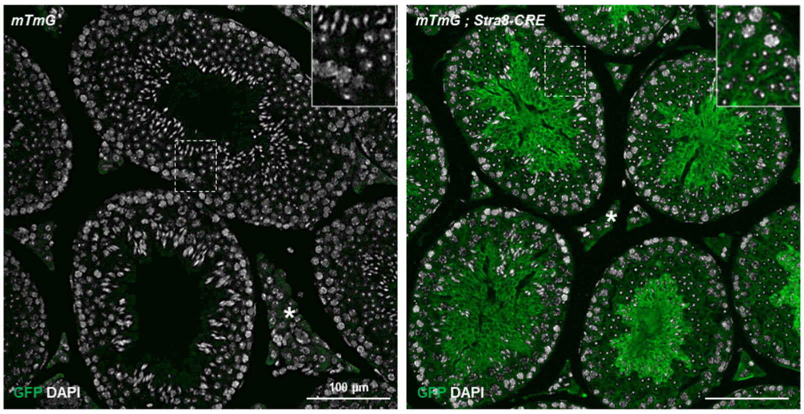
mTmG
mTmG ; Stra8-CRE
GFP DAPI
GFP DAPI
100 µm

1I

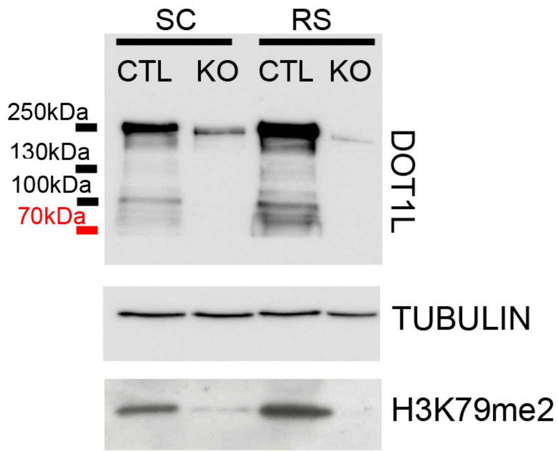

Appendix Figure S1. Complement to Figure 1. A) RNA expression overview of *DOT1L* RNA in human and mouse tissues. Data were obtained from The Human Protein Atlas [https://www.proteinatlas.org/; consensus dataset, consisting of normalized expression (nTPM) levels for 55 tissue types, created by combining the HPA and GTEx transcriptomics datasets] or from mouse tissues from mouse ENCODE transcriptome data (Data ref: Mouse ENCODE transcriptome data, NCBI Sequence Read Archive PRJNA66167, 2011). **B) Expression of *Dot1l* mRNA based on single cell RNA-seq analyses from adult testicular cells** (Data ref: (Green *et al*, 2018) analyzed using https://rgv.genouest.org/ (Darde *et al*, 2019; Darde *et al*, 2015)). **C) Summary of DOT1L protein expression in mouse germ cells based on immunofluorescence detection on testicular sections. Scheme modified after Russell et al. (Russell *et al*, 1990).** DOT1L protein is detected in all germ cells but is particularly abundant in late spermatocytes and in round spermatids. **D) Generation of *Dot1l* KO allele.** Drawing representing the beginning of mouse *Dot1l* gene (from exon 1 to exon 3), the location of loxP sites and genotyping primers. Bottom right insert shows typical pictures of products from PCR using *Dot1l* genotyping primers (F+R1+R2) following run on a 2% agarose gel. **E) Estimation of *Dot1l* knockout efficiency by western blot (uncropped western blot image of Figure 1B).** Detection of DOT1L and TUBULIN in whole testicular protein extracts from CTL and *Dot1l*-KO mice. The most abundant and high molecular weight bands are DOT1L testis-specific (~185KDa) and canonical proteoform. Additional bands most likely corresponding to smaller, less abundant proteoforms (such as Q679P5 at ~122KDa or Q6XZL7 at ~68KDa) are also visible in case of long exposure. All DOT1L proteoforms are markedly reduced in *Dot1l*-KO testicular extracts. On the right is shown a graphic representation of the quantification of DOT1L signal (all bands) normalized to TUBULIN signal**. F) Immunohistochemistry detection of DOT1L in testicular sections from CTL and *Dot1l*-KO (KO) adult mice counterstained with hematoxylin (complement panel to Fig 1C).** SC = Spermatocytes, RS = Round spermatids, ES = Elongating spermatids. Pictures were taken using the same parameters. Scale bars indicate 50 µm. **G) Estimation of *Dot1l* knockout efficiency by immunofluorescence.** Representative pictures of immunofluorescence detection of DOT1L (Abcam 64077 antibody, in green) on testicular sections from adult CTL or KO mice. DAPI (grey or blue) was used to stain nuclei; Lectin (pink) was used to stain acrosome. Scale bar indicates 20µm. In CTL testicular sections, tubules containing round spermatids are uniformly stained (bottom panel). In *Dot1l*-KO sections, the majority of tubules do not show any DOTL1 protein signal (top panel) but in some tubules, DOT1L signal can still be visible (middle panel). Below the immunofluorescence pictures is shown a graphic representation of the percentage of round spermatids with a visible DOT1L signal. N=5 CTL, 3 HET and 6 KO males between 2.5-4 month old (6 to 10 tubules per individual). Quantification was performed on stages I to V tubules. **H) Verification of *Stra8-Cre* recombinase activity and specificity in adult mouse testis.** The Cre reporter mouse line *mTmG* carries a *GFP* transgene expressed upon Cre induction. In this transgene, GFP is targeted to the cell membrane (Muzumdar *et al*, 2007). In testicular sections from *mTmG* mice (left panel), using an anti-GFP antibody, no signal is detected, excepted for faint auto-fluorescence from Leydig cells (asterisk). In *mTmG ; Stra8-Cre* mice (right panel) the Cre recombinase is specifically expressed in germ cells and GFP (in green) is detected in most germ

cells. DAPI (grey) has been used to stain cell nuclei. Top right panel represents a 2X magnification. **I) Uncropped western blot image of Fig 1D.** Note that the membrane was cut at ~70KDa and ~35KDa to generate three pieces which were incubated with anti-DOT1L antibody (top part), with anti-TUBULIN antibody (mid part) or with anti-H3K79me2 antibody (bottom part).

Appendix Figure S2

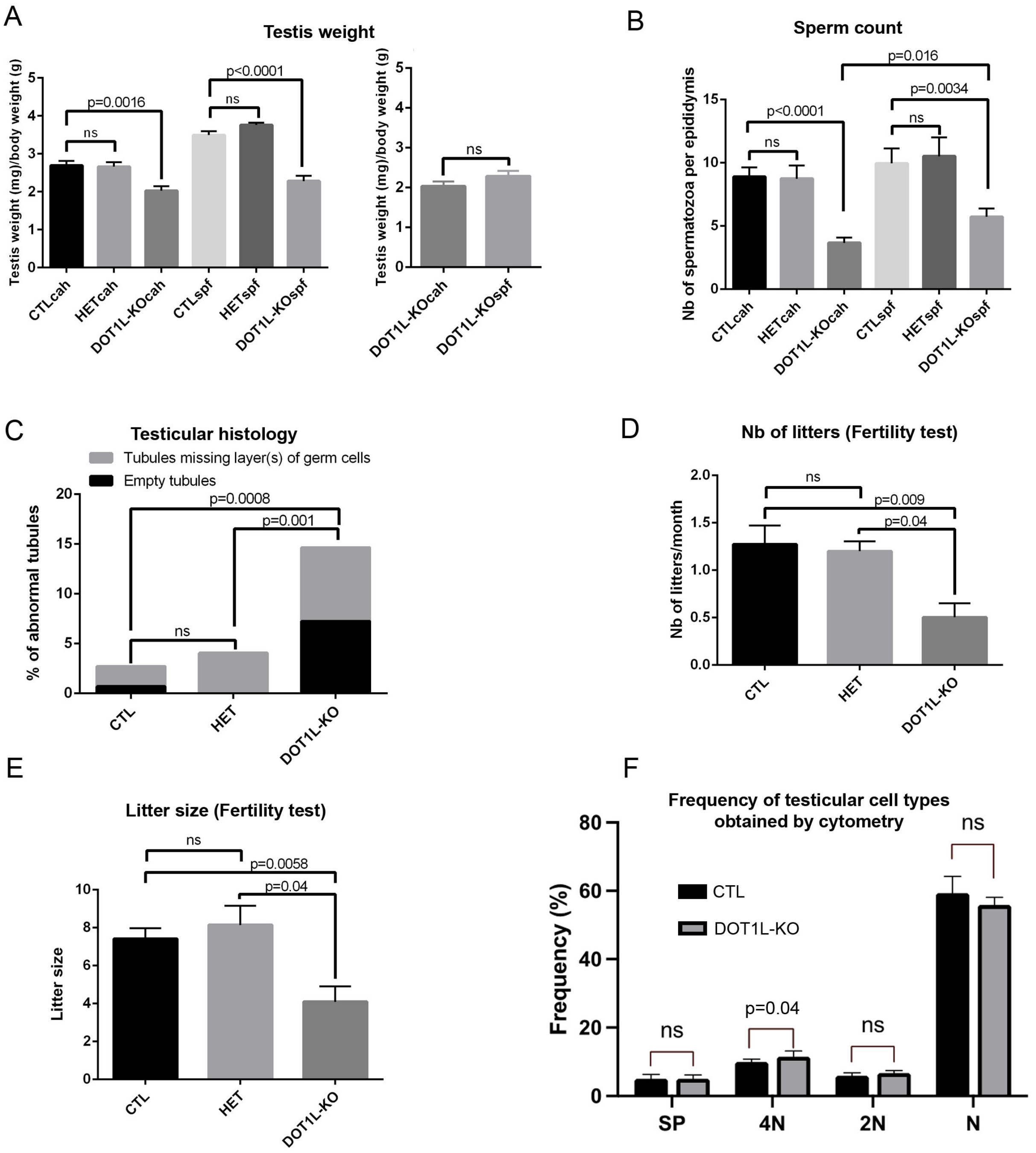

G

In vitro fertilization

	**% of oocytes in vitro fertilized (nb of fertilized/total)**
WT 1	52.4 (11/21)
WT 2	34.8 (24/69)
CTL 1	36.7 (18/49)
CTL 2	21.9 (14/64)
HET 1	37.5 (9/24)
DOT1L-KO 1	0 (0/37)
DOT1L-KO 2	0 (0/29)
DOT1L-KO 3	0 (0/27)
DOT1L-KO 4	0 (0/87)

Appendix Figure S2. Complement to Figure 2. Detailed analyses of reproductive parameters from *Dot1l*-KO males and CTL. 'cah' indicates animals produced in a conventional animal house. 'spf' indicates animals produced in the specific pathogen-free animal house. HET are heterozygous animals of the following genotype $Dot1l^{Fl/\Delta}$. Obtained p-values (following unpaired t-tests) are indicated above; "ns" indicates non-significant difference. **A**). Average testis weight (normalized to body weight, mean value ± SEM) in different groups of males (N=9 for CTLcah, N=6 for HETcah, N= 9 for DOT1Lcah, N=12 for CTLspf, N=2 for HETspf and N= 17 for DOT1Lspf). **B**) Average number of epididymal spermatozoa (mean value ± SEM) in different groups of males (N=12 for CTLcah, N=6 for HETcah, N=12 for DOT1Lcah, N=9 for CTLspf, N=2 for HETspf and N= 13 for DOT1Lspf). **C**) Graphic representation of the percentage of abnormal tubules in *Dot1l*-KO (N=5) CTL (N=6) and HET (N=4) testes (from both cah and spf). Obtained p-values (following unpaired t-tests on empty tubules values) are indicated above. **D**) and **E**) Results from the tests of fertility (natural mating in spf with wild type females for ~3 months) of *Dot1l*-KO, HET and CTL males (mean ± SEM; N=7 for KO, N= 2 for HET and N=6 for CTL). **F**) Schematic diagram representing the frequency of each cell type in CTL and *Dot1l*-KO testes, as calculated by FACS (mean ± SEM; N= for CTL and 3 for KO). 4N = Primary spermatocytes, 2N = Secondary spermatocytes, N = spermatids, SP = side population representing premeiotic germ cells (i.e. spermatogonia). **G)** Results from *in vitro* fertilization assays. The percentage of fertilized oocytes is indicated for each male. Values obtained for *Dot1l*-KO spermatozoa are significantly different from WT or CTL spermatozoa (chi-square, p<0.01).

Appendix Figure S3

3A

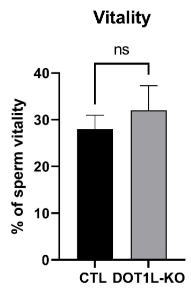

3B

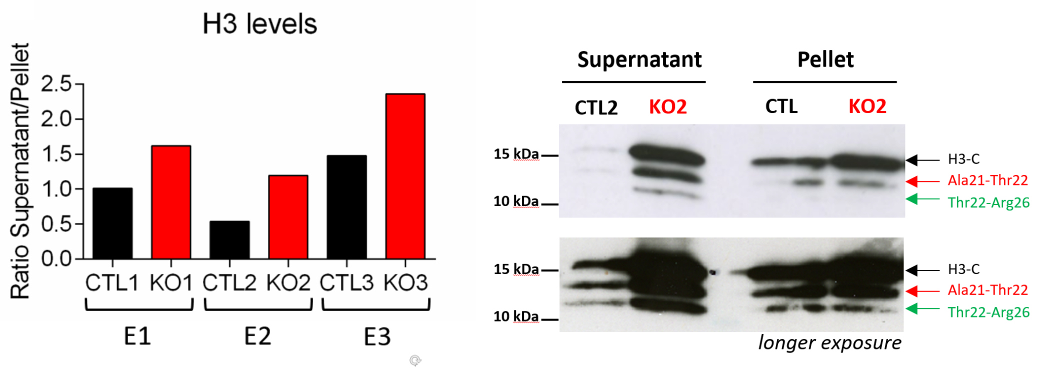

3C

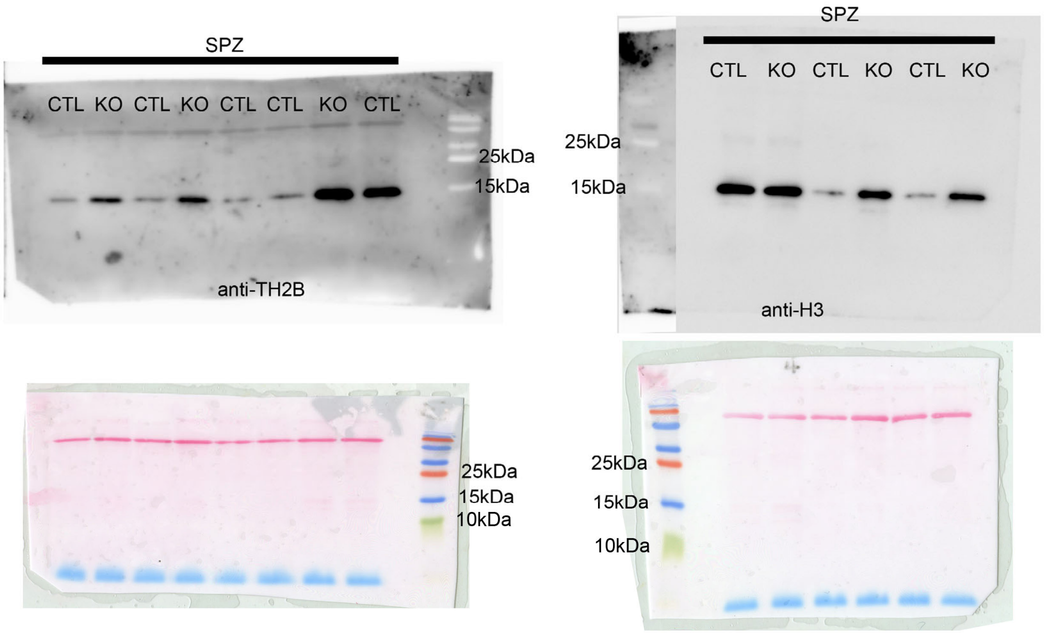

3D

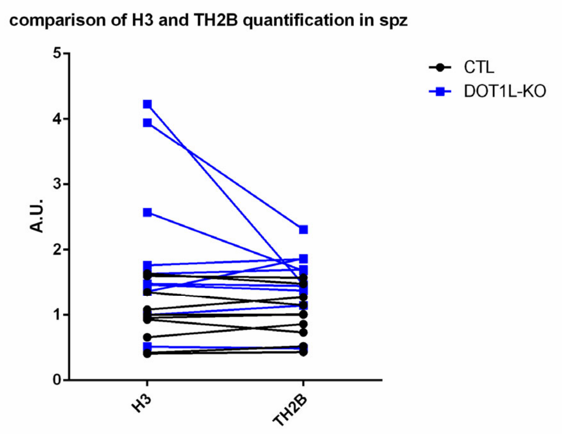

3E

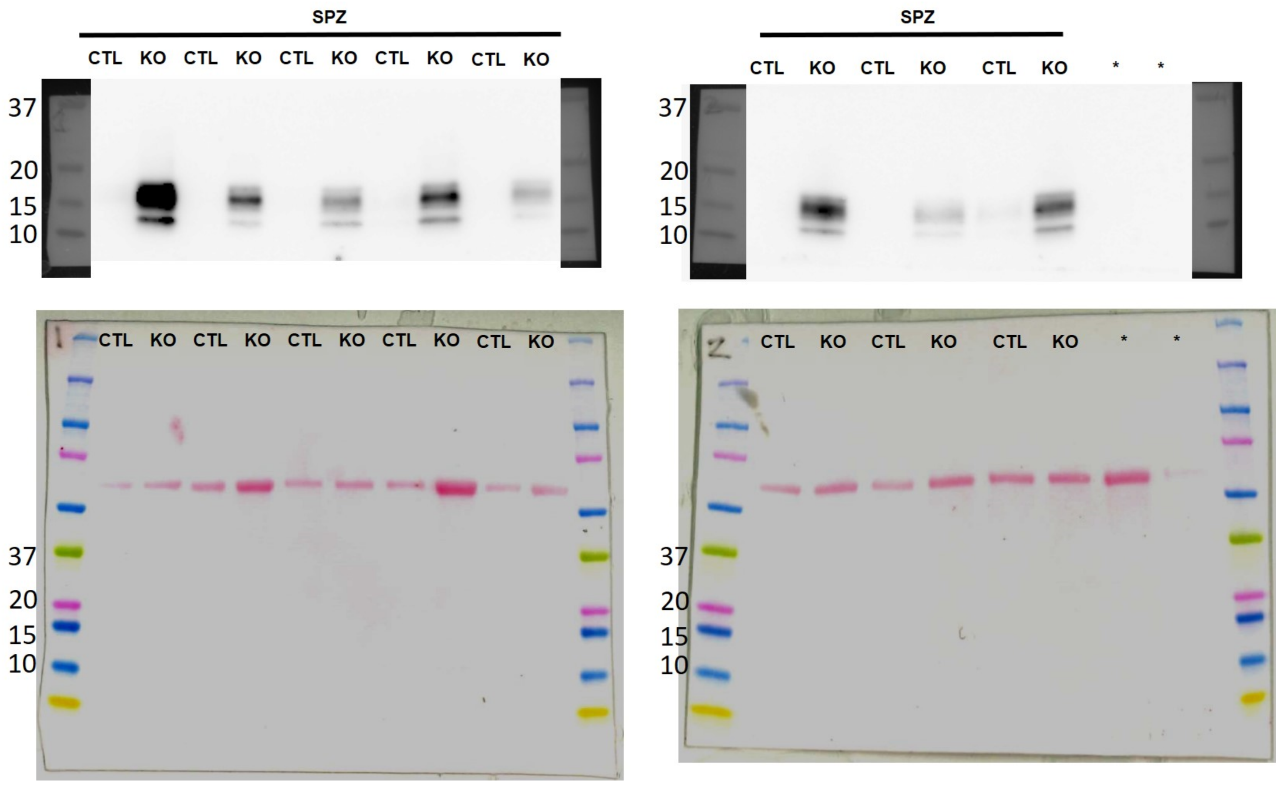
SPZ
CTL KO CTL KO CTL KO CTL KO CTL KO
37
20
15
10
SPZ
CTL KO CTL KO CTL KO * *
37
20
15
10
CTL KO CTL KO CTL KO CTL KO CTL KO
37
20
15
10
CTL KO CTL KO CTL KO * *
37
20
15
10

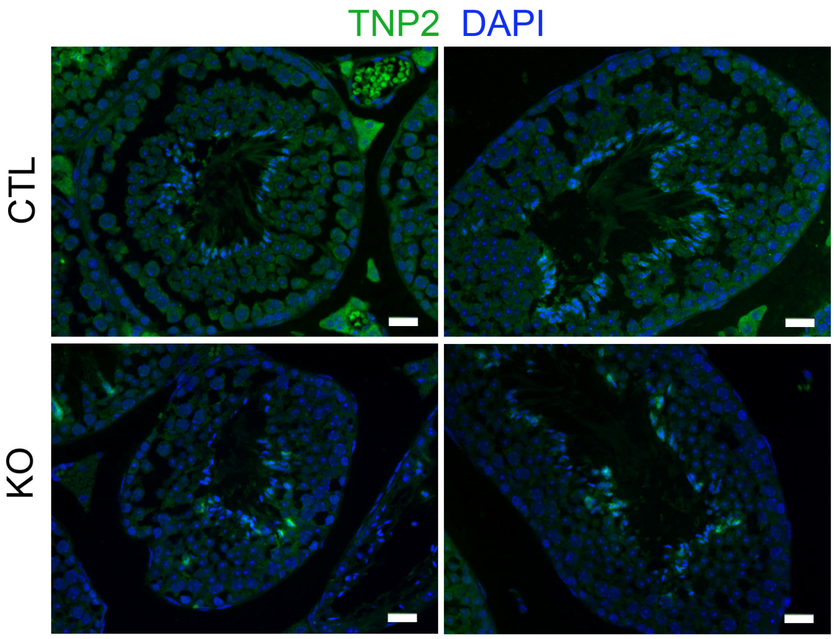
TNP2 DAPI
CTL
KO

3F

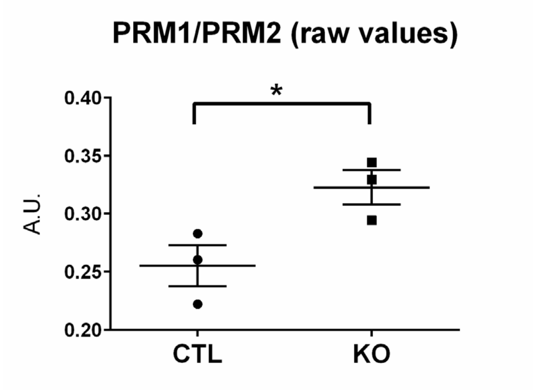

Appendix Figure S3. Complement to Figure 3. A) Sperm vitality. Bar graph showing sperm vitality measured by eosin-nigrosine staining in which dead spermatozoa appear violet (mean ± SEM). Sperm samples from 4 CTL and 4 *Dot1l*-KO males were analyzed. NS indicates non-significant difference between the two genotypes (t-test). **B) *Dot1l*-KO sperm chromatin is sensitive to nucleoplasmin-induced decompaction.** Quantification of histone H3 levels following NPM (nucleoplasmin) partial decondensation of CTL and *Dot1l*-KO (KO) epididymal spermatozoa. Quantification was performed on the two fractions obtained after centrifugation: (i) the supernatant which contains histones from the NPM-de-compacted chromatin, and ii) the pellet which contains compact chromatin, i.e. “resistant” to the NPM treatment we used. Left panel: Bar graph showing quantification results of supernatant/pellet levels in 3 independent experiments (E1, E2 and E3, which are biological replicates). Right panel: Images of western blot using anti-histone H3 antibody (PanH3), corresponding to CTL2 and KO2 (the bottom image was used for quantification). PanH3 is directed against the C-terminal part of histone H3 and detects full length histone H3 at 17 kDa (black arrow, H3-C) and its two cleaved forms previously described in (Yamaguchi et al., 2018): one occurring between Ala21-Thr22 (red arrow) and the other one between Thr22-Arg26 (the smallest form at 13 kDa, green arrow). **C)** Uncropped western blot images from TH2B and H3 detections in spermatozoa (shown in Fig 3E), and corresponding Ponceau staining of the membranes. A non-specific band is visible in all lanes with Ponceau; it indicates that similar amounts of material were loaded in all lanes. **D)** Comparison of TH2B and H3 levels in the same CTL (black) and *Dot1l*-KO (blue) samples. The graph shows a good correlation between TH2B and H3 quantifications. **E)** Top panel: uncropped western blot images from TNP2 detection in spermatozoa (shown in Fig 3F), and corresponding Ponceau staining of the membranes. A non-specific band is visible in all lanes with Ponceau; it indicates that similar quantity of material was loaded in all lanes. Bottom panel: immunofluorescence detection of TNP2 (green) on stage VI-VIII testicular sections. At this stage, the chromatin of condensed spermatids is packaged with protamines, and TNP2 signal is not (or weak) in CTL. In *Dot1l*-KO, some condensed spermatids (inside of the tubule) have a stronger signal. DAPI (blue) was used to stain nuclei. **F)** Box plot of the raw PRM1/PRM2 ratio following acid urea gel electrophoresis (mean ± SEM) complement to Fig 3G. A.U. = arbitrary units. *p-value <0.05 (unpaired t-test).

Appendix Figure S4

4A

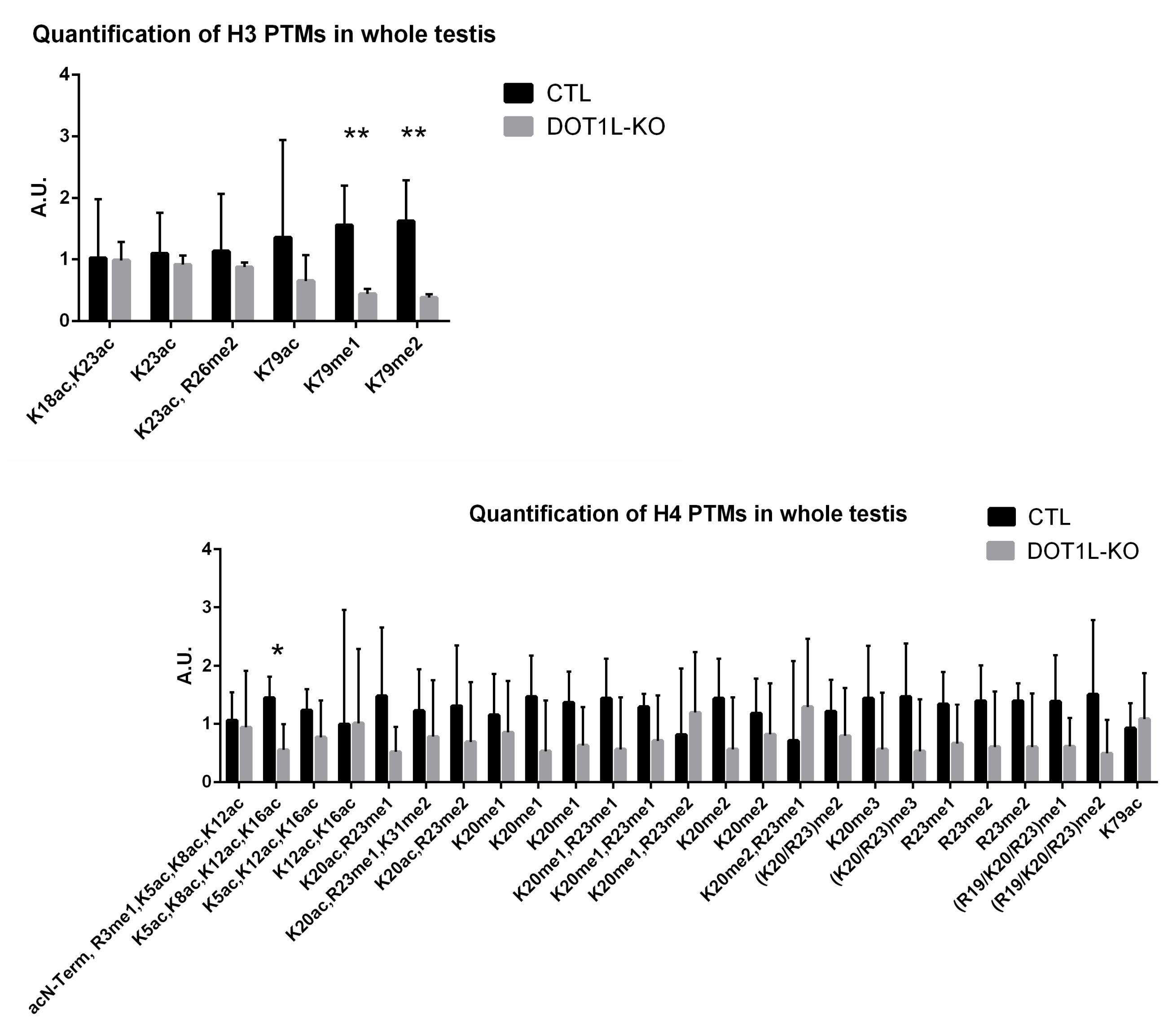

4B

H2A PTMs in ES

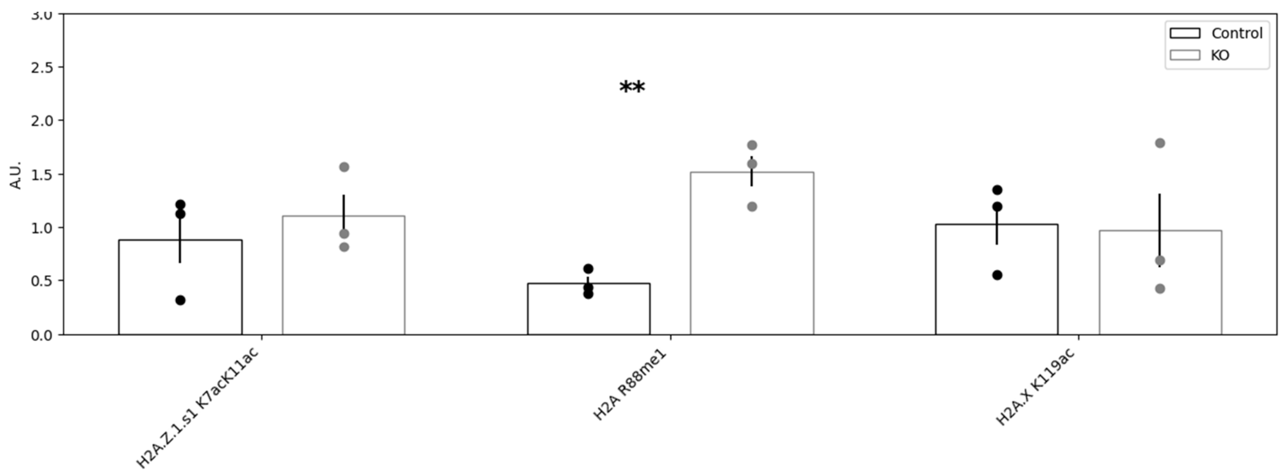

H2B PTMs in ES

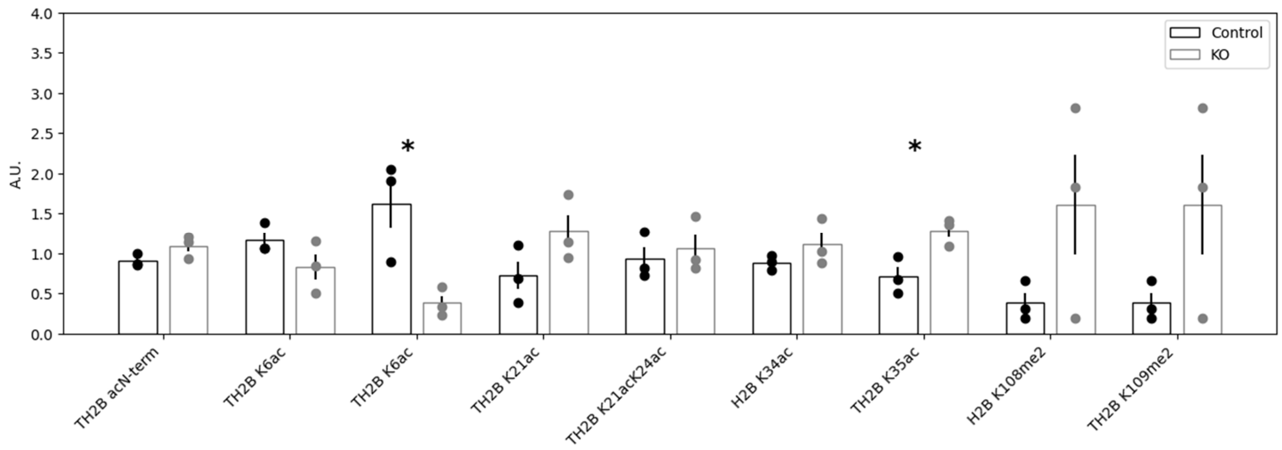

4C

Histone variant levels in ES

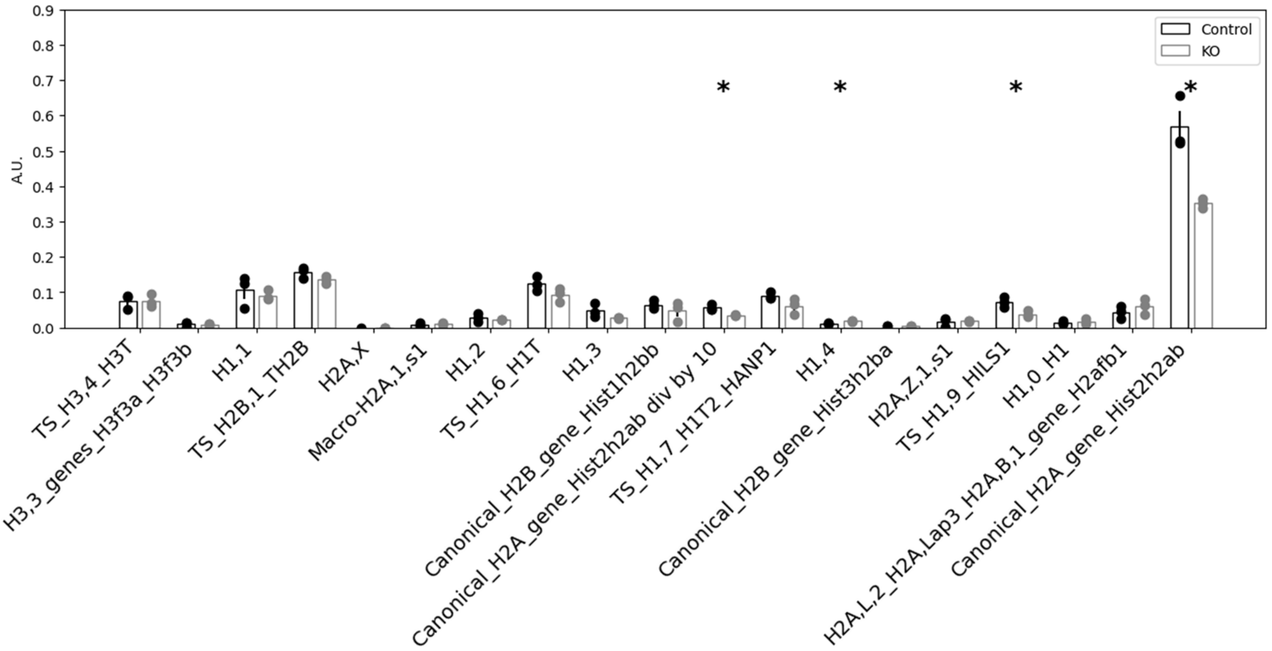

Histone variant levels in RS

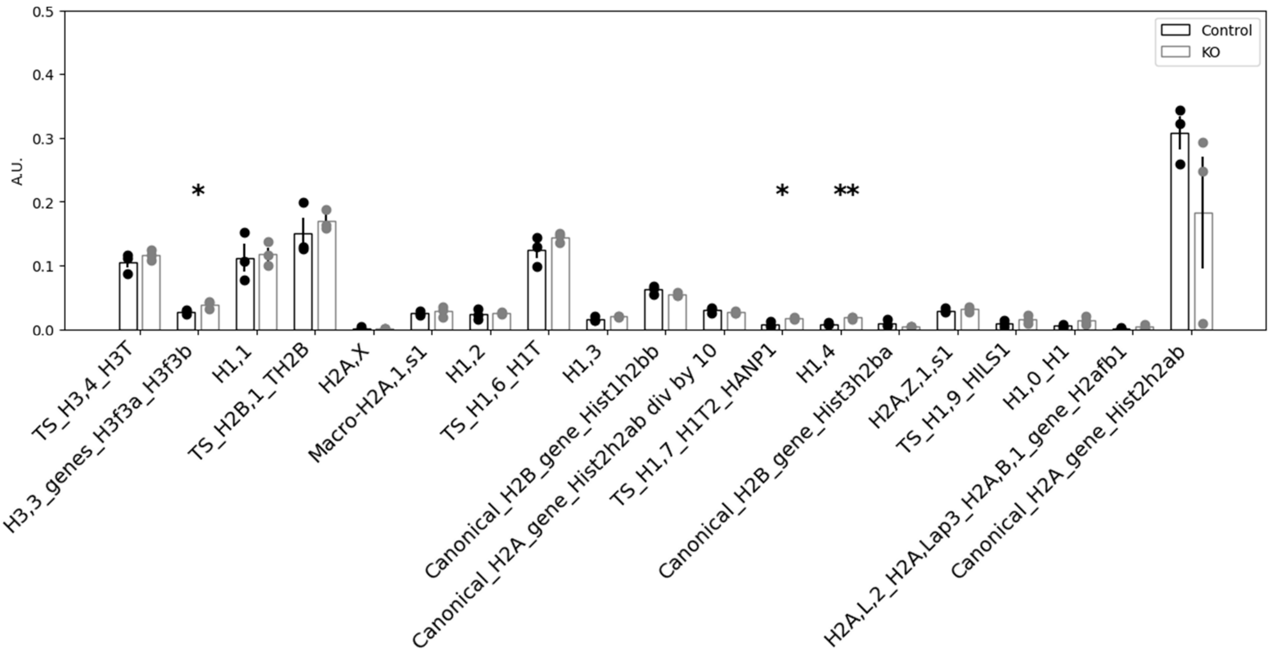

4D

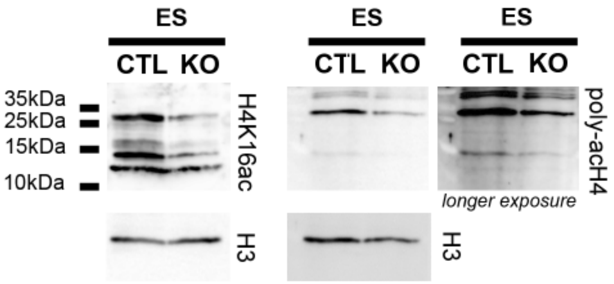
ES
CTL KO
35kDa
25kDa
15kDa
10kDa
H4K16ac
H3
ES
CTL KO
H3
ES
CTL KO
poly-acH4
longer exposure

4E

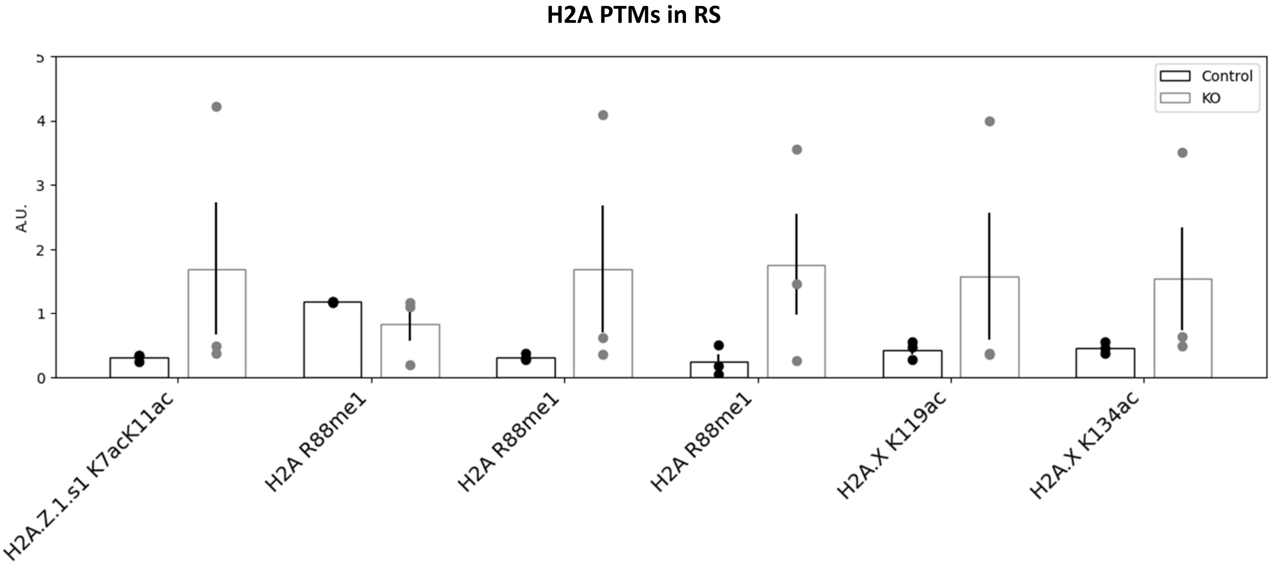
H2A PTMs in RS
A.U.
Control
KO
H2A.Z.1.s1 K7acK11ac
H2A R88me1
H2A R88me1
H2A R88me1
H2A.X K119ac
H2A.X K134ac

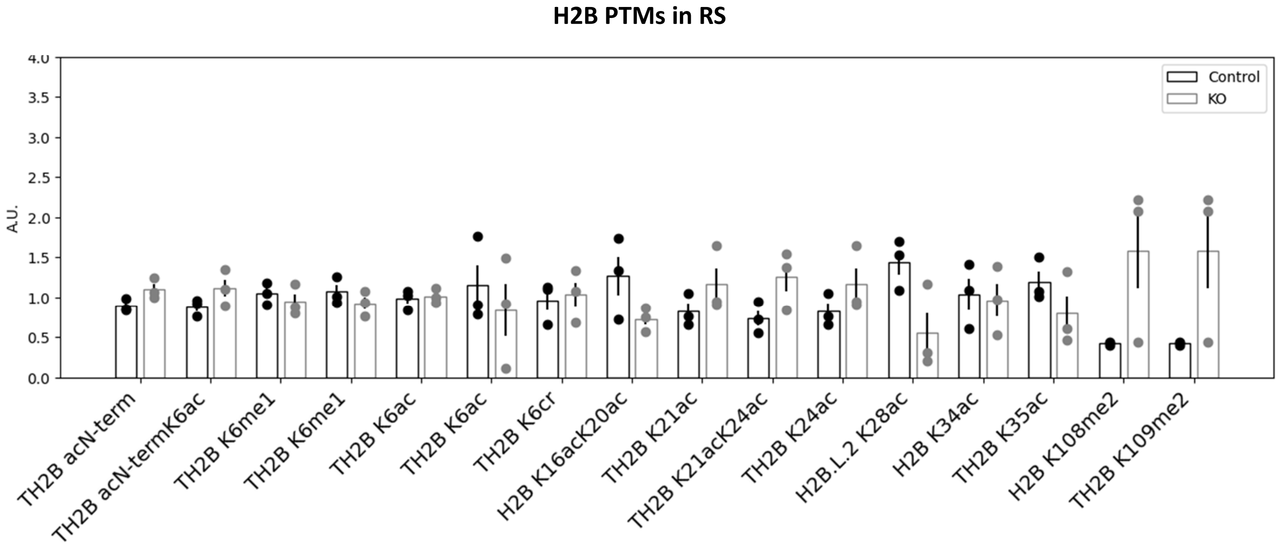
H2B PTMs in RS
A.U.
Control
KO
TH2B acN-term
TH2B acN-termK6ac
TH2B K6me1
TH2B K6me1
TH2B K6ac
TH2B K6ac
TH2B K6cr
H2B K16acK20ac
TH2B K21ac
TH2B K21acK24ac
TH2B K24ac
H2B.L.2 K28ac
H2B K34ac
TH2B K35ac
H2B K108me2
TH2B K109me2

4F

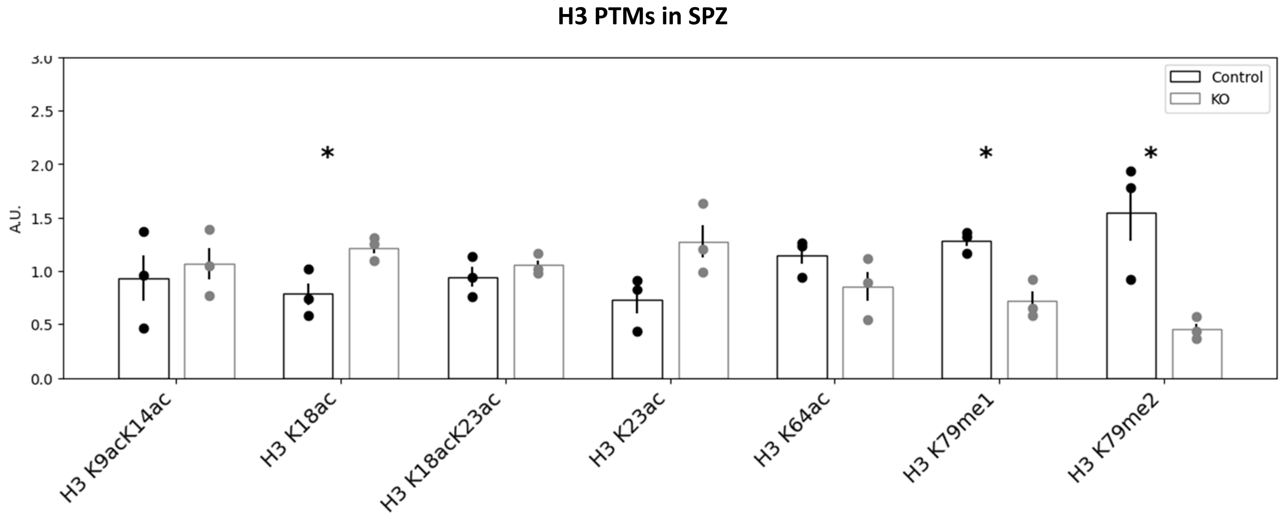

Appendix Figure S4. Complement to Figure 4. Quantification of histone PTMs and variants in *Dot1l*-KO and CTL samples. One star indicates a p-value <0.05, two stars a p-value<0.005 and three stars a p-value <0.0005, obtained with t-tests. **A) Mass spectrometry quantification of H3 and H4 PTMs in whole testes (N= 4 CTL and 4 KO).** After normalization to be at constant amount of histone H3 or H4 in each analyzed sample (see Material and methods), mass spectrometry signals were divided by the average signal in both conditions (CTL and *Dot1l*-KO), so as to be able to represent all peptides in the same figure, whatever their MS intensity. Error bars represent standard deviations calculated on the measurements made on biological replicates. Each modified peptide identified and quantified by MS analysis is defined as a list of PTMs separated by commas. Some modified sites were detected and quantified in several tryptic peptides due to possible missed cleavages (e.g. H4K20me1 was quantified in K20VLRDNIQGITK, K20VLRDNIQGITKPAIR and K20VLRDNIQGITKPAIRR); we indicated the measurements made on such peptides with the same label (K20me1 in the aforementioned example), which allows verifying the coherence of quantitative measurements. In a few instances, a precise localization of the modification site was not possible from the MS/MS spectra. This was typically the case between H4R19, K20 and R23 on which a methyl (me1) and dimethyl (me2) were detected. We indicated such uncertainties by (R19/K20/R23)me1 and (R19/K20/R23)me2, respectively. **B) Mass spectrometry quantification of H2A and H2B in elongating/condensing spermatids (ES) (N=3 CTL and 3 KO).** LC-MS/MS data acquired on H2A and H2B were processed as described above for H3 and H4 in whole testes, except that MS signals detected on modified peptides were normalized to be at constant amount of H2A or H2B in each analyzed sample. **C) Mass spectrometry quantification of histone variants in elongating/condensing spermatids (ES) and in round spermatids (RS).** The abundance of each histone variant was estimated from the sum of intensities of its identified specific proteolytic peptides. The abundance of each protein was then normalized to be at constant amount of histone H4 between samples. For some variants, MS values were divided by 10, so as to be able to visualize all the proteins on the same figure, whatever their MS intensity. Error bars represent standard deviations calculated on the measurements made on biological replicates. **D) Confirmation of a decrease in H4 acetylation in elongating spermatids (ES)**. Western blot detection of H4K16ac, poly-H4ac and H3. Higher molecular weight bands likely represent non-denatured forms of histone H4. Western blot normalization was performed by detecting the same membranes with anti-H3 antibody. Anti-H3 rather than anti-H4 antibody was chosen to avoid interference with acetylated H4 signal. Histones H3 and H4 are organized as obligate tetramers (or occasionally heterodimers) in nucleosomes; therefore, they are expected to be present in the chromatin in the same quantity. **E) Mass spectrometry quantification of histone H2A and H2B PTMs in round spermatids (N= CTL and 3 KO).** LC-MS/MS data acquired on H2A and H2B were processed as described above for ES. **F) Mass spectrometry quantification of histone H3 PTMs in spermatozoa (N= 4 CTL and 4 KO).** Same legend as above. H4 PTMs levels were too weak to be quantified.

Appendix Figure S5

5A

Deregulated genes identified with DEseq2, with a Fold change > 1.5 and FDR < 0.05

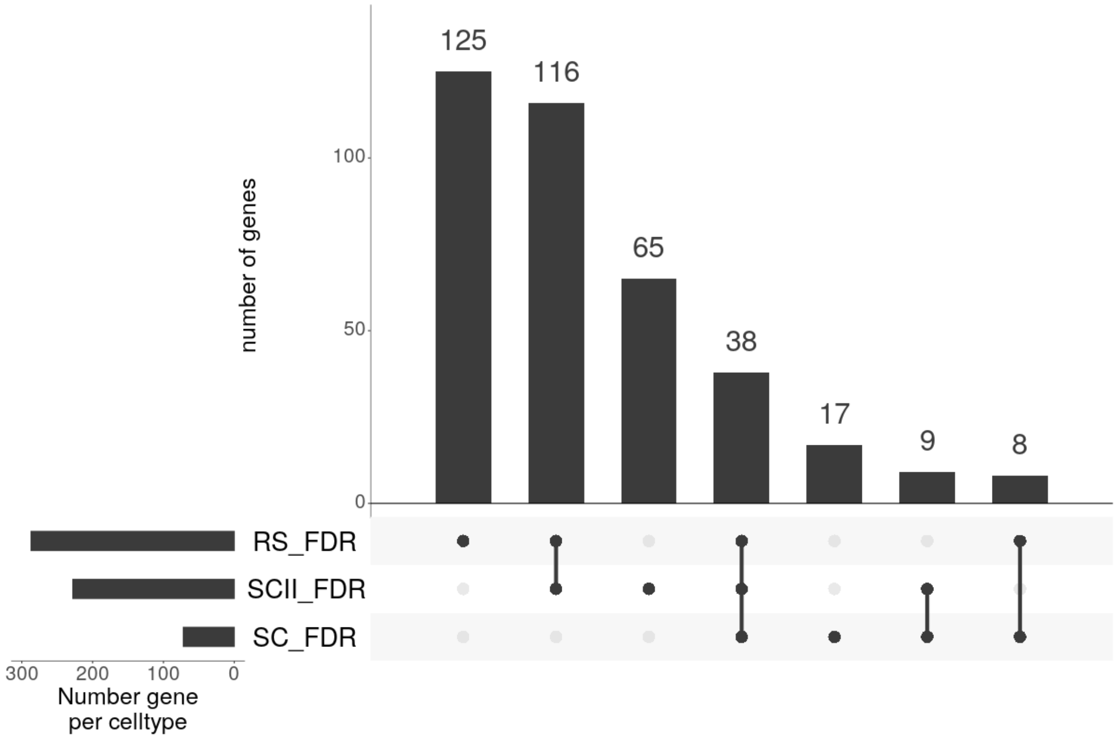

Deregulated genes identified with DEseq2, with a Fold change > 1.5 and p.value < 0.05

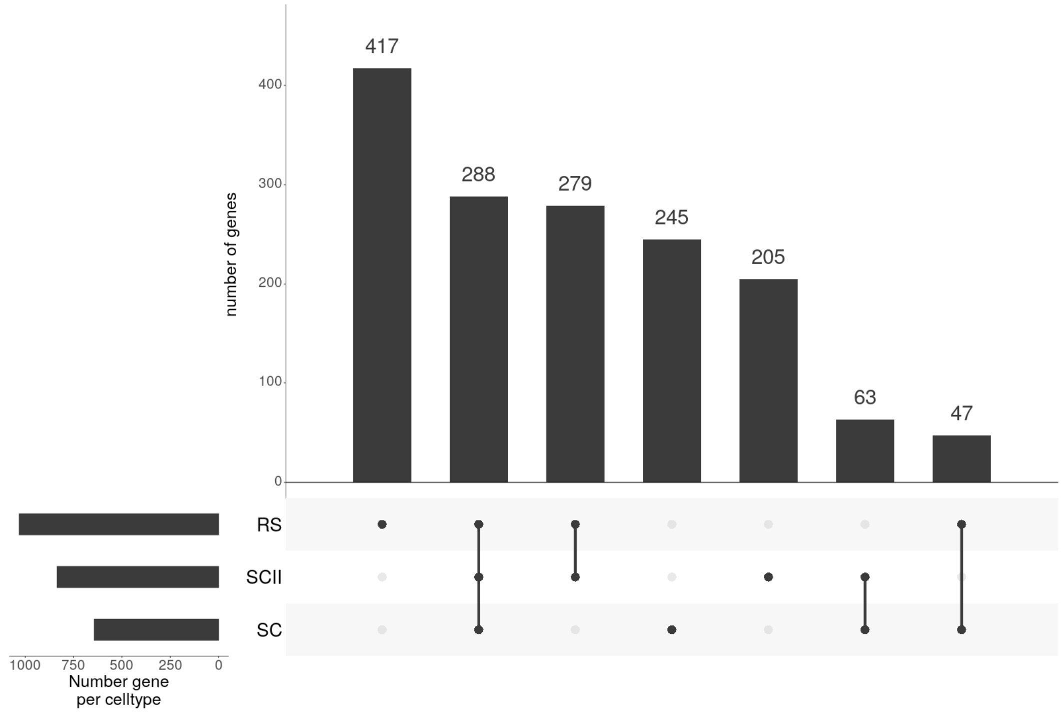

5B

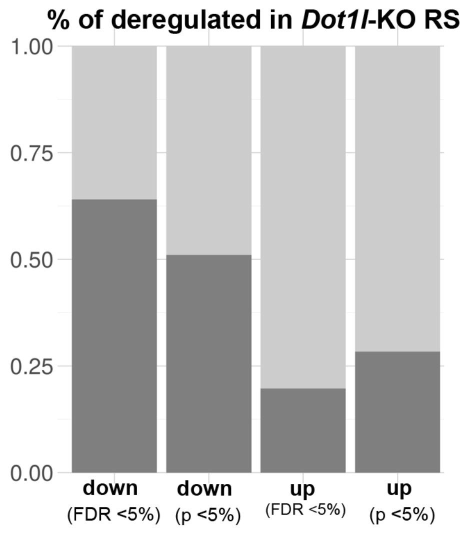

5C

gene_name	logFC	logCPM	PValue	FDR	gl	H3K79me2 enrichment
Slc10a3	-1.26745740995669	0.999004290396908	0.00390006777314618	0.1545955595244	Down-regulated	yes
Slc6a8	-2.46727862572467	0.52781470462503	7.72654921275588e-07	0.000364372904035715	Down-regulated	yes
Slc7a2	-1.0665290941501	2.27338480123705	0.0150834110914719	0.387940102031817	Down-regulated	no
Slc12a1	1.3955625700193	0.681122253169346	0.000169908367523562	0.0174799371397939	Up-regulated	no
Slc13a4	1.54000399067509	2.76366740898566	4.27870310972635e-05	0.00657135254678289	Up-regulated	no
Slc18a2	1.25758541410194	-0.793930612861824	0.00237996137428981	0.110886263192696	Up-regulated	no
Slc1a4	1.10997163431429	0.860924966152978	0.00103762614100171	0.0601552770604185	Up-regulated	no
Slc1a5	1.06878712737505	2.2418481205103	0.00176357549579316	0.0883504245162868	Up-regulated	yes
Slc25a23	0.926874320430452	2.4842629146059	0.00744458216430107	0.240033828551142	Up-regulated	yes
Slc25a37	0.83494237600095	2.8363663126306	0.0494170665013017	0.856402093077086	Up-regulated	no
Slc25a48	0.844791121775937	0.601566750878029	0.035227161221668	0.691260215461133	Up-regulated	yes
Slc26a11	1.65452313717142	1.74006812886016	0.000661650590507997	0.044270891766396	Up-regulated	yes
Slc28a2	0.882471439124064	0.348580484195426	0.0475801028844169	0.835942882356513	Up-regulated	no
Slc29a3	1.00130602592956	3.99897275785481	0.024747552656584	0.549198182271674	Up-regulated	yes
Slc37a3	0.864250867428252	1.06283489334379	0.0157601580162183	0.398622878559951	Up-regulated	no
Slc38a2	1.19621193912572	2.2217849521669	0.000373937024375424	0.0301146206327321	Up-regulated	yes
Slc38a8	1.03918406075334	-0.0156846590541735	0.0201914826533337	0.472852642452675	Up-regulated	no
Slc45a1	1.75519777185066	-0.803109163412898	0.000635433125120995	0.0430016682924086	Up-regulated	no

5D

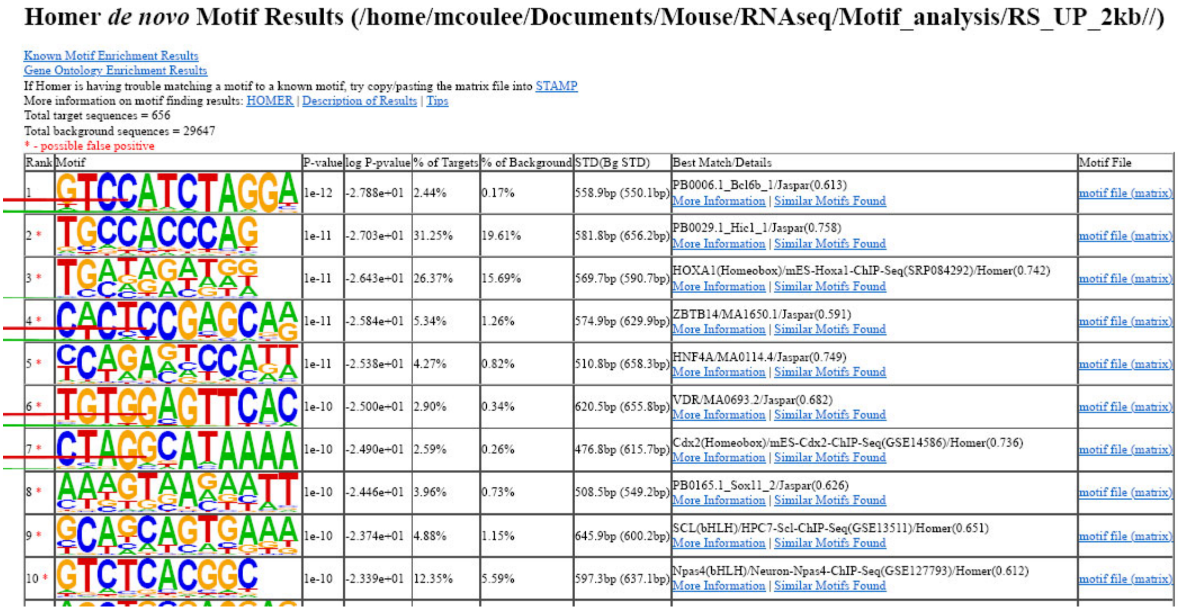

Homer *de novo* Motif Results (/home/mcoulee/Documents/Mouse/RNAseq/Motif_analysis/RS_UP_2kb//)

Known Motif Enrichment Results
Gene Ontology Enrichment Results
If Homer is having trouble matching a motif to a known motif, try copy/pasting the matrix file into STAMP
More information on motif finding results: HOMER | Description of Results | Tips
Total target sequences = 656
Total background sequences = 29647
* - possible false positive

Rank	Motif	P-value	log P-pvalue	% of Targets	% of Background	STD(Bg STD)	Best Match/Details	Motif File
1	GTCCATCTAGGA	1e-12	-2.788e+01	2.44%	0.17%	558.9bp (550.1bp)	PB0006.1_Bcl6b_1/Jaspar(0.613) More Information | Similar Motifs Found	motif file (matrix)
2 *	TGCCACCCAG	1e-11	-2.703e+01	31.25%	19.61%	581.8bp (656.2bp)	PB0029.1_Hic1_1/Jaspar(0.758) More Information | Similar Motifs Found	motif file (matrix)
3 *	TGATAGATGG	1e-11	-2.643e+01	26.37%	15.69%	569.7bp (590.7bp)	HOXA1(Homeobox)/mES-Hoxa1-ChIP-Seq(SRP084292)/Homer(0.742) More Information | Similar Motifs Found	motif file (matrix)
4 *	CACTCCGAGCAA	1e-11	-2.584e+01	5.34%	1.26%	574.9bp (629.9bp)	ZBTB14/MA1650.1/Jaspar(0.591) More Information | Similar Motifs Found	motif file (matrix)
5 *	CCAGAGTCCATT	1e-11	-2.538e+01	4.27%	0.82%	510.8bp (658.3bp)	HNF4A/MA0114.4/Jaspar(0.749) More Information | Similar Motifs Found	motif file (matrix)
6 *	TGTGGAGTTCAC	1e-10	-2.500e+01	2.90%	0.34%	620.5bp (655.8bp)	VDR/MA0693.2/Jaspar(0.682) More Information | Similar Motifs Found	motif file (matrix)
7 *	CTAGGCATAAAA	1e-10	-2.490e+01	2.59%	0.26%	476.8bp (615.7bp)	Cdx2(Homeobox)/mES-Cdx2-ChIP-Seq(GSE14586)/Homer(0.736) More Information | Similar Motifs Found	motif file (matrix)
8 *	AAAGTAAGAATT	1e-10	-2.446e+01	3.96%	0.73%	508.5bp (549.2bp)	PB0165.1_Sox11_2/Jaspar(0.626) More Information | Similar Motifs Found	motif file (matrix)
9 *	GCAGCAGTGAAA	1e-10	-2.374e+01	4.88%	1.15%	645.9bp (600.2bp)	SCL(bHLH)/HPC7-Scl-ChIP-Seq(GSE13511)/Homer(0.651) More Information | Similar Motifs Found	motif file (matrix)
10 *	GTCTCACGGC	1e-10	-2.339e+01	12.35%	5.59%	597.3bp (637.1bp)	Npas4(bHLH)/Neuron-Npas4-ChIP-Seq(GSE127793)/Homer(0.612) More Information | Similar Motifs Found	motif file (matrix)

gene_name	logFC	logCPM	PValue	FDR	gl	H3K79me2 enrichment
Cacna1e	1.381540782	2.204690766	0.002726556	0.121318141	Up-regulated	Yes
Gpsm1	1.115505708	1.018472463	0.002648857	0.119051423	Up-regulated	No
Tmem74b	1.021769193	0.247820597	0.013177832	0.354170753	Up-regulated	No
Gata5	1.673646912	-1.21958131	0.000102450	0.012941114	Up-regulated	No
Padi1	1.006558376	-0.52514070	0.027404915	0.589073299	Up-regulated	No
Ptpn13	0.896515079	3.191552902	0.026370274	0.572363590	Up-regulated	No
Megf8	0.875273628	5.337855272	0.023642554	0.532645802	Up-regulated	No
Kctd21	0.837477696	0.230864728	0.024428532	0.543473782	Up-regulated	No
Irf1	1.521852615	0.397338881	0.006670929	0.224722281	Up-regulated	No
Lhfpl2	1.068600482	-0.60887569	0.028270799	0.599286273	Up-regulated	No
Grid1	1.387320345	2.179807865	0.000243895	0.022484676	Up-regulated	Yes
Dscc1	1.087546432	3.096511586	0.000497737	0.036606338	Up-regulated	No
Baiap2l2	0.874498322	0.165478650	0.034369221	0.684231999	Up-regulated	No
Zfp52	1.351603089	-0.97586843	0.001088020	0.062633064	Up-regulated	No
Plin3	0.983536252	2.207539245	0.007122447	0.233024479	Up-regulated	Yes

Appendix Figure S5. Complement to Fig 5. A) Upset plot showing the number of deregulated genes in *Dot1l*-KO vs CTL primary spermatocytes (SC), secondary spermatocytes (SCII) and round spermatids (RS) obtained using DEseq2 differential gene expression analyses. Top panel: with Fold Change > 1.5 and FDR < 0.05. Bottom panel: with Fold Change>1.5 and p value <0.05 (FDR was calculated *a posteriori* on the selected gene lists and found to be <0.05). **B)** Bar graph showing the percentage of deregulated genes in *Dot1l*-KO RS found with the two types of differential analyses and which are enriched in H3K79me2 at their gene body in WT RS. **C)** List of *Slc* genes found deregulated in *Dot1l*-KO round spermatids. **D)** Top: results from HOMER motif analysis on the promoter (2kb) of upregulated genes (p-value < 0.05) in KO RS (top 10). BCL6/BCL6B binding DNA motif is the most significant of the identified motifs (and the only one which does not appear as “possible false positive”). Bottom: list of genes identified with HOMER which have BCL6 DNA binding site in their promoter.

Appendix Table S1

**Table S1**		
**genotyping primers**	**sequence**	**PCR condition**
**Dot1l-F**	**GCAAGCCTACAGCCTTCATC**	**Annealing temperature = 58°C**
**Dot1l-R1**	**CACCGGATAGTCTCAATAATCTCA**	
**Dot1l-R2**	**GAACCACAGGATGCTTCAG**	
**STRA8CRE_oIMR9266F**	**AGATGCCAGGACATCAGGAACCTG**	**Annealing temperature = 62°C**
**STRA8CRE_oIMR9267R**	**ATCAGCCACACCAGACACAGAGATC**	
**Ymtxfp10_ internal control**	**TCACACAGATAAGAGGGTATTG**	
**Ymtxrp13_ internal control**	**GTTTTCCTATCAGGCCATCCA**	

Appendix Table S2

Table S2			
**Western Blot - Primary antibodies**			
**Target**	**Manufacturer**	**Reference**	**Usage**
**DOT1L**	**CST**	**D4O2T**	**1/500 - 1/1000**
**Tubulin**	**Upstate**	**05-661**	**1/3000**
**H3K79me2**	**Abcam**	**ab3594**	**1/500**
**TH2B**	**Upstate**	**07-680**	**1/5000**
**H3**	**Abcam**	**ab 1791**	**1/3000 - 1/10000**
**H4K16ac**	**Upstate**	**07-329**	**1/750**
**Poly ac-H4**	**Millipore**	**06-866**	**1/250**
**TNP2**	**Santa Cruz Biotechnology**	**sc21106**	**1/250**
**PRM2**	**Briar Patch Biosciences**	**MAb-Hup2B**	**1/500**
**Western Blot - Primary antibodies**			
**Target**	**Manufacturer**	**Reference**	**Usage**
**Donkey anti-goat HRP**	**Santa Cruz Biotechnology**	**sc2020**	**1/5000**
**Goat anti-mouse HRP**	**ThermoFisher**	**31430**	**1/5000**
**Goat anti-rabbit HRP**	**ThermoFisher**	**31460**	**1/5000**
**Immunofluorescence-Immunohistochemistry**			
**Target**	**Brand**	**Reference**	**Usage**
**DOT1L**	**CST**	**D402T**	**1/100**
**DOT1L**	**Abcam**	**64077**	**1/100**
**H3K79me2**	**Diagenode**	**C15410051**	**1/100**
**GFP**	**ThermoFisher**	**CAB4211**	**1/100**
**Poly ac-H4**	**Millipore**	**06-866**	**1/200**
**TNP2**	**Santa Cruz Biotechnology**	**sc21106**	**1/50**
**Chromatin Immunoprecipitation**			
**Target**	**Brand**	**Reference**	
**H3K79me2**	**Diagenode**	**C15410051**	

References

Darde TA, Lecluze E, Lardenois A, Stevant I, Alary N, Tuttelmann F, Collin O, Nef S, Jegou B, Rolland AD et al (2019) The ReproGenomics Viewer: a multi-omics and cross-species resource compatible with single-cell studies for the reproductive science community. Bioinformatics 35: 3133-3139

Darde TA, Sallou O, Becker E, Evrard B, Monjeaud C, Le Bras Y, Jegou B, Collin O, Rolland AD, Chalmel F (2015) The ReproGenomics Viewer: an integrative cross-species toolbox for the reproductive science community. Nucleic Acids Res 43: W109-116

Green CD, Ma Q, Manske GL, Shami AN, Zheng X, Marini S, Moritz L, Sultan C, Gurczynski SJ, Moore BB et al (2018) A Comprehensive Roadmap of Murine Spermatogenesis Defined by Single-Cell RNA-Seq. Developmental cell 46: 651-667 e610

Green CD, Ma Q, Manske GL, Shami AN, Zheng X, Marini S, Moritz L, Sultan C, Gurczynski SJ, Moore BB et al (2018) Gene Expression Omnibus GSE112393 (https://www.ncbi.nlm.nih.gov/geo/query/acc.cgi?acc=GSE112393) [DATASET].

Mouse ENCODE transcriptome data (2011) NCBI Sequence Read Archive PRJNA66167 (https://www.ncbi.nlm.nih.gov/bioproject/?term=PRJNA66167) [DATASET].

Muzumdar MD, Tasic B, Miyamichi K, Li L, Luo L (2007) A global double-fluorescent Cre reporter mouse. Genesis 45: 593-605

Russell LD, Hikim APS, Ettlin RA, Clegg ED (1990) Histological and Histopathological Evaluation of the Testis. Cache River Press
