# Supplementary material for: DOT1L regulates chromatin reorganization and gene expression during sperm differentiation
Source: EMBO Rep. 2023 Apr 26;24(6):e56316. doi: 10.15252/embr.202256316 (PMC10240200; doi:10.15252/embr.202256316)
Supplement: Supplementary file 2 — Expanded View Figures PDF [file EMBR-24-e56316-s007.pdf]

Expanded View Figures

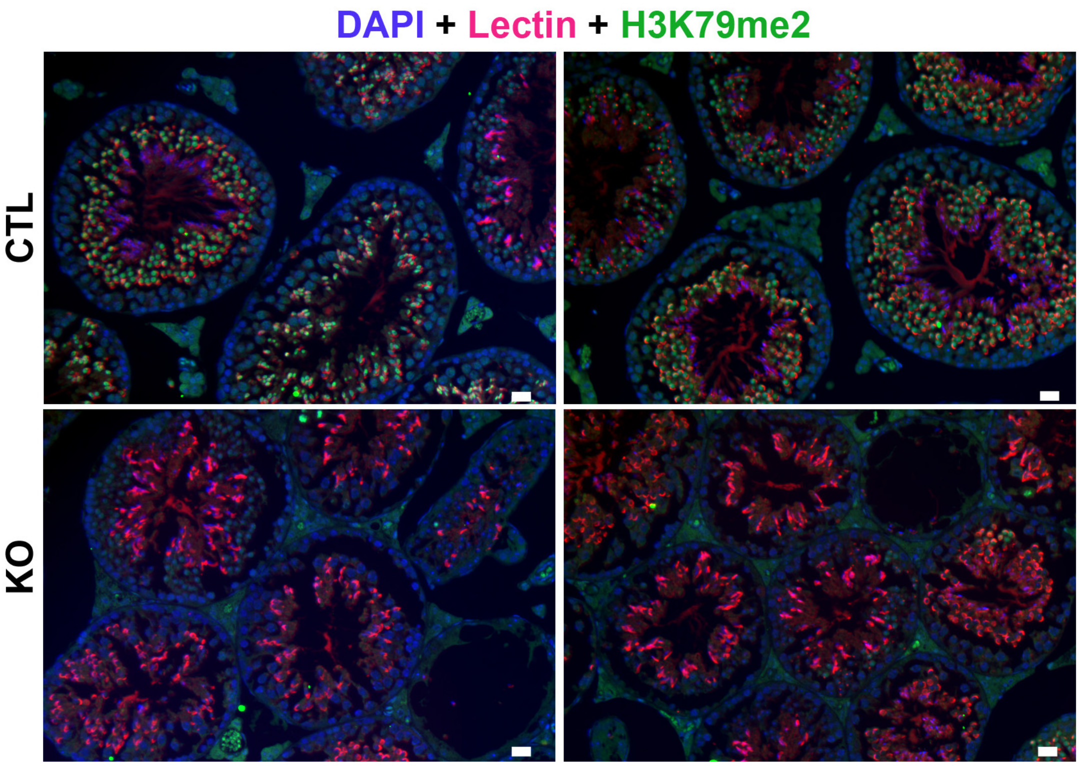

Figure EV1. Immunofluorescence detection of H3K79me2 on testicular sections from adult CTL or *Dot1l*-KO mice.

H3K79me2 signal is shown in green. DAPI (blue) was used to stain nuclei. Lectin (pink) was used to stain acrosome. Scale bar indicates 20 μm.

Figure EV2. Detailed analyses of CTL and *Dot1l*-KO spermatozoa.

A Scatter plots showing the quantification of sperm abnormalities (mean ± SEM; N = 4–5 and four biological replicates for CTL and KO, respectively). Stars indicate significant differences obtained using t-tests after angular transformation of the percentages (**P*-value < 0.05, ***P*-value < 0.005, and ****P*-value < 0.0005).

B Motility parameters from *Dot1l*-KO and CTL spermatozoa following CASA (computer-assisted sperm analyses) (mean ± SEM; N = 12 and 10 biological replicates for CTL and KO, respectively). Specifically, *Dot1l*-KO spermatozoa are slower and more static. They have a more linear movement (LIN) and a decreased straightness (STR), decreased lateral displacements of their heads (ALH) with a lower curvilinear velocity (VCL) resulting in a straight-line velocity (VSL) similar to CTL spermatozoa but a higher Average Path Velocity (VAP). Stars indicate significant differences obtained using Mann–Whitney *t*-tests after angular transformation of the percentages (***P*-value < 0.01, ****P*-value < 0.001, *****P*-value < 0.0001, ns' indicates non-significance). The Beat Cross Frequency (BCF) of CTL and *Dot1l* -KO spermatozoa is similar.

© 2023 The Authors

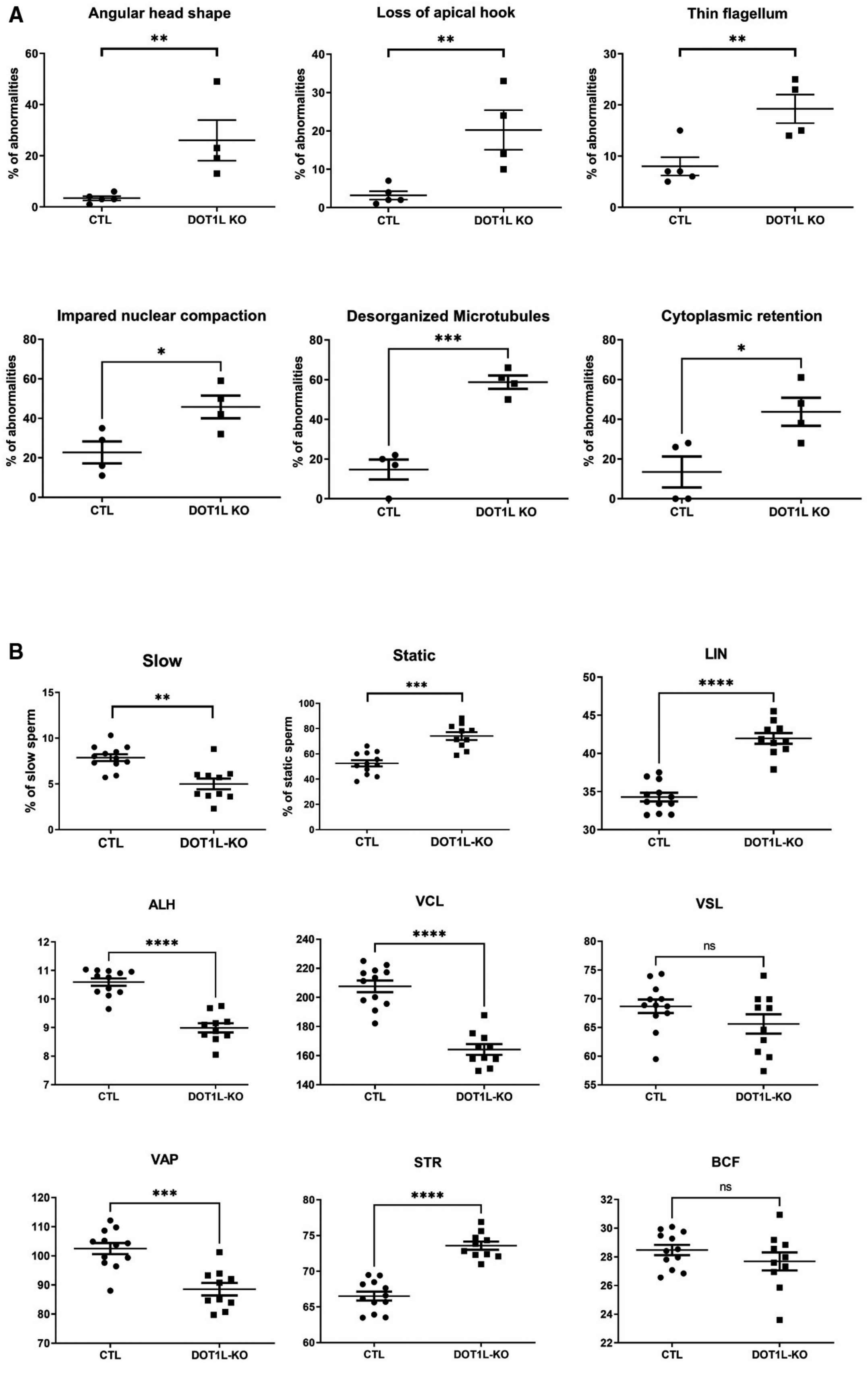

Figure EV2.

 © 2023 The Authors

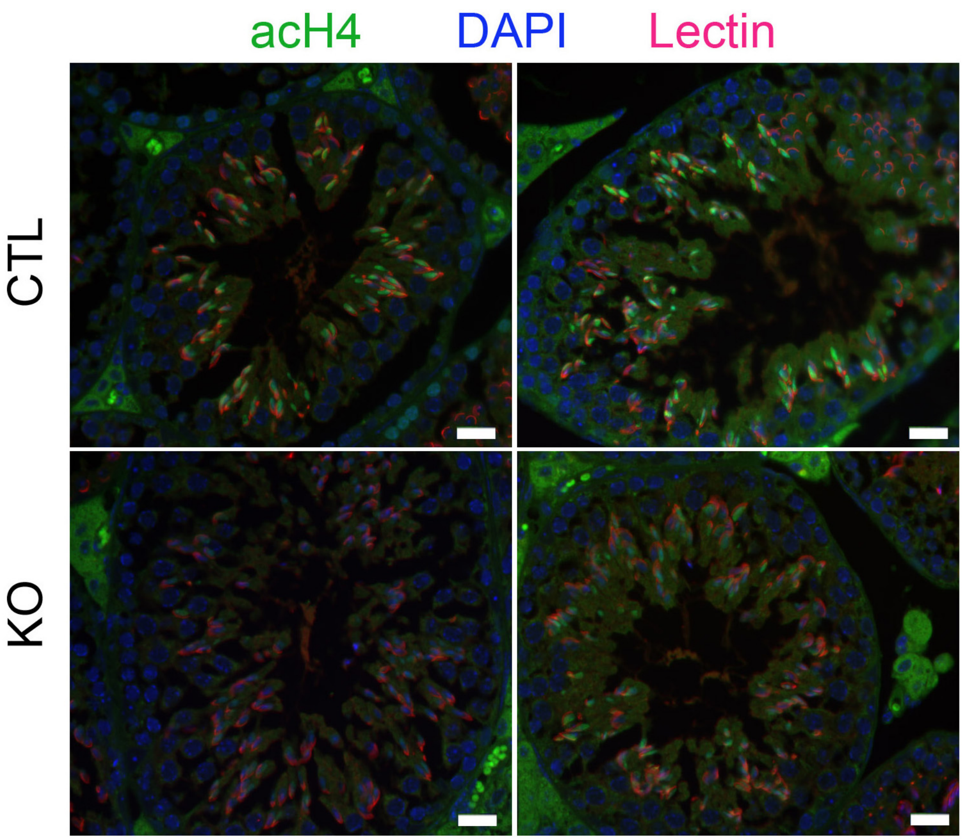

Figure EV3. Confirmation of a decrease in H4 acetylation in elongating spermatids (ES) by immunofluorescence.

Representative pictures of immunofluorescence detection of acH4 (anti-poly-H4ac, green) on stage XI testicular sections. At this stage, acH4 level is strong in CTL elongating spermatids but appears weaker in *Dot1l*-KO. DAPI (blue) was used to stain nuclei and lectin (red) to stage the acrosome and facilitates tubule staging. Scale bar indicates 20 μm.

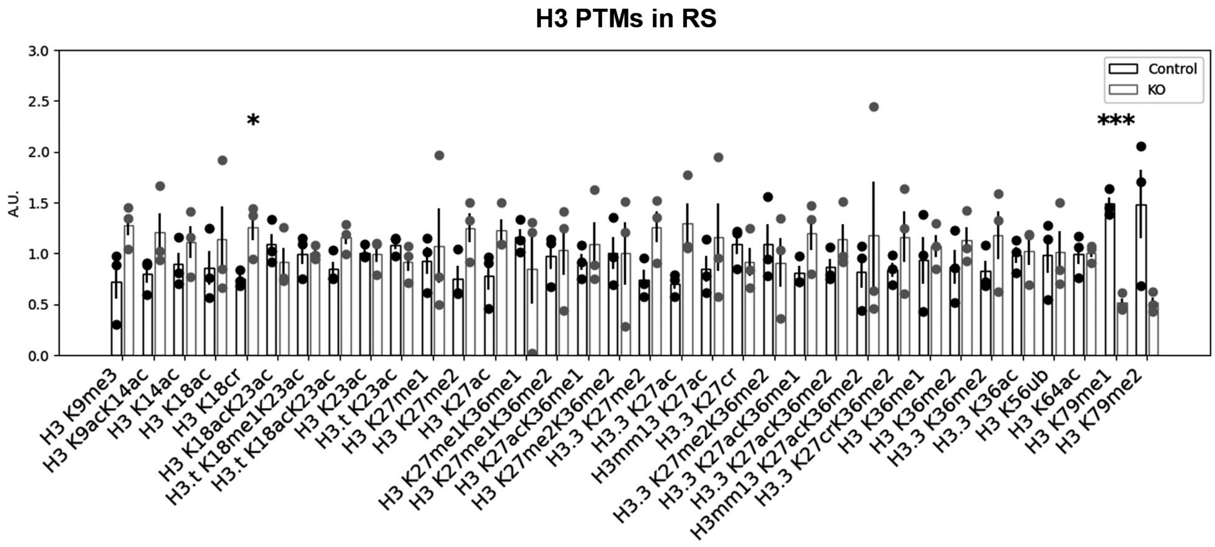

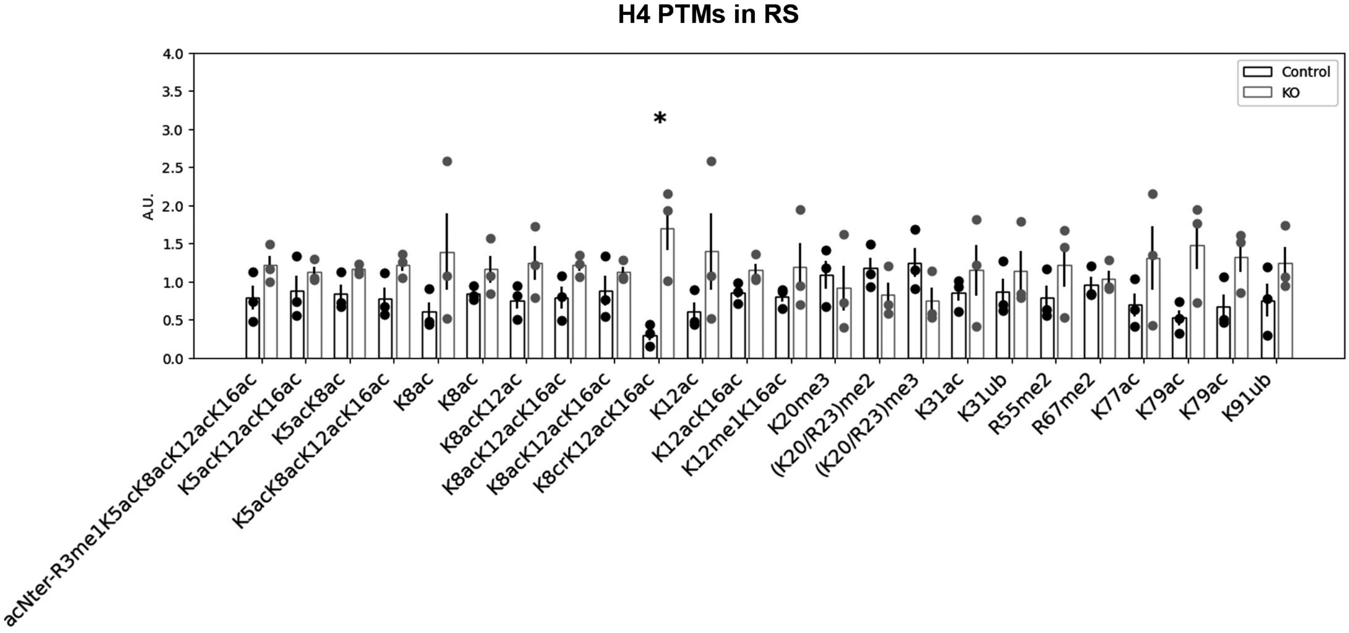

Figure EV4. Quantification of histone H3 and H4 PTMs in *Dot1l*-KO and CTL round spermatids.

LC–MS/MS data acquired on H3 and H4 were processed as described in Fig 4. Quantitative data obtained on biological replicates ($N = 3$ for CTL and 3 for KO) were plotted as dots, and the height of the bars indicates the average value (mean ± SD). A.U. = arbitrary units. *P-value < 0.05, **P-value < 0.005, and ***P-value < 0.0005, obtained with t-tests.

 © 2023 The Authors

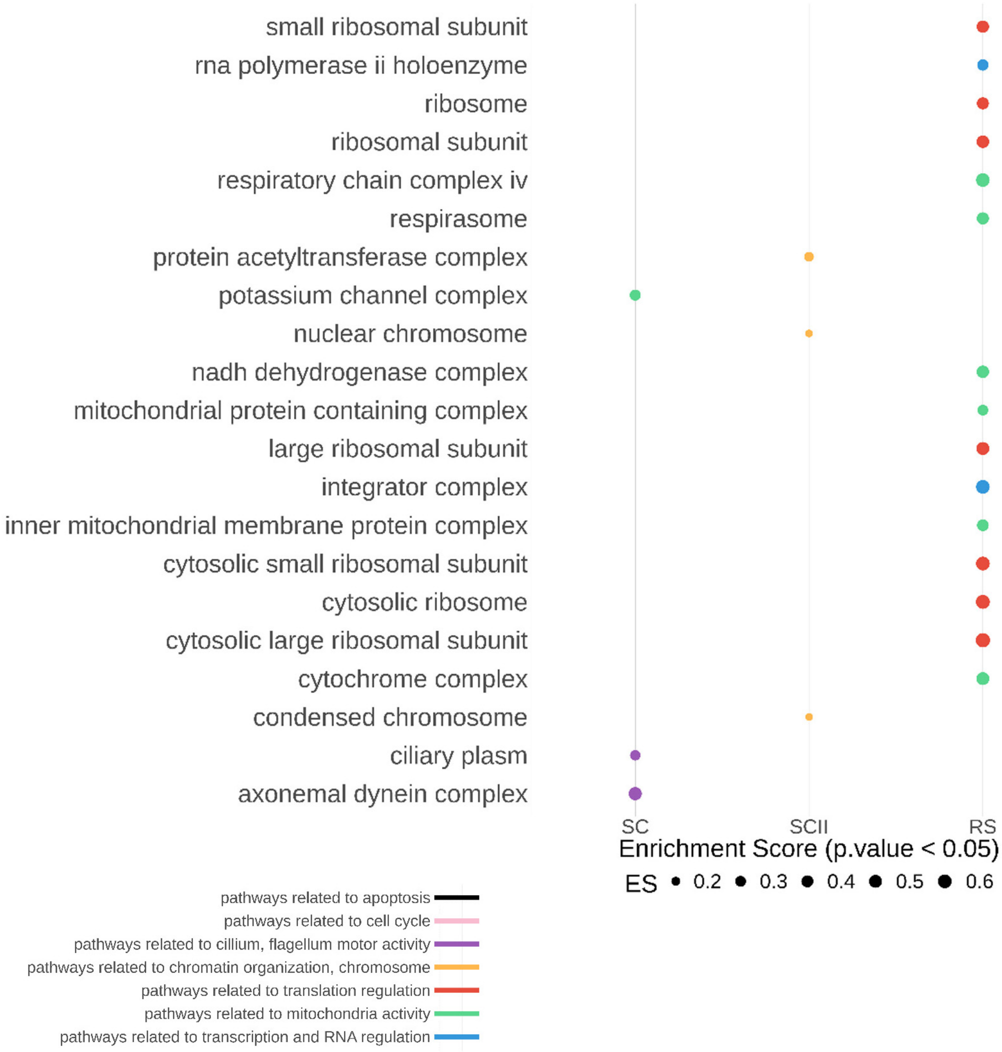

Figure EV5. GSEA analysis of RNA-seq data from *Dot1l*-KO versus CTL SC, SCII, and RS.

The figure shows all the "cellular components" found significantly downregulated in *Dot1l*-KO primary spermatocytes (SC), secondary spermatocytes (SCII) and round spermatids ($P < 0.05$), ranked by their enrichment score (ES).

© 2023 The Authors
