# Supplementary material for: DOT1L regulates chromatin reorganization and gene expression during sperm differentiation
Source: EMBO Rep. 2023 Apr 26;24(6):e56316. doi: 10.15252/embr.202256316 (PMC10240200; doi:10.15252/embr.202256316)
Supplement: Supplementary file 3 — PDF+ [file EMBR-24-e56316-s008.pdf]

Article

SOURCE DATA | TRANSPARENT PROCESS | OPEN ACCESS

EMBO reports

DOT1L regulates chromatin reorganization and gene expression during sperm differentiation

Mélina Blanco[1], Laila El Khattabi[1,2,†], Clara Gobé[1,†], Marion Crespo[3,4], Manon Coulée[1], Alberto de la Iglesia[1,5], Côme Ialy-Radio[1], Clementine Lapoujade[6], Maëlle Givelet[6], Marion Delessard[1], Ivan Seller-Corona[1], Kosuke Yamaguchi[7], Nadège Vernet[8], Fred Van Leeuwen[9], Alban Lermine[10], Yuki Okada[7], Romain Daveau[10], Rafael Oliva[5,11], Pierre Fouchet[6], Ahmed Ziyyat[1,12], Delphine Pflieger[3] & Julie Cocquet[1,*]

Abstract

Spermatozoa have a unique genome organization. Their chromatin is almost completely devoid of histones and is formed instead of protamines, which confer a high level of compaction and preserve paternal genome integrity until fertilization. Histone-to-protamine transition takes place in spermatids and is indispensable for the production of functional sperm. Here, we show that the H3K79-methyltransferase DOT1L controls spermatid chromatin remodeling and subsequent reorganization and compaction of the spermatozoon genome. Using a mouse model in which *Dot1l* is knocked-out (KO) in postnatal male germ cells, we found that *Dot1l*-KO sperm chromatin is less compact and has an abnormal content, characterized by the presence of transition proteins, immature protamine 2 forms and a higher level of histones. Proteomic and transcriptomic analyses performed on spermatids reveal that *Dot1l*-KO modifies the chromatin prior to histone removal and leads to the deregulation of genes involved in flagellum formation and apoptosis during spermatid differentiation. As a consequence of these chromatin and gene expression defects, *Dot1l*-KO spermatozoa have less compact heads and are less motile, which results in impaired fertility.

Keywords flagellum development; gene regulation; H3K79 methylation; histone-to-protamine transition; spermatogenesis

Subject Categories Chromatin, Transcription, & Genomics; Development; Post-translational Modifications & Proteolysis

DOI 10.15252/embr.202256316 | Received 17 October 2022 | Revised 31 March 2023 | Accepted 13 April 2023 | Published online 26 April 2023

EMBO Reports (2023) 24: e56316

Introduction

During the postmeiotic phase of spermatogenesis, male germ cells known as spermatids undergo profound morphological and functional changes to differentiate in spermatozoa. This process is driven by a rich genetic program characterized by the expression of thousands of genes in round spermatids (Soumillon *et al*, 2013; da Cruz *et al*, 2016; Chen *et al*, 2018; Green *et al*, 2018; Ernst *et al*, 2019). Then, when spermatids elongate, their chromatin is extensively remodeled which ultimately results in the eviction of ~ 85–99% of nuclear histones and their replacement by more basic, smaller proteins, called protamines (for reviews, see Rathke *et al*, 2014; Bao & Bedford, 2016). This unique chromatin reorganization induces a 6–10 times higher level of compaction of the spermatozoon chromatin compared to the canonical nucleosome-based chromatin (Ward & Coffey, 1991). Non-histone packaging of male germ cell genome is conserved throughout evolution and expected to be important to protect paternal DNA from damages and prepare

1 Université Paris Cité, INSERM, CNRS, Institut Cochin, Paris, France
2 Chromosomal Genomics Unit, Medical Genetics Department, Sorbonne Université and APHP, Hôpital Armand Trousseau, Paris, France
3 University Grenoble Alpes, CEA, INSERM, UA13 BGE, CNRS, CEA, FR2048, Grenoble, France
4 ADLIN Science, Pépinière « Genopole Entreprises », Evry, France
5 Molecular Biology of Reproduction and Development Research Group, Department of Biomedical Sciences, Faculty of Medicine and Health Sciences, Institut d'Investigacions Biomèdiques August Pi I Sunyer (IDIBAPS), Fundació Clínic per a la Recerca Biomèdica, Universitat de Barcelona (UB), Barcelona, Spain
6 Université de Paris and Université Paris-Saclay, iRCM/IBFJ CEA, UMR Stabilité Génétique Cellules Souches et Radiations, Laboratoire des Cellules Souches Germinales, Fontenay-aux-Roses, France
7 Institute for Quantitative Biosciences, The University of Tokyo, Tokyo, Japan
8 Institut de Génétique et de Biologie Moléculaire et Cellulaire (IGBMC), Département de Génétique Fonctionnelle et Cancer, CNRS, INSERM, Université de Strasbourg, Illkirch, France
9 Division of Gene Regulation, Netherlands Cancer Institute, Amsterdam, The Netherlands
10 MOABI-APHP Bioinformatics Platform-I&D-DSI, Assistance Publique-Hôpitaux de Paris, Paris, France
11 Biochemistry and Molecular Genetics Service, Clinic Barcelona, Barcelona, Spain
12 Service d'Histologie, d'Embryologie, Biologie de la Reproduction, AP-HP, Hôpital Cochin, Paris, France
*Corresponding author. Tel: +33 1 44 41 23 10; E-mail: julie.cocquet@inserm.fr
[†]These authors contributed equally to this work

© 2023 The Authors. Published under the terms of the CC BY 4.0 license.

its reprograming in the zygote, if fertilization occurs (Rathke *et al*, 2014). A compact genome is also an advantage for the motile spermatozoa as it is compatible with a smaller and more hydrodynamic head (Braun, 2001).

In the past decades, the molecular mechanisms driving histone eviction and subsequent compaction of the sperm genome have been the focus of several studies which have demonstrated the essential role of transition proteins (Zhao *et al*, 2004a,b), of histone variants (Montellier *et al*, 2013; Barral *et al*, 2017), of histone post-translational modifications (PTMs), in particular hyperacetylation (Oliva *et al*, 1987; Goudarzi *et al*, 2016), and of writers and readers of histone acetylation (Shang *et al*, 2007; Gaucher *et al*, 2012; Dong *et al*, 2017; Shiota *et al*, 2018; Luense *et al*, 2019). Interestingly, high levels of histone H3 Lysine 79 di- and tri-methylation (H3K79me2 and me3) have been observed at the same time as histone hyperacetylation, just prior to histone removal (Dottermusch-Heidel *et al*, 2014a,b; Moretti *et al*, 2017; Fig 1A), but their biological significance remains to date unknown. H3K79 methylation is mediated by one enzyme, encoded by the gene *Dot1l*, of which pattern of expression and sequence are conserved from *Drosophila* to mammals. *Dot1l* has been implicated in development, cell reprogramming, differentiation, and proliferation (for review, see Kim *et al*, 2014; Vlaming & van Leeuwen, 2016). It has recently been found to be essential for spermatogonial stem cell self-renewal (Lin *et al*, 2022) but its role in postmeiotic male germ cells, where it is the most highly expressed (Dottermusch-Heidel *et al*, 2014a,b; Moretti *et al*, 2017), has not been addressed.

In the present paper, we investigated *Dot1l* role in postmeiotic cells by a comprehensive analysis of the phenotypic and molecular consequences of its knockout in mouse male germ cells. We found that *Dot1l* is required for gene regulation and chromatin reorganization in spermatids and that its knockout leads to the production of abnormally shaped nonfunctional spermatozoa with a deficient chromatin and nucleus compaction.

Results

Dot1l is essential for spermatogenesis and male fertility

In both mouse and human, *Dot1l/DOT1L* gene is highly expressed in the testis (Appendix Fig S1A). More precisely, it is expressed in all testicular cells with a strong expression at postnatal stage in meiotic cells (spermatocytes) and postmeiotic cells (spermatids; Appendix Fig S1A–C; Dottermusch-Heidel *et al*, 2014a,b; Moretti *et al*, 2017; Green *et al*, 2018). To investigate the role of *Dot1l* in male germ cells, we generated a conditional knockout using *Stra8-Cre* recombinase (Sadate-Ngatchou *et al*, 2008) and a mouse transgenic line in which *Dot1l* exon 2 is flanked by LoxP sites (Jo *et al*, 2011; Appendix Fig S1D). Previous studies have reported that *Stra8-Cre* is specifically expressed in the male germline where it is expressed from postnatal day 3 and CRE recombinase reaches maximum efficiency in pachytene spermatocytes (Sadate-Ngatchou *et al*, 2008; Bao *et al*, 2013). Bao *et al* (2013) have also observed that *Stra8-Cre* is more efficient at cutting one allele rather than two. We, therefore, generated mice in which one allele of *Dot1l* is floxed and one allele is already deleted (Δ) to increase efficiency of floxed exon excision upon *Cre* recombinase expression ($Dot1l^{Fl/\Delta}$; *Stra8-Cre* mice, hereafter called *Dot1l*-KO or KO). First, to estimate the efficiency of *Dot1l* knockout, we detected DOTL1 protein by western blot and immunochemistry on adult testes from *Dot1l*-KO ($Dot1l^{Fl/\Delta}$; *Stra8-Cre*) and controls ($Dot1l^{Fl/Fl}$; Fig 1B and C and Appendix Fig S1E–G). We found a reduction of > 85% of DOT1L signal but not complete abolishment/knockout of DOT1L level, despite strong activity of *Stra8-Cre* in most adult germ cells (Appendix Fig S1H). Analyses of purified germ cell fractions confirmed reduced level but not complete absence of DOTL1 protein, along with a reduction of H3K79me2 level (Figs 1D and EV1 and Appendix Fig S1I). Multiple protein isoforms of DOT1L have been described in the literature and several of them were observed in our western blots, including the canonical (~ 165 kDa) and testis-specific (~ 185 kDa) isoforms (Zhang *et al*, 2004; Dottermusch-Heidel *et al*, 2014b), as well as less studied isoforms such as Q679P5 (~ 122KDa) or Q6XZL7 (~ 68KDa; https://www.uniprot.org). All of them were markedly reduced in *Dot1l*-KO testicular extracts (Appendix Fig S1E and I).

To investigate the impact of *Dot1l* knockout on spermatogenesis and male fertility, the reproductive parameters of adult *Dot1l*-KO males were investigated: significant decreases in testis weight and sperm count were observed (independently of body weight, Fig 2A and Appendix Fig S2A and B). Histological analyses revealed the presence of “empty” tubules without germ cells or missing germ cell layers (Fig 2B and Appendix Fig S2C) highlighting a spermatogonial defect as described by Lin *et al* (2022). However, there was no homogenous blockage/arrest at a specific stage of *Dot1l*-KO spermatogenesis. These observations were confirmed by cytometry analyses in which the number of all germ cell populations (i.e., spermatogonia with Side Population phenotype, spermatocytes, and spermatids) was found decreased in *Dot1l*-KO testes (Fig 2C), but their frequency was overall not altered (Appendix Fig S2F). Collectively, these data show that the *Dot1l*-KO we produced does not lead to a spermatogenesis arrest. When mated to WT females, *Dot1l*-KO males were found to be hypofertile with significant reductions in the number of litters and in litter size compared to that of control (CTL) males (Fig 2D and Appendix Fig S2D and E). We genotyped the few pups obtained and observed that the floxed allele was transmitted to 35% of babies (12 out of 34) and the deleted allele to 65% of them. This indicates that the deleted allele can be transmitted but less efficiently than what was expected based on our quantification of *Dot1l*-KO spermatids by immunofluorescence (i.e., only 65% of transmission of the deleted allele although > 85% of spermatids were devoid of DOT1L protein).

The reduction in litter number and size could result from impaired fertilizing ability of *Dot1l*-KO sperm or from a developmental defect. To address this question, we measured the fertilization rate 24 h after WT females were mated with *Dot1l*-KO or CTL males. The numbers of fertilized and non-fertilized oocytes collected in the oviduct ampulla were counted in both groups (a total of 113 oocytes were analyzed from 9 WT females mated with 4 CTL, and 73 oocytes from 6 WT females mated with 3 KO). A significant reduction in the fertilization rate was observed for *Dot1l*-KO indicating that *Dot1l*-KO sperm are less functional than CTL sperm (Fig 2E). In addition to *in vivo* fertility monitoring, we performed *in vitro* fertilization (IVF) assays using the same number of spermatozoa per experiment. Using spermatozoa from *Dot1l*-KO males, no oocyte was fertilized *in vitro*, while spermatozoa from control males

 © 2023 The Authors

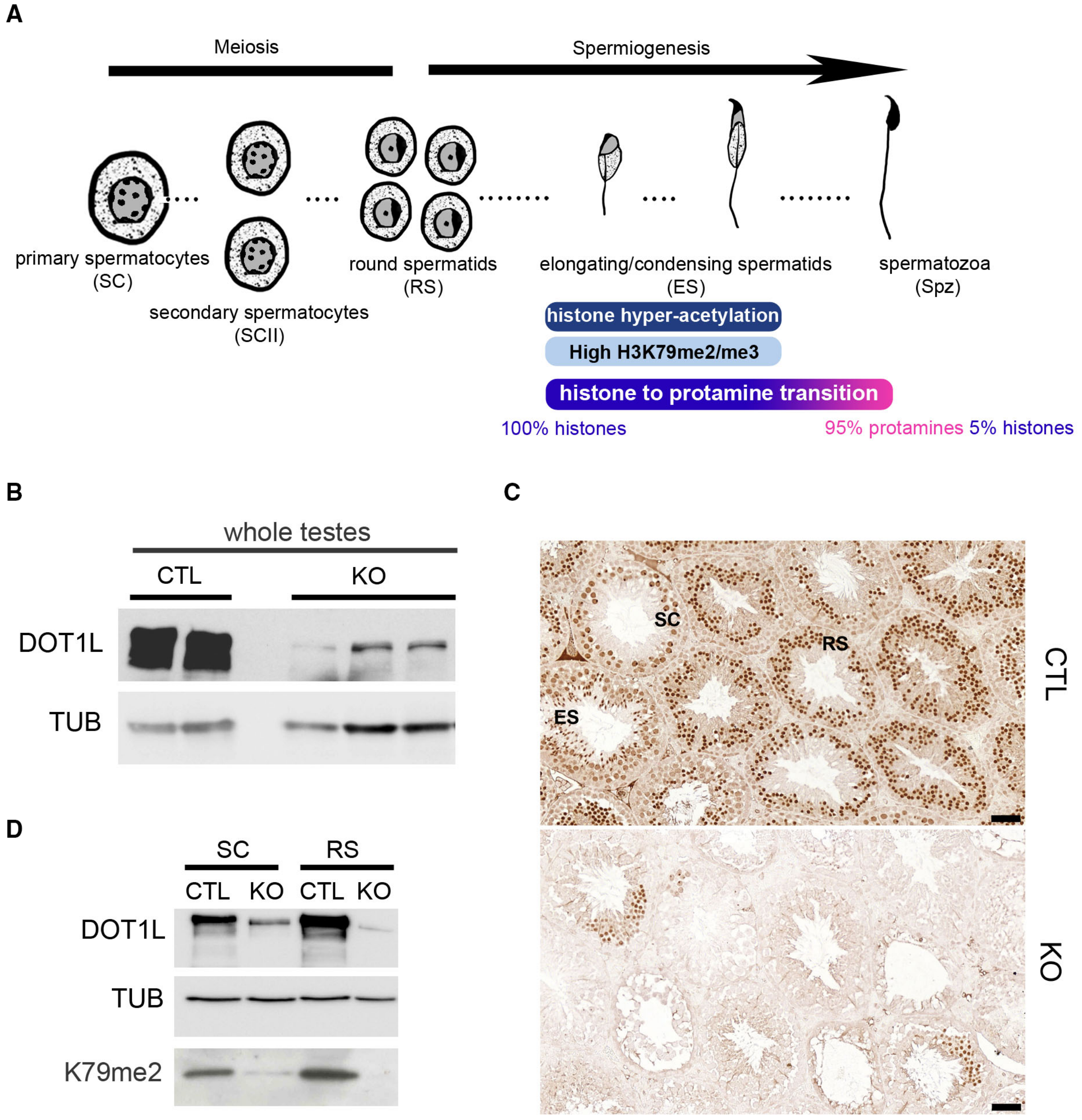

Figure 1. DOT1L expression in meiotic and postmeiotic germ cells of *Dot1l*-KO and CTL male mice.

A Spermatogenesis scheme representing the dynamic of histone to protamine transition.

B Western blot detection of DOT1L and TUBULIN (TUB) in whole testicular protein extracts from CTL and *Dot1l*-KO (KO) adult mice.

C Immunohistochemistry detection of DOT1L in testicular sections from CTL and *Dot1l*-KO (KO) adult mice. ES, elongating spermatids; RS, round spermatids; SC, spermatocytes. Pictures were taken using the same parameters. Scale bars indicate 50 µm.

D Western blot detection of DOT1L and H3K79me2 in *Dot1l*-KO (KO) and CTL germ cells. Normalization was performed by detecting the membrane with anti-TUBULIN (TUB). SC = primary spermatocytes, RS = round spermatids.

Data information: See also Fig EV1 and Appendix Fig S1.
Source data are available online for this figure.

(including WT, CTL and HET males) led to the fertilization of ~ 37% of oocytes (Fig 2E, and see Appendix Fig S2G for individual values). When using oocytes devoid of zona pellucida, in order to bypass the zona pellucida crossing step, spermatozoa from *Dot1l*-KO males still performed very poorly compared to that of CTL males (~ 3% of fertilized oocytes using *Dot1l*-KO sperm compared to ~ 88% for CTL, Fig 2E), confirming that *Dot1l*-KO dramatically reduces sperm fertilizing ability. IVF is known to be less efficient and to lead to lower fertilization rates than *in vivo*. It is, therefore, more sensitive to sperm defects, as observed in other studies (Fujihara *et al*, 2010; Kawano *et al*, 2010; Barraud-Lange *et al*, 2020; Gadadhar *et al*, 2021).

© 2023 The Authors

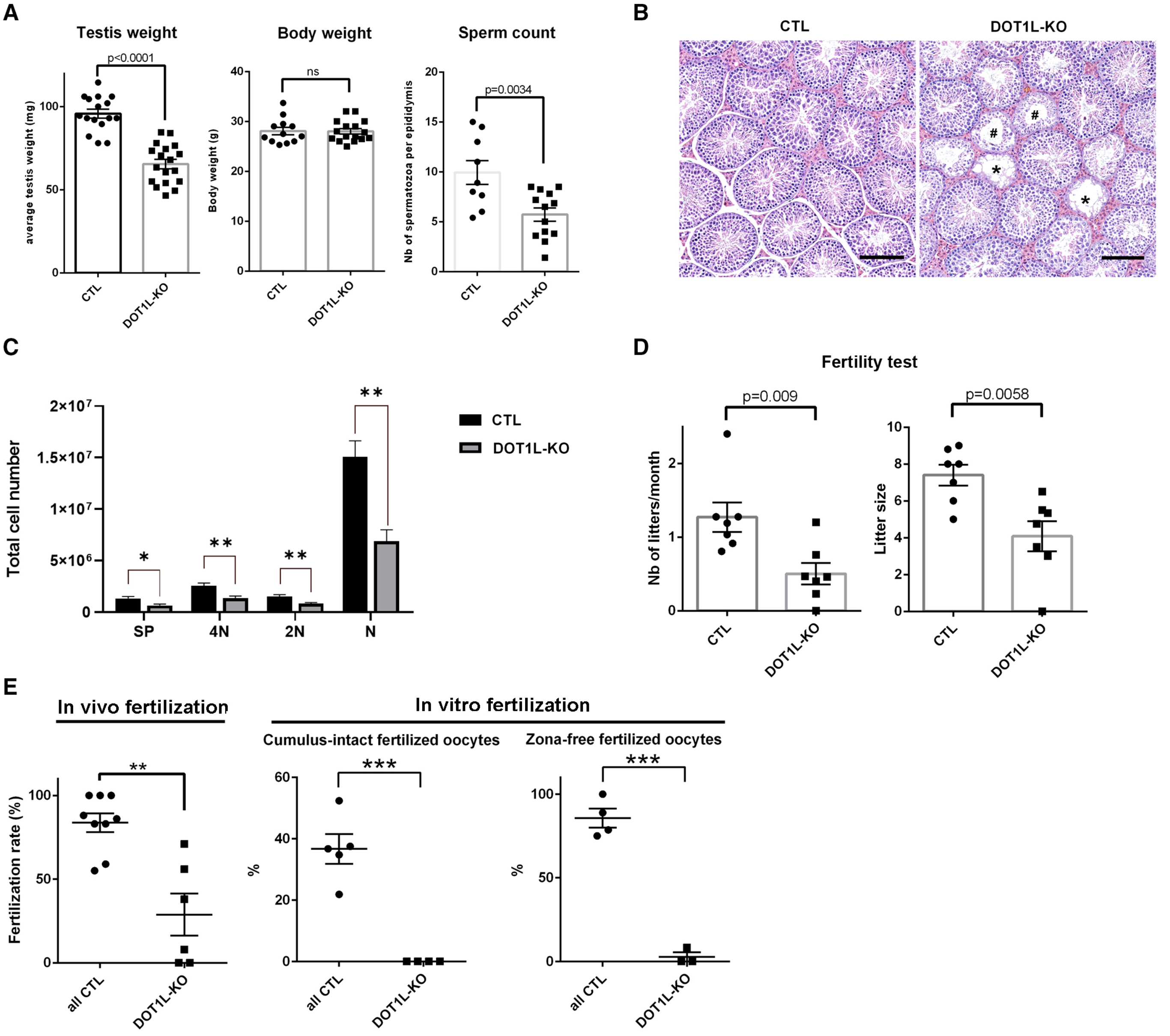

Figure 2. *Dot1l*-KO in male germ cells impairs spermatogenesis and male fertility.

A Scatter plots (mean ± standard error of the mean, SEM) showing the average testis weight, body weight and sperm count in *Dot1l*-KO and CTL adult mice (from ~ 2- to 4-month-old males, $N > 10$ biological replicates for CTL and *Dot1l*-KO).

B Histology of *Dot1l*-KO and CTL testes (periodic acid-Schiff staining). Scale bars indicate 200 μm.

C Schematic diagram representing total number of each cell type per testis in CTL and *Dot1l*-KO males, as calculated by FACS (mean ± SEM). 4N = Primary spermatocytes, 2N = Secondary spermatocytes, N = spermatids, SP = Side Population representing premeiotic germ cells (spermatogonia). Testes from 4 CTL and 3 *Dot1l*-KO males were analyzed (biological replicates). *P-value < 0.05 or **P-value < 0.01 (Student t-test).

D Results from the tests of fertility (natural mating) of *Dot1l*-KO and CTL males (seven males in each group mated for ~ 3 months with wild-type females). Schematic diagrams representing the number of litters and the litter size from progeny of *Dot1l*-KO or CTL males (mean ± SEM).The P-value obtained with a Student t-test is indicated on each graph.

E *In vivo* (left panel) and *in vitro* fertilization results. The percentages of fertilized oocytes are indicated for WT, CTL, and HET males ('all CTL') and for *Dot1l*-KO males. The left panel shows the mean (± SEM) fertilization rate of oocytes collected in superovulated females 24 h after natural mating. Nine and six females with a positive vaginal plug were analyzed after mating with CTL and *Dot1l*-KO males, respectively. This represents 113 and 73 oocytes analyzed after mating with CTL or KO males, respectively. The central and right panels show *in vitro* fertilization results (mean fertilization rate ± SEM) using oocytes with intact cumulus, or using oocytes of which the zona pellucida was removed beforehand. **P-value < 0.005 or ***P-value < 0.0005 (unpaired t-test performed after angular transformation of percentages).

Data information: See also Appendix Fig S2.
Source data are available online for this figure.

 © 2023 The Authors

Dot1l-KO disrupts spermiogenesis and leads to the production of malformed nonfunctional spermatozoa

To determine the reason why *Dot1l*-KO spermatozoa are less functional than CTL ones, we investigated their morphology and motility. Spermatozoa are highly specialized cells characterized by a small head which encompasses their very compact nucleus, a long flagellum that confers motility and almost no cytoplasm. First, we observed that *Dot1l*-KO epididymal spermatozoa displayed malformed heads and abnormal flagella (characterized by thinning of some sections in their midpiece; Figs 3A and EV2A). Inside the flagellum, the axoneme normally contains a ring of nine outer microtubule doublets and two central microtubules (9 + 2 axoneme). In *Dot1l*-KO sperm, ultrastructure analyses revealed abnormal axonemes, characterized by disorganized microtubules (< 9 + 2 microtubules; Fig 3B). Nuclear compaction was also found impaired, observed as less contrasted nuclei in KO in comparison with CTL sperm. *Dot1l*-KO sperm also showed an increased cytoplasmic retention (Figs 3B and EV2A). Using CASA (computer-assisted sperm analyses), we observed that *Dot1l*-KO sperm motility was impaired, with a reduced proportion of motile and progressively motile (i.e., swimming in an overall straight line or in very large circles) spermatozoa (Figs 3C and EV2B). This was not due to cell death as sperm vitality was comparable between CTL and *Dot1l*-KO mice (Appendix Fig S3A). Axoneme/flagellum organization, nucleus compaction, and cytoplasm elimination occur during the differentiation of spermatids in spermatozoa (i.e., spermiogenesis). We measured apoptosis during this transition by TUNEL assay on testicular sections, and found a higher incidence of apoptotic elongating/condensing spermatids in *Dot1l*-KO than in CTL, another indication of a defective spermiogenesis (i.e., 6% of tubules containing apoptotic elongating/condensing spermatids in CTL vs. 40% of tubules in DOT1L-KO, $P = 0.0007$, Fig 3D). Collectively, our data indicate that *Dot1l*-KO spermiogenesis is impaired at multiple levels and produces less functional spermatozoa.

Dot1l-KO modifies the chromatin of postmeiotic male germ cells, which results in abnormal chromatin reorganization and compaction in spermatozoa

During spermiogenesis, spermatid chromatin undergoes specific reorganization and compaction as most histones are removed and replaced with protamines (Rathke *et al*, 2014; Bao & Bedford, 2016). Histone variants (such as TH2B or H2AL2) and transition proteins (TNP) are highly expressed in spermatids during histone-to-protamine transition and are essential for the correct reorganization and compaction of the sperm genome with protamines (Montellier *et al*, 2011; Barral *et al*, 2017). In addition to the nuclear compaction defects observed by electron microscopy (Fig 3B), we noticed that *Dot1l*-KO sperm chromatin is more sensitive to nucleoplasmin-induced decompaction than CTL sperm chromatin. Nucleoplasmin, or NPM, is a histone chaperone known to facilitate paternal chromatin decompaction and reorganization after fertilization owing to its ability to bind histones and sperm nuclear basic proteins (Frehlick *et al*, 2007). Following NPM treatment, a higher proportion of soluble histones was found in *Dot1l*-KO compared to CTL sperm (Appendix Fig S3B) in agreement with a chromatin compaction defect. We next quantified TH2B and H3 histones in epididymal spermatozoa by western blot and, despite inter individual variability, we found significantly more residual histones in *Dot1l*-KO than in CTL sperm chromatin (Fig 3E and H and Appendix Fig S3C and D). We also quantified transition protein 2 (TNP2) and found a clear retention of this protein in *Dot1l*-KO spermatozoa while nothing could be detected in CTL sperm (Fig 3F and H and Appendix

Figure 3. *Dot1l*-KO in male germ cells leads to multiple spermiogenesis defects including flagellar abnormalities and incomplete sperm chromatin reorganization and compaction. ▶

A Representative pictures of *Dot1l*-KO and CTL epididymal spermatozoa. The black arrow indicates an abnormal sperm head (without apical hook) and white arrows indicate thinning of the flagellum. Scale bars represent 10 µm.

B Ultrastructure pictures (electron microscopy) from epididymal spermatozoa showing multiple abnormalities in *Dot1l*-KO compared to CTL: disorganized microtubules (blue arrow), impaired nuclear compaction (top panels) and increased cytoplasmic retention (bottom right panel). Scale bars represent 1 µm.

C Scatter plots (mean values following angular transformation ± SEM) of the percentage of motile and progressively motile spermatozoa in *Dot1l*-KO and CTL adult mice ($N = 12$ biological replicates for CTL and 10 biological replicates for *Dot1l*-KO) obtained following CASA (computer-assisted sperm analysis). ***P-value < 0.0005 (Mann–Whitney test performed after angular transformation of the percentages).

D Scatter plots showing the percentage of testicular tubules with TUNEL-positive elongating/condensing spermatids (ES) (mean per animal ± SEM, $N = 5$ biological replicates for CTL and 6 biological replicates for *Dot1l*-KO). **P-value < 0.005 (unpaired t-test performed after angular transformation of percentages). A representative picture of *Dot1l*-KO testicular sections following TUNEL assay is shown on the left. TUNEL positive elongating/condensing spermatids are visible inside the tubule (in green, white arrowhead). DAPI (blue) was used to stain nuclei. Scale bar indicates 20 µm.

E Western blot detection of histones H3 and TH2B in spermatozoa from *Dot1l*-KO and CTL males. The same quantity of material was loaded in each well (i.e., extracts from 2 million spermatozoa). NS indicates a non-specific band also observed on the membrane stained with Ponceau (see Appendix Fig S3C).

F Western blot detection of transition protein 2 (TNP2) in spermatozoa from *Dot1l*-KO and CTL males. The same quantity of material was loaded in each well (i.e., extracts from 2 million spermatozoa).

G Coomassie-stained protamine extracts from CTL and *Dot1l*-KO spermatozoa following acid urea gel electrophoresis (same gel, two different intensities). The same quantity of material has been loaded in each well (i.e., extracts from 1.4 million spermatozoa). Protamine 1 and 2 bands (PRM1 and PRM2, respectively) are detected at the bottom of the gel. The rectangle highlights the bands that are likely immature forms of Protamine 2 (i.e., non-cleaved precursor forms). The right panel shows a western blot detection using anti-PRM2 antibody which confirms that one of the high molecular weight band only observed in *Dot1l*-KO spermatozoa is an immature form of Protamine 2 (Pre-PRM2). The corresponding Coomassie-stained gel is also shown.

H Quantification of histones TH2B and H3 ($N = 11$ and 10 biological replicates for CTL and KO, respectively), of TNP2 ($N = 8$ biological replicates for CTL and for KO) and of PRM1/PRM2 ratio ($N = 3$ biological replicates for CTL and for KO) in *Dot1l*-KO and CTL spermatozoa, related to Fig 3E–G. The data shown are normalized to control mean values (± SEM). *P-value < 0.05, ***P-value < 0.0005 (unpaired t-tests). A.U. = arbitrary units.

Data information: See also Fig EV2 and Appendix Fig S3.
Source data are available online for this figure.

© 2023 The Authors

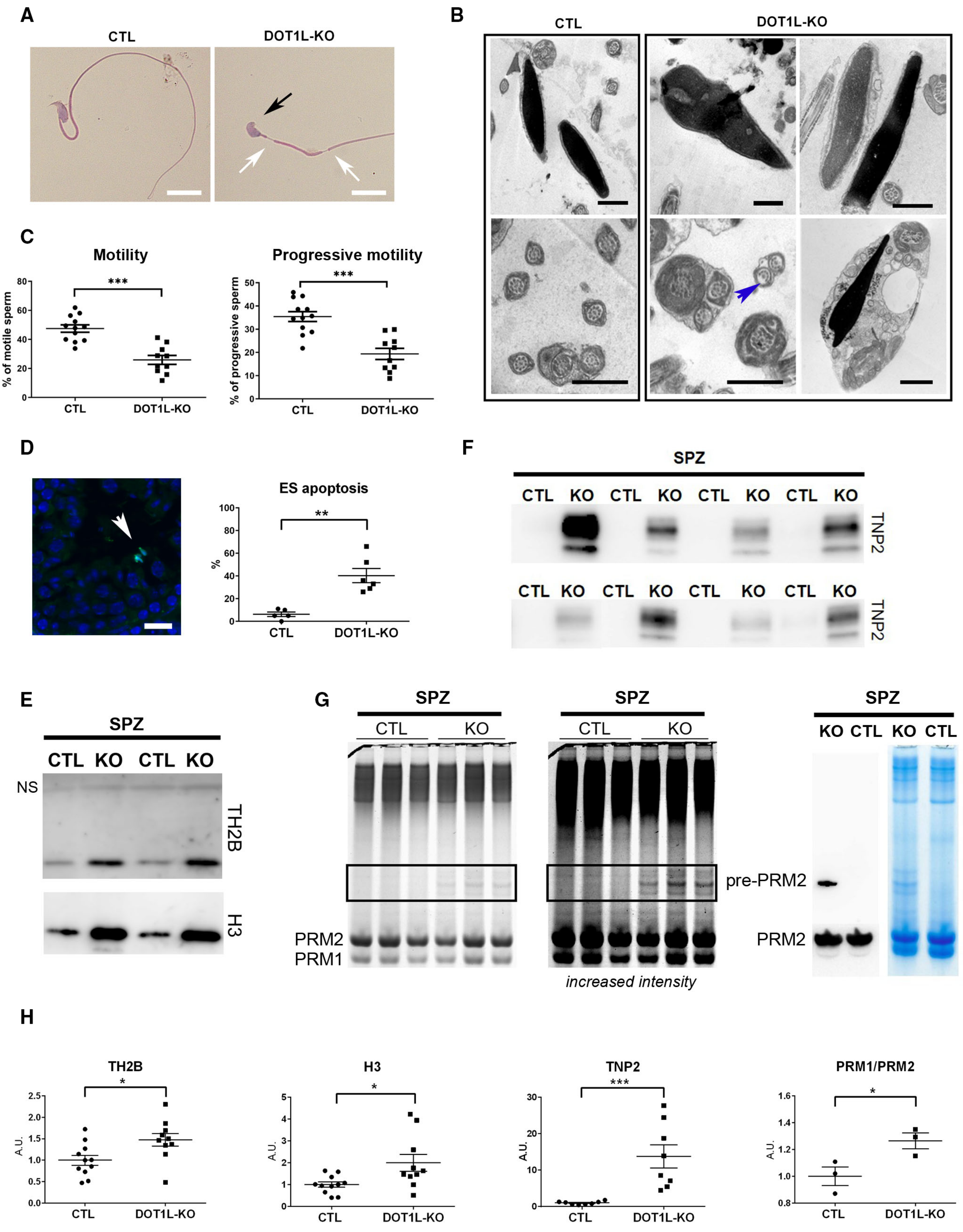

Figure 3.

 © 2023 The Authors

Fig S3E). Finally, we quantified protamines on acid-urea gels and observed an accumulation of immature (non-cleaved) forms of Protamine 2 in *Dot1l*-KO spermatozoa versus. CTL (Fig 3G). A slight but significant distorted Protamine1/Protamine2 (PRM1/PRM2) ratio was also observed in *Dot1l*-KO spermatozoa (Fig 3G and H and Appendix Fig S3F).

The transition from nucleosomes to protamine-based chromatin starts in elongating spermatids with the incorporation of histone post-translational modifications (PTMs) and histone variants (for reviews see Oliva, 2006; Rathke *et al*, 2014; Bao & Bedford, 2016). To better understand how DOT1L impacts on chromatin organization prior to histone removal, we quantified histone PTMs and variants by liquid chromatography–tandem mass spectrometry (LC–MS/MS) in whole testes and in elongating/condensing spermatids. In whole testis histone extracts, we observed, as expected, a clear decrease of H3K79 mono- and di-methylation in *Dot1l*-KO samples compared to CTL (Appendix Fig S4A). The tri-methylation of H3K79 was not detectable with this analysis, possibly due to its small stoichiometry. No other significant differences in histone H3 PTMs were detected between *Dot1l*-KO and CTL whole testicular histone extracts. However, a significant decrease in histone H4 hyperacetylated form (i.e., H4K5ac, K8ac, K12ac, K16ac) was observed (Appendix Fig S4A). In elongating/condensing spermatids (ES), the stage during which chromatin is extensively remodeled, a significant decrease in H3K79 mono- and di-methylation levels was also observed (Fig 4A). Here also, H3K79 tri-methylation was not detectable. Several other quantitative changes in histone PTMs were not only observed, such as modest increases in H3K23ac, H2AR88me1, and TH2BK35ac, but also a dramatic decrease of H4K20 or R23 mono-, di-, and tri-methylated forms (Fig 4 and Appendix Fig S4B). Quantification of histone variants showed significant changes in H1 variants and in canonical H2A (Appendix Fig S4C). The most remarkable change was, as in whole testes, a severe decrease in H4 acetylation in *Dot1l*-KO elongating/condensing spermatids, in particular, hyperacetylated forms such as H4K5ac, K12ac, K16ac, H4K8ac, K12ac, K16ac, and H4K5ac, K8ac, K12ac, K16ac (Fig 4B). In elongating spermatids, histone H4 hyperacetylation is an essential step of histone-to-protamine transition, as it facilitates chromatin loosening and nucleosome disassembly prior to histone removal (for review see Oliva, 2006). By western blot and immmunofluorescence, we also observed a decrease in H4 acetylation in *Dot1l*-KO elongating/condensing spermatids compared to CTL using anti-H4K16ac or anti-poly H4ac antibody (Fig EV3 and Appendix Fig S4D).

We next quantified histone PTMs and variants at an earlier stage of spermiogenesis, in round spermatids (RS), and did not see any decrease in H4 acetylation or H4K20 methylation in KO versus. CTL RS (Fig EV4) in contrast with what was observed in ES (Fig 4). Apart from the expected decrease in H3K79 methylation, the only quantitative changes that were detected in RS were increases in H3K18 crotonylation (H3K18cr), in H4K8cr, K12ac, K16ac and in H1 variants (Fig EV4 and Appendix Fig S4C and E). In spermatozoa, some H3K79 methylation has been detected on persistent histones (Luense *et al*, 2016; Moretti *et al*, 2017) and a significant decrease in H3K79me1 and H3K79me2 levels was visible in *Dot1l*-KO spermatozoa using LC–MS/MS. Apart from a modest increase in H3K18ac, no other changes were detected at this stage (Appendix Fig S4F).

All these observations indicate that *Dot1l* KO has a profound impact on postmeiotic chromatin in ES at the time of its reorganization, which disrupts histone-to-protamine transition and leads to a higher proportion of retained histones, transition proteins, and unprocessed protamine 2 in spermatozoa.

DOT1L regulates the expression of genes involved in essential processes of spermatid differentiation

In order to gain insight into the gene expression changes associated with *Dot1l* deficiency in male germ cells, we performed RNA-Seq analyses on purified germ cells from three different spermatogenic stages, each in five replicates: primary spermatocytes (SC), secondary spermatocytes (SCII), and round spermatids (RS; Fig 5A). We did not include elongating/condensing spermatids in this analysis because their transcriptome is very similar to that of round spermatids, and, at this stage, transcription progressively shuts down as a consequence of histone-to-protamine transition and genome compaction. RNA-Seq differential analyses showed hundreds of significantly deregulated genes in *Dot1l*-KO versus CTL samples (DEseq2, with a Fold change > 1.5 and FDR < 0.05). In parallel, we performed a less stringent analysis (DEseq2, with a Fold change > 1.5 and $P < 0.05$, then *a posteriori* validation of FDR) and found between ~ 600 and 1,000 deregulated genes at each stage (Fig 5B and Appendix Fig S5A). With both analyses, the number of deregulated genes increased with progression of male germ cell differentiation and was therefore highest in RS (Fig 5B and Appendix Fig S5A), and approximately half of deregulated genes were found in common in at least two stages (Appendix Fig S5A). In the three cell types, more genes were found upregulated than downregulated in KO samples (Fig 5B) in agreement with results obtained recently in *Dot1l*-KO somatic cells (Aslam *et al*, 2021; Cattaneo *et al*, 2022). To link gene deregulation in *Dot1l*-KO mice with DOT1L methyltransferase activity, we compared deregulated genes with the genomic location of H3K79me2 that we obtained by ChIP-seq in RS. H3K79me2 is the best characterized H3K79 methylation mark and is known to mark the body of transcriptionally active genes. H3K79me1 and me3 have similar trends, though broader for the former, and more restricted for the latter (Vlaming & van Leeuwen, 2016). Here, in wild type RS, H3K79me2 was found enriched at the body (with the strongest signal found near the Transcriptional Start Site) of genes which were downregulated in the KO but not those found upregulated (Fig 5C and Appendix Fig S5B). Observation of ChIP peaks confirmed the presence of H3K79me2 mark at down but not upregulated genes (Fig 5D).

We next investigated deregulated pathways using GSEA (Gene Set Enrichment Analysis), a threshold-free method which considers all genes based on their differential expression rank and found a downregulation of biological pathways related to apoptosis, transcription/RNA regulation, chromatin/chromosome organization, and mitochondria activity in *Dot1l*-KO versus CTL round spermatids (Fig 5E). Prior to spermiogenesis, pathways related to cilium or flagellum motor activity were found downregulated in *Dot1l*-KO primary spermatocytes while, in *Dot1l*-KO secondary spermatocytes, pathways related to chromatin/chromosome organization were the most significantly deregulated (Fig 5E). When looking at gene ontology for "cellular components," pathways related to ribosomal structure or to mitochondrial proteins were found deregulated in RS (Fig EV5). Strikingly, several *Slc* genes were found deregulated in *Dot1l*-KO RS (Appendix Fig S5C). *Slc* genes encode solute carriers,

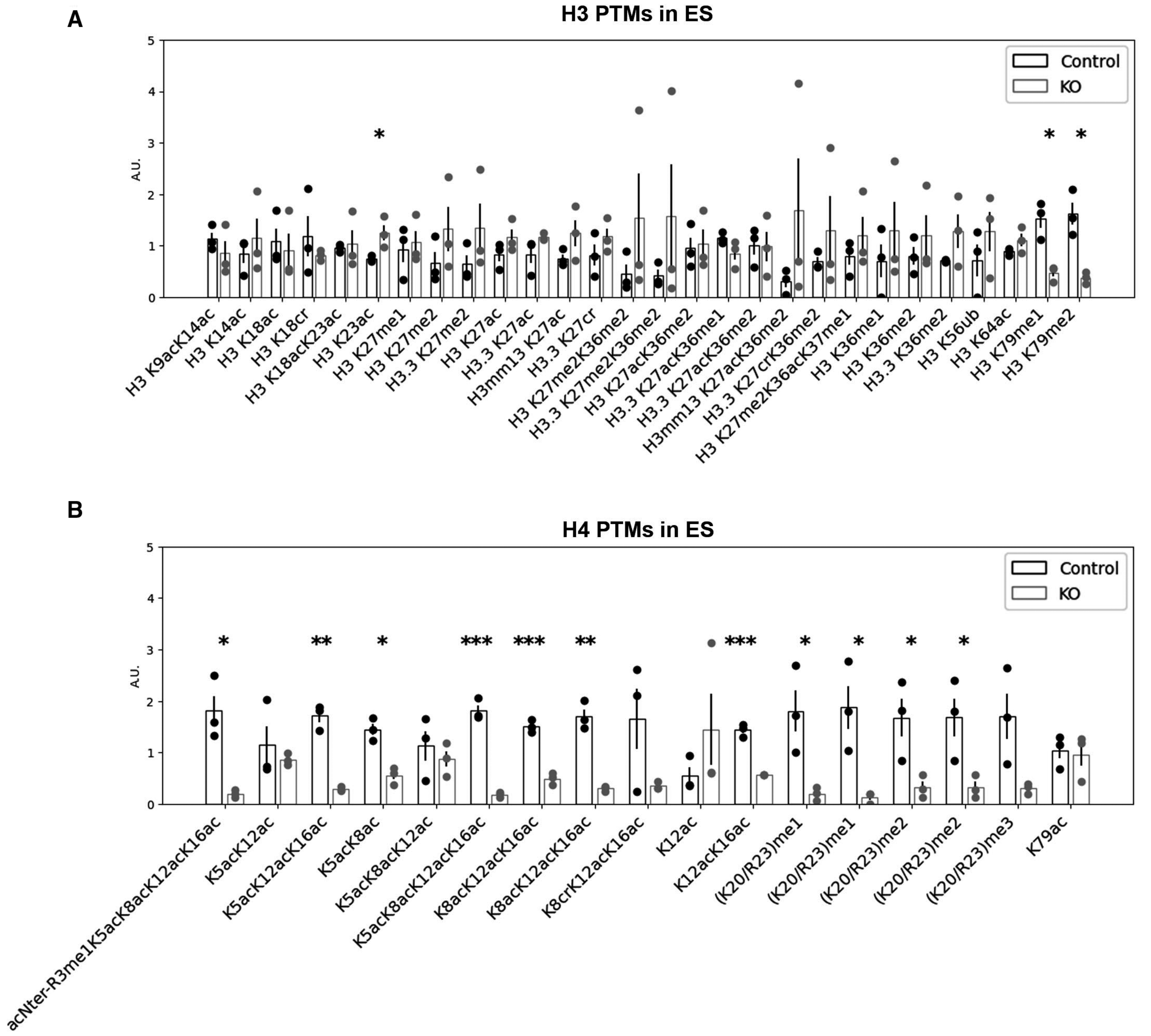

Figure 4. ***Dot1l*-KO modifies the chromatin of elongating/condensing spermatids.**

A, B Bar plots showing the quantification of post-translational modifications (PTMs) in histones H3 and H4 in ES (elongating/condensing spermatids) by LC–MS/MS. After normalization to be at constant amount of histone H3 or H4 in each analyzed sample (see Materials and Methods), mass spectrometry signals were divided by the average signal in both conditions (CTL and KO), so as to be able to represent all peptides in the same figure, whatever their MS intensity. Quantitative data obtained on biological replicates ($N = 3$ for CTL and 3 for KO) were plotted as dots, and the height of the bars indicates the average value (mean ± standard deviation, SD). *T*-tests were performed to estimate the possible significant difference of modified peptide abundances between the two genotypes. **P*-value < 0.05, ** *P*-value < 0.005, and ****P*-value < 0.0005. When interpreting our mass spectrometry data against the database MS_histoneDB, we were able to identify various H3 variants, namely canonical H3 and variants H3.3, testis-specific H3t and H3mm13. "H3" indicates sequences shared among several variants; "H3.3," "H3t," or "H3mm13", each variant. A.U. = arbitrary units.

Data information: See also Figs EV3 and 4 and Appendix Fig S4.
Source data are available online for this figure.

and several of them have been implicated in sperm motility via their effects on flagellum differentiation and/or sperm energy production (Maruyama *et al*, 2016; Toure, 2019; Kuang *et al*, 2021).

In agreement with the downregulation of pathways related to apoptosis, *Bcl6*, *Jak3*, and *Tsc22d3* which encode proteins with anti-apoptotic effects (Thomis *et al*, 1997; Kurosu *et al*, 2003; Aguilar *et al*, 2014) were found significantly downregulated in RS. *Blc6* codes for a transcription repressor (Chang *et al*, 1996) and appears to be a direct target of DOT1L since it is downregulated upon *Dot1l* loss, and enriched in H3K79me2 in wild-type RS (Fig 5E).

 © 2023 The Authors

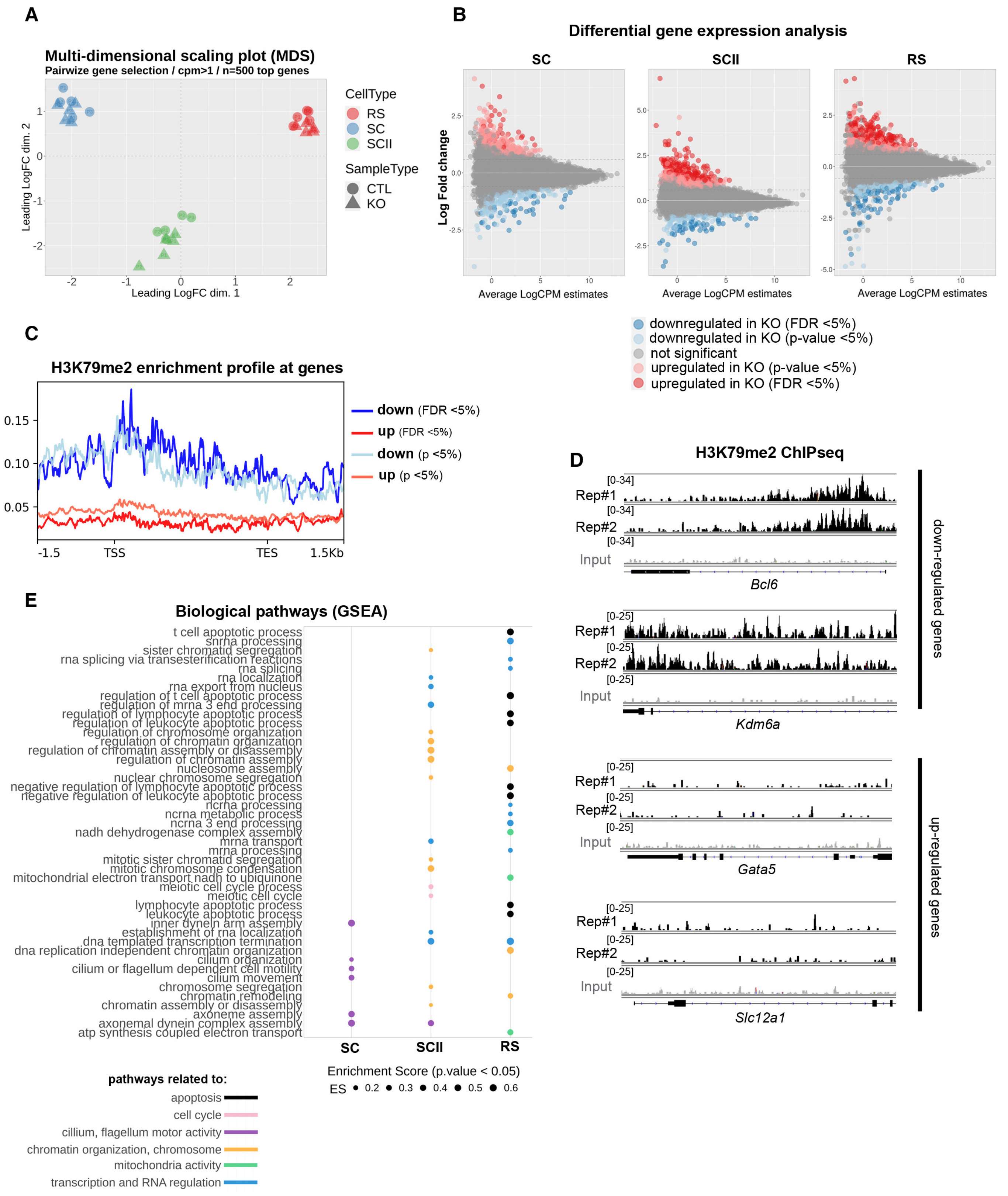
A
Multi-dimensional scaling plot (MDS)
Pairwize gene selection / cpm>1 / n=500 top genes
Leading LogFC dim. 2
Leading LogFC dim. 1
CellType
RS
SC
SCII
SampleType
CTL
KO
B
Differential gene expression analysis
SC
SCII
RS
Log Fold change
Average LogCPM estimates
downregulated in KO (FDR <5%)
downregulated in KO (p-value <5%)
not significant
upregulated in KO (p-value <5%)
upregulated in KO (FDR <5%)
C
H3K79me2 enrichment profile at genes
down (FDR <5%)
up (FDR <5%)
down (p <5%)
up (p <5%)
-1.5
TSS
TES
1.5Kb
D
H3K79me2 ChIPseq
Rep#1
Rep#2
Input
Bcl6
Kdm6a
Gata5
Slc12a1
down-regulated genes
up-regulated genes
E
Biological pathways (GSEA)
t cell apoptotic process
snrna processing
sister chromatid segregation
rna splicing via transesterification reactions
rna splicing
rna localization
rna export from nucleus
regulation of t cell apoptotic process
regulation of mrna 3 end processing
regulation of lymphocyte apoptotic process
regulation of leukocyte apoptotic process
regulation of chromosome organization
regulation of chromatin organization
regulation of chromatin assembly or disassembly
regulation of chromatin assembly
nucleosome assembly
nuclear chromosome segregation
negative regulation of lymphocyte apoptotic process
negative regulation of leukocyte apoptotic process
ncrna processing
ncrna metabolic process
ncrna 3 end processing
nadh dehydrogenase complex assembly
mrna transport
mrna processing
mitotic sister chromatid segregation
mitotic chromosome condensation
mitochondrial electron transport nadh to ubiquinone
meiotic cell cycle process
meiotic cell cycle
lymphocyte apoptotic process
leukocyte apoptotic process
inner dynein arm assembly
establishment of rna localization
dna templated transcription termination
dna replication independent chromatin organization
cilium organization
cilium or flagellum dependent cell motility
cilium movement
chromosome segregation
chromatin remodeling
chromatin assembly or disassembly
axoneme assembly
axonemal dynein complex assembly
atp synthesis coupled electron transport
SC
SCII
RS
Enrichment Score (p.value < 0.05)
ES 0.2 0.3 0.4 0.5 0.6
pathways related to:
apoptosis
cell cycle
cillium, flagellum motor activity
chromatin organization, chromosome
mitochondria activity
transcription and RNA regulation

Figure 5.

Figure 5. *Dot1l*-KO leads to the deregulation of genes involved in flagellum function, transcription regulation, chromatin organization, mitochondria function and apoptosis.

A Multi-dimensional scaling plot on the 500 top genes in *Dot1l*-KO versus CTLs primary spermatocytes (SC), secondary spermatocytes (SCII), and round spermatids (RS) using pairwise gene selection.

B MD plot of the genes found deregulated in *Dot1l*-KO versus CTLs SC, SCII, and RS, using differential gene expression analyses with two different parameters (FDR < 0.05 and *P*-value < 0.05).

C H3K79me2 enrichment profile at deregulated genes (same parameters as in B).

D H3K79me2 enrichment profile at selected deregulated genes, in two biological replicates of ChIP-Seq performed in wild type RS (Rep#1 and 2). The input value is shown in gray.

E GSEA analysis of RNA-Seq data from *Dot1l*-KO versus CTL SC, SCII and RS. The figure shows all the biological pathways found significantly downregulated in *Dot1l*-KO primary spermatocytes (SC), secondary spermatocytes (SCII), and round spermatids (*P* < 0.05), ranked by their enrichment score (ES).

Data information: See also Fig EV5 and Appendix Fig S5.

Interestingly, an unsupervised search for DNA motifs identified BCL6/BCL6B DNA binding site as the most significantly enriched motif in the promoter of upregulated genes (15 upregulated genes, Appendix Fig S5D). This finding is in agreement with a model in which downregulated genes are direct H3K79me2/DOT1L targets while upregulated genes are indirect targets.

Discussion

In the present study, using a conditional knockout mouse model, we demonstrate that the H3K79 methyltransferase DOT1L is essential for gene regulation and chromatin remodeling during spermatid differentiation.

DOT1L is highly conserved among eukaryotes. In mammalian it has been extensively studied and found to be involved in many different biological processes such as mixed lineage leukemia (Okada *et al*, 2005; Nguyen *et al*, 2011), cell cycle (Kim *et al*, 2014), development (Jones *et al*, 2008), reprogramming (Onder *et al*, 2012), DNA damage repair (Lin *et al*, 2009; Zhu *et al*, 2018), or transcription activation (Steger *et al*, 2008; Wang *et al*, 2008). In the male germline, DOT1L is highly expressed at the RNA and protein levels, particularly in postmeiotic cells (Dottermusch-Heidel *et al*, 2014a,b; Moretti *et al*, 2017). Before meiosis, DOTL1 has been found to be required for the self-renewal of adult stem cells. Indeed, with a *Dot1l* KO induced by a Cre recombinase expressed earlier than the one used in the present study, Lin *et al* (2022) observed a progressive (age-dependent) loss of all male germ cell types, ultimately resulting in a Sertoli cell only syndrome (i.e., absence of germ cells). In the *Dot1l*-KO males we characterized here, we also found empty tubules as well as a decrease of all germ cell types, but milder, which enabled us to address the role of DOT1L in adult postmeiotic germ cells (i.e., spermatids). Strikingly, we found that *Dot1l*-KO spermatozoa present multiple anomalies such as thinner and distorted flagella, cytoplasmic retention and impaired nuclear compaction. As a result, KO spermatozoa are less motile and their fertilizing ability is compromised. These defects are associated with the deregulation of hundreds of genes in *Dot1l*-KO meiotic and postmeiotic germ cells. Previous studies have shown that spermatid differentiation is associated with the specific expression (or highly enriched expression) of thousands of genes, and that this genetic program starts as early as in primary spermatocytes (Soumillon *et al*, 2013; da Cruz *et al*, 2016; Chen *et al*, 2018; Green *et al*, 2018; Ernst *et al*, 2019). Here, our analyses indicate that pathways related to "cilium/flagellum assembly or motility" are deregulated from the spermatocyte stage, and those related to "nucleosome assembly, chromatin remodelling" are deregulated in secondary spermatocytes and round spermatids. A significant proportion of *Dot1l*-KO spermatids undergo apoptosis and this was also visible by RNA-Seq with the deregulation of "apoptosis" related pathways in RS, including the downregulation of genes with anti-apoptotic effects such as *Bcl6* or *Jak3* (Thomis *et al*, 1997; Kurosu *et al*, 2003). Interestingly, several potential BCL6 target genes were identified among the genes which are upregulated in *Dot1l*-KO spermatids. Like other upregulated genes, most of them are devoid of H3K79me2 in wild type spermatids and may not be directly regulated by DOT1L. In contrast, *Bcl6* and many other downregulated genes in *Dot1l*-KO spermatids are marked by H3K79me2 in wild-type cells and are likely directly regulated by DOT1L. Our findings are in agreement with recent studies in which *Dot1l* knockout was investigated in B cells, T cells or cardiomyocytes. Each time, H3K79me2 was found to be enriched at the body of downregulated genes but not of upregulated genes (Kwesi-Maliepaard *et al*, 2020; Aslam *et al*, 2021; Cattaneo *et al*, 2022).

Strikingly, many genes encoding members of the solute carrier family were deregulated in *Dot1l*-KO male germ cells. Several members of this family such as *Slc22a14* and *Slc26a8* have previously been implicated in sperm motility via their effects on flagellar differentiation and/or sperm energy production (Maruyama *et al*, 2016; Toure, 2019; Kuang *et al*, 2021); the deregulation of *Slc* genes with similar function could, therefore, contribute to the sperm flagellar and motility defects of *Dot1l*-KO males. Overall, these data indicate that *Dot1l*-KO-induced gene deregulation is responsible for the multiple defects of postmeiotic male germ cell differentiation.

In addition to being involved in the regulation of spermatid gene expression, we show that DOT1L is essential for spermatid chromatin remodeling and subsequent reorganization and compaction of spermatozoon genome. Indeed, the chromatin of *Dot1l*-KO spermatozoa is less compact, and characterized by a higher level of retained histones, of transition protein 2, and of immature forms of protamine 2, as well as altered protamine 1/2 ratio. Several other studies have observed similar anomalies in particular retained histones and immature forms of protamine 2, as a result of incomplete histone-to-protamine transition, such as *Tnp1*-KO (*transition protein 1*; Yu *et al*, 2000), *H2al2*-KO (*H2A histone family member L2A*; Barral *et al*, 2017), deletion of the mouse Y chromosome long arm (Yamauchi *et al*, 2010), or *Kat2a* –KO (a.k.a. *Gcn5)* which encodes the lysine acetyltransferase 2A (Luense *et al*, 2019). Similarly, a recent study has shown that aberrant pre-PRM2 cleavage leads to altered levels of histone variants and transition proteins in sperm

 © 2023 The Authors

(Arevalo *et al*, 2022). In all those mouse models as well as in the *Dot1l*-KO presented here, abnormal sperm chromatin content is associated with fertility problems. This is also the case in humans, since impaired protamine 1/2 ratio and accumulation of pre-PRM2 are associated with male infertility (Torregrosa *et al*, 2006; de Mateo *et al*, 2009).

In elongating spermatids, histones—in particular histone H4—are hyperacetylated prior to their replacement. This phenomenon is a prerequisite for histone-to-protamine transition as it creates a permissive state for histone removal (for reviews see Oliva, 2006; Rathke *et al*, 2014; Bao & Bedford, 2016). H3K79me2/3 levels peak at the time of H4 hyperacetylation (Dottermusch-Heidel *et al*, 2014a, b; Moretti *et al*, 2017) and we show here that both H3K79 methylation and H4 hyperacetylation are drastically and specifically reduced in *Dot1l*-KO elongating spermatids. Several acetyltransferases are involved in H4 hyperacetylation such as KAT2A, CBP-P300, or NuA4 complex (Boussouar *et al*, 2014; Shiota *et al*, 2018; Luense *et al*, 2019) but none of them was found deregulated in *Dot1l*-KO spermatids. We, therefore, propose that the decrease in histone acetylation is a consequence of impaired H3K79 methylation. In other contexts than the male germline, H4 acetylation and H3K79 methylation have previously been shown to influence each other in different ways. In 2016, Gilan *et al* proposed that DOT1L-mediated H3K79me2 leads to a more open chromatin which facilitates the recruitment of the histone acetyltransferase P300 which, in turn, increases histone H4 acetylation (in particular, H4K5ac), in a cell model of acute myeloid leukemia (Gilan *et al*, 2016). In contrast, H4K16ac has been shown to stabilize the binding of yeast Dot1 and human DOT1L on the nucleosome and consequently stimulates its methyltransferase activity *in vitro* (Valencia-Sanchez *et al*, 2021). In elongating spermatids, H3K79 methylation and H4 acetylation could therefore contribute to increase each other signal. It would be interesting to test if H3K79 methylation level is affected in spermatids in which histone acetylation is impaired, for instance in *Kat2a* knockout spermatids.

In our study, we also observed a decrease in the three methylation states of H4K20 in elongating spermatids. This is in line with the observation made by Jones *et al* (2008) that H4K20 trimethylation level is reduced at centromeres and telomeres of *Dot1l*-KO embryonic stem cells. H3K9 di-methylation (but not trimethylation) was also found reduced in KO embryonic stem cells, but this histone PTM could not be detected in our analyses. Methylation of H4K20 has been shown to be associated with chromatin accessibility. H4K20me1, by changing the conformation of nucleosomes, increases accessibility for other factors involved in DNA processing and expression (Shoaib *et al*, 2021). In contrast, H4K20me2 and H4K20me3 are associated with chromatin compaction (Evertts *et al*, 2013). For the moment, the impact of these marks during spermatid differentiation is unknown, but H4K20me3 is one of the most dynamic histone modifications during spermatogenesis, with a peak in elongating spermatids; H4K20me2 follows the same pattern but is less abundant than H4K20me3 (Luense *et al*, 2016; Wang *et al*, 2021). One can, therefore, speculate that H4K20 methylation is also important for histone to protamine transition. Interestingly, Ho *et al* (2021) have found that the H4K20me1 methyltransferase, SET8, and DOT1L (which is a non-SET domain methyl-transferase) bind to the nucleosomal acidic patch by a similar mechanism. Further work will be needed to understand the mechanism by which DOTL1 and/or H3K79 methylation may influence H4K20 methylation.

Other, more modest quantitative changes in histone PTMs and variants were observed in elongating spermatids. They could be compensatory mechanisms for H3K79 methylation or H4 acetylation decrease, as observed in *Th2b* knock-out spermatids where H2B was upregulated and several histone PTMs changed to compensate for the loss of H2B variant TH2B (Montellier *et al*, 2013). The deregulation of genes associated to "chromatin function" could also contribute to the changes in chromatin content observed in *Dot1l*-KO spermatids.

In conclusion, our data show that DOT1L is indispensable for the differentiation of postmeiotic germ cells into functional spermatozoa. Indeed, DOT1L regulates the expression of genes essential for spermatid differentiation and, DOT1L-mediated H3K79 methylation is necessary for proper histone H4 hyperacetylation in elongating spermatids and for an efficient histone-to-protamine transition and subsequent compaction of the sperm chromatin.

Materials and Methods

Mouse strains and genotyping

Conditional *Dot1l* knockout mice were obtained from the Knockout Mouse Project (CSD29070; to F.V.D.). This line has previously been described in (Jo *et al*, 2011). In these mice, *Dot1l* exon 2 is flanked by loxP sites (see Appendix Fig S1D). Recombination of loxP sites by Cre recombinase leads to the deletion of exon 2 and consequently a frameshift and premature stop codon in *Dot1l* Coding DNA sequence. Tg(*Stra8-iCre*)1Reb/J were obtained from the Jackson lab (JAX stock #017490; Sadate-Ngatchou *et al*, 2008). All animals analyzed in this study were from a C57BL6/J background. Genotypes of animals were determined by polymerase chain reaction (PCR) using *Dot1l* primers F + R1 + R2 (Appendix Fig S1D) or *iCre* primers as described on the Jackson lab website. The latter PCR was performed with *Ymtx* internal control (see Appendix Table S1 for primer list and sequences).

Unless specified, all experiments were performed on (adult) 3–5-month-old males. Reproductive parameters were analyzed on two types of *Dot1l* knocked-out animal models. First, on animals hosted in a conventional animal house (cah) and of the following genotypes: *Dot1l*$^{Fl/Fl}$; *Stra8-Cre* mice (DOT1Lcah) and *Dot1l*$^{Fl/Fl}$ siblings (without *Stra8*-Cre transgene) as controls (CTLcah). Second, on animals hosted in a Specific Pathogen-Free (SPF) animal house and of the following genotypes: *Dot1l*$^{Fl/\Delta}$; *Stra8-Cre* (hereafter called DOT1L-KO) and *Dot1l*$^{Fl/Fl}$ siblings as controls (hereafter called CTL). Analyses of the reproductive parameters of both types of KO males (*Dot1l*$^{Fl/Fl}$;*Stra8-Cre* or *Dot1l*$^{Fl/\Delta}$; *Stra8-Cre*) gave similar results (Appendix Fig S2A and B). Heterozygous *Dot1l*$^{Fl/\Delta}$ males (HET) were also analyzed. They did not differ from controls (Appendix Fig S2A–E). *Dot1l*$^{Fl/\Delta}$; *Stra8-Cre* animals were generated because it had been shown that *Stra8-iCre* is not 100% efficient (Bao *et al*, 2013). In these animals, one allele of *Dot1l* is floxed and one allele is already deleted (Δ) to increase the efficiency of floxed exon excision upon *Cre* recombinase expression. Nevertheless, some remaining DOTL1 protein signal was observed by western blot and Immunofluorescence (see Fig 1B and D and Appendix Fig S1E–I).

All other phenotypic analyses and all molecular analyses were performed on *Dot1l*$^{Fl/\Delta}$; *Stra8-Cre*, hereafter called *Dot1l*-KO, and on *Dot1l*$^{Fl/Fl}$ siblings as CTL. Mice from mTmG line (Muzumdar *et al*, 2007) were crossed to *Stra8-Cre* line to verify *Stra8-Cre* recombinase location and activity.

The mice were fed ad libitum with a standard diet and maintained in a temperature- and light-controlled room. Animal procedures were approved by Universite de Paris ethical committee (Comite d'Ethique pour l'Experimentation Animale; registration number CEEA34.JC.114.12, APAFIS 14214-2017072510448522v26).

Fertility tests

Dot1l-KO and CTL males, aged from 8 to 10 weeks, were housed with two wild-type C57BL6/J females per cage (aged from 6 weeks, Janvier Labs, France) for up to 4 months. Vaginal plugs were checked and females separated when found. For each group, litter size and number were assessed.

Another series of test were performed too evaluate the fertilization rate following mating (*in vivo* fertilization). For this WT females were first superovulated by injection of 5 IU of PMSG (pregnant mare's serum gonadotrophin; Intervet, France) followed by 5 IU of hCG (human chorionic gonadotrophin; Intervet) 48 h later. Females were then mated with WT or *Dot1l*-KO males at night and the next day females with a vaginal plug were isolated. Oocytes were collected from ampullae of oviducts and freed from the cumulus cells by brief incubation at 37°C with hyaluronidase (Sigma, St. Louis, MO, USA) in M2 medium (Sigma). Oocytes were rinsed and the number of fertilized oocytes was evaluated using DAPI immunefluorescent staining to visualize the DNA.

IVF

Oocyte preparation

WT C57BL6/J female mice aged 6–8 weeks (Janvier Labs, France) were superovulated with 5 IU of pregnant mare serum gonadotropin (PMSG) and 5 IU human chorionic gonadotropin (hCG; Intervet, France) 48 h apart. About 14 h after hCG injection, animals were sacrificed by cervical dislocation. Cumulus oocyte complexes were collected by tearing the ampulla wall of the oviduct, placed in Ferticult medium (FertiPro N.V, Belgium) supplemented with 3% BSA (Sigma–Aldrich), and maintained at 37°C under 5% CO_2 in air under mineral oil (FertiPro N.V, Belgium). When experiments were performed with Zona-free oocytes, cumulus cells were first removed by a brief exposure to hyaluronidase IV-S (1 mg/ml, Sigma–Aldrich). The zona pellucida was then dissolved with acidic Tyrode's (AT) solution (pH 2.5; Sigma–Aldrich) under visual monitoring. Zona-free eggs were rapidly washed five times and kept at 37°C under 5% CO_2 in air for 2–3 h to recover their fertilization ability.

Capacitated sperm preparation: mouse spermatozoa were obtained from the cauda epididymides of DOT1L-KO, CTL, HET and WT C57BL6/J males (aged 8–12 weeks) and capacitated at 37°C under 5% CO2 for 90 min in a 500 µl drop of Ferticult medium supplemented with 3% BSA, under mineral oil.

In vitro fertilization: cumulus-intact and Zona-free eggs were inseminated with capacitated spermatozoa for 3 h in a 100 µl drop of Ferticult medium, 3% BSA at a final concentration of 10^6 or 10^5 per ml, respectively. Then, they were washed and directly mounted in Vectashield/DAPI (Vector laboratories, CA, USA) for observation under UV light (Nikon Eclipse E600 microscope). Only oocytes showing at least one fluorescent decondensed sperm head within their cytoplasm were considered fertilized.

Testicular germ cell purification by elutriation or FACS

Germ cells were purified from adult males using fluorescence-activated cell sorting (FACS) or elutriation as previously described in (Cocquet *et al*, 2009; Comptour *et al*, 2014) and more recently in (Crespo *et al*, 2020). Elutriated fractions with a purity ~ 95–99% for elongating/condensing spermatids and ~ 80% for round spermatids, were used for LC–MS/MS analyses. Highly enriched fractions of primary or secondary spermatocytes (> 90%) and round spermatids (~ 99%) were used for RNA-Seq analyses. Flow cytometric analysis of testicular cell suspension were performed as previously described (Corbineau *et al*, 2017; Ragazzini *et al*, 2019).

Sperm collection and purification with Percoll

Spermatozoa were extracted from cauda epididymis in pre-warmed (37°C) M2 medium (Sigma-Aldrich) by gentle pressure. Then, epididymides were perforated with a thin needle and incubated in M2 for 10 min at 37°C to allow remaining sperm to swim up. For molecular analyses, spermatozoa were next purified on a Percoll gradient. In brief, spermatozoa were centrifuged at 2,000 *g* for 10 min and pellets incubated in somatic cell lysis buffer (1× PBS, 0.1% SDS, 0.5% Triton X-100) for 10 min on ice. Sperm cells were washed with PBS-BSA (1× PBS, 0.5% BSA, 2 mM EDTA) and briefly sonicated to remove flagella (ON 5 s – OFF 30 s × 3 Cycles, bioruptor Pico, Diagenode). The samples were transferred in low retention tubes, loaded on 50% Percoll (Sigma-Aldrich) and centrifuged at 2,500 *g* for 5 min to remove somatic cells and flagella. This step was repeated once and sperm cells were washed in PBS-BSA twice and counted (minimum 1,000 cells). The purity was > 99.7%. Spermhead pellets were snap-frozen in liquid nitrogen and stored at −80°C prior to use.

Nucleoplasmin decompaction

Experiments were performed as previously described in (Yamaguchi *et al*, 2018). Following Percoll purification, sperm heads were washed in 1× PBS, 0.5% Tween, permeabilized with 0.02 U/M Streptolysin O (Sigma-Aldrich) in 1× PBS for 10 min on ice and washed once with 1× PBS, 0.5% Tween. Cells were then incubated with 1× PBS containing 1 mM DTT for 10 min at 37°C, washed with 1× PBS, 0.5% Tween, resuspended in KH buffer (100 mM KCl, 20 mM Hepes-KOH, pH 7.7) in low retention tubes and stored at −80°C for later use. Ten million of sperm heads were resuspended in 1 mL NPM treatment buffer (20 mM HEPES-KOH, pH 7.7, 100 mM KCl, 2.5 mM MgCl2, 5 mM Na-But, 10 mM DTT, 1X complete EDTA-free protease inhibitors, 250 µM NPM) and incubated for 2 h at 37°C in a Thermomixer (Mixer HC, Starlab) at 1,000 rpm. After centrifugation at 20,000 *g* for 5 min at 4°C, cells were washed with 5 mM Na-But in 1× PBS, 0.5% Tween twice and sperm heads were fixed with 1% PFA for 10 min at RT, quenched with 250 mM glycine and washed in 5 mM Na-But in PBS/Tween 0.5% twice.

After fixation, sperm pellets were resuspended in NP40 Buffer (10 mM Tris–HCl, pH 8.0, 10 mM NaCl, 0.5% NP40, 1× complete

 © 2023 The Authors

EDTA-free protease inhibitors, Sigma Aldrich) and incubated on ice for 30 min. Samples were washed with 1× PBS, 0.5% Tween, 5 mM Na-But, and resuspended in 200 μl of sonication buffer (10 mM Tris–HCl, pH 8.0, 20% glycerol, 0.25% SDS, 5 mM Na-But, 1× complete EDTA-free protease inhibitors). Sperm cells were sonicated (ON 30s – OFF 30s x13 cycles, Bioruptor Pico, Diagenode) and the sheared chromatin was centrifuged at 20,000 *g* for 10 min at 4°C. Supernatants were transferred in new 1.5 ml tubes and pellets resuspended in 200 μl of KH buffer. Prior to western blot analyses, both supernatant and pellets were resuspended in 4× NuPage/10% β-mercapto-ethanol (ThermoFisher), heated 10 min at 95°C and briefly sonicated.

Histone extraction from spermatozoa

Experiments were performed as described in (Crespo *et al*, 2020) with minor modifications. In brief, 5 million of Percoll-purified and sonicated spermheads were incubated in 50 mM DTT for 30 min (at 4°C) then mixed with sulfuric acid (0.4 M final volume), sonicated and acid extracted with 20% trichloroacetic acid (TCA). Histone pellets were washed with cold acetone containing 0.05% HCl, dried at room temperature, and resuspended in 50 μl of SDS–PAGE loading buffer containing 10% β-mercaptoethanol.

Western blot

Electrophoresis was performed in polyacrylamide gels at 120 V in denaturating buffer containing 25 mM Tris Base, 190 mM glycine, 0.1% SDS, and proteins were transferred on nitrocellulose membranes (GE Healthcare). Membranes were then rinsed and incubated 3 min in Ponceau stain to visualize transfer efficiency. Then membranes were incubated in 1× PBS, 0.01% Tween, 5% milk. All primary antibodies were incubated over night at 4°C (see Appendix Table S2 for references and dilutions) and 2 h at room temperature for secondary antibodies. The revelation was performed with SuperSignal West Pico Plus® ECL from ThermoFisher (34580) and Immobilon ECL Ultra Western HRP substrate from Millipore (WBULS0100) on ImageQuant™ LAS 4000 imager.

Extraction and analysis of protamines

PRM1/PRM2 protamine ratio was analyzed following procedures of protamine-rich fraction of sperm nuclear proteins extraction and analysis previously described, with minor modifications (Soler-Ventura *et al*, 2018). Briefly, histones and other basic proteins loosely attached to DNA were extracted incubating sperm in 0.5 M HCl for 10 min at 37°C after vortexing, and centrifuged at 2,000 *g* 20 min at 4°C. This step was repeated three times. Resulting pellet was resuspended in 0.5% Triton X-100, 20 mM Tris–HCl (pH 8), and 2 mM $MgCl_2$. After centrifugation at 8,940 *g* 5 min at 4°C, the sediment was resuspsended in milliQ H_2O with 1 mM PMSF, centrifuging again at the same conditions. Chromatin was then denaturated by resuspending the pellets in 20 mM EDTA, 1 mM PMSF, 100 mM Tris–HCl- pH8 and adding 1 volume of 575 mM DTT in 6 M GuHCl prior vortexing. The solution was incubated at 37°C with 0.8% 4-vynilpyridine to inhibit cysteine disulfide bonds for 30 min, vortexing every 5 min, in fumehood and in dark conditions. Chromatin was then precipitated with a minimum of 10 min incubation with cold ethanol at −20°C, followed by centrifugation 12,880 *g* for 15 min at 4°C. Basic nuclear proteins (protamine-enriched fraction) were extracted from DNA incubating with 0.5 M HCl at 37°C and recovered in the supernatant after centrifugation at 17,530 *g* for 10 min at 4°C. Precipitation was carried out with 20% TCA on ice and centrifugation using the same conditions. Protamines were washed twice with 1% β-mercaptoethanol in acetone and dried out at room temperature. For in-gel quantification, dried purified extracts were resuspended 5.5 M urea, 20% β-mercaptoethanol, 5% acetic acid, and separated using acetic acid urea gel electrophoresis (AU-PAGE). Gels were stained with EZBlue™ Gel Staining Reagent (#G1041, Sigma Aldrich) and optic density of the bands corresponding to mouse PRM1 and PRM2 was quantified using Quantity One 1-D analysis software (BioRad, Hercules, CA, USA) to calculate the PRM1/PRM2 ratio. PRM1/PRM2 ratios were normalized against the mean value of the control group.

For western blot detection, basic nuclear protein extracts from 2.1 M spermatozoa were loaded into AU-PAGE. Proteins were then transferred towards the negative pole onto a 0.45-μm pore size nitrocellulose membrane (88018, ThermoFisher) for 4 h at 4°C using an acidic transfer buffer consisting of 0.9 mM acetic acid. The rest of the procedure (blocking, antibody incubation, and ECL detection) was similar as for other western blot experiment (see dedicated paragraph above).

Histological analyses

Mouse testes were fixed in 4% buffered paraformaldehyde (PFA) for minimum 4 h, before they were cut in halves and incubated overnight in Bouin (Sigma). Testes were then washed in 70% ethanol at room temperature for 30 min, dehydrated, and embedded in paraffin. Four-micrometer sections were stained with periodic acid–Schiff (PAS).

Immunohistochemistry and immunofluorescence

Mouse testes were fixed in 4% buffered paraformaldehyde (PFA) for minimum 4 h, before they were cut in halves and incubated overnight in 4% PFA. Testes were then washed in 70% ethanol at room temperature for 30 min, dehydrated, and embedded in paraffin. Immunohistochemistry or immunofluorescence experiments were performed on 4-μm sections using Novolink polymer detection system (7140-K, Leica Micro-systems) according to the manufacturer's instructions, or following the procedure described in (Comptour *et al*, 2014) with the following modifications. Permeabilization was performed for 15 min in 1× PBS, 0.5% Triton X-100. Blocking was performed for 30 min to 1 h at room temperature in 1× PBS, 0.1% Tween, 1% BSA. Primary antibody was incubated overnight at 4°C (dilution and reference of each antibody are available in Appendix Table S2). Some slides were counterstained with Hematoxylin. For immunofluorescence experiments, lectin (L-21409 or L-32459, ThermoFisher) was diluted at 1/500 in 1× PBS and incubated for 1 h at room temperature along with secondary antibodies (see Comptour *et al*, 2014). Lectin was used to stain the developing acrosome and determine the stage of testis tubules as described in (Ahmed & de Rooij, 2009). DAPI (in VECTASHIELD Mounting Medium, Vectorlab) was used to stain nuclei. TUNEL assay was performed on 4-μm paraffin-embedded testicular sections using In Situ Cell Death Detection Kit, Fluorescein as described by the manufacturer (Roche,

© 2023 The Authors

Sigma-Aldrich). GFP immunofluorescence pictures were taken with a NikonE600 microscope using the Nikon Digital Sight SD-V3 camera (DS-Fi1) with the NIS software. All other immunofluorescence pictures were taken with an Olympus BX63 microscope and analyzed using ImageJ 1.48v (http://imagej.nih.gov/ij/). Immunohistochemistry pictures were taken with Perkin Elmer Lamina slide scanner and analyzed using CaseViewer software.

Papanicolaou staining of spermatozoa

Spermatozoa were collected from cauda epididymides in M2 medium (Sigma-Aldrich) and spread onto a Superfrost Plus slide (ThermoFisher). Cells were fixed in 4% PFA for 10 min and stained using Papanicolaou staining. Briefly, the slides were washed in 95% ethanol, incubated in Harris hematoxylin for 3 min for nucleus counterstaining, washed and stained with OG-6 dye (RAL diagnostics 05.12013, Martillac, France) and with EA-50 (RAL diagnostics 05.12019, Martillac, France). The slides were then dehydrated and mounted with permanent mounting medium. Spermatozoa were then observed with NikonE600 optic microscope and pictures were taken with the 40X objective.

Electron microscopy

Ten to 20 million epidydimal spermatozoa were collected in 2 ml of fresh M2 medium (Sigma-Aldrich) prewarmed at 37°C. After pelleting the sperm at room temperature (300 *g*, 10 min), the pellet was fixed in 3% glutaraldehyde for 1 h before washing twice in 1× PBS (1,000 *g*, 10 min). Samples were then dehydrated in ethanol baths and incubated in propylene oxide before inclusion in gelatin. Slices of 60 nm were cut with a Diatome and observed with a *JEOL 1011* microscope at a magnification of 1,500× or 2,000×.

Computer assisted sperm motility analysis (CASA)

Immediately after collection of epididymal spermatozoa in M2 medium (Sigma-Aldrich), sperm motility was analyzed by computer-assisted sperm analysis (CASA) systems CASA CEROS II (Hamilton Thorne, Beverly, MA, USA), using a Zeiss microscope.

Sample preparation for RNA-Seq

Total RNA from 150,000 to 600,000 cells of sorted primary spermatocytes (SCI), secondary spermatocytes (SCII) and round spermatids (RS) was extracted using the Ambion RNAqueous micro kit (ThermoFisher) following manufacturers' instructions. For each cell type, five replicates were analyzed. Quantity and quality were assessed using Bioanalyzer chips (ThermoFisher). Libraries were prepared using the NEBNext Ultra II Directional RNA Library Prep Kit (New England Biolabs) according to supplier recommendations. Paired-end sequencing of 100-bp reads was then carried out on the Illumina HiSeq4000 system.

RNA-Seq analyses

Snakemake was used for RNA-seq analyses (Koster & Rahmann, 2018; v. 3.9.0). Adaptors were trimmed and reads with poor quality (quality < 20) were filtered out with BBduk from BBTools (v. 38.23, https://sourceforge.net/projects/bbmap/). Alignment was performed on the mouse genome build mm10 (GRCm38.p6) using STAR (Dobin & Gingeras, 2015; v. 2.7.2d) and Gencode vM19 gene annotation GTF with following arguments: --outMultimapperOrder Random --quantMode Genecounts --sjdbOverhang 74. For each sample, the number of aligned reads was converted in cpm (count per million). Genes with an expression level of at least > 1 cpm in a minimum of two samples were included in the analysis, to exclude genes with a very low expression. Differential expression analysis was carried out using DESeq2 (Love *et al*, 2014) and edgeR (Robinson *et al*, 2010) packages using default parameters and a design using genotype and cell type. Differentially expressed genes (FDR < 5%) were obtained using glmTreat edgeR's function which conducts a modified likelihood ratio test (LRT) against the fold change threshold (> 1.5). This method is based on TREAT method (McCarthy & Smyth, 2009). Two analyses were performed in parallel: one selecting the deregulated genes with a FDR < 5% (adjusted *P*-values), and one selecting deregulated genes with a *P*-value < 5% (non-adjusted *P*-values). On this second category of genes, the FDR was calculated *a posteriori* using Benjamini–Hochberg correction (Benjamini & Hochberg, 1995) and was confirmed to be < 5%. Enrichment analysis was performed with GSEA (v. 4.0.3, c5.all.v7.1.symbols.gmt, GO cellular components, biological pathways, or all) on gene expression cpm values between KO and control samples (Subramanian *et al*, 2005; Appendix Fig S5E). Beforehand, mouse Ensembl gene ID were converted to human Ensembl gene ID (hg38) with the biomaRt R package (Durinck *et al*, 2005). The search for DNA motif in the promoter regions of deregulated genes was performed using HOMER (Heinz *et al*, 2010) with the findMotifs.pl function and the following parameters: -start -2000 -end 0.

To generate Appendix Fig S1B, we used https://rgv.genouest.org/ (Darde *et al*, 2015, 2019).

ChIP-Seq analyses

Snakemake was used for ChIP-Seq analyses. Adaptor were trimmed and filtered using the same parameters than RNA-Seq analyses. Alignment was performed on the mouse genome (mm10) using bowtie2 (Langmead & Salzberg, 2012) with the following arguments: --local. Peak calling was process with MACS2 (Zhang *et al*, 2008) with default parameters against input file to obtains peaks enrichment score of H3K79me2. Mitochondrial peaks and peaks with FDR > 5% were filtered. Peaks were annotated with the R package ChIPseeker (Yu *et al*, 2015) using the annotatePeak() function with the following parameters tssRegion = c(−3000,3000).

Code availability

The fully reproducible and documented analysis for RNA-Seq and ChIP-Seq is available on github at https://github.com/ManonCoulee/RNAseq_DOT1L_Blanco_2022.

MS quantification of histone PTMs and of histone variants

To identify histone PTMs, samples were prepared and processed essentially as described in Crespo *et al* (2020). Briefly, histones were precipitated by acid extraction from whole testes which were

© 2023 The Authors

beforehand reduced into powder using a pestle and mortar on dry ice, or from elutriated elongating/condensing spermatids (ES). They were resuspended in loading gel buffer (LDS sample buffer reference 84788, supplemented with NuPAGE sample reducing agent reference NP0009, ThermoFisher) and separated on a 4–12% NuPAGE acrylamide gel (reference NP0321BOX, ThermoFisher). After blue staining (SimplyBlue SafeStain, ThermoFisher), gel bands corresponding to H3 (at about 17 kDa) and H4 (at about 14 kDa) and two gel slices in-between containing H2A and H2B, were cut and then reduced with dithiothreitol, alkylated with iodoacetamide and in-gel digested with 0.1 μg trypsin (V511, Promega) per slice using a Freedom EVO150 robotic platform (Tecan Traging AG, Switzerland). Alternatively, to analyze the relative abundance of histone variants between conditions, histone samples were simply loaded on a gel and migrated for a few millimeter. The whole zone was cut for robotic in-gel tryptic digestion. The resulting tryptic peptides were analyzed by a liquid chromatography–tandem mass spectrometry coupling made up of a C18 reversed-phase capillary column (75 μm i.d. × 25 cm ReproSil-Pur C18-AQ, 1.9 μm particles, Cluzeau, France) using the UltiMate™ 3000 RSLCnano system (ThermoFisher) coupled to a Q-Exactive HF mass spectrometer (ThermoFisher). The mobile phases consisted of water with 0.1% formic acid (A) and acetonitrile with 0.08% (v/v) formic acid (B). Peptides were eluted with a gradient consisting of an increase of solvent B from 2.8% to 7.5% for 7.5 min, then from 7.5% to 33.2% over 33.5 min and finally from 33.2% to 48% over 6.5 min. Mass spectrometry acquisitions were carried out by alternating one full MS scan with Orbitrap detection acquired over the mass range 300 to 1,300 m/z and data-dependent MS/MS spectra on the 10 most abundant precursor ions detected in MS. The peptides were isolated for fragmentation by higher energy collisional dissociation (HCD) with a collision energy of 27.

Identification of modified peptides was obtained using our in-house established database of histone sequences, MS_histoneDB (El Kennani *et al*, 2017), completed with a list of 500 common contaminants. MS/MS data interpretation was carried out with the program Mascot (http://www.matrixscience.com/) with the following search parameters. The precursor and fragment mass tolerances were 5 ppm and 25 mmu, respectively; enzyme specificity was trypsin; the maximum number of trypsin missed cleavages was set to 5, carbamidomethyl (Cys) was specified as a fixed modification. We indicated as variable PTMs in Mascot acetylation, crotonylation, and ubiquitination (more precisely the dipeptide "GlyGly") of Lys residues, methylation and di-methylation of Lys/Arg and trimethylation of Lys, as well as N-terminal acetylation of proteins. Filtering of peptide identifications and quantification was performed by using the program Proline (Bouyssie *et al*, 2020). All peptide/spectrum matches of scores below 15 were filtered out; next, all identifications of modified peptides suggested by Mascot for H3 and H4 were visually validated by demanding that a continuous stretch of minimally 5 amino acids be identified in terms of b or y fragment ions, and by ascertaining PTM positioning on Lys/Arg residues. This score threshold is very low, yet it was relevant to use so as to be able to identify very short peptides, such as H3-K18modQLATK. When interpreting our mass spectrometry data against the database MS_histoneDB, we were able to identify and quantify various H3 variants, namely canonical H3 and variants H3.3, testis-specific H3t, and H3mm13. H3t differs from canonical H3 at residue 24, while H3.3 and H3mm13 differ from H3 at residues 31 and 29. To estimate the relative abundance of modified histone peptides between samples, normalization to be at constant amount of total H2A, H2B, H3, or H4 was done by dividing the raw MS signals of modified peptides by the sum of the MS signals of modified and unmodified peptides for each histone. To estimate the relative abundance of histone variants between *Dot1l*-KO and CTL samples, variants were quantified by Proline by relying only on peptides specific of these sequences. Samples were normalized to be at constant amount of histone H4. The mass spectrometry proteomics data have been deposited to the ProteomeXchange Consortium via the PRIDE (Perez-Riverol *et al*, 2019) partner repository with the dataset identifier PXD030734.

Statistical analysis

Chi-square and unpaired *t*-test were used to analyze IVF data. Sperm motility parameters were analyzed using a Mann–Whitney tests following angular transformation of percentages. To compare the incidence of sperm head abnormalities, and apoptotic (TUNEL+) spermatids between *Dot1l*-KO and CTL, percentages were converted in angles prior to performing *t*-tests. Student *t*-test (corrected for multiple testing when appropriate) was used for all other analyses (fertility, sperm count, testis weight, histology, histone PTM, western blot, and normalized PRM1/2 ratio quantifications). Statistical analyses for RNA-Seq/ChIP-Seq are described in the dedicated paragraphs.

Data availability

RNA-Seq data have been submitted to ENA repository under project number PRJEB50887 (https://www.ebi.ac.uk/ena/). H3K79me2 ChIP-Seq project number is PRJNA643726. The mass spectrometry proteomics data have been deposited to the ProteomeXchange Consortium via the PRIDE (Perez-Riverol *et al*, 2019) partner repository with the dataset identifier PXD030734 (https://www.ebi.ac.uk/pride/).

Expanded View for this article is available online.

Acknowledgements

We would like to thank Cochin Institute (INSERM U1016, CNRS UMR8104, Universite Paris Cité) core facilities, in particular, Alain Schmitt from the electron microscopy platform, and all the staff from the animal house, histology (HistIM), genomic (GENOM'IC), cytometry (CYBIO), and imaging (IMAG'IC) core facilities. We would also like to thank Guillaume Meurice for advices on bioinformatics analyses, and Aminata Touré and Marjorie Whitfield for advices and discussion on sperm morphology and motility analyses. M.Cr. and D.P. are grateful to their colleagues in EDyP for their support on LC–MS instruments and in informatics. This work was supported by the Agence Nationale de la Recherche (ANR-17-CE12-0004-01 to J.C., ANR-21-CE44-0035 to D.P.), the Fondation pour la Recherche Médicale (SPF201909009274 to C.G.) and grants from Institut Cochin (PIC-2018 to L.E.K.), "Ministerio de Economía y competitividad" FI17/00224 to A.I., and "Ministerio de Ciencia e Innovación" PI20/00936 to R.O. and MV20/00026 to A.I. M.B and M.Co. received a PhD funding from Université Paris Cité, M.Cr., from University Grenoble Alps (UGA). The proteomic experiments were partially supported by Agence Nationale de

© 2023 The Authors

la Recherche under projects ProFI (Proteomics French Infrastructure, ANR-10-INBS-08) and GRAL, a program from the Chemistry Biology Health (CBH) Graduate School of University Grenoble Alpes (ANR-17-EURE-0003). This publication is also based upon work from COST Action CA20119 (ANDRONET) supported by European Cooperation in Science and Technology (www.cost.eu).

Author contributions

Melina Blanco: Formal analysis; methodology; writing – original draft. **Laila El Khattabi:** Conceptualization; formal analysis; writing – review and editing. **Clara Gobé:** Formal analysis; methodology. **Marion Crespo:** Formal analysis; visualization. **Manon Coulée:** Formal analysis; visualization. **Alberto de la Iglesia:** Formal analysis; methodology. **Côme Ialy-Radio:** Formal analysis. **Clementine Lapoujade:** Formal analysis. **Maëlle Givelet:** Formal analysis. **Marion Delessard:** Formal analysis. **Ivan Seller-Corona:** Formal analysis. **Kosuke Yamaguchi:** Resources; methodology. **Nadége Vernet:** Formal analysis. **Fred Van Leeuwen:** Resources. **Alban Lermine:** Resources. **Yuki Okada:** Resources; methodology. **Romain Daveau:** Resources; formal analysis. **Rafael Oliva:** Resources. **Pierre Fouchet:** Conceptualization; formal analysis. **Ahmed Ziyyat:** Conceptualization; formal analysis. **Delphine Pflieger:** Conceptualization; formal analysis; funding acquisition; methodology; writing – review and editing. **Julie Cocquet:** Conceptualization; formal analysis; supervision; funding acquisition; investigation; writing – original draft; project administration; writing – review and editing.

Disclosure and competing interests statement

The authors declare that they have no conflict of interest.

References

Aguilar D, Strom J, Chen QM (2014) Glucocorticoid induced leucine zipper inhibits apoptosis of cardiomyocytes by doxorubicin. *Toxicol Appl Pharmacol* 276: 55 – 62

Ahmed EA, de Rooij DG (2009) Staging of mouse seminiferous tubule cross-sections. *Methods Mol Biol* 558: 263 – 277

Arevalo L, Merges GE, Schneider S, Oben FE, Neumann IS, Schorle H (2022) Loss of the cleaved-protamine 2 domain leads to incomplete histone-to-protamine exchange and infertility in mice. *PLoS Genet* 18: e1010272

Aslam MA, Alemdehy MF, Kwesi-Maliepaard EM, Muhaimin FI, Caganova M, Pardieck IN, van den Brand T, van Welsem T, de Rink I, Song JY *et al* (2021) Histone methyltransferase DOT1L controls state-specific identity during B cell differentiation. *EMBO Rep* 22: e51184

Bao J, Bedford MT (2016) Epigenetic regulation of the histone-to-protamine transition during spermiogenesis. *Reproduction* 151: R55 – R70

Bao J, Ma HY, Schuster A, Lin YM, Yan W (2013) Incomplete cre-mediated excision leads to phenotypic differences between Stra8-iCre; Mov10l1(lox/lox) and Stra8-iCre; Mov10l1(lox/Delta) mice. *Genesis* 51: 481 – 490

Barral S, Morozumi Y, Tanaka H, Montellier E, Govin J, de Dieuleveult M, Charbonnier G, Coute Y, Puthier D, Buchou T *et al* (2017) Histone variant H2A.L.2 guides transition protein-dependent protamine assembly in male germ cells. *Mol Cell* 66: 89 – 101.e8

Barraud-Lange V, Ialy-Radio C, Chalas C, Holtzmann I, Wolf JP, Barbaux S, Ziyyat A (2020) Partial sperm beta1 integrin subunit deletion proves its involvement in mouse gamete adhesion/fusion. *Int J Mol Sci* 21: 8494

Benjamini Y, Hochberg Y (1995) Controlling the false discovery rate: a practical and powerful approach to multiple testing. *J R Stat Soc B Methodol* 57: 289 – 300

Boussouar F, Goudarzi A, Buchou T, Shiota H, Barral S, Debernardi A, Guardiola P, Brindle P, Martinez G, Arnoult C *et al* (2014) A specific CBP/p300-dependent gene expression programme drives the metabolic remodelling in late stages of spermatogenesis. *Andrology* 2: 351 – 359

Bouyssie D, Hesse AM, Mouton-Barbosa E, Rompais M, Macron C, Carapito C, Gonzalez de Peredo A, Coute Y, Dupierris V, Burel A *et al* (2020) Proline: an efficient and user-friendly software suite for large-scale proteomics. *Bioinformatics* 36: 3148 – 3155

Braun RE (2001) Packaging paternal chromosomes with protamine. *Nat Genet* 28: 10 – 12

Cattaneo P, Hayes MGB, Baumgarten N, Hecker D, Peruzzo S, Aslan GS, Kunderfranco P, Larcher V, Zhang L, Contu R *et al* (2022) DOT1L regulates chamber-specific transcriptional networks during cardiogenesis and mediates postnatal cell cycle withdrawal. *Nat Commun* 13: 7444

Chang CC, Ye BH, Chaganti RS, Dalla-Favera R (1996) BCL-6, a POZ/zinc-finger protein, is a sequence-specific transcriptional repressor. *Proc Natl Acad Sci USA* 93: 6947 – 6952

Chen Y, Zheng Y, Gao Y, Lin Z, Yang S, Wang T, Wang Q, Xie N, Hua R, Liu M *et al* (2018) Single-cell RNA-seq uncovers dynamic processes and critical regulators in mouse spermatogenesis. *Cell Res* 28: 879 – 896

Cocquet J, Ellis PJ, Yamauchi Y, Mahadevaiah SK, Affara NA, Ward MA, Burgoyne PS (2009) The multicopy gene sly represses the sex chromosomes in the male mouse germline after meiosis. *PLoS Biol* 7: e1000244

Comptour A, Moretti C, Serrentino ME, Auer J, Ialy-Radio C, Ward MA, Toure A, Vaiman D, Cocquet J (2014) SSTY proteins co-localize with the post-meiotic sex chromatin and interact with regulators of its expression. *FEBS J* 281: 1571 – 1584

Corbineau S, Lassalle B, Givelet M, Souissi-Sarahoui I, Firlej V, Romeo PH, Allemand I, Riou L, Fouchet P (2017) Spermatogonial stem cells and progenitors are refractory to reprogramming to pluripotency by the transcription factors Oct3/4, c-Myc, Sox2 and Klf4. *Oncotarget* 8: 10050 – 10063

Crespo M, Damont A, Blanco M, Lastrucci E, Kennani SE, Ialy-Radio C, Khattabi LE, Terrier S, Louwagie M, Kieffer-Jaquinod S *et al* (2020) Multi-omic analysis of gametogenesis reveals a novel signature at the promoters and distal enhancers of active genes. *Nucleic Acids Res* 48: 4115 – 4138

da Cruz I, Rodriguez-Casuriaga R, Santinaque FF, Farias J, Curti G, Capoano CA, Folle GA, Benavente R, Sotelo-Silveira JR, Geisinger A (2016) Transcriptome analysis of highly purified mouse spermatogenic cell populations: gene expression signatures switch from meiotic-to postmeiotic-related processes at pachytene stage. *BMC Genomics* 17: 294

Darde TA, Sallou O, Becker E, Evrard B, Monjeaud C, Le Bras Y, Jegou B, Collin O, Rolland AD, Chalmel F (2015) The ReproGenomics viewer: an integrative cross-species toolbox for the reproductive science community. *Nucleic Acids Res* 43: W109 – W116

Darde TA, Lecluze E, Lardenois A, Stevant I, Alary N, Tuttelmann F, Collin O, Nef S, Jegou B, Rolland AD *et al* (2019) The ReproGenomics viewer: a multi-omics and cross-species resource compatible with single-cell studies for the reproductive science community. *Bioinformatics* 35: 3133 – 3139

Dobin A, Gingeras TR (2015) Mapping RNA-seq reads with STAR. *Curr Protoc Bioinformatics* 51: 11.14.1 – 11.14.19

Dong Y, Isono KI, Ohbo K, Endo TA, Ohara O, Maekawa M, Toyama Y, Ito C, Toshimori K, Helin K *et al* (2017) EPC1/TIP60-mediated histone acetylation facilitates spermiogenesis in mice. *Mol Cell Biol* 37: e00082-17

Dottermusch-Heidel C, Gartner SM, Tegeder I, Rathke C, Barckmann B, Bartkuhn M, Bhushan S, Steger K, Meinhardt A, Renkawitz-Pohl R (2014a)

 © 2023 The Authors

H3K79 methylation: a new conserved mark that accompanies H4 hyperacetylation prior to histone-to-protamine transition in drosophila and rat. *Biol Open* 3: 444–452

Dottermusch-Heidel C, Klaus ES, Gonzalez NH, Bhushan S, Meinhardt A, Bergmann M, Renkawitz-Pohl R, Rathke C, Steger K (2014b) H3K79 methylation directly precedes the histone-to-protamine transition in mammalian spermatids and is sensitive to bacterial infections. *Andrology* 2: 655–665

Durinck S, Moreau Y, Kasprzyk A, Davis S, De Moor B, Brazma A, Huber W (2005) BioMart and Bioconductor: a powerful link between biological databases and microarray data analysis. *Bioinformatics* 21: 3439–3440

El Kennani S, Adrait A, Shaytan AK, Khochbin S, Bruley C, Panchenko AR, Landsman D, Pflieger D, Govin J (2017) MS_HistoneDB, a manually curated resource for proteomic analysis of human and mouse histones. *Epigenetics Chromatin* 10: 2

Ernst C, Eling N, Martinez-Jimenez CP, Marioni JC, Odom DT (2019) Staged developmental mapping and X chromosome transcriptional dynamics during mouse spermatogenesis. *Nat Commun* 10: 1251

Evertts AG, Manning AL, Wang X, Dyson NJ, Garcia BA, Coller HA (2013) H4K20 methylation regulates quiescence and chromatin compaction. *Mol Biol Cell* 24: 3025–3037

Frehlick LJ, Eirin-Lopez JM, Ausio J (2007) New insights into the nucleophosmin/nucleoplasmin family of nuclear chaperones. *Bioessays* 29: 49–59

Fujihara Y, Murakami M, Inoue N, Satouh Y, Kaseda K, Ikawa M, Okabe M (2010) Sperm equatorial segment protein 1, SPESP1, is required for fully fertile sperm in mouse. *J Cell Sci* 123: 1531–1536

Gadadhar S, Alvarez Viar G, Hansen JN, Gong A, Kostarev A, Ialy-Radio C, Leboucher S, Whitfield M, Ziyyat A, Toure A *et al* (2021) Tubulin glycylation controls axonemal dynein activity, flagellar beat, and male fertility. *Science* 371: eabd4914

Gaucher J, Boussouar F, Montellier E, Curtet S, Buchou T, Bertrand S, Hery P, Jounier S, Depaux A, Vitte AL *et al* (2012) Bromodomain-dependent stage-specific male genome programming by Brdt. *EMBO J* 31: 3809–3820

Gilan O, Lam EY, Becher I, Lugo D, Cannizzaro E, Joberty G, Ward A, Wiese M, Fong CY, Ftouni S *et al* (2016) Functional interdependence of BRD4 and DOT1L in MLL leukemia. *Nat Struct Mol Biol* 23: 673–681

Goudarzi A, Zhang D, Huang H, Barral S, Kwon OK, Qi S, Tang Z, Buchou T, Vitte AL, He T *et al* (2016) Dynamic competing histone H4 K5K8 acetylation and Butyrylation are hallmarks of highly active gene promoters. *Mol Cell* 62: 169–180

Green CD, Ma Q, Manske GL, Shami AN, Zheng X, Marini S, Moritz L, Sultan C, Gurczynski SJ, Moore BB *et al* (2018) A comprehensive roadmap of murine spermatogenesis defined by single-cell RNA-seq. *Dev Cell* 46: 651–667.e10

Heinz S, Benner C, Spann N, Bertolino E, Lin YC, Laslo P, Cheng JX, Murre C, Singh H, Glass CK (2010) Simple combinations of lineage-determining transcription factors prime cis-regulatory elements required for macrophage and B cell identities. *Mol Cell* 38: 576–589

Ho CH, Takizawa Y, Kobayashi W, Arimura Y, Kimura H, Kurumizaka H (2021) Structural basis of nucleosomal histone H4 lysine 20 methylation by SET8 methyltransferase. *Life Sci Alliance* 4: e202000919

Jo SY, Granowicz EM, Maillard I, Thomas D, Hess JL (2011) Requirement for Dot1l in murine postnatal hematopoiesis and leukemogenesis by MLL translocation. *Blood* 117: 4759–4768

Jones B, Su H, Bhat A, Lei H, Bajko J, Hevi S, Baltus GA, Kadam S, Zhai H, Valdez R *et al* (2008) The histone H3K79 methyltransferase Dot1L is essential for mammalian development and heterochromatin structure. *PLoS Genet* 4: e1000190

Kawano N, Kang W, Yamashita M, Koga Y, Yamazaki T, Hata T, Miyado K, Baba T (2010) Mice lacking two sperm serine proteases, ACR and PRSS21, are subfertile, but the mutant sperm are infertile in vitro. *Biol Reprod* 83: 359–369

Kim W, Choi M, Kim JE (2014) The histone methyltransferase Dot1/DOT1L as a critical regulator of the cell cycle. *Cell Cycle* 13: 726–738

Koster J, Rahmann S (2018) Snakemake-a scalable bioinformatics workflow engine. *Bioinformatics* 34: 3600

Kuang W, Zhang J, Lan Z, Deepak R, Liu C, Ma Z, Cheng L, Zhao X, Meng X, Wang W *et al* (2021) SLC22A14 is a mitochondrial riboflavin transporter required for sperm oxidative phosphorylation and male fertility. *Cell Rep* 35: 109025

Kurosu T, Fukuda T, Miki T, Miura O (2003) BCL6 overexpression prevents increase in reactive oxygen species and inhibits apoptosis induced by chemotherapeutic reagents in B-cell lymphoma cells. *Oncogene* 22: 4459–4468

Kwesi-Maliepaard EM, Aslam MA, Alemdehy MF, van den Brand T, McLean C, Vlaming H, van Welsem T, Korthout T, Lancini C, Hendriks S *et al* (2020) The histone methyltransferase DOT1L prevents antigen-independent differentiation and safeguards epigenetic identity of CD8(+) T cells. *Proc Natl Acad Sci USA* 117: 20706–20716

Langmead B, Salzberg SL (2012) Fast gapped-read alignment with bowtie 2. *Nat Methods* 9: 357–359

Lin YH, Kakadia PM, Chen Y, Li YQ, Deshpande AJ, Buske C, Zhang KL, Zhang Y, Xu GL, Bohlander SK (2009) Global reduction of the epigenetic H3K79 methylation mark and increased chromosomal instability in CALM-AF10-positive leukemias. *Blood* 114: 651–658

Lin H, Cheng K, Kubota H, Lan Y, Riedel SS, Kakiuchi K, Sasaki K, Bernt KM, Bartolomei MS, Luo M *et al* (2022) Histone methyltransferase DOT1L is essential for self-renewal of germline stem cells. *Genes Dev* 36: 752–763

Love MI, Huber W, Anders S (2014) Moderated estimation of fold change and dispersion for RNA-seq data with DESeq2. *Genome Biol* 15: 550

Luense LJ, Wang X, Schon SB, Weller AH, Lin Shiao E, Bryant JM, Bartolomei MS, Coutifaris C, Garcia BA, Berger SL (2016) Comprehensive analysis of histone post-translational modifications in mouse and human male germ cells. *Epigenetics Chromatin* 9: 24

Luense LJ, Donahue G, Lin-Shiao E, Rangel R, Weller AH, Bartolomei MS, Berger SL (2019) Gcn5-mediated histone acetylation governs nucleosome dynamics in spermiogenesis. *Dev Cell* 51: 745–758.e6

Maruyama SY, Ito M, Ikami Y, Okitsu Y, Ito C, Toshimori K, Fujii W, Yogo K (2016) A critical role of solute carrier 22a14 in sperm motility and male fertility in mice. *Sci Rep* 6: 36468

de Mateo S, Gazquez C, Guimera M, Balasch J, Meistrich ML, Ballesca JL, Oliva R (2009) Protamine 2 precursors (pre-P2), protamine 1 to protamine 2 ratio (P1/P2), and assisted reproduction outcome. *Fertil Steril* 91: 715–722

McCarthy DJ, Smyth GK (2009) Testing significance relative to a fold-change threshold is a TREAT. *Bioinformatics* 25: 765–771

Montellier E, Rousseaux S, Zhao Y, Khochbin S (2011) Histone crotonylation specifically marks the haploid male germ cell gene expression program: post-meiotic male-specific gene expression. *Bioessays* 34: 187–193

Montellier E, Boussouar F, Rousseaux S, Zhang K, Buchou T, Fenaille F, Shiota H, Debernardi A, Hery P, Curtet S *et al* (2013) Chromatin-to-nucleoprotamine transition is controlled by the histone H2B variant TH2B. *Genes Dev* 27: 1680–1692

Moretti C, Serrentino ME, Ialy-Radio C, Delessard M, Soboleva TA, Tores F, Leduc M, Nitschke P, Drevet JR, Tremethick DJ *et al* (2017) SLY regulates genes involved in chromatin remodeling and interacts with TBL1XR1 during sperm differentiation. *Cell Death Differ* 24: 1029–1044

Muzumdar MD, Tasic B, Miyamichi K, Li L, Luo L (2007) A global double-fluorescent Cre reporter mouse. *Genesis* 45: 593–605

Nguyen AT, Taranova O, He J, Zhang Y (2011) DOT1L, the H3K79 methyltransferase, is required for MLL-AF9-mediated leukemogenesis. *Blood* 117: 6912 – 6922

Okada Y, Feng Q, Lin Y, Jiang Q, Li Y, Coffield VM, Su L, Xu G, Zhang Y (2005) hDOT1L links histone methylation to leukemogenesis. *Cell* 121: 167 – 178

Oliva R (2006) Protamines and male infertility. *Hum Reprod Update* 12: 417 – 435

Oliva R, Bazett-Jones D, Mezquita C, Dixon GH (1987) Factors affecting nucleosome disassembly by protamines in vitro. Histone hyperacetylation and chromatin structure, time dependence, and the size of the sperm nuclear proteins. *J Biol Chem* 262: 17016 – 17025

Onder TT, Kara N, Cherry A, Sinha AU, Zhu N, Bernt KM, Cahan P, Marcarci BO, Unternaehrer J, Gupta PB *et al* (2012) Chromatin-modifying enzymes as modulators of reprogramming. *Nature* 483: 598 – 602

Perez-Riverol Y, Csordas A, Bai J, Bernal-Llinares M, Hewapathirana S, Kundu DJ, Inuganti A, Griss J, Mayer G, Eisenacher M *et al* (2019) The PRIDE database and related tools and resources in 2019: improving support for quantification data. *Nucleic Acids Res* 47: D442 – D450

Ragazzini R, Perez-Palacios R, Baymaz IH, Diop S, Ancelin K, Zielinski D, Michaud A, Givelet M, Borsos M, Aflaki S *et al* (2019) EZHIP constrains Polycomb repressive complex 2 activity in germ cells. *Nat Commun* 10: 3858

Rathke C, Baarends WM, Awe S, Renkawitz-Pohl R (2014) Chromatin dynamics during spermiogenesis. *Biochim Biophys Acta* 1839: 155 – 168

Robinson MD, McCarthy DJ, Smyth GK (2010) edgeR: a Bioconductor package for differential expression analysis of digital gene expression data. *Bioinformatics* 26: 139 – 140

Sadate-Ngatchou PI, Payne CJ, Dearth AT, Braun RE (2008) Cre recombinase activity specific to postnatal, premeiotic male germ cells in transgenic mice. *Genesis* 46: 738 – 742

Shang E, Nickerson HD, Wen D, Wang X, Wolgemuth DJ (2007) The first bromodomain of Brdt, a testis-specific member of the BET sub-family of double-bromodomain-containing proteins, is essential for male germ cell differentiation. *Development* 134: 3507 – 3515

Shiota H, Barral S, Buchou T, Tan M, Coute Y, Charbonnier G, Reynoird N, Boussouar F, Gerard M, Zhu M *et al* (2018) Nut directs p300-dependent, genome-wide H4 hyperacetylation in male germ cells. *Cell Rep* 24: 3477 – 3487.e6

Shoaib M, Chen Q, Shi X, Nair N, Prasanna C, Yang R, Walter D, Frederiksen KS, Einarsson H, Svensson JP *et al* (2021) Histone H4 lysine 20 mono-methylation directly facilitates chromatin openness and promotes transcription of housekeeping genes. *Nat Commun* 12: 4800

Soler-Ventura A, Castillo J, de la Iglesia A, Jodar M, Barrachina F, Ballesca JL, Oliva R (2018) Mammalian sperm protamine extraction and analysis: a step-by-step detailed protocol and brief review of protamine alterations. *Protein Pept Lett* 25: 424 – 433

Soumillon M, Necsulea A, Weier M, Brawand D, Zhang X, Gu H, Barthes P, Kokkinaki M, Nef S, Gnirke A *et al* (2013) Cellular source and mechanisms of high transcriptome complexity in the mammalian testis. *Cell Rep* 3: 2179 – 2190

Steger DJ, Lefterova MI, Ying L, Stonestrom AJ, Schupp M, Zhuo D, Vakoc AL, Kim JE, Chen J, Lazar MA *et al* (2008) DOT1L/KMT4 recruitment and H3K79 methylation are ubiquitously coupled with gene transcription in mammalian cells. *Mol Cell Biol* 28: 2825 – 2839

Subramanian A, Tamayo P, Mootha VK, Mukherjee S, Ebert BL, Gillette MA, Paulovich A, Pomeroy SL, Golub TR, Lander ES *et al* (2005) Gene set enrichment analysis: a knowledge-based approach for interpreting genome-wide expression profiles. *Proc Natl Acad Sci USA* 102: 15545 – 15550

Thomis DC, Lee W, Berg LJ (1997) T cells from Jak3-deficient mice have intact TCR signaling, but increased apoptosis. *J Immunol* 159: 4708 – 4719

Torregrosa N, Dominguez-Fandos D, Camejo MI, Shirley CR, Meistrich ML, Ballesca JL, Oliva R (2006) Protamine 2 precursors, protamine 1/protamine 2 ratio, DNA integrity and other sperm parameters in infertile patients. *Hum Reprod* 21: 2084 – 2089

Toure A (2019) Importance of SLC26 transmembrane anion exchangers in sperm post-testicular maturation and fertilization potential. *Front Cell Dev Biol* 7: 230

Valencia-Sanchez MI, De Ioannes P, Wang M, Truong DM, Lee R, Armache JP, Boeke JD, Armache KJ (2021) Regulation of the Dot1 histone H3K79 methyltransferase by histone H4K16 acetylation. *Science* 371: eabc6663

Vlaming H, van Leeuwen F (2016) The upstreams and downstreams of H3K79 methylation by DOT1L. *Chromosoma* 125: 593 – 605

Wang Z, Zang C, Rosenfeld JA, Schones DE, Barski A, Cuddapah S, Cui K, Roh TY, Peng W, Zhang MQ *et al* (2008) Combinatorial patterns of histone acetylations and methylations in the human genome. *Nat Genet* 40: 897 – 903

Wang Y, Iwamori T, Kaneko T, Iida H, Iwamori N (2021) Comparative distributions of RSBN1 and methylated histone H4 lysine 20 in the mouse spermatogenesis. *PLoS one* 16: e0253897

Ward WS, Coffey DS (1991) DNA packaging and organization in mammalian spermatozoa: comparison with somatic cells. *Biol Reprod* 44: 569 – 574

Yamaguchi K, Hada M, Fukuda Y, Inoue E, Makino Y, Katou Y, Shirahige K, Okada Y (2018) Re-evaluating the localization of sperm-retained histones revealed the modification-dependent accumulation in specific genome regions. *Cell Rep* 23: 3920 – 3932

Yamauchi Y, Riel JM, Stoytcheva Z, Burgoyne PS, Ward MA (2010) Deficiency in mouse Y chromosome long arm gene complement is associated with sperm DNA damage. *Genome Biol* 11: R66

Yu YE, Zhang Y, Unni E, Shirley CR, Deng JM, Russell LD, Weil MM, Behringer RR, Meistrich ML (2000) Abnormal spermatogenesis and reduced fertility in transition nuclear protein 1-deficient mice. *Proc Natl Acad Sci USA* 97: 4683 – 4688

Yu G, Wang LG, He QY (2015) ChIPseeker: an R/Bioconductor package for ChIP peak annotation, comparison and visualization. *Bioinformatics* 31: 2382 – 2383

Zhang W, Hayashizaki Y, Kone BC (2004) Structure and regulation of the mDot1 gene, a mouse histone H3 methyltransferase. *Biochem J* 377: 641 – 651

Zhang Y, Liu T, Meyer CA, Eeckhoute J, Johnson DS, Bernstein BE, Nusbaum C, Myers RM, Brown M, Li W *et al* (2008) Model-based analysis of ChIP-seq (MACS). *Genome Biol* 9: R137

Zhao M, Shirley CR, Hayashi S, Marcon L, Mohapatra B, Suganuma R, Behringer RR, Boissonneault G, Yanagimachi R, Meistrich ML (2004a) Transition nuclear proteins are required for normal chromatin condensation and functional sperm development. *Genesis* 38: 200 – 213

Zhao M, Shirley CR, Mounsey S, Meistrich ML (2004b) Nucleoprotein transitions during spermiogenesis in mice with transition nuclear protein Tnp1 and Tnp2 mutations. *Biol Reprod* 71: 1016 – 1025

Zhu B, Chen S, Wang H, Yin C, Han C, Peng C, Liu Z, Wan L, Zhang X, Zhang J *et al* (2018) The protective role of DOT1L in UV-induced melanomagenesis. *Nat Commun* 9: 259

License: This is an open access article under the terms of the Creative Commons Attribution License, which permits use, distribution and reproduction in any medium, provided the original work is properly cited.

Expanded View Figures

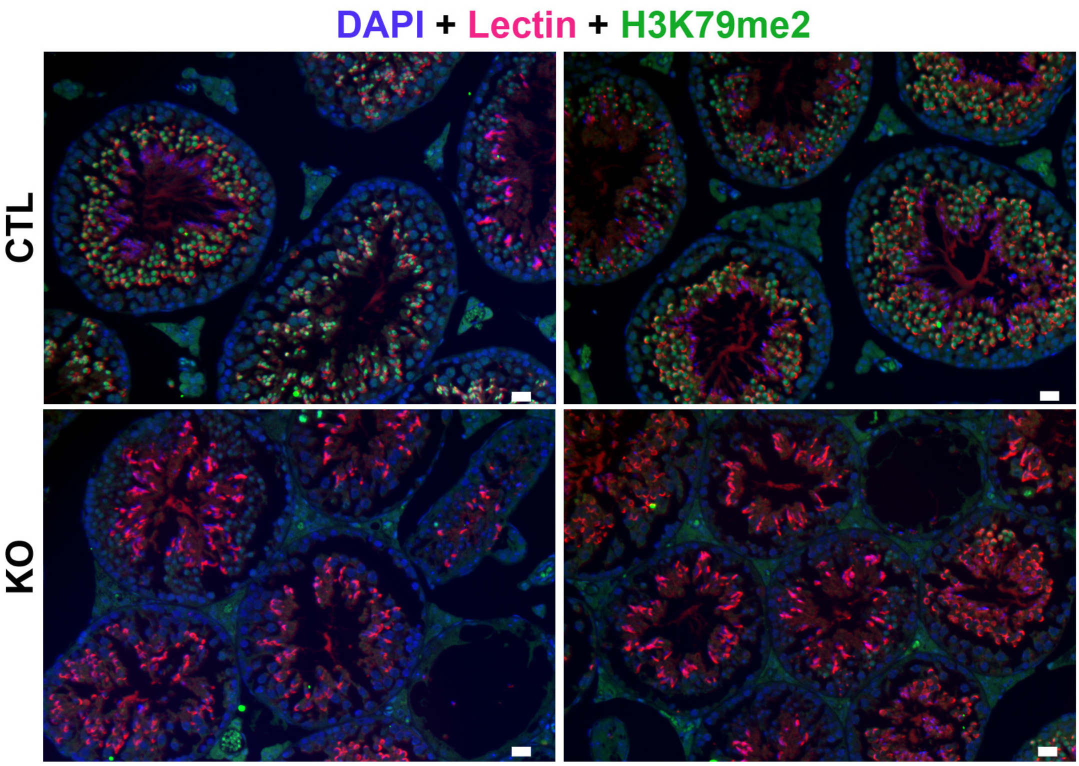

Figure EV1. Immunofluorescence detection of H3K79me2 on testicular sections from adult CTL or *Dot1l*-KO mice.

H3K79me2 signal is shown in green. DAPI (blue) was used to stain nuclei. Lectin (pink) was used to stain acrosome. Scale bar indicates 20 μm.

Figure EV2. Detailed analyses of CTL and *Dot1l*-KO spermatozoa.

A Scatter plots showing the quantification of sperm abnormalities (mean ± SEM; $N = 4$–5 and four biological replicates for CTL and KO, respectively). Stars indicate significant differences obtained using t-tests after angular transformation of the percentages (**P*-value < 0.05, ***P*-value < 0.005, and ****P*-value < 0.0005).

B Motility parameters from *Dot1l*-KO and CTL spermatozoa following CASA (computer-assisted sperm analyses) (mean ± SEM; $N = 12$ and 10 biological replicates for CTL and KO, respectively). Specifically, *Dot1l*-KO spermatozoa are slower and more static. They have a more linear movement (LIN) and a decreased straightness (STR), decreased lateral displacements of their heads (ALH) with a lower curvilinear velocity (VCL) resulting in a straight-line velocity (VSL) similar to CTL spermatozoa but a higher Average Path Velocity (VAP). Stars indicate significant differences obtained using Mann–Whitney *t*-tests after angular transformation of the percentages (***P*-value < 0.01, ****P*-value < 0.001, *****P*-value < 0.0001, ns' indicates non-significance). The Beat Cross Frequency (BCF) of CTL and *Dot1l* -KO spermatozoa is similar.

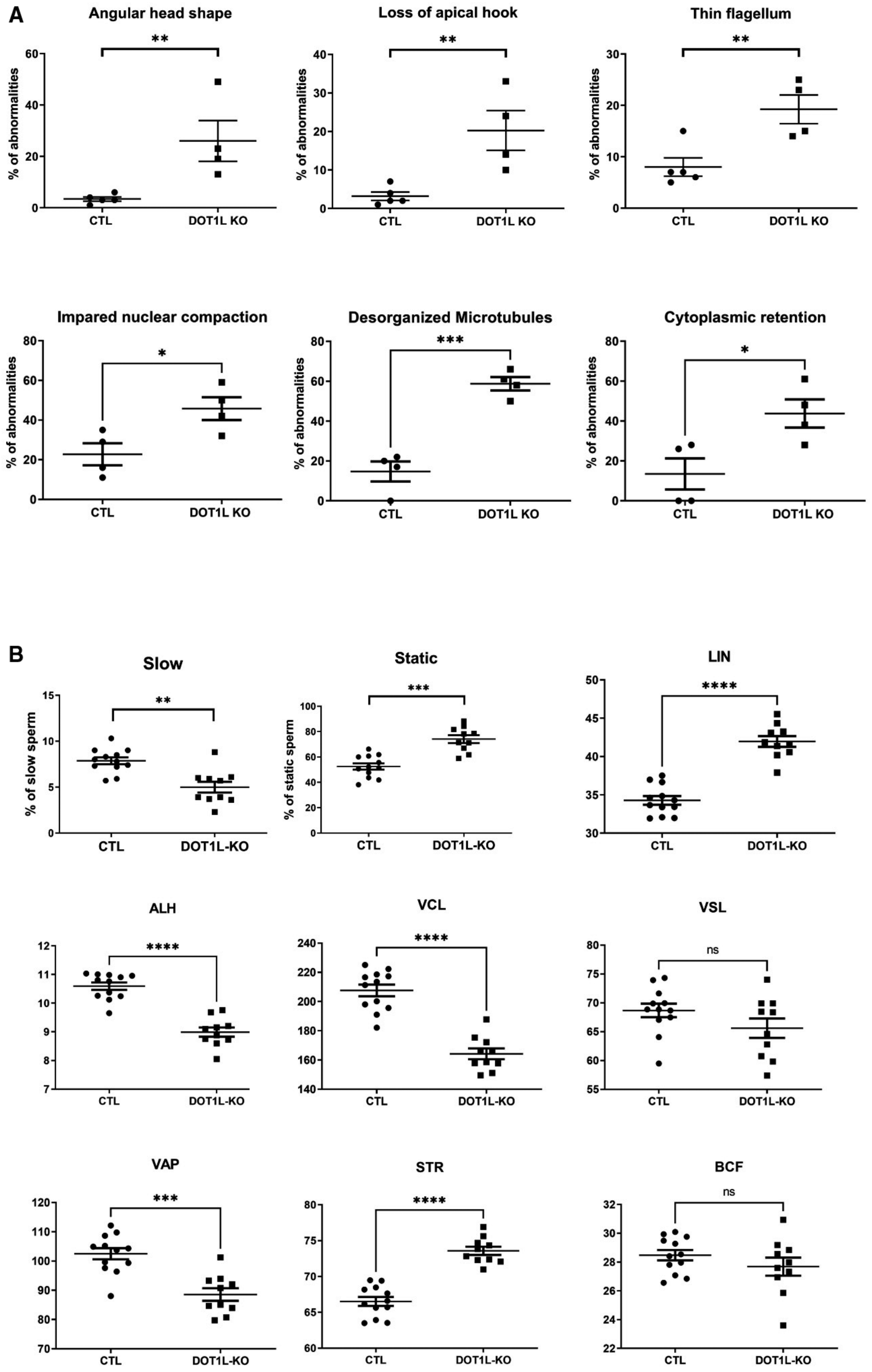

Figure EV2.

 © 2023 The Authors

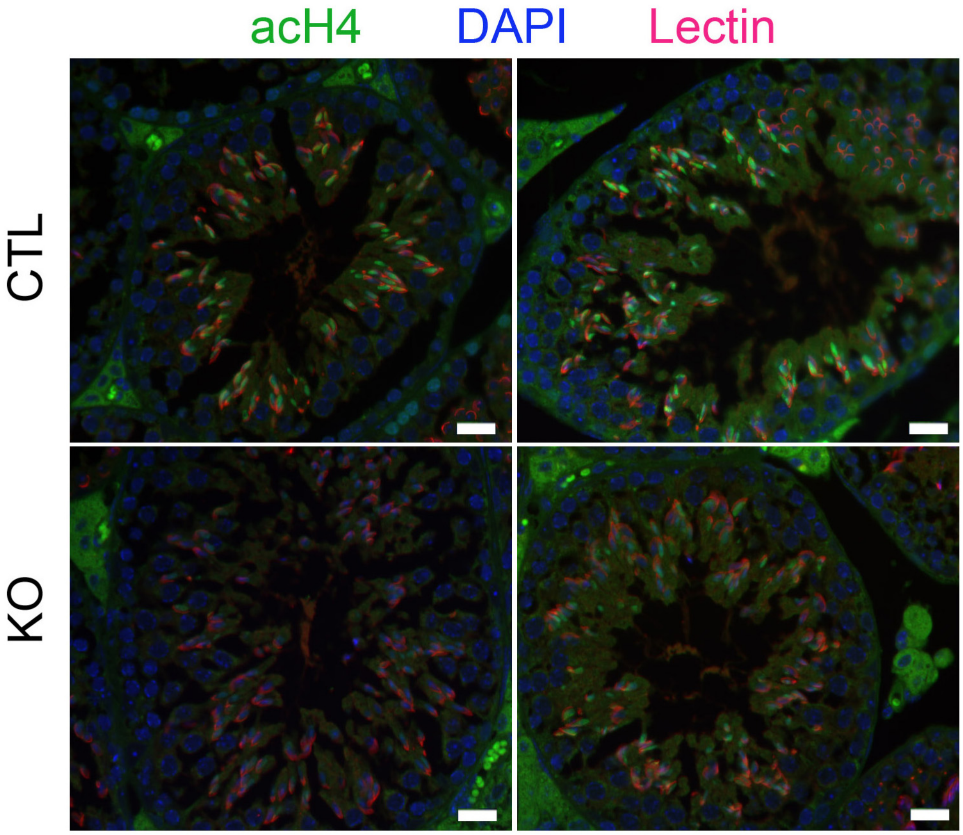

Figure EV3. Confirmation of a decrease in H4 acetylation in elongating spermatids (ES) by immunofluorescence.

Representative pictures of immunofluorescence detection of acH4 (anti-poly-H4ac, green) on stage XI testicular sections. At this stage, acH4 level is strong in CTL elongating spermatids but appears weaker in *Dot1l*-KO. DAPI (blue) was used to stain nuclei and lectin (red) to stage the acrosome and facilitates tubule staging. Scale bar indicates 20 μm.

© 2023 The Authors

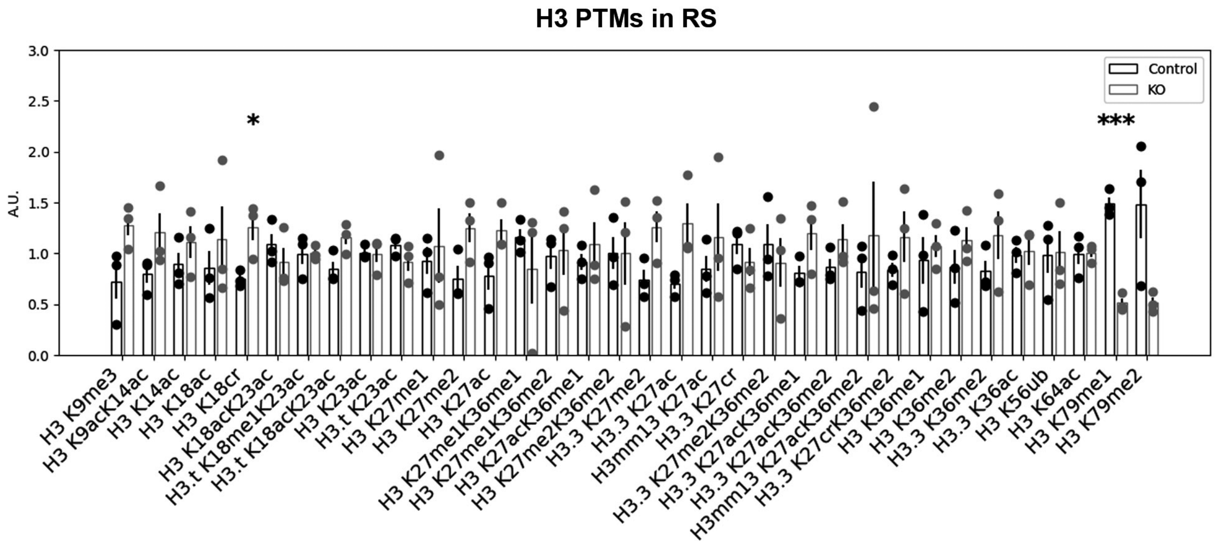

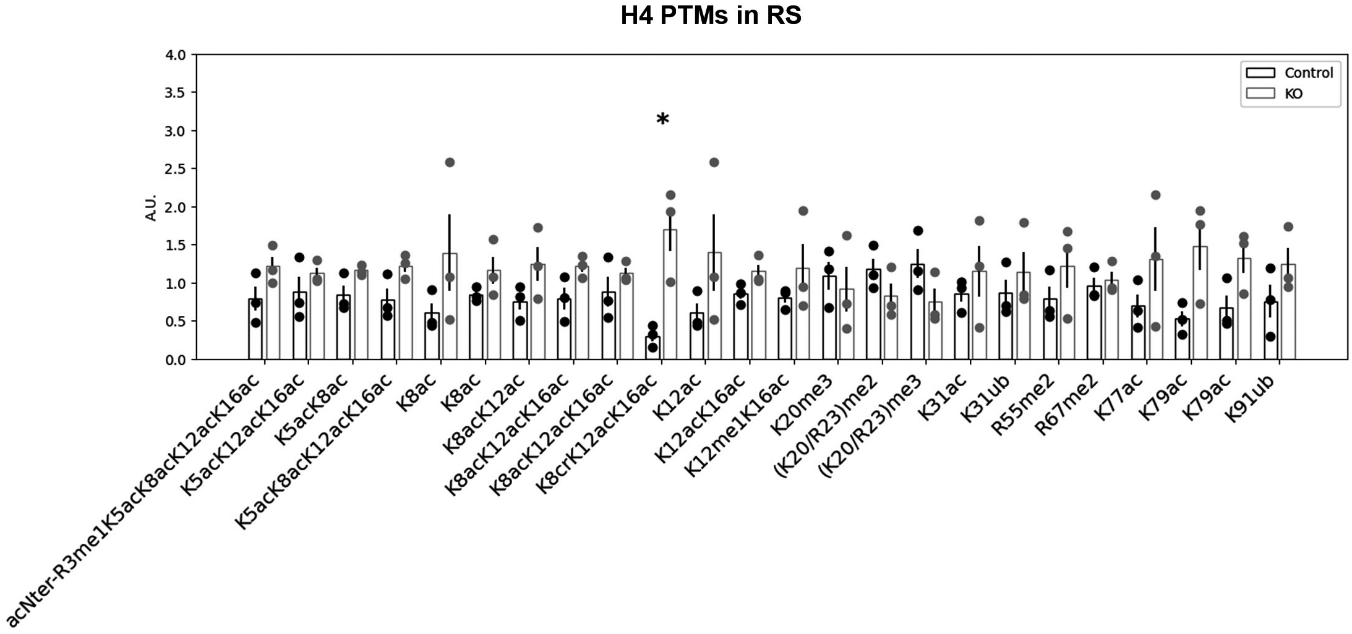

Figure EV4. Quantification of histone H3 and H4 PTMs in *Dot1l*-KO and CTL round spermatids.

LC–MS/MS data acquired on H3 and H4 were processed as described in Fig 4. Quantitative data obtained on biological replicates ($N = 3$ for CTL and 3 for KO) were plotted as dots, and the height of the bars indicates the average value (mean ± SD). A.U. = arbitrary units. *P-value < 0.05, **P-value < 0.005, and ***P-value < 0.0005, obtained with t-tests.

 © 2023 The Authors

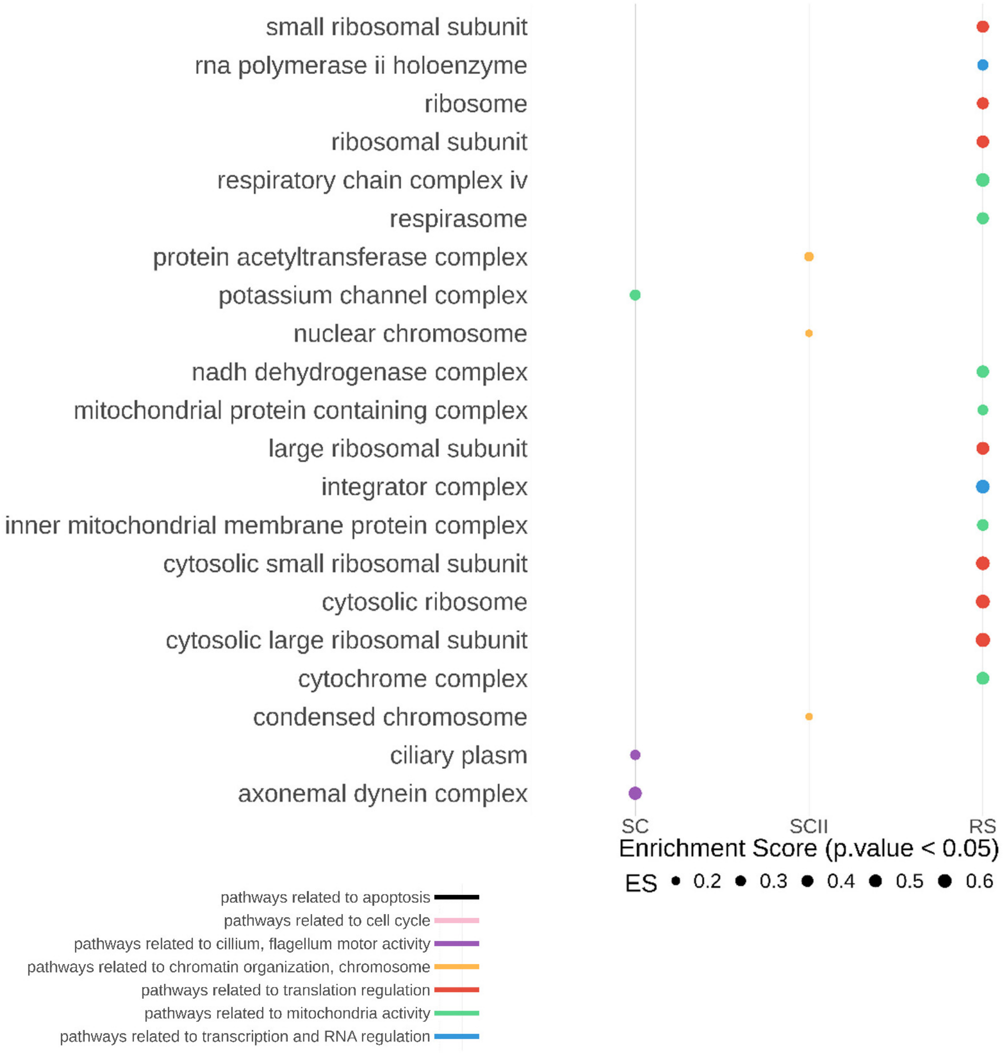

Figure EV5. GSEA analysis of RNA-seq data from *Dot1l*-KO versus CTL SC, SCII, and RS.

The figure shows all the "cellular components" found significantly downregulated in *Dot1l*-KO primary spermatocytes (SC), secondary spermatocytes (SCII) and round spermatids ($P < 0.05$), ranked by their enrichment score (ES).

© 2023 The Authors

Table of contents

Appendix Figure S1 2
Appendix Figure S2 8
Appendix Figure S3 10
Appendix Figure S4 13
Appendix Figure S5 18
Appendix Table S1 21
Appendix Table S2 22
References 23

Appendix Figure S1

1A

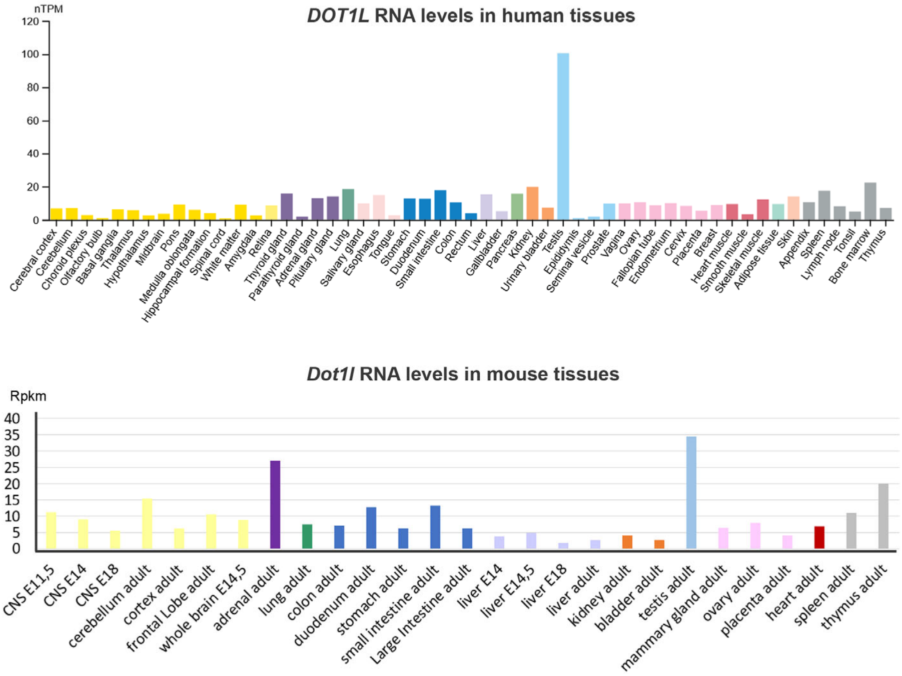

1B

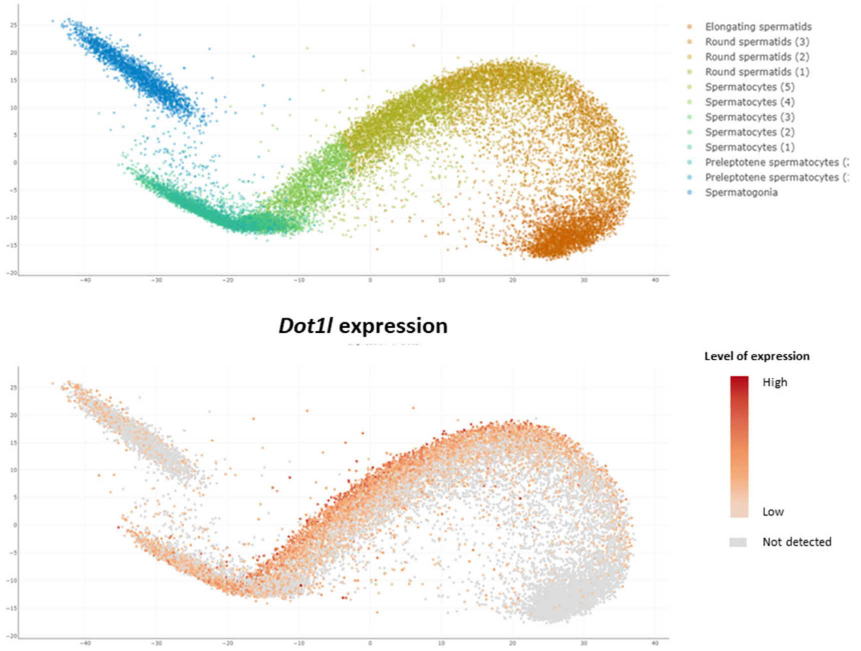

1C

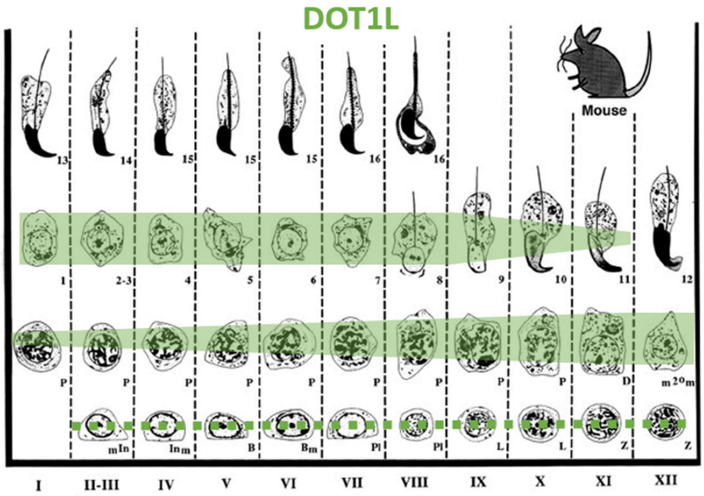
DOT1L
Mouse
I
II-III
IV
V
VI
VII
VIII
IX
X
XI
XII

1D

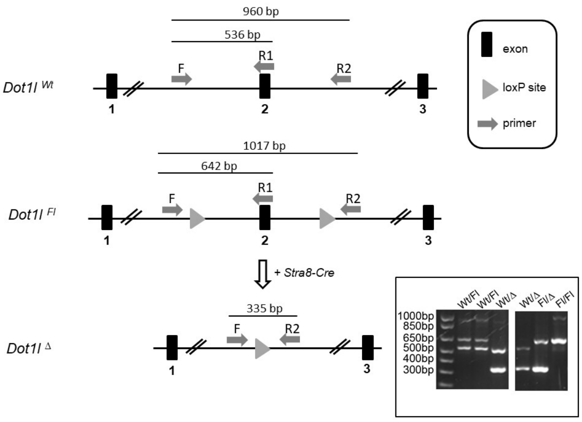
960 bp
536 bp
F
R1
R2
Dot1l Wt
1
2
3
exon
loxP site
primer
1017 bp
642 bp
Dot1l Fl
+ Stra8-Cre
335 bp
Dot1l Δ
1000bp
850bp
650bp
500bp
400bp
300bp
Wt/Fl
Wt/Fl
Wt/Δ
Wt/Δ
Fl/Δ
Fl/Fl

1E

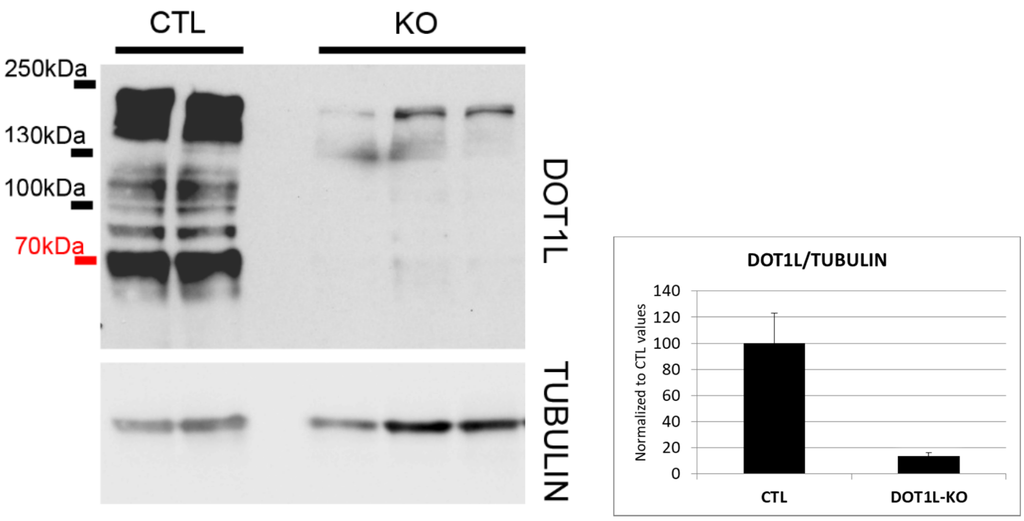
CTL
KO
250kDa
130kDa
100kDa
70kDa
DOT1L
TUBULIN
DOT1L/TUBULIN
Normalized to CTL values
140
120
100
80
60
40
20
0
CTL
DOT1L-KO

1F

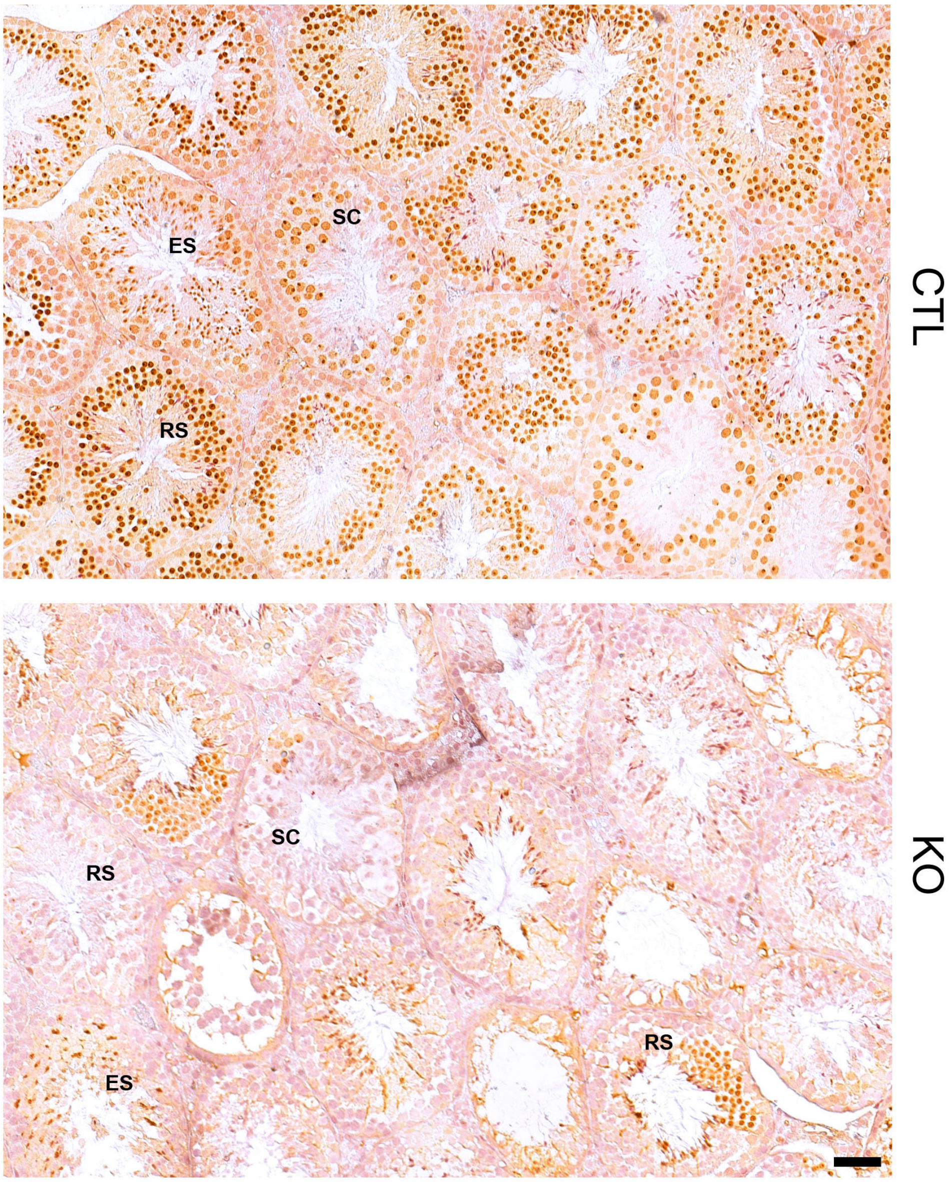

1G

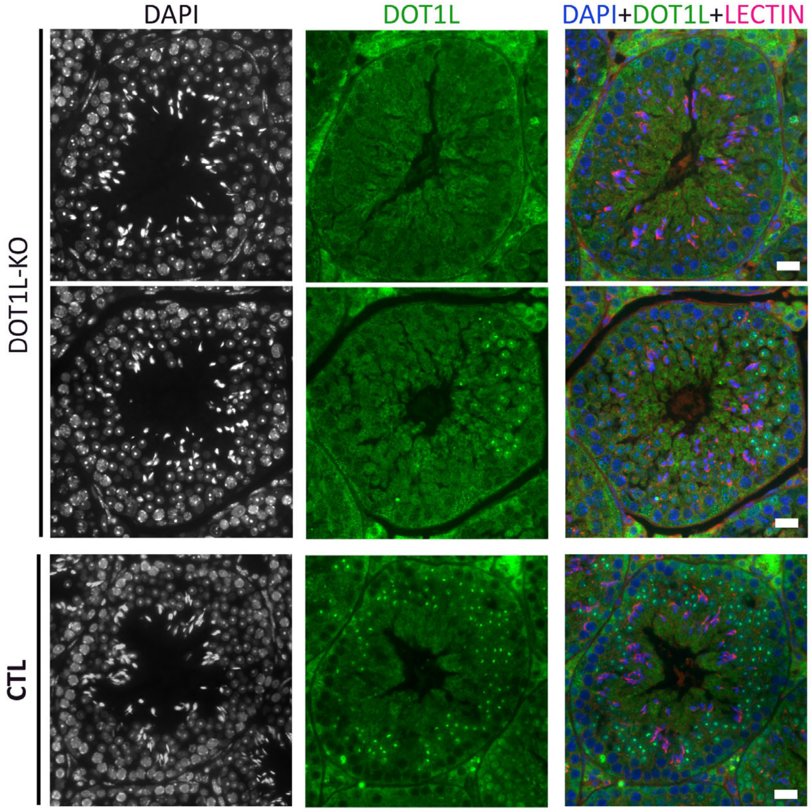
DAPI
DOT1L
DAPI+DOT1L+LECTIN
DOT1L-KO
CTL

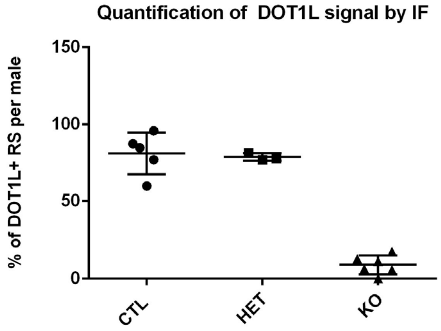
Quantification of DOT1L signal by IF
% of DOT1L+ RS per male
150
100
50
0
CTL
HET
KO

1H

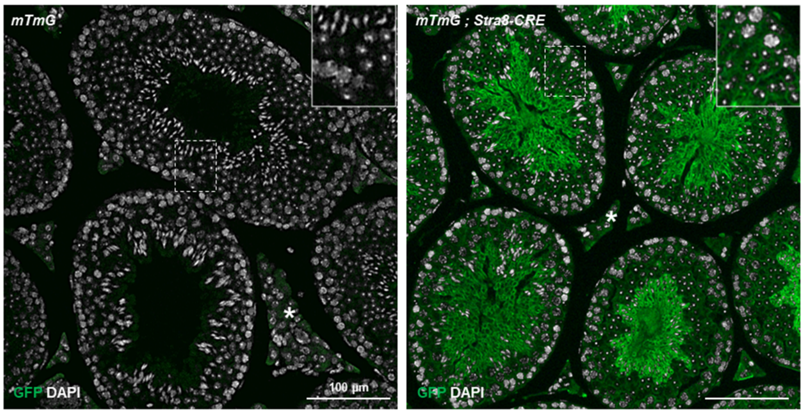
mTmG
mTmG ; Stra8-CRE
GFP DAPI
100 µm
GFP DAPI

1I

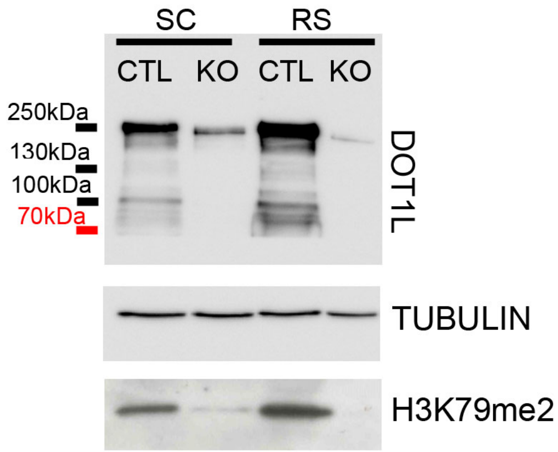

Appendix Figure S1. Complement to Figure 1. A) RNA expression overview of *DOT1L* RNA in human and mouse tissues. Data were obtained from The Human Protein Atlas [https://www.proteinatlas.org/; consensus dataset, consisting of normalized expression (nTPM) levels for 55 tissue types, created by combining the HPA and GTEx transcriptomics datasets] or from mouse tissues from mouse ENCODE transcriptome data (Data ref: Mouse ENCODE transcriptome data, NCBI Sequence Read Archive PRJNA66167, 2011). **B) Expression of *Dot1l* mRNA based on single cell RNA-seq analyses from adult testicular cells** (Data ref: (Green *et al*, 2018) analyzed using https://rgv.genouest.org/ (Darde *et al*, 2019; Darde *et al*, 2015)). **C) Summary of DOT1L protein expression in mouse germ cells based on immunofluorescence detection on testicular sections. Scheme modified after Russell et al. (Russell *et al*, 1990).** DOT1L protein is detected in all germ cells but is particularly abundant in late spermatocytes and in round spermatids. **D) Generation of *Dot1l* KO allele.** Drawing representing the beginning of mouse *Dot1l* gene (from exon 1 to exon 3), the location of loxP sites and genotyping primers. Bottom right insert shows typical pictures of products from PCR using *Dot1l* genotyping primers (F+R1+R2) following run on a 2% agarose gel. **E) Estimation of *Dot1l* knockout efficiency by western blot (uncropped western blot image of Figure 1B).** Detection of DOT1L and TUBULIN in whole testicular protein extracts from CTL and *Dot1l*-KO mice. The most abundant and high molecular weight bands are DOT1L testis-specific (~185KDa) and canonical proteoform. Additional bands most likely corresponding to smaller, less abundant proteoforms (such as Q679P5 at ~122KDa or Q6XZL7 at ~68KDa) are also visible in case of long exposure. All DOT1L proteoforms are markedly reduced in *Dot1l*-KO testicular extracts. On the right is shown a graphic representation of the quantification of DOT1L signal (all bands) normalized to TUBULIN signal**. F) Immunohistochemistry detection of DOT1L in testicular sections from CTL and *Dot1l*-KO (KO) adult mice counterstained with hematoxylin (complement panel to Fig 1C).** SC = Spermatocytes, RS = Round spermatids, ES = Elongating spermatids. Pictures were taken using the same parameters. Scale bars indicate 50 µm. **G) Estimation of *Dot1l* knockout efficiency by immunofluorescence.** Representative pictures of immunofluorescence detection of DOT1L (Abcam 64077 antibody, in green) on testicular sections from adult CTL or KO mice. DAPI (grey or blue) was used to stain nuclei; Lectin (pink) was used to stain acrosome. Scale bar indicates 20µm. In CTL testicular sections, tubules containing round spermatids are uniformly stained (bottom panel). In *Dot1l*-KO sections, the majority of tubules do not show any DOTL1 protein signal (top panel) but in some tubules, DOT1L signal can still be visible (middle panel). Below the immunofluorescence pictures is shown a graphic representation of the percentage of round spermatids with a visible DOT1L signal. N=5 CTL, 3 HET and 6 KO males between 2.5-4 month old (6 to 10 tubules per individual). Quantification was performed on stages I to V tubules. **H) Verification of *Stra8-Cre* recombinase activity and specificity in adult mouse testis.** The Cre reporter mouse line *mTmG* carries a *GFP* transgene expressed upon Cre induction. In this transgene, GFP is targeted to the cell membrane (Muzumdar *et al*, 2007). In testicular sections from *mTmG* mice (left panel), using an anti-GFP antibody, no signal is detected, excepted for faint auto-fluorescence from Leydig cells (asterisk). In *mTmG ; Stra8-Cre* mice (right panel) the Cre recombinase is specifically expressed in germ cells and GFP (in green) is detected in most germ

cells. DAPI (grey) has been used to stain cell nuclei. Top right panel represents a 2X magnification. **I) Uncropped western blot image of Fig 1D.** Note that the membrane was cut at ~70KDa and ~35KDa to generate three pieces which were incubated with anti-DOT1L antibody (top part), with anti-TUBULIN antibody (mid part) or with anti-H3K79me2 antibody (bottom part).

Appendix Figure S2

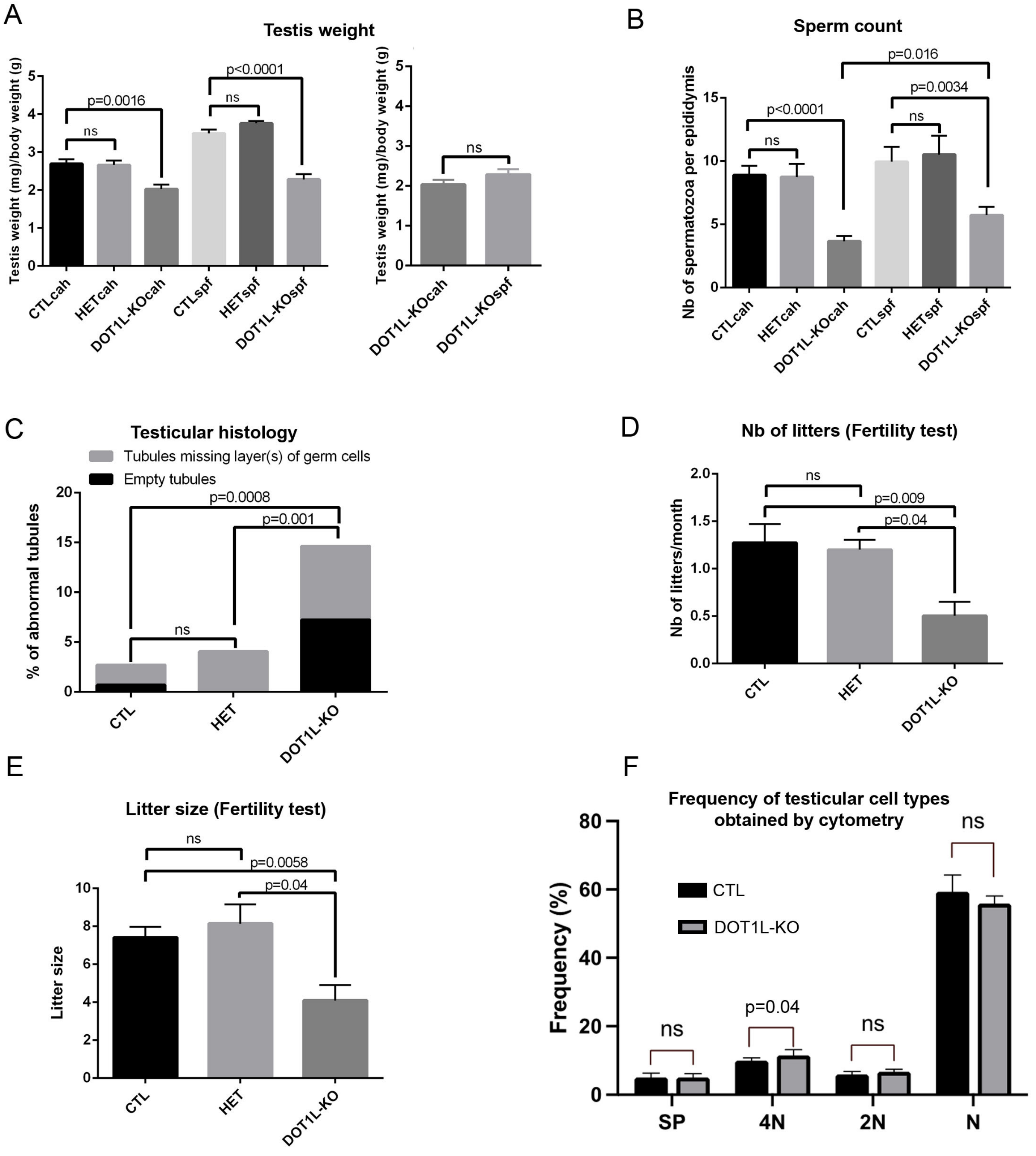

G

In vitro fertilization

	% of oocytes in vitro fertilized (nb of fertilized/total)
WT 1	52.4 (11/21)
WT 2	34.8 (24/69)
CTL 1	36.7 (18/49)
CTL 2	21.9 (14/64)
HET 1	37.5 (9/24)
DOT1L-KO 1	0 (0/37)
DOT1L-KO 2	0 (0/29)
DOT1L-KO 3	0 (0/27)
DOT1L-KO 4	0 (0/87)

Appendix Figure S2. Complement to Figure 2. Detailed analyses of reproductive parameters from *Dot1l*-KO males and CTL. 'cah' indicates animals produced in a conventional animal house. 'spf' indicates animals produced in the specific pathogen-free animal house. HET are heterozygous animals of the following genotype *Dot1l*$^{Fl/\Delta}$. Obtained p-values (following unpaired t-tests) are indicated above; "ns" indicates non-significant difference. **A**). Average testis weight (normalized to body weight, mean value ± SEM) in different groups of males (N=9 for CTLcah, N=6 for HETcah, N= 9 for DOT1Lcah, N=12 for CTLspf, N=2 for HETspf and N= 17 for DOT1Lspf). **B**) Average number of epididymal spermatozoa (mean value ± SEM) in different groups of males (N=12 for CTLcah, N=6 for HETcah, N=12 for DOT1Lcah, N=9 for CTLspf, N=2 for HETspf and N= 13 for DOT1Lspf). **C**) Graphic representation of the percentage of abnormal tubules in *Dot1l*-KO (N=5) CTL (N=6) and HET (N=4) testes (from both cah and spf). Obtained p-values (following unpaired t-tests on empty tubules values) are indicated above. **D**) and **E**) Results from the tests of fertility (natural mating in spf with wild type females for ~3 months) of *Dot1l*-KO, HET and CTL males (mean ± SEM; N=7 for KO, N= 2 for HET and N=6 for CTL). **F**) Schematic diagram representing the frequency of each cell type in CTL and *Dot1l*-KO testes, as calculated by FACS (mean ± SEM; N= for CTL and 3 for KO). 4N = Primary spermatocytes, 2N = Secondary spermatocytes, N = spermatids, SP = side population representing premeiotic germ cells (i.e. spermatogonia). **G)** Results from *in vitro* fertilization assays. The percentage of fertilized oocytes is indicated for each male. Values obtained for *Dot1l*-KO spermatozoa are significantly different from WT or CTL spermatozoa (chi-square, p<0.01).

Appendix Figure S3

3A **3B**

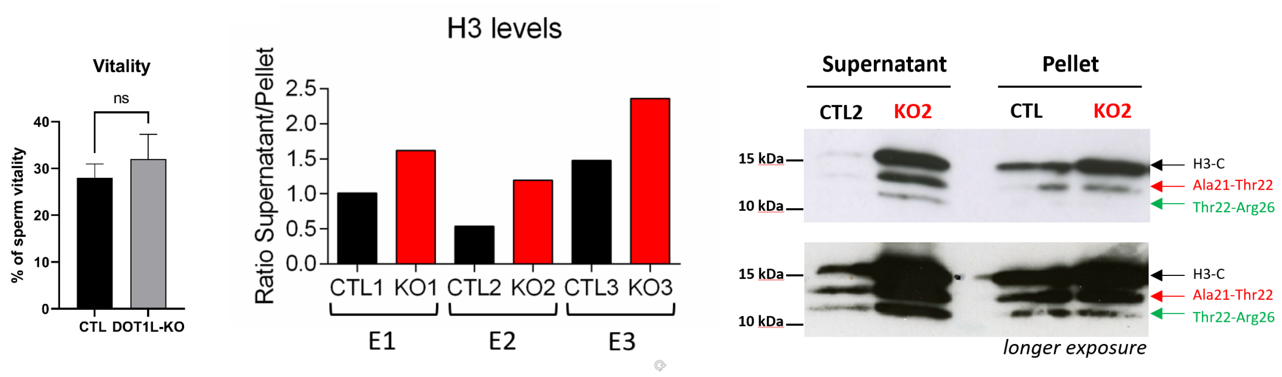

3C

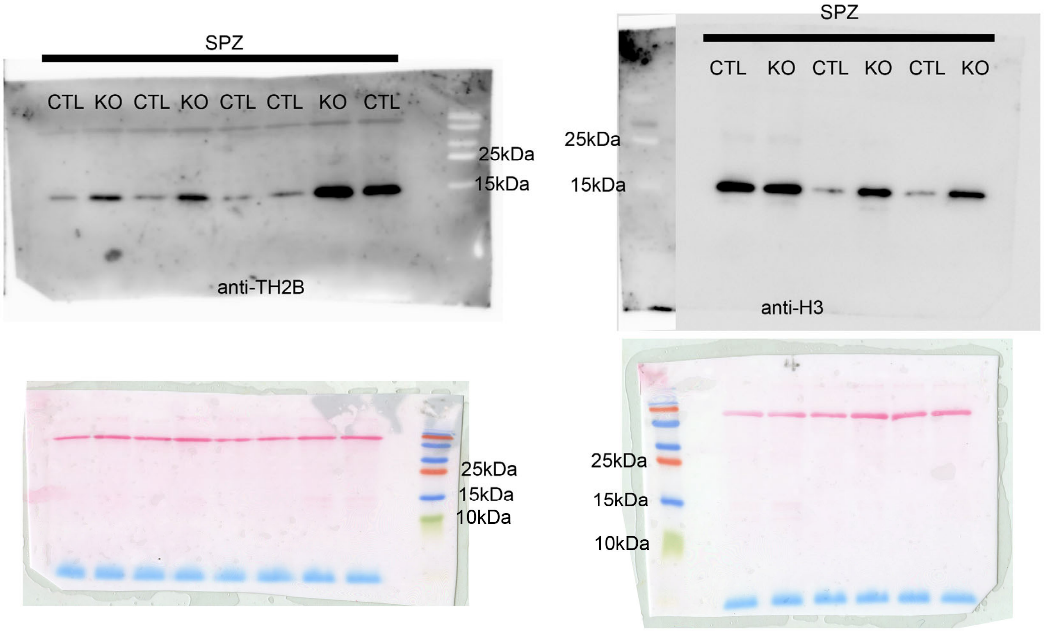

3D

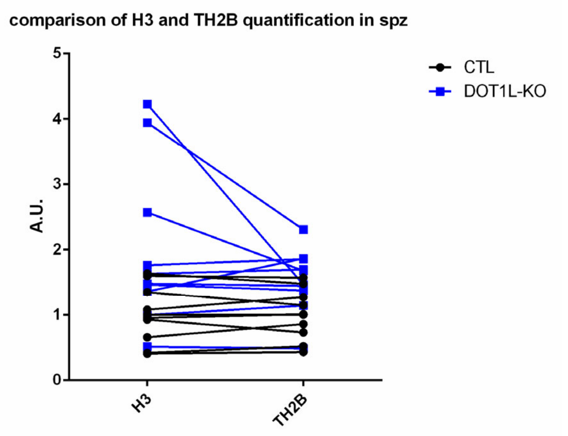

3E

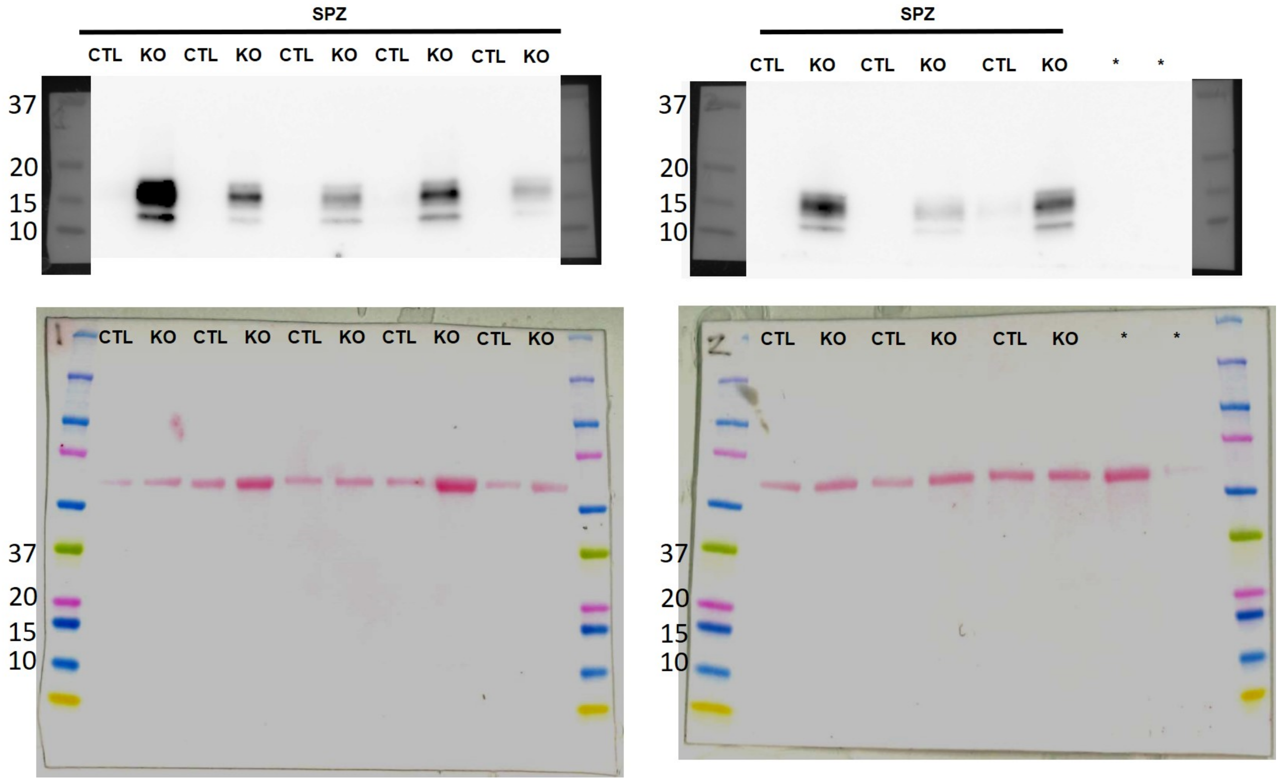
SPZ
CTL KO CTL KO CTL KO CTL KO CTL KO
37
20
15
10
SPZ
CTL KO CTL KO CTL KO * *
CTL KO CTL KO CTL KO CTL KO CTL KO
CTL KO CTL KO CTL KO * *

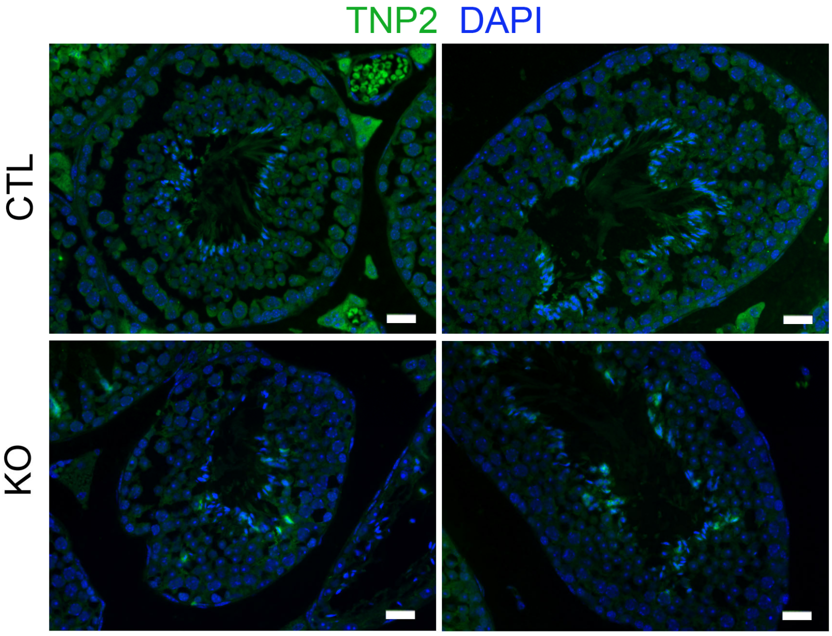
TNP2 DAPI
CTL
KO

3F

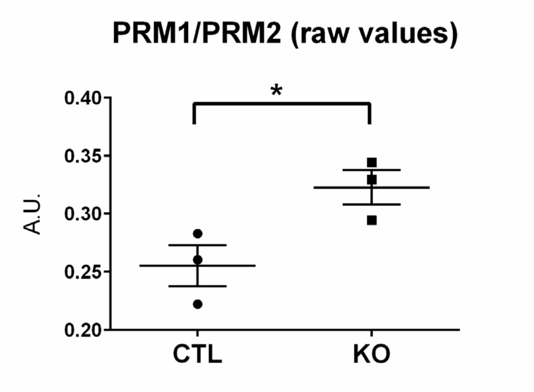

Appendix Figure S3. Complement to Figure 3. A) Sperm vitality. Bar graph showing sperm vitality measured by eosin-nigrosine staining in which dead spermatozoa appear violet (mean ± SEM). Sperm samples from 4 CTL and 4 *Dot1l*-KO males were analyzed. NS indicates non-significant difference between the two genotypes (t-test). **B) *Dot1l*-KO sperm chromatin is sensitive to nucleoplasmin-induced decompaction.** Quantification of histone H3 levels following NPM (nucleoplasmin) partial decondensation of CTL and *Dot1l*-KO (KO) epididymal spermatozoa. Quantification was performed on the two fractions obtained after centrifugation: (i) the supernatant which contains histones from the NPM-de-compacted chromatin, and ii) the pellet which contains compact chromatin, i.e. “resistant” to the NPM treatment we used. Left panel: Bar graph showing quantification results of supernatant/pellet levels in 3 independent experiments (E1, E2 and E3, which are biological replicates). Right panel: Images of western blot using anti-histone H3 antibody (PanH3), corresponding to CTL2 and KO2 (the bottom image was used for quantification). PanH3 is directed against the C-terminal part of histone H3 and detects full length histone H3 at 17 kDa (black arrow, H3-C) and its two cleaved forms previously described in (Yamaguchi et al., 2018): one occurring between Ala21-Thr22 (red arrow) and the other one between Thr22-Arg26 (the smallest form at 13 kDa, green arrow). **C)** Uncropped western blot images from TH2B and H3 detections in spermatozoa (shown in Fig 3E), and corresponding Ponceau staining of the membranes. A non-specific band is visible in all lanes with Ponceau; it indicates that similar amounts of material were loaded in all lanes. **D)** Comparison of TH2B and H3 levels in the same CTL (black) and *Dot1l*-KO (blue) samples. The graph shows a good correlation between TH2B and H3 quantifications. **E)** Top panel: uncropped western blot images from TNP2 detection in spermatozoa (shown in Fig 3F), and corresponding Ponceau staining of the membranes. A non-specific band is visible in all lanes with Ponceau; it indicates that similar quantity of material was loaded in all lanes. Bottom panel: immunofluorescence detection of TNP2 (green) on stage VI-VIII testicular sections. At this stage, the chromatin of condensed spermatids is packaged with protamines, and TNP2 signal is not (or weak) in CTL. In *Dot1l*-KO, some condensed spermatids (inside of the tubule) have a stronger signal. DAPI (blue) was used to stain nuclei. **F)** Box plot of the raw PRM1/PRM2 ratio following acid urea gel electrophoresis (mean ± SEM) complement to Fig 3G. A.U. = arbitrary units. *p-value <0.05 (unpaired t-test).

Appendix Figure S4

4A

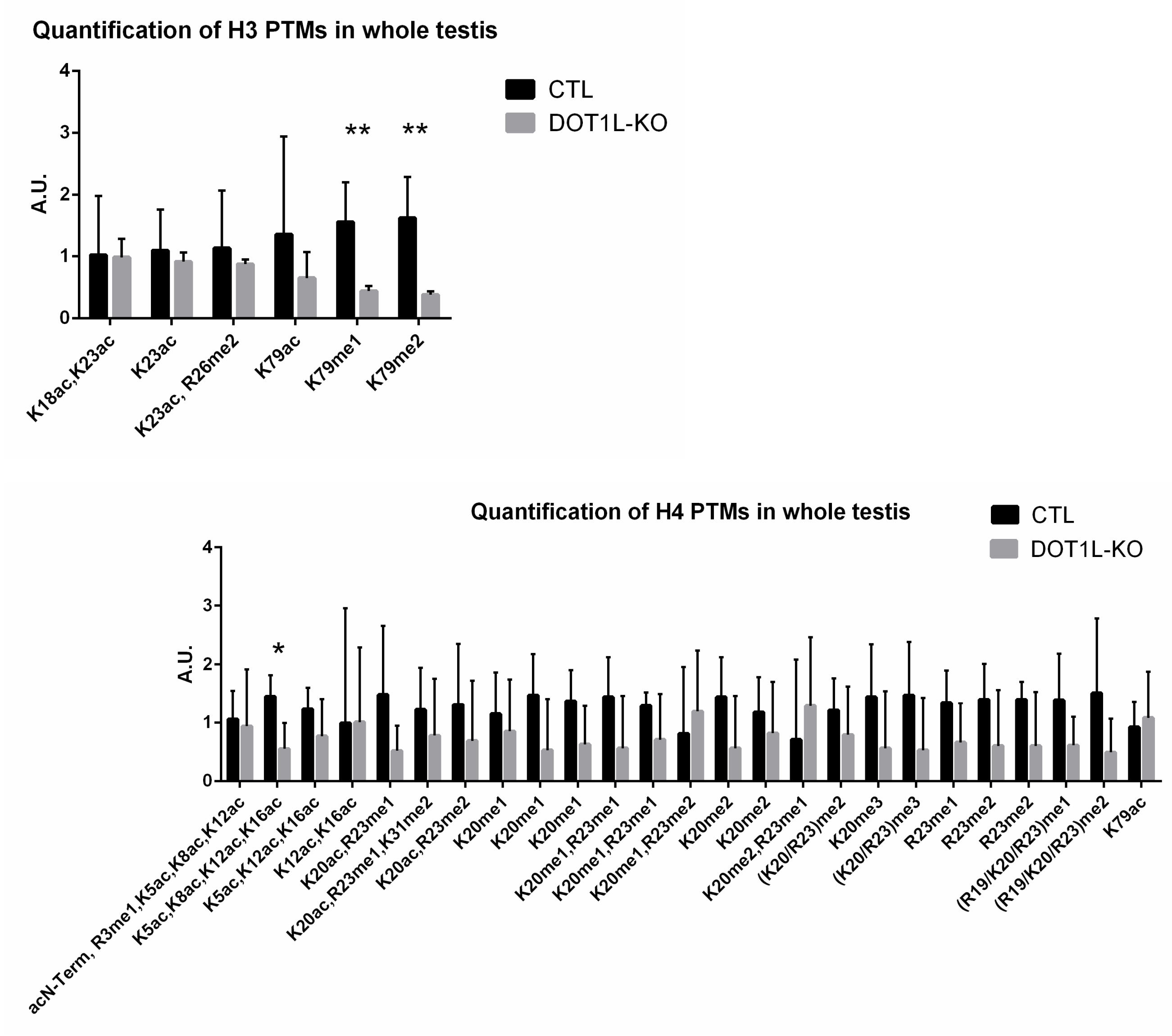

4B

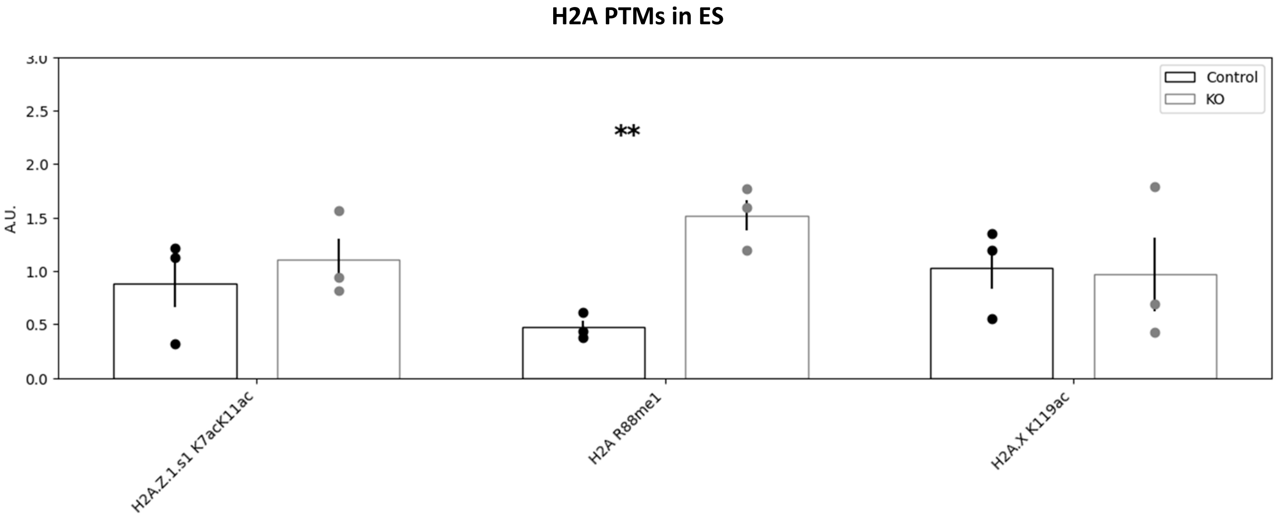
H2A PTMs in ES
Control
KO
**
A.U.
3.0
2.5
2.0
1.5
1.0
0.5
0.0
H2A.Z.1.s1 K7acK11ac
H2A R88me1
H2A.X K119ac

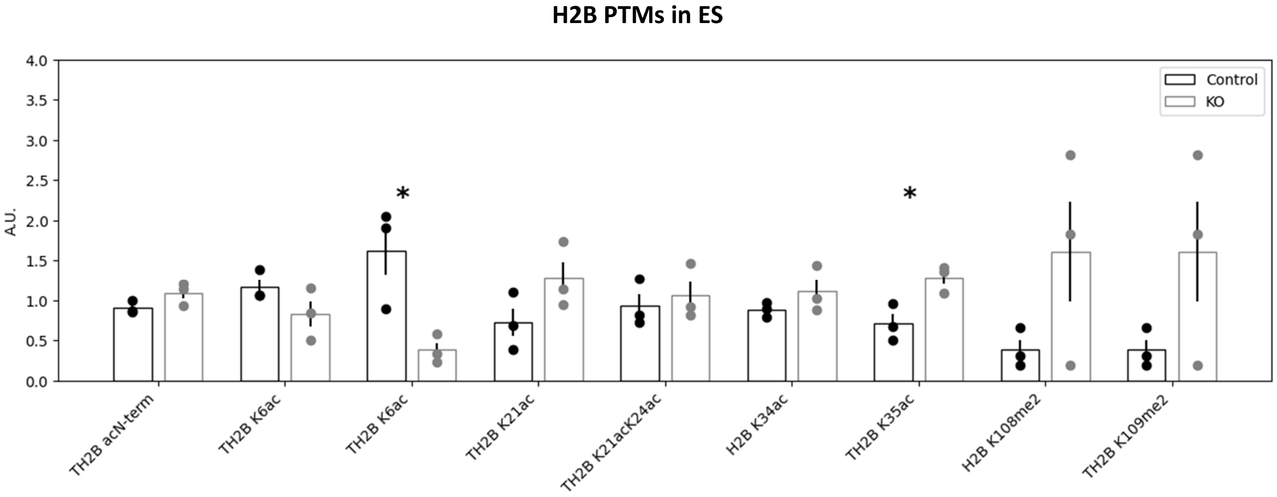
H2B PTMs in ES
Control
KO
*
*
A.U.
4.0
3.5
3.0
2.5
2.0
1.5
1.0
0.5
0.0
TH2B acN-term
TH2B K6ac
TH2B K6ac
TH2B K21ac
TH2B K21acK24ac
H2B K34ac
TH2B K35ac
H2B K108me2
TH2B K109me2

4C

Histone variant levels in ES

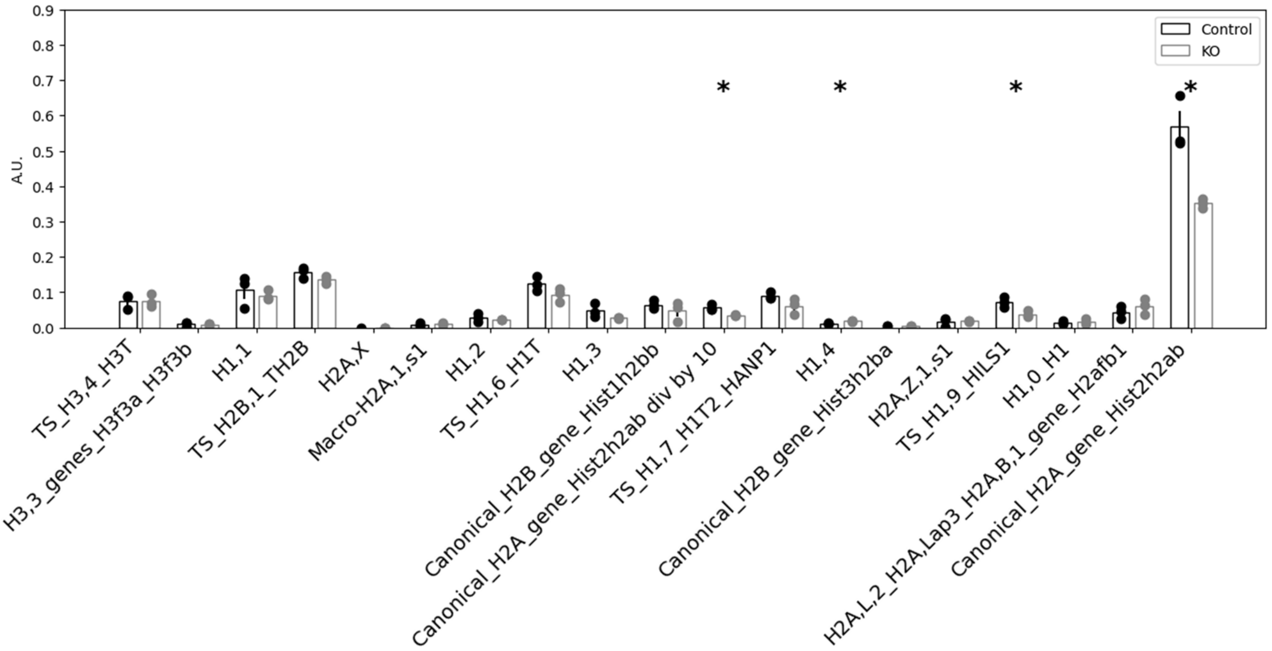

Histone variant levels in RS

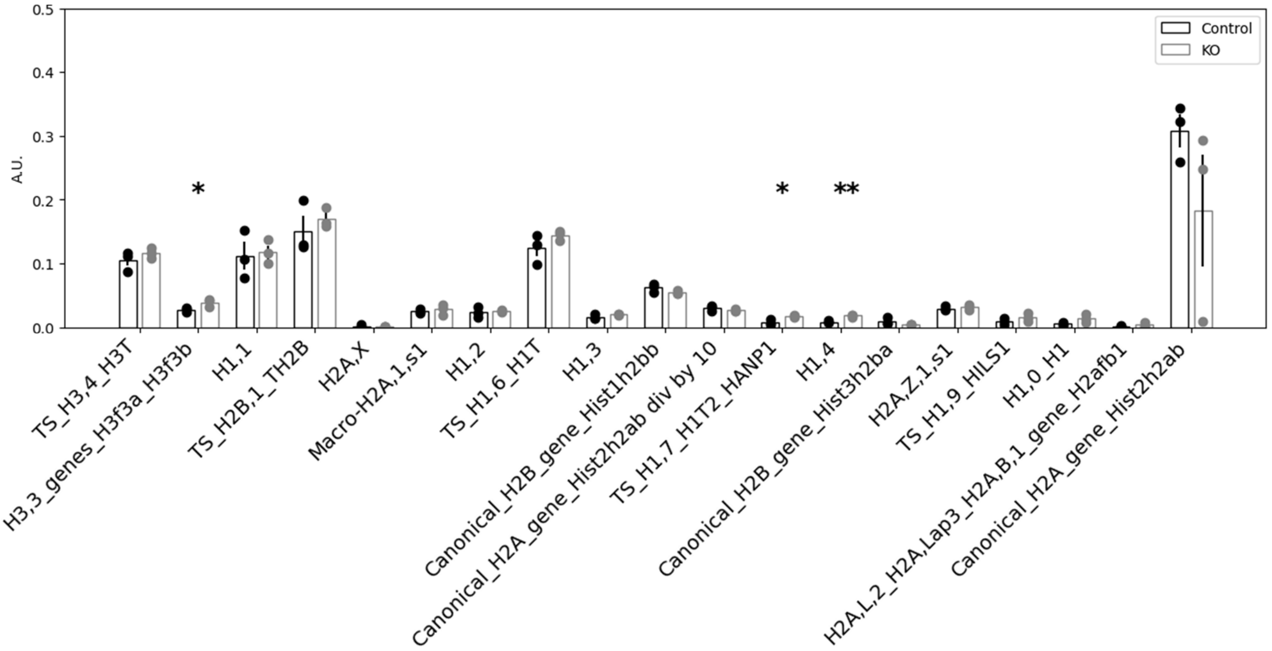

4D

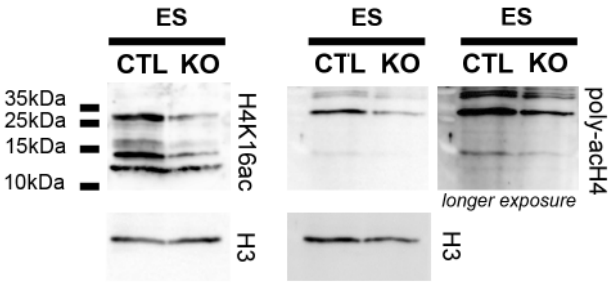
ES
CTL KO
35kDa
25kDa
15kDa
10kDa
H4K16ac
H3
poly-acH4
longer exposure

4E

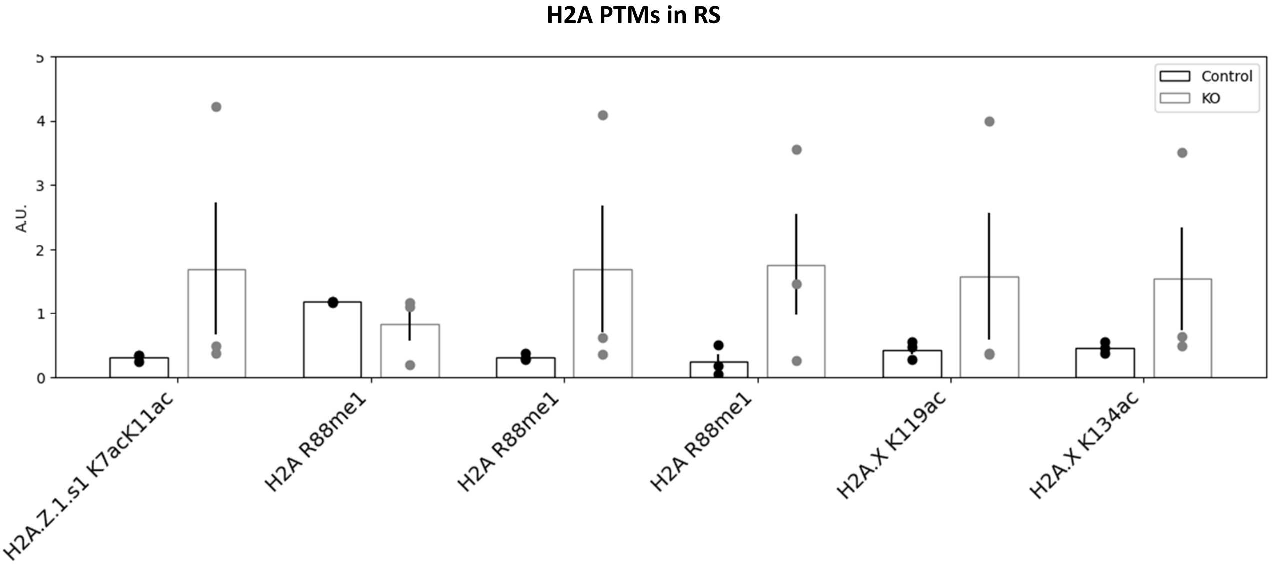
H2A PTMs in RS
Control
KO
A.U.
H2A.Z.1.s1 K7acK11ac
H2A R88me1
H2A R88me1
H2A R88me1
H2A.X K119ac
H2A.X K134ac

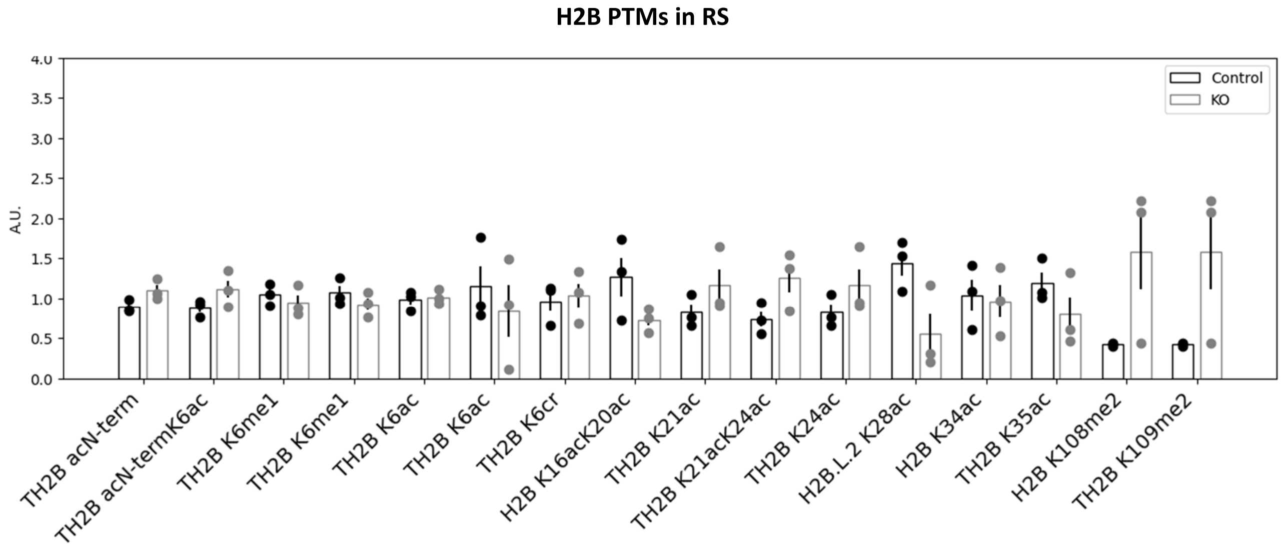
H2B PTMs in RS
Control
KO
A.U.
TH2B acN-term
TH2B acN-termK6ac
TH2B K6me1
TH2B K6me1
TH2B K6ac
TH2B K6ac
TH2B K6cr
H2B K16acK20ac
TH2B K21ac
TH2B K21acK24ac
TH2B K24ac
H2B.L.2 K28ac
H2B K34ac
TH2B K35ac
H2B K108me2
TH2B K109me2

4F

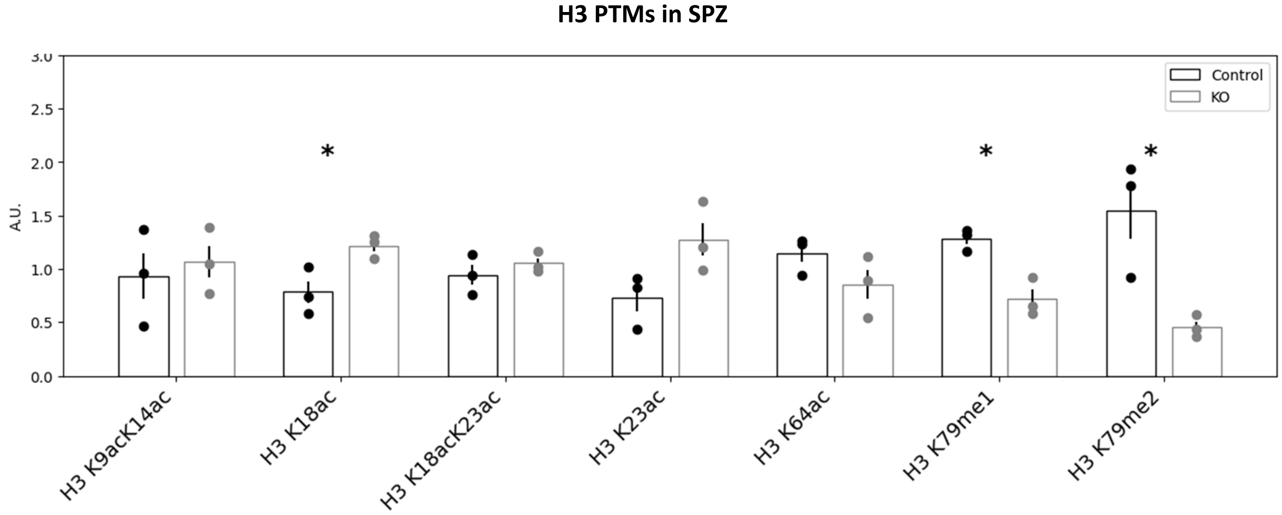

Appendix Figure S4. Complement to Figure 4. Quantification of histone PTMs and variants in *Dot1l*-KO and CTL samples. One star indicates a p-value <0.05, two stars a p-value<0.005 and three stars a p-value <0.0005, obtained with t-tests. **A) Mass spectrometry quantification of H3 and H4 PTMs in whole testes (N= 4 CTL and 4 KO).** After normalization to be at constant amount of histone H3 or H4 in each analyzed sample (see Material and methods), mass spectrometry signals were divided by the average signal in both conditions (CTL and *Dot1l*-KO), so as to be able to represent all peptides in the same figure, whatever their MS intensity. Error bars represent standard deviations calculated on the measurements made on biological replicates. Each modified peptide identified and quantified by MS analysis is defined as a list of PTMs separated by commas. Some modified sites were detected and quantified in several tryptic peptides due to possible missed cleavages (e.g. H4K20me1 was quantified in K20VLRDNIQGITK, K20VLRDNIQGITKPAIR and K20VLRDNIQGITKPAIRR); we indicated the measurements made on such peptides with the same label (K20me1 in the aforementioned example), which allows verifying the coherence of quantitative measurements. In a few instances, a precise localization of the modification site was not possible from the MS/MS spectra. This was typically the case between H4R19, K20 and R23 on which a methyl (me1) and dimethyl (me2) were detected. We indicated such uncertainties by (R19/K20/R23)me1 and (R19/K20/R23)me2, respectively. **B) Mass spectrometry quantification of H2A and H2B in elongating/condensing spermatids (ES) (N=3 CTL and 3 KO).** LC-MS/MS data acquired on H2A and H2B were processed as described above for H3 and H4 in whole testes, except that MS signals detected on modified peptides were normalized to be at constant amount of H2A or H2B in each analyzed sample. **C) Mass spectrometry quantification of histone variants in elongating/condensing spermatids (ES) and in round spermatids (RS).** The abundance of each histone variant was estimated from the sum of intensities of its identified specific proteolytic peptides. The abundance of each protein was then normalized to be at constant amount of histone H4 between samples. For some variants, MS values were divided by 10, so as to be able to visualize all the proteins on the same figure, whatever their MS intensity. Error bars represent standard deviations calculated on the measurements made on biological replicates. **D) Confirmation of a decrease in H4 acetylation in elongating spermatids (ES)**. Western blot detection of H4K16ac, poly-H4ac and H3. Higher molecular weight bands likely represent non-denatured forms of histone H4. Western blot normalization was performed by detecting the same membranes with anti-H3 antibody. Anti-H3 rather than anti-H4 antibody was chosen to avoid interference with acetylated H4 signal. Histones H3 and H4 are organized as obligate tetramers (or occasionally heterodimers) in nucleosomes; therefore, they are expected to be present in the chromatin in the same quantity. **E) Mass spectrometry quantification of histone H2A and H2B PTMs in round spermatids (N= CTL and 3 KO).** LC-MS/MS data acquired on H2A and H2B were processed as described above for ES. **F) Mass spectrometry quantification of histone H3 PTMs in spermatozoa (N= 4 CTL and 4 KO).** Same legend as above. H4 PTMs levels were too weak to be quantified.

Appendix Figure S5

5A

Deregulated genes identified with DEseq2, with a Fold change > 1.5 and FDR < 0.05

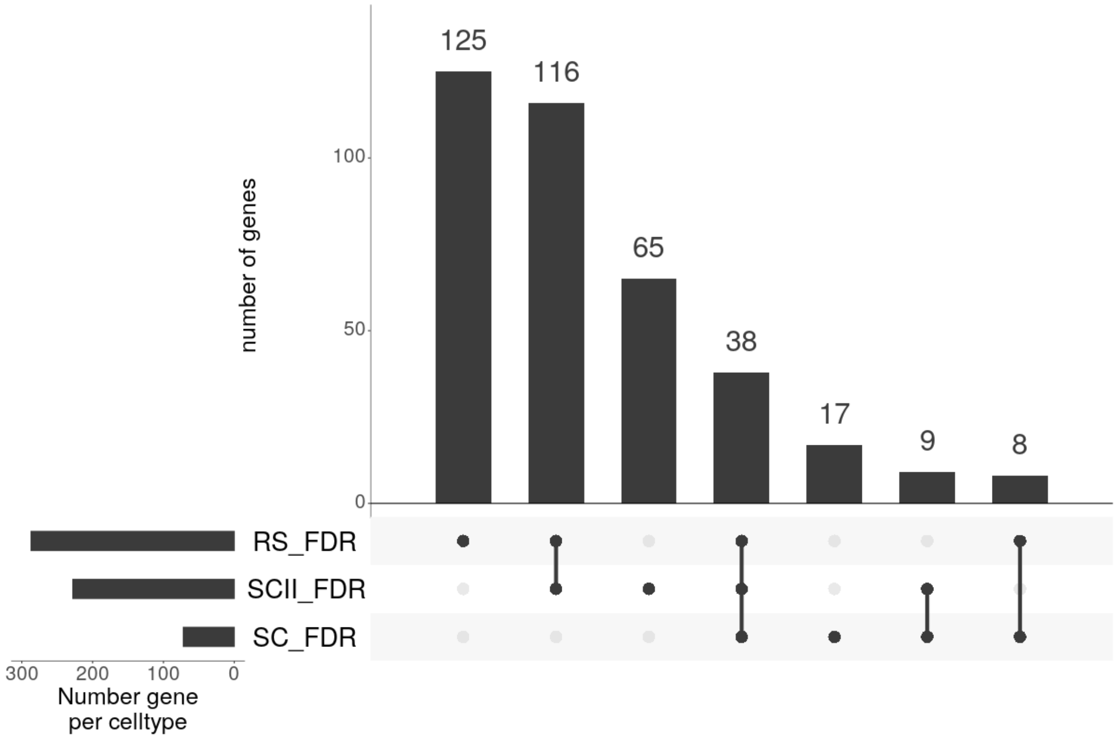

Deregulated genes identified with DEseq2, with a Fold change > 1.5 and p.value < 0.05

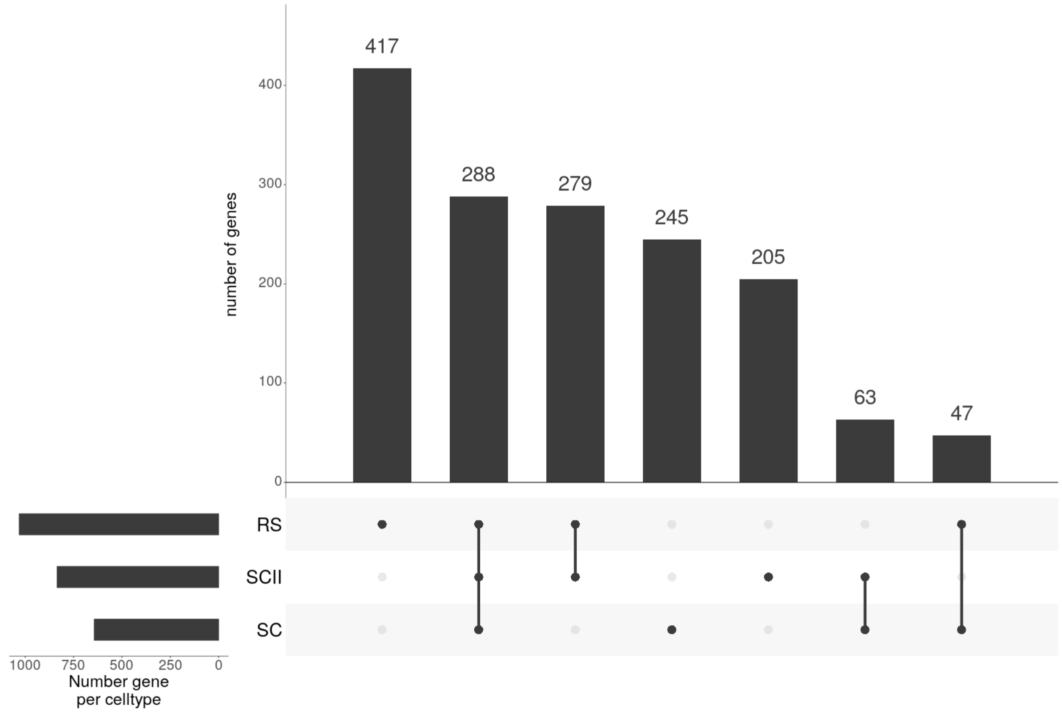

5B

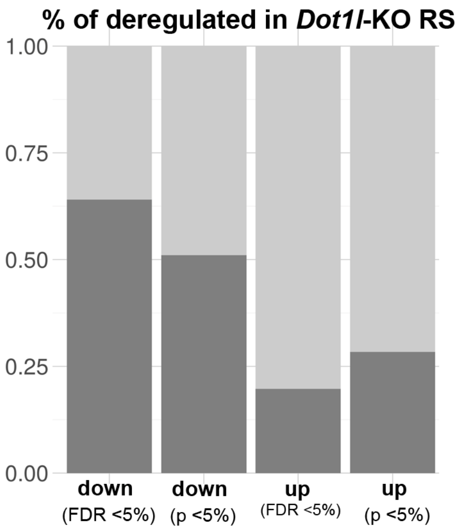

5C

gene_name	logFC	logCPM	PValue	FDR	gl	H3K79me2 enrichment
Slc10a3	-1.26745740995669	0.999004290396908	0.00390006777314618	0.1545955595244	Down-regulated	yes
Slc6a8	-2.46727862572467	0.52781470462503	7.72654921275588e-07	0.000364372904035715	Down-regulated	yes
Slc7a2	-1.0665290941501	2.27338480123705	0.0150834110914719	0.387940102031817	Down-regulated	no
Slc12a1	1.3955625700193	0.681122253169346	0.000169908367523562	0.0174799371397939	Up-regulated	no
Slc13a4	1.54000399067509	2.76366740898566	4.27870310972635e-05	0.00657135254678289	Up-regulated	no
Slc18a2	1.25758541410194	-0.793930612861824	0.00237996137428981	0.110886263192696	Up-regulated	no
Slc1a4	1.10997163431429	0.860924966152978	0.00103762614100171	0.0601552770604185	Up-regulated	no
Slc1a5	1.06878712737505	2.2418481205103	0.00176357549579316	0.0883504245162868	Up-regulated	yes
Slc25a23	0.926874320430452	2.4842629146059	0.00744458216430107	0.240033828551142	Up-regulated	yes
Slc25a37	0.83494237600095	2.8363663126306	0.0494170665013017	0.856402093077086	Up-regulated	no
Slc25a48	0.844791121775937	0.601566750878029	0.035227161221668	0.691260215461133	Up-regulated	yes
Slc26a11	1.65452313717142	1.74006812886016	0.000661650590507997	0.044270891766396	Up-regulated	yes
Slc28a2	0.882471439124064	0.348580484195426	0.0475801028844169	0.835942882356513	Up-regulated	no
Slc29a3	1.00130602592956	3.99897275785481	0.024747552656584	0.549198182271674	Up-regulated	yes
Slc37a3	0.864250867428252	1.06283489334379	0.0157601580162183	0.398622878559951	Up-regulated	no
Slc38a2	1.19621193912572	2.2217849521669	0.000373937024375424	0.0301146206327321	Up-regulated	yes
Slc38a8	1.03918406075334	-0.0156846590541735	0.0201914826533337	0.472852642452675	Up-regulated	no
Slc45a1	1.75519777185066	-0.803109163412898	0.000635433125120995	0.0430016682924086	Up-regulated	no

5D

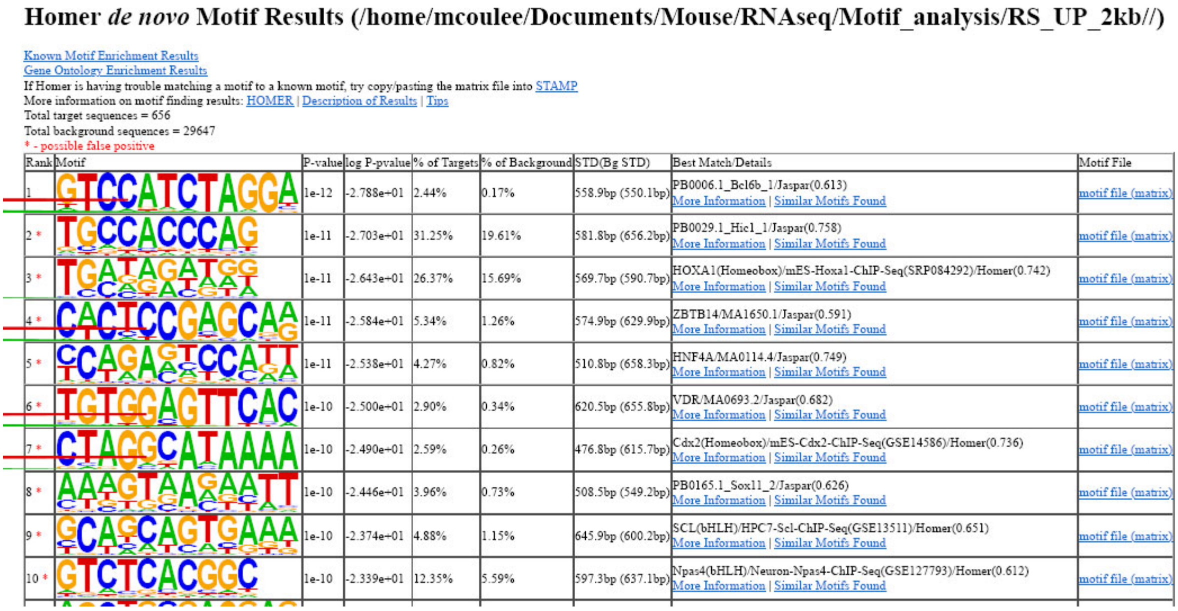

Homer *de novo* Motif Results (/home/mcoulee/Documents/Mouse/RNAseq/Motif_analysis/RS_UP_2kb//)

Known Motif Enrichment Results
Gene Ontology Enrichment Results
If Homer is having trouble matching a motif to a known motif, try copy/pasting the matrix file into STAMP
More information on motif finding results: HOMER | Description of Results | Tips
Total target sequences = 656
Total background sequences = 29647
* - possible false positive

Rank	Motif	P-value	log P-pvalue	% of Targets	% of Background	STD(Bg STD)	Best Match/Details	Motif File
1	GTCCATCTAGGA	1e-12	-2.788e+01	2.44%	0.17%	558.9bp (550.1bp)	PB0006.1_Bcl6b_1/Jaspar(0.613) More Information | Similar Motifs Found	motif file (matrix)
2 *	TGCCACCCAG	1e-11	-2.703e+01	31.25%	19.61%	581.8bp (656.2bp)	PB0029.1_Hic1_1/Jaspar(0.758) More Information | Similar Motifs Found	motif file (matrix)
3 *	TGATAGATGG	1e-11	-2.643e+01	26.37%	15.69%	569.7bp (590.7bp)	HOXA1(Homeobox)/mES-Hoxa1-ChIP-Seq(SRP084292)/Homer(0.742) More Information | Similar Motifs Found	motif file (matrix)
4 *	CACTCCGAGCAA	1e-11	-2.584e+01	5.34%	1.26%	574.9bp (629.9bp)	ZBTB14/MA1650.1/Jaspar(0.591) More Information | Similar Motifs Found	motif file (matrix)
5 *	CCAGAGTCCATT	1e-11	-2.538e+01	4.27%	0.82%	510.8bp (658.3bp)	HNF4A/MA0114.4/Jaspar(0.749) More Information | Similar Motifs Found	motif file (matrix)
6 *	TGTGGAGTTCAC	1e-10	-2.500e+01	2.90%	0.34%	620.5bp (655.8bp)	VDR/MA0693.2/Jaspar(0.682) More Information | Similar Motifs Found	motif file (matrix)
7 *	CTAGGCATAAAA	1e-10	-2.490e+01	2.59%	0.26%	476.8bp (615.7bp)	Cdx2(Homeobox)/mES-Cdx2-ChIP-Seq(GSE14586)/Homer(0.736) More Information | Similar Motifs Found	motif file (matrix)
8 *	AAAGTAAGAATT	1e-10	-2.446e+01	3.96%	0.73%	508.5bp (549.2bp)	PB0165.1_Sox11_2/Jaspar(0.626) More Information | Similar Motifs Found	motif file (matrix)
9 *	GCAGCAGTGAAA	1e-10	-2.374e+01	4.88%	1.15%	645.9bp (600.2bp)	SCL(bHLH)/HPC7-Scl-ChIP-Seq(GSE13511)/Homer(0.651) More Information | Similar Motifs Found	motif file (matrix)
10 *	GTCTCACGGC	1e-10	-2.339e+01	12.35%	5.59%	597.3bp (637.1bp)	Npas4(bHLH)/Neuron-Npas4-ChIP-Seq(GSE127793)/Homer(0.612) More Information | Similar Motifs Found	motif file (matrix)

gene_name	logFC	logCPM	PValue	FDR	gl	H3K79me2 enrichment
Cacna1e	1.381540782	2.204690766	0.002726556	0.121318141	Up-regulated	Yes
Gpsm1	1.115505708	1.018472463	0.002648857	0.119051423	Up-regulated	No
Tmem74b	1.021769193	0.247820597	0.013177832	0.354170753	Up-regulated	No
Gata5	1.673646912	-1.21958131	0.000102450	0.012941114	Up-regulated	No
Padi1	1.006558376	-0.52514070	0.027404915	0.589073299	Up-regulated	No
Ptpn13	0.896515079	3.191552902	0.026370274	0.572363590	Up-regulated	No
Megf8	0.875273628	5.337855272	0.023642554	0.532645802	Up-regulated	No
Kctd21	0.837477696	0.230864728	0.024428532	0.543473782	Up-regulated	No
Irf1	1.521852615	0.397338881	0.006670929	0.224722281	Up-regulated	No
Lhfpl2	1.068600482	-0.60887569	0.028270799	0.599286273	Up-regulated	No
Grid1	1.387320345	2.179807865	0.000243895	0.022484676	Up-regulated	Yes
Dscc1	1.087546432	3.096511586	0.000497737	0.036606338	Up-regulated	No
Baiap2l2	0.874498322	0.165478650	0.034369221	0.684231999	Up-regulated	No
Zfp52	1.351603089	-0.97586843	0.001088020	0.062633064	Up-regulated	No
Plin3	0.983536252	2.207539245	0.007122447	0.233024479	Up-regulated	Yes

Appendix Figure S5. Complement to Fig 5. A) Upset plot showing the number of deregulated genes in *Dot1l*-KO vs CTL primary spermatocytes (SC), secondary spermatocytes (SCII) and round spermatids (RS) obtained using DEseq2 differential gene expression analyses. Top panel: with Fold Change > 1.5 and FDR < 0.05. Bottom panel: with Fold Change>1.5 and p value <0.05 (FDR was calculated *a posteriori* on the selected gene lists and found to be <0.05). **B)** Bar graph showing the percentage of deregulated genes in *Dot1l*-KO RS found with the two types of differential analyses and which are enriched in H3K79me2 at their gene body in WT RS. **C)** List of *Slc* genes found deregulated in *Dot1l*-KO round spermatids. **D)** Top: results from HOMER motif analysis on the promoter (2kb) of upregulated genes (p-value < 0.05) in KO RS (top 10). BCL6/BCL6B binding DNA motif is the most significant of the identified motifs (and the only one which does not appear as "possible false positive"). Bottom: list of genes identified with HOMER which have BCL6 DNA binding site in their promoter.

Appendix Table S1

**Table S1**		
**genotyping primers**	**sequence**	**PCR condition**
**Dot1l-F**	**GCAAGCCTACAGCCTTCATC**	**Annealing temperature = 58°C**
**Dot1l-R1**	**CACCGGATAGTCTCAATAATCTCA**	
**Dot1l-R2**	**GAACCACAGGATGCTTCAG**	
**STRA8CRE_oIMR9266F**	**AGATGCCAGGACATCAGGAACCTG**	**Annealing temperature = 62°C**
**STRA8CRE_oIMR9267R**	**ATCAGCCACACCAGACACAGAGATC**	
**Ymtxfp10_ internal control**	**TCACACAGATAAGAGGGTATTG**	
**Ymtxrp13_ internal control**	**GTTTTCCTATCAGGCCATCCA**	

Appendix Table S2

Table S2			
**Western Blot - Primary antibodies**			
**Target**	**Manufacturer**	**Reference**	**Usage**
**DOT1L**	**CST**	**D4O2T**	**1/500 - 1/1000**
**Tubulin**	**Upstate**	**05-661**	**1/3000**
**H3K79me2**	**Abcam**	**ab3594**	**1/500**
**TH2B**	**Upstate**	**07-680**	**1/5000**
**H3**	**Abcam**	**ab 1791**	**1/3000 - 1/10000**
**H4K16ac**	**Upstate**	**07-329**	**1/750**
**Poly ac-H4**	**Millipore**	**06-866**	**1/250**
**TNP2**	**Santa Cruz Biotechnology**	**sc21106**	**1/250**
**PRM2**	**Briar Patch Biosciences**	**MAb-Hup2B**	**1/500**
**Western Blot - Primary antibodies**			
**Target**	**Manufacturer**	**Reference**	**Usage**
**Donkey anti-goat HRP**	**Santa Cruz Biotechnology**	**sc2020**	**1/5000**
**Goat anti-mouse HRP**	**ThermoFisher**	**31430**	**1/5000**
**Goat anti-rabbit HRP**	**ThermoFisher**	**31460**	**1/5000**
**Immunofluorescence-Immunohistochemistry**			
**Target**	**Brand**	**Reference**	**Usage**
**DOT1L**	**CST**	**D402T**	**1/100**
**DOT1L**	**Abcam**	**64077**	**1/100**
**H3K79me2**	**Diagenode**	**C15410051**	**1/100**
**GFP**	**ThermoFisher**	**CAB4211**	**1/100**
**Poly ac-H4**	**Millipore**	**06-866**	**1/200**
**TNP2**	**Santa Cruz Biotechnology**	**sc21106**	**1/50**
**Chromatin Immunoprecipitation**			
**Target**	**Brand**	**Reference**	
**H3K79me2**	**Diagenode**	**C15410051**	

References

Darde TA, Lecluze E, Lardenois A, Stevant I, Alary N, Tuttelmann F, Collin O, Nef S, Jegou B, Rolland AD et al (2019) The ReproGenomics Viewer: a multi-omics and cross-species resource compatible with single-cell studies for the reproductive science community. Bioinformatics 35: 3133-3139

Darde TA, Sallou O, Becker E, Evrard B, Monjeaud C, Le Bras Y, Jegou B, Collin O, Rolland AD, Chalmel F (2015) The ReproGenomics Viewer: an integrative cross-species toolbox for the reproductive science community. Nucleic Acids Res 43: W109-116

Green CD, Ma Q, Manske GL, Shami AN, Zheng X, Marini S, Moritz L, Sultan C, Gurczynski SJ, Moore BB et al (2018) A Comprehensive Roadmap of Murine Spermatogenesis Defined by Single-Cell RNA-Seq. Developmental cell 46: 651-667 e610

Green CD, Ma Q, Manske GL, Shami AN, Zheng X, Marini S, Moritz L, Sultan C, Gurczynski SJ, Moore BB et al (2018) Gene Expression Omnibus GSE112393 (https://www.ncbi.nlm.nih.gov/geo/query/acc.cgi?acc=GSE112393) [DATASET].

Mouse ENCODE transcriptome data (2011) NCBI Sequence Read Archive PRJNA66167 (https://www.ncbi.nlm.nih.gov/bioproject/?term=PRJNA66167) [DATASET].

Muzumdar MD, Tasic B, Miyamichi K, Li L, Luo L (2007) A global double-fluorescent Cre reporter mouse. Genesis 45: 593-605

Russell LD, Hikim APS, Ettlin RA, Clegg ED (1990) Histological and Histopathological Evaluation of the Testis. Cache River Press
