# Supplementary figures and images for: DOT1L regulates chromatin reorganization and gene expression during sperm differentiation
Source: EMBO Rep. 2023 Apr 26;24(6):e56316. doi: 10.15252/embr.202256316 (PMC10240200; doi:10.15252/embr.202256316)

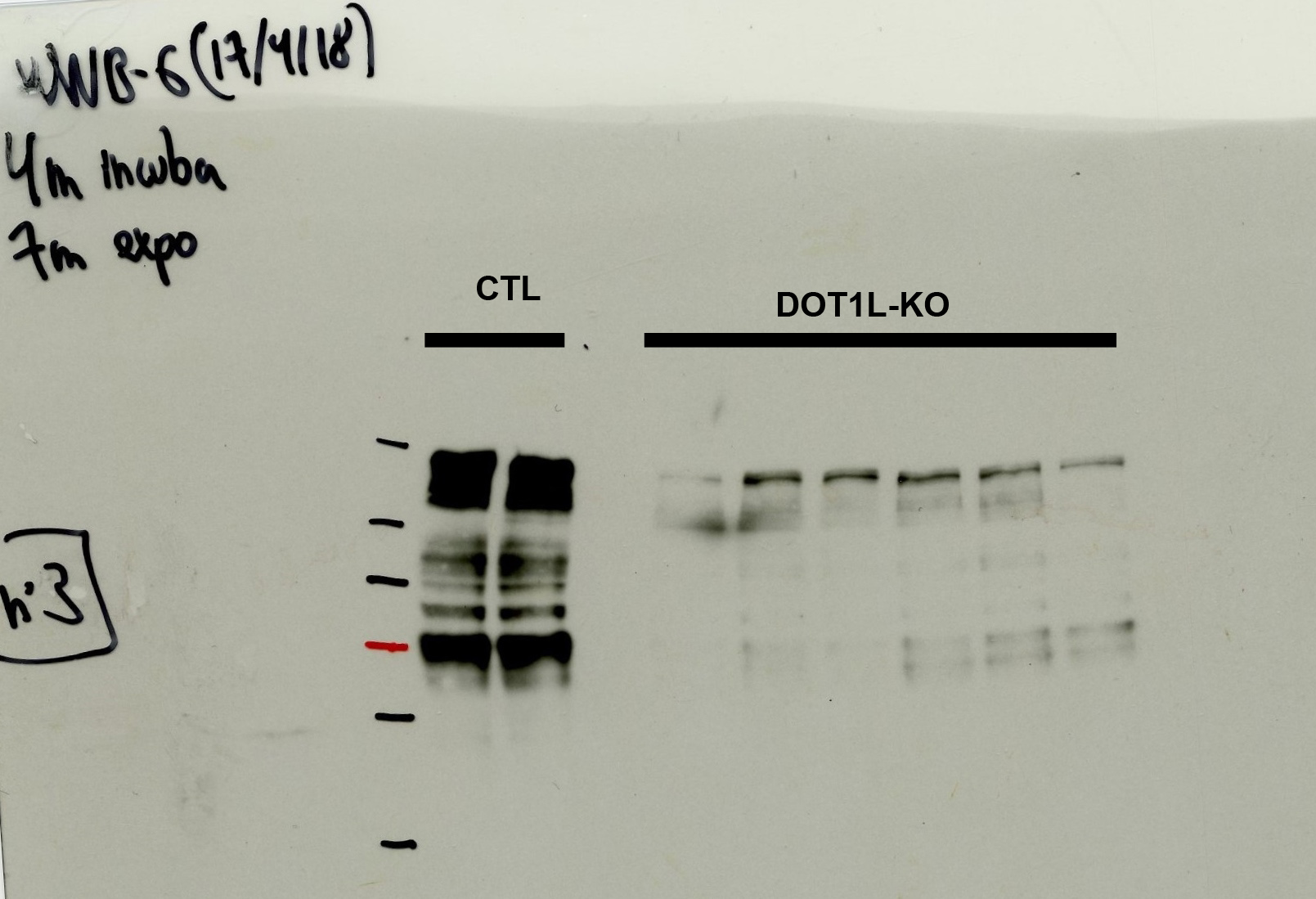

Supplement: Supplementary file 4 — Source Data for Figure 1 [file EMBR-24-e56316-s003.zip › Figure1/1B/1B_source_data_anti_DOT1L_annotated.jpg]

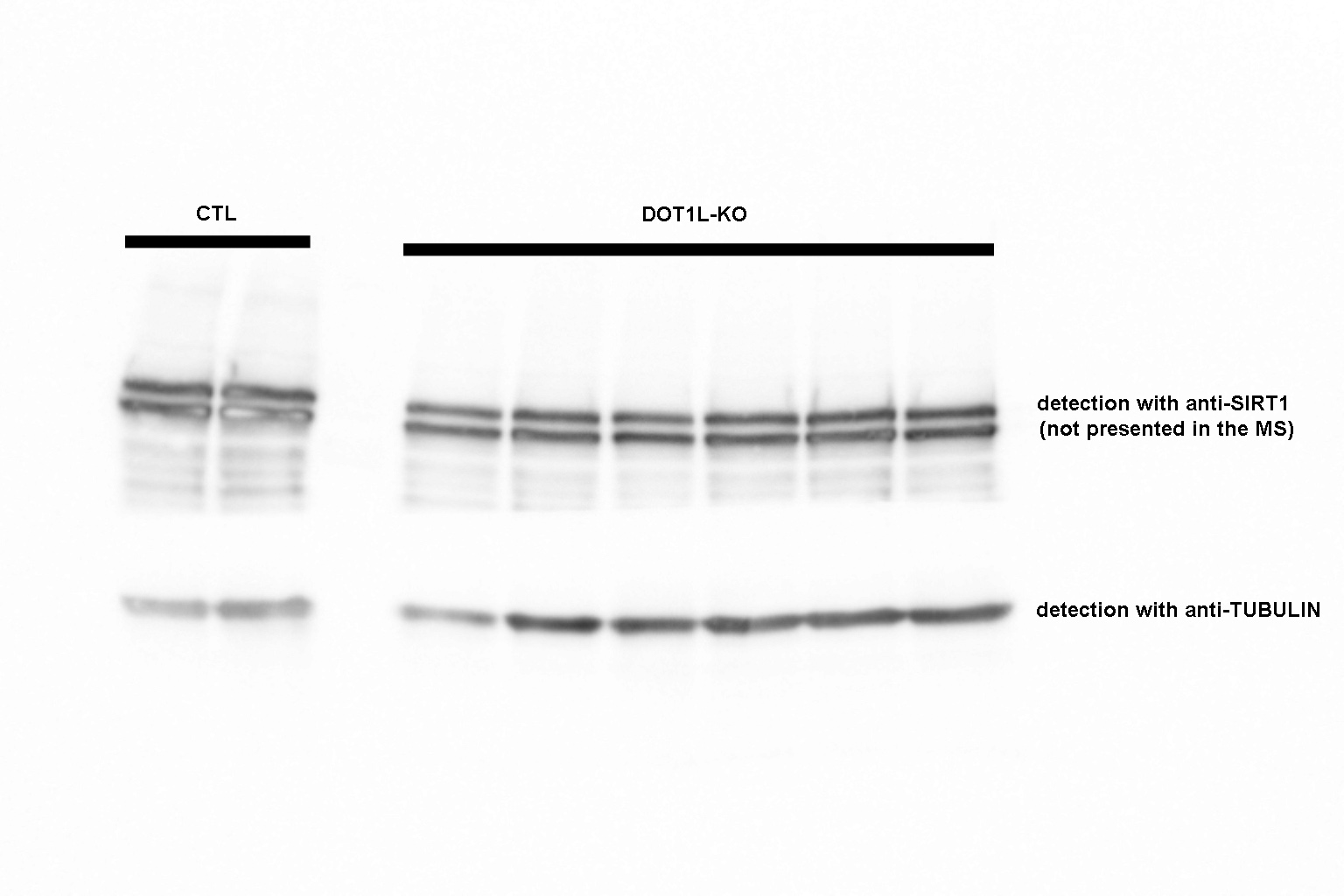

Supplement: Supplementary file 4 — Source Data for Figure 1 [file EMBR-24-e56316-s003.zip › Figure1/1B/1B_source_data_anti_Tubulin_detection_annotated.jpg]

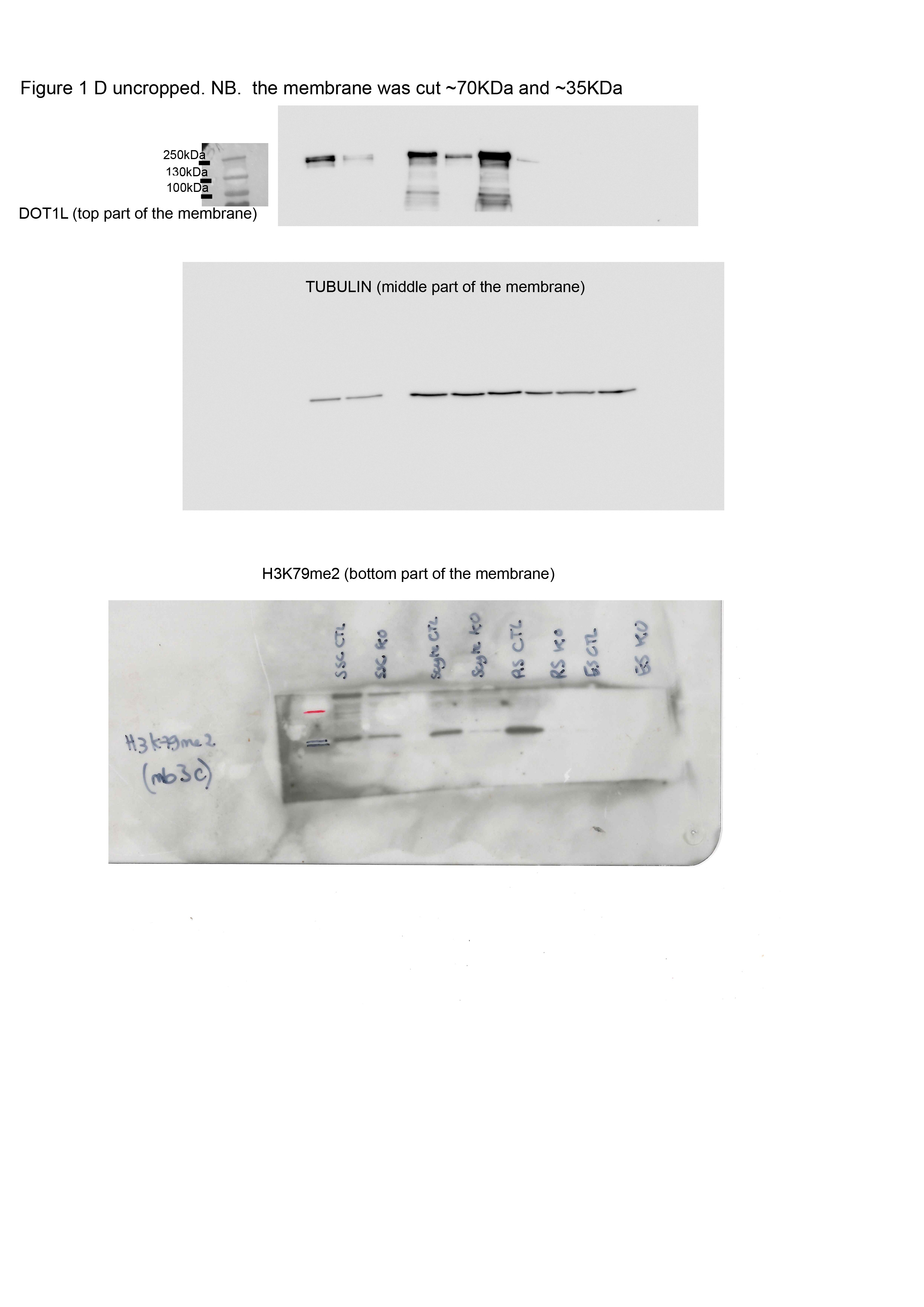

Supplement: Supplementary file 4 — Source Data for Figure 1 [file EMBR-24-e56316-s003.zip › Figure1/1D/Figure1D_uncropped_images.jpg]

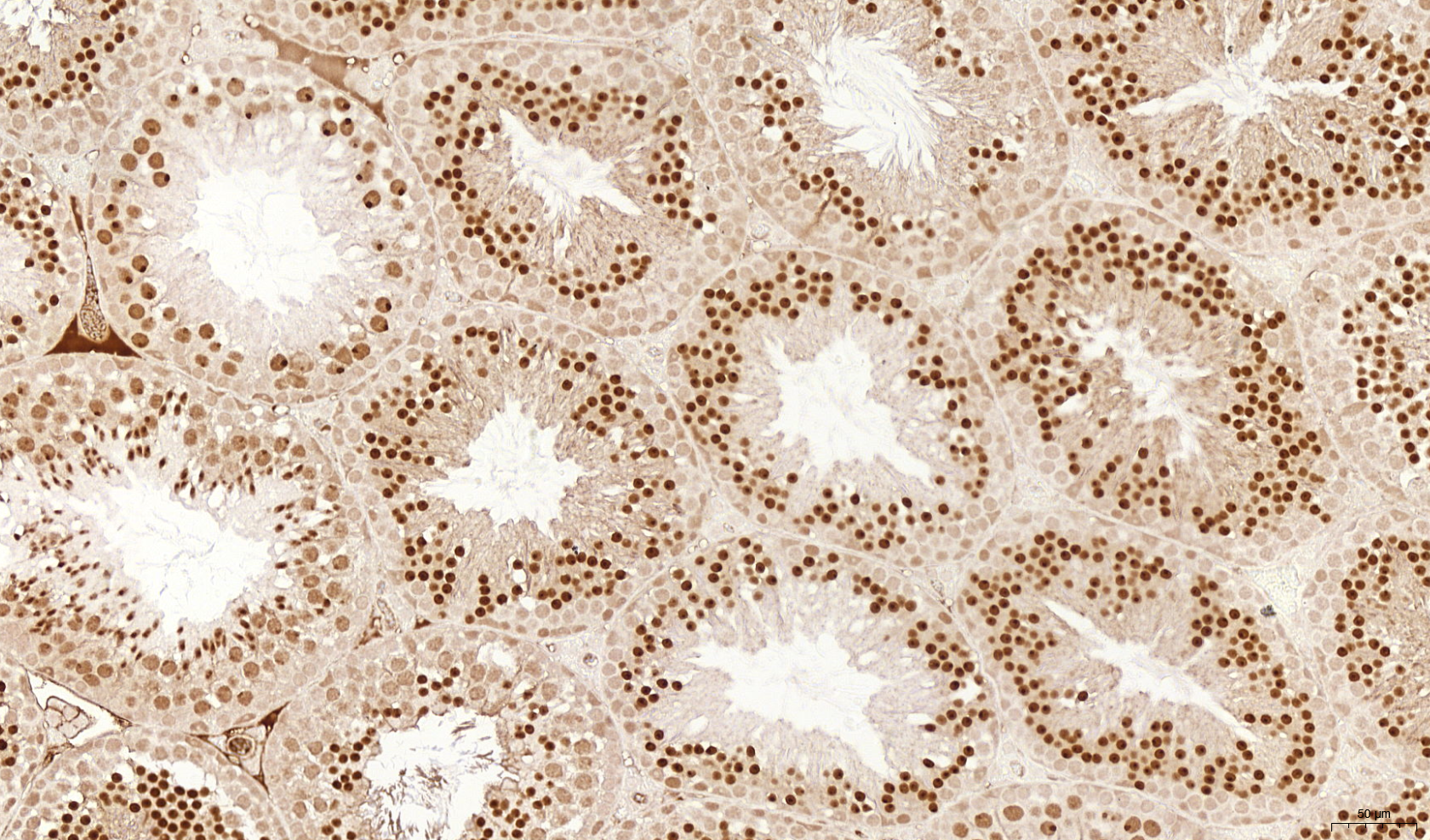

Supplement: Supplementary file 4 — Source Data for Figure 1 [file EMBR-24-e56316-s003.zip › Figure1/1C/1C_CTL.tif]

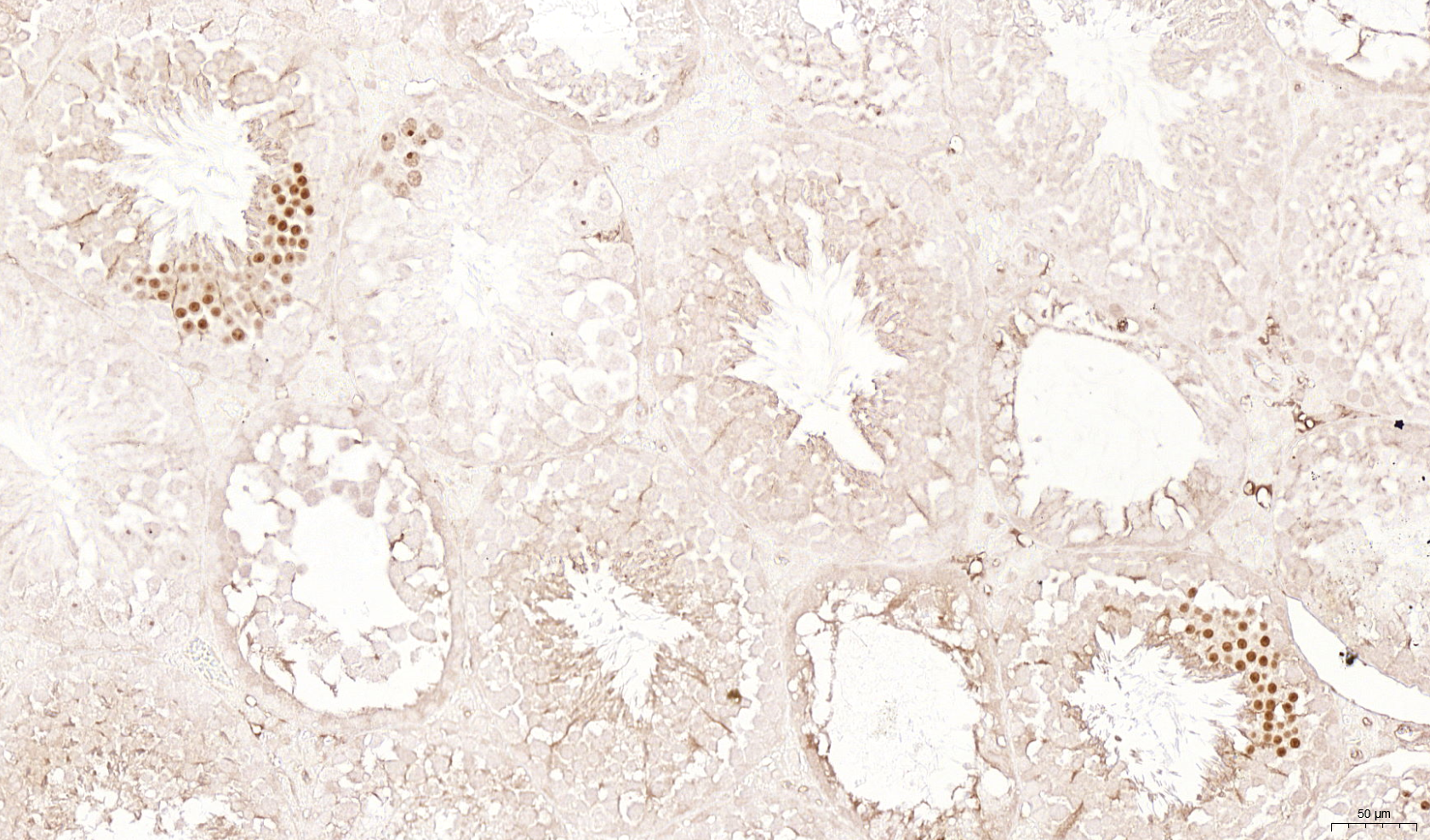

Supplement: Supplementary file 4 — Source Data for Figure 1 [file EMBR-24-e56316-s003.zip › Figure1/1C/1C_KO.tif]

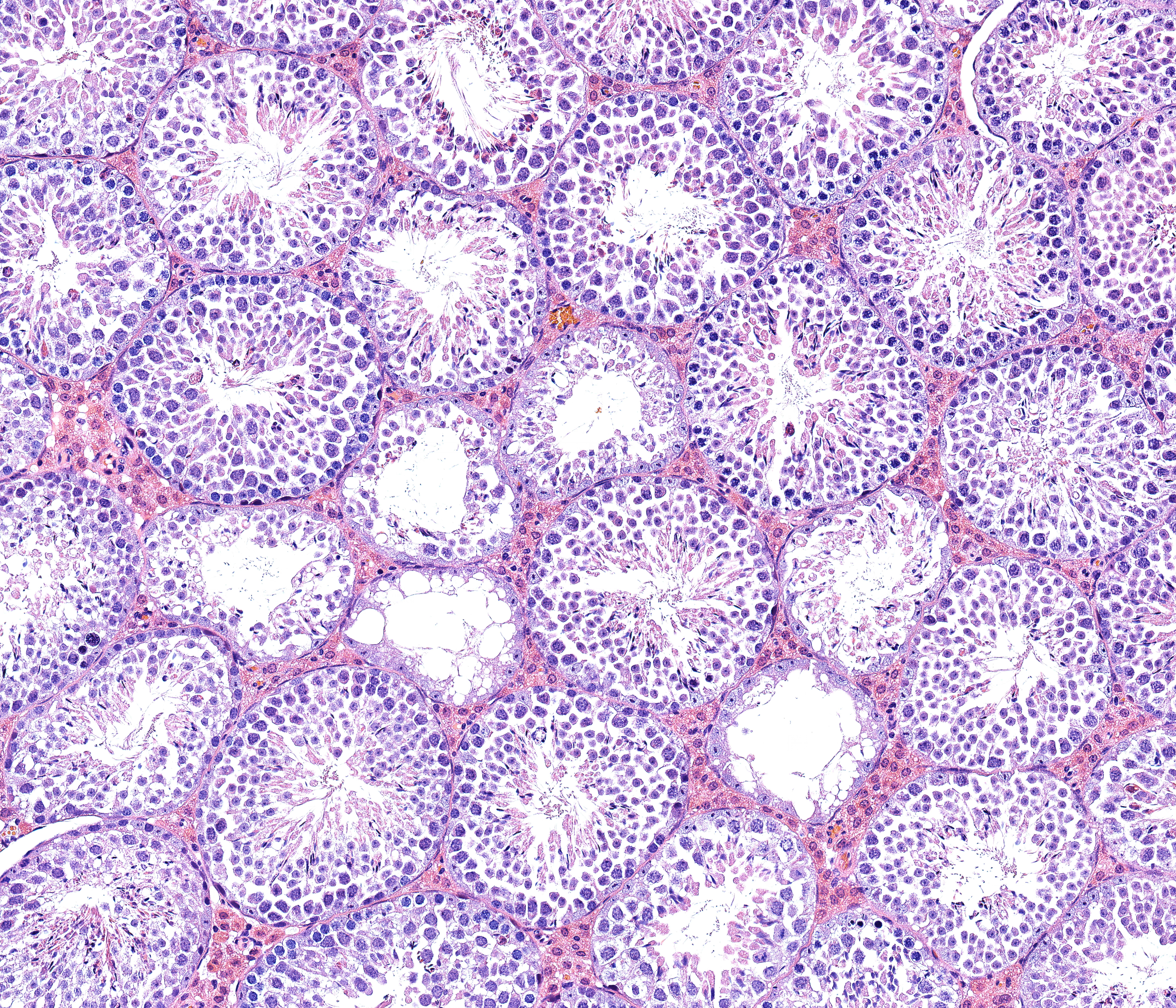

Supplement: Supplementary file 5 — Source Data for Figure 2 [file EMBR-24-e56316-s002.zip › Figure2/2B/Fig2B_KO.jpg]

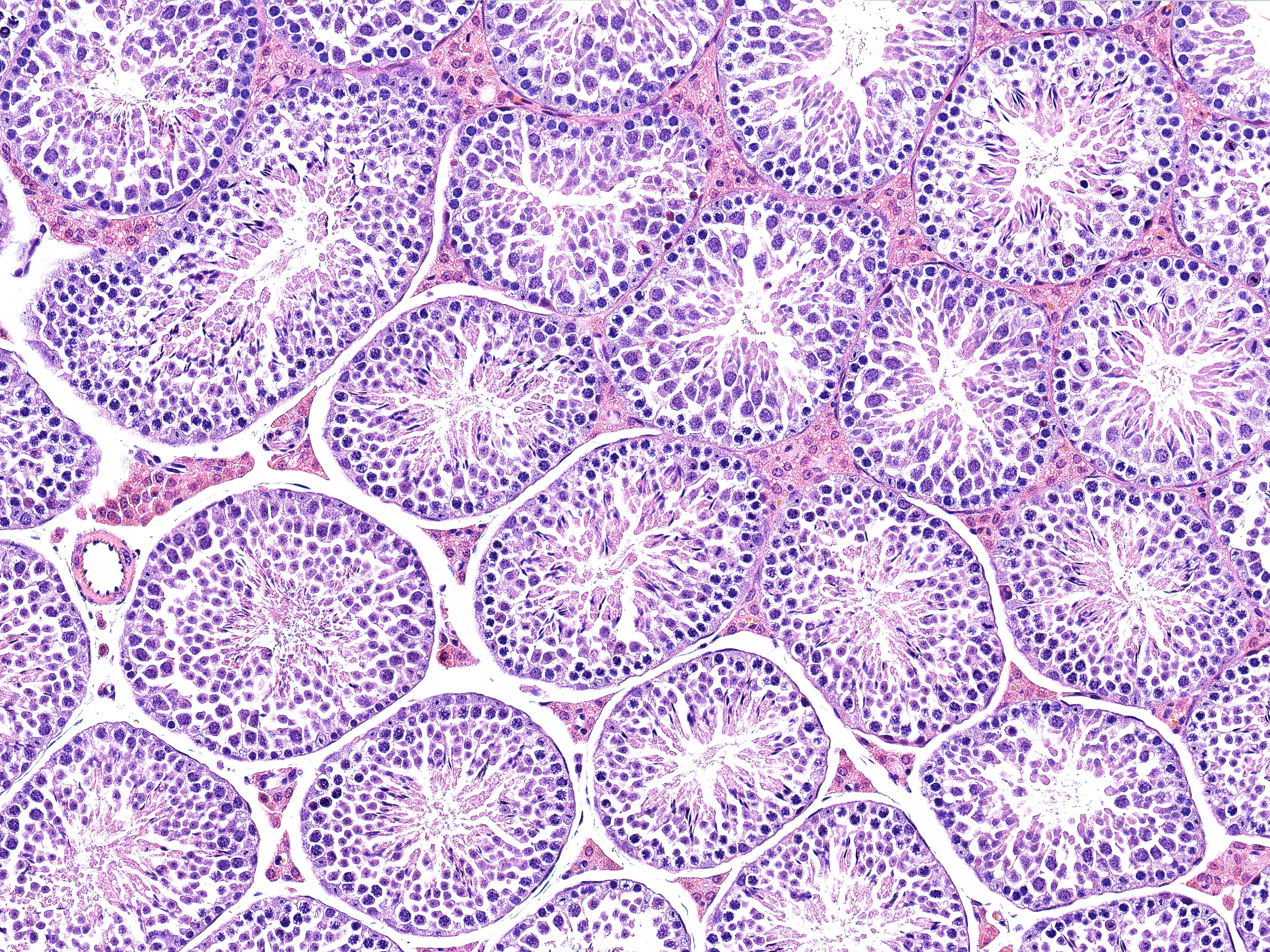

Supplement: Supplementary file 5 — Source Data for Figure 2 [file EMBR-24-e56316-s002.zip › Figure2/2B/Fig2B_CTL.jpg]

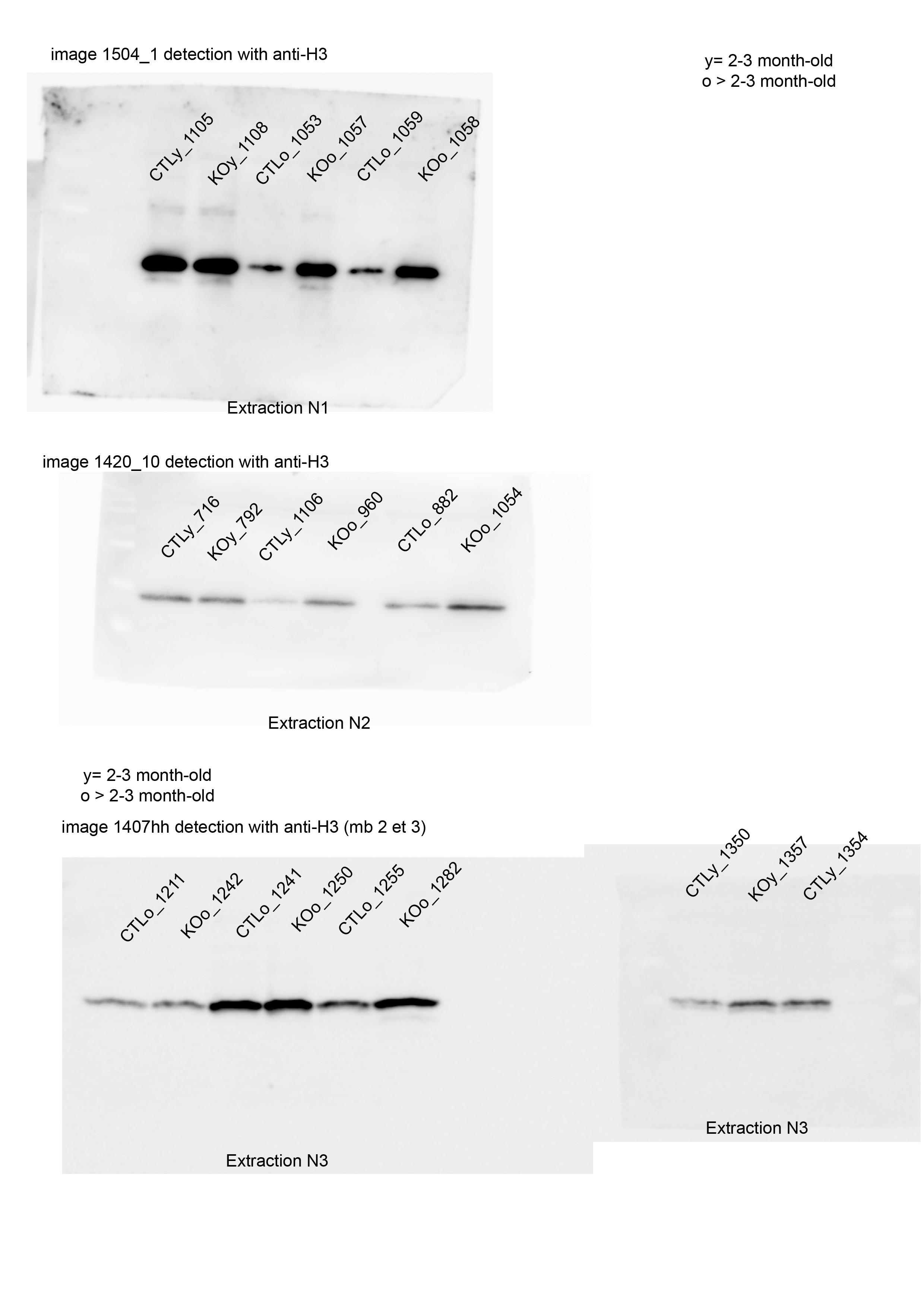

Supplement: Supplementary file 6 — Source Data for Figure 3 [file EMBR-24-e56316-s005.zip › Figure3/3E/source_data_Fig3E_bottom.jpg]

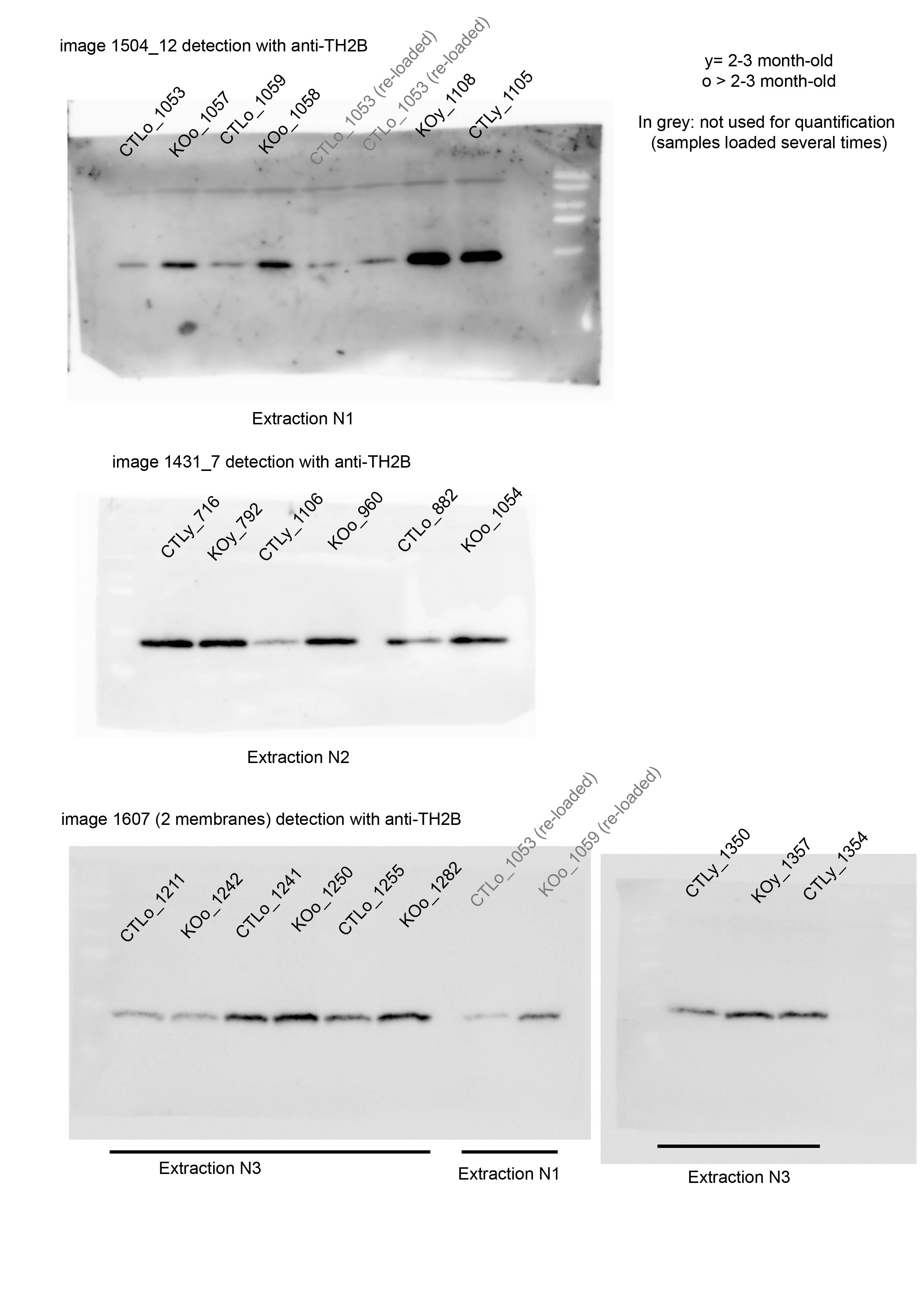

Supplement: Supplementary file 6 — Source Data for Figure 3 [file EMBR-24-e56316-s005.zip › Figure3/3E/source_data_Fig3E_top.jpg]

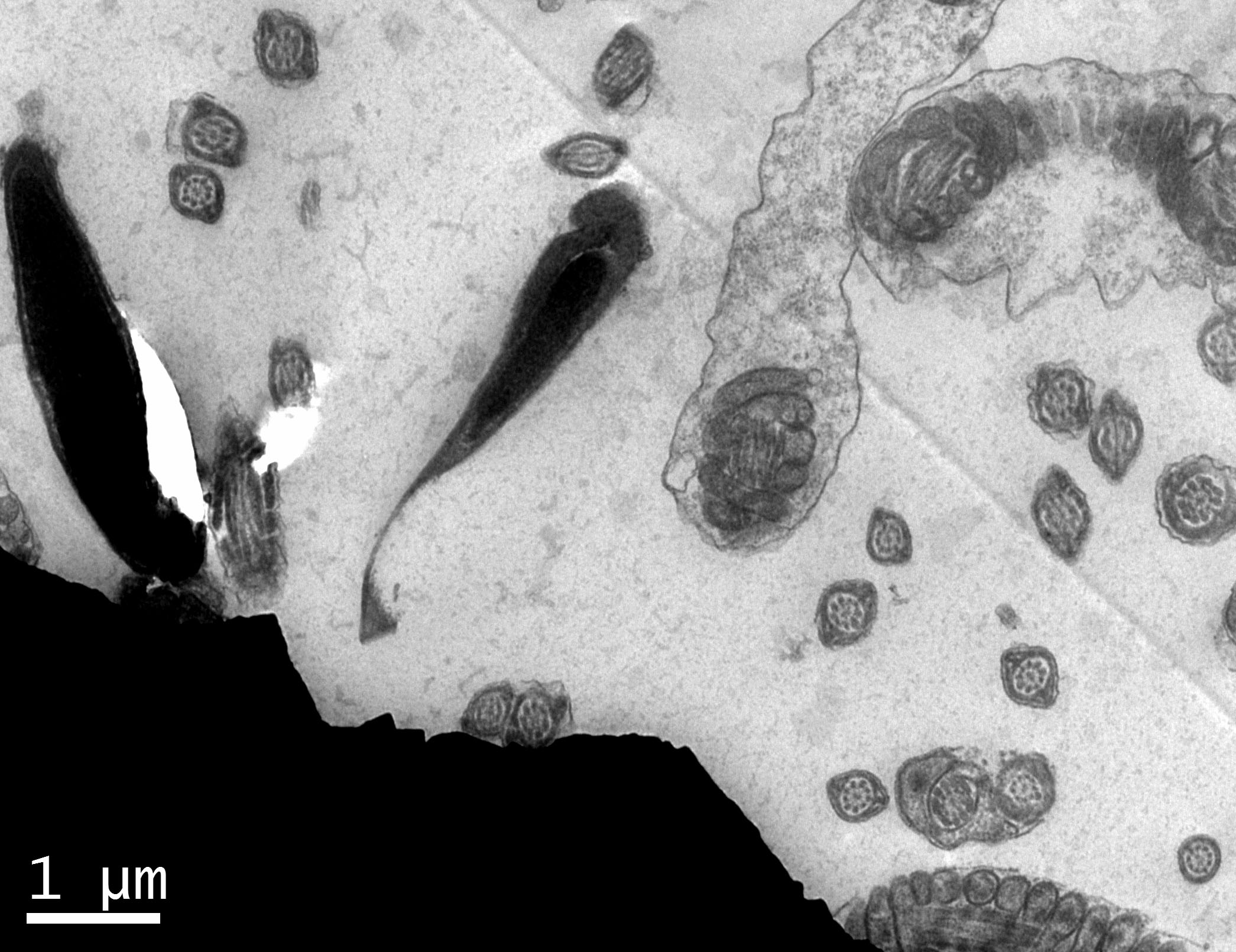

Supplement: Supplementary file 6 — Source Data for Figure 3 [file EMBR-24-e56316-s005.zip › Figure3/3B/3B_CTL_pict2.jpg]

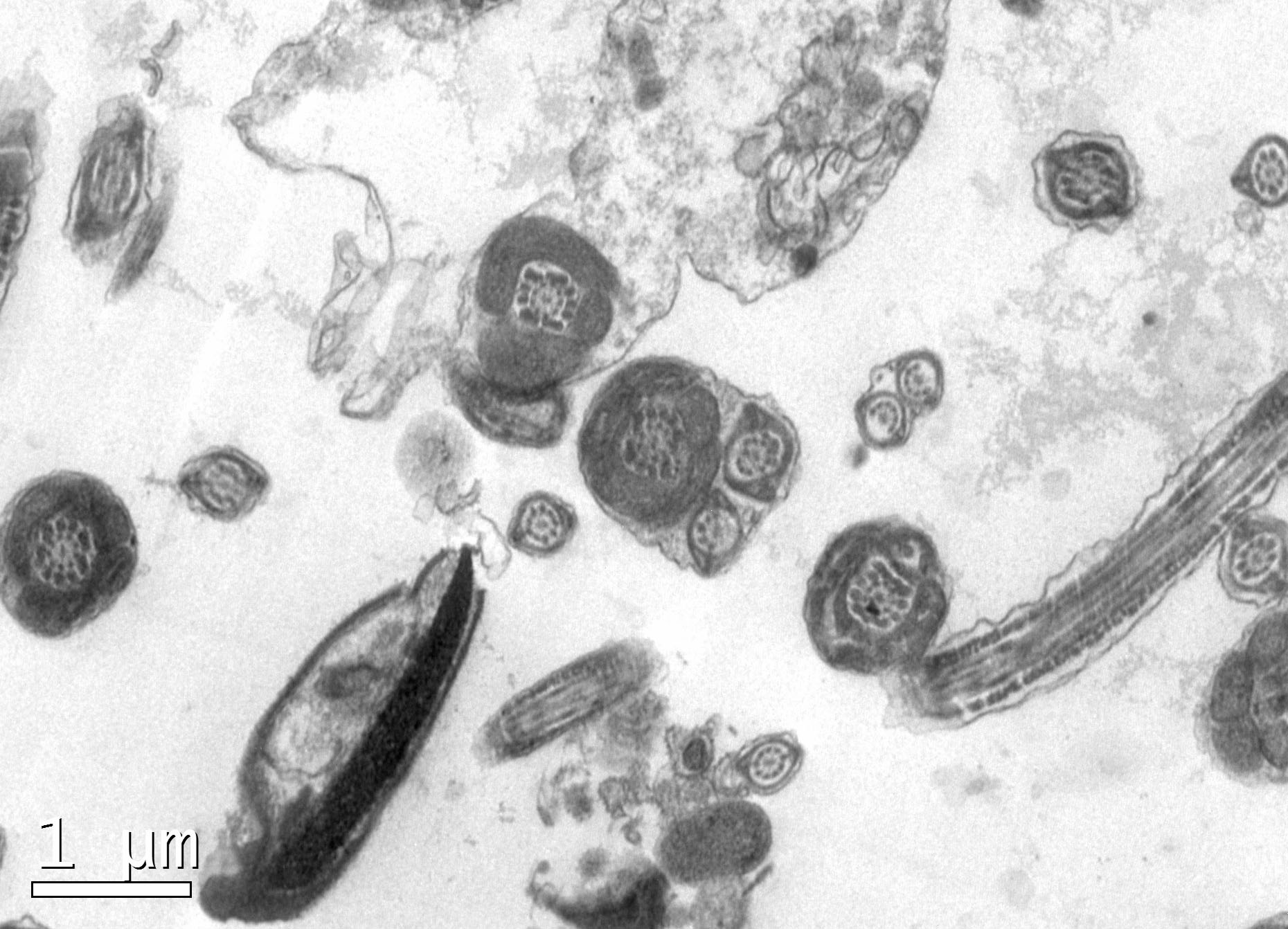

Supplement: Supplementary file 6 — Source Data for Figure 3 [file EMBR-24-e56316-s005.zip › Figure3/3B/3B_KO_pict3.jpg]

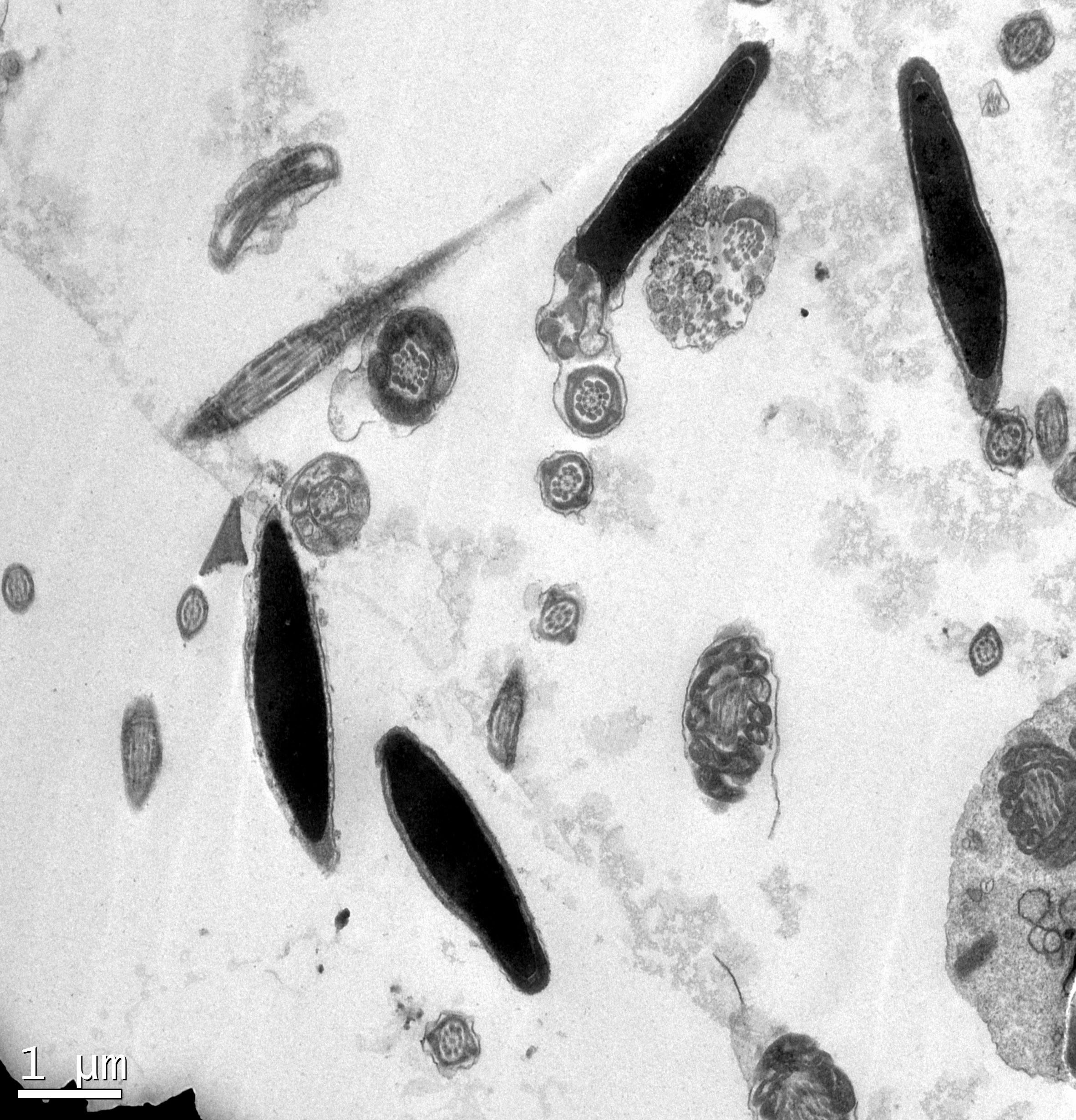

Supplement: Supplementary file 6 — Source Data for Figure 3 [file EMBR-24-e56316-s005.zip › Figure3/3B/3B_CTL_pict1.jpg]

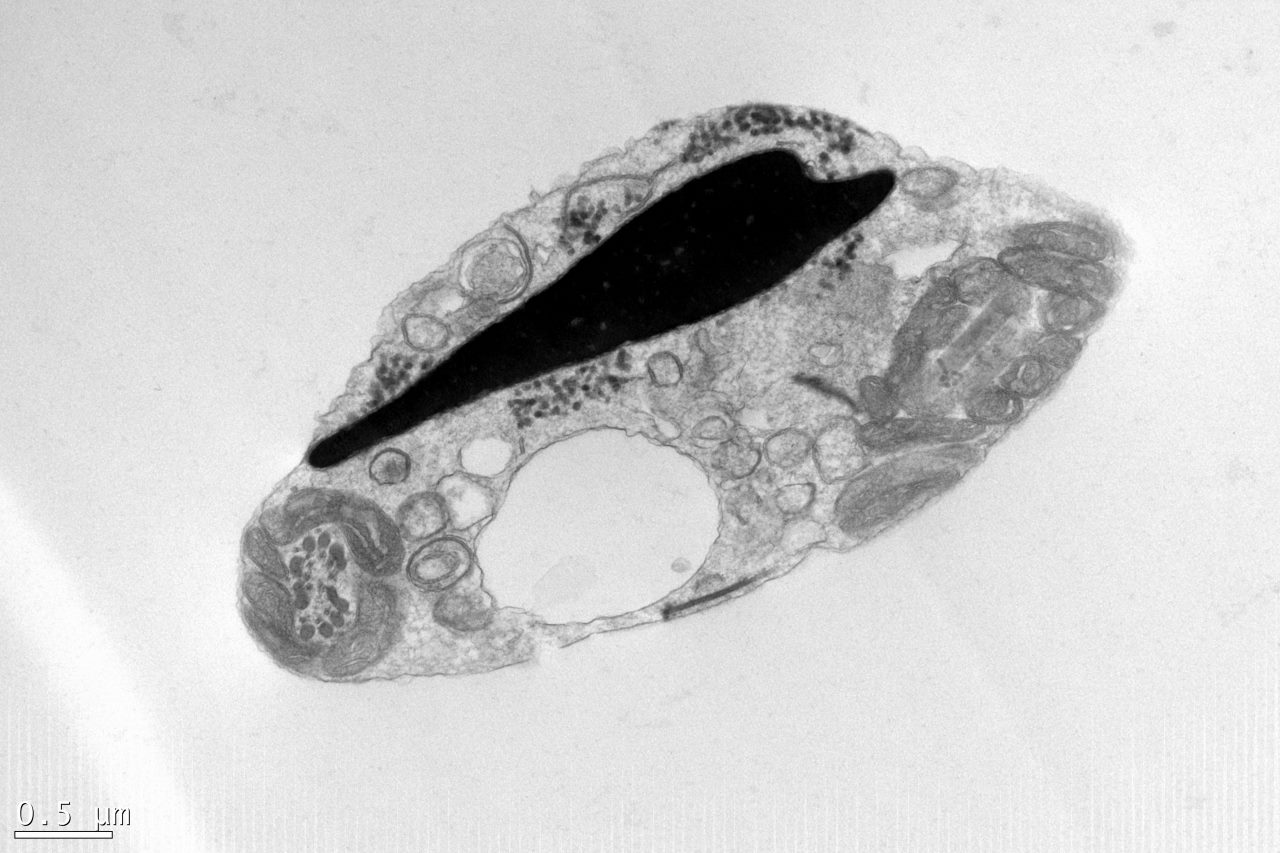

Supplement: Supplementary file 6 — Source Data for Figure 3 [file EMBR-24-e56316-s005.zip › Figure3/3B/3B_KO_pict4.tif]

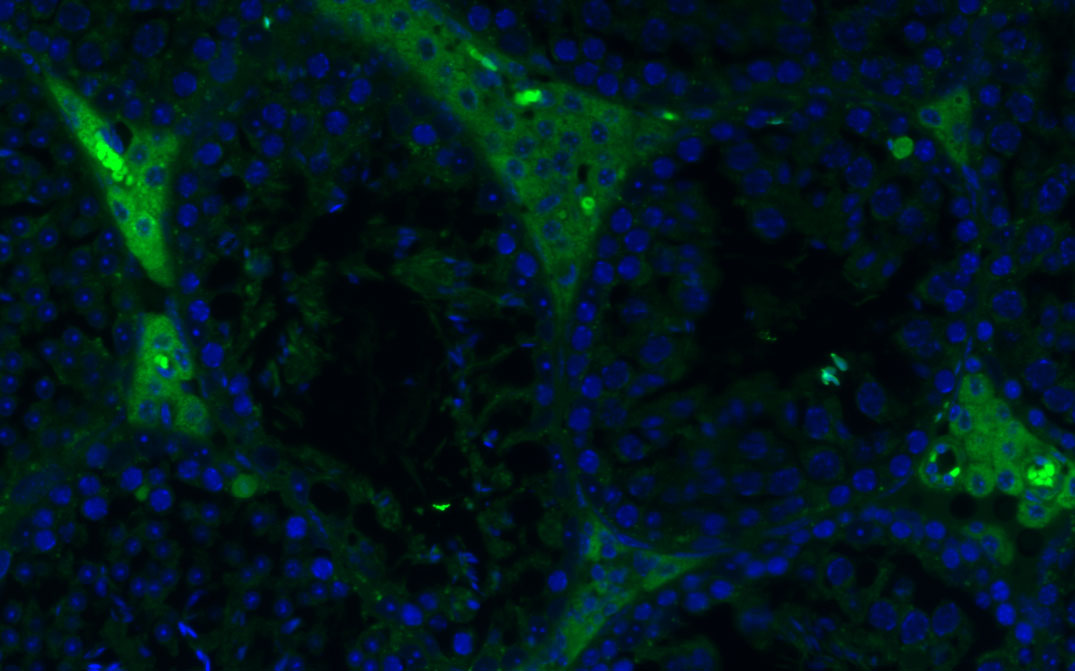

Supplement: Supplementary file 6 — Source Data for Figure 3 [file EMBR-24-e56316-s005.zip › Figure3/3D/Fig3D.jpg]

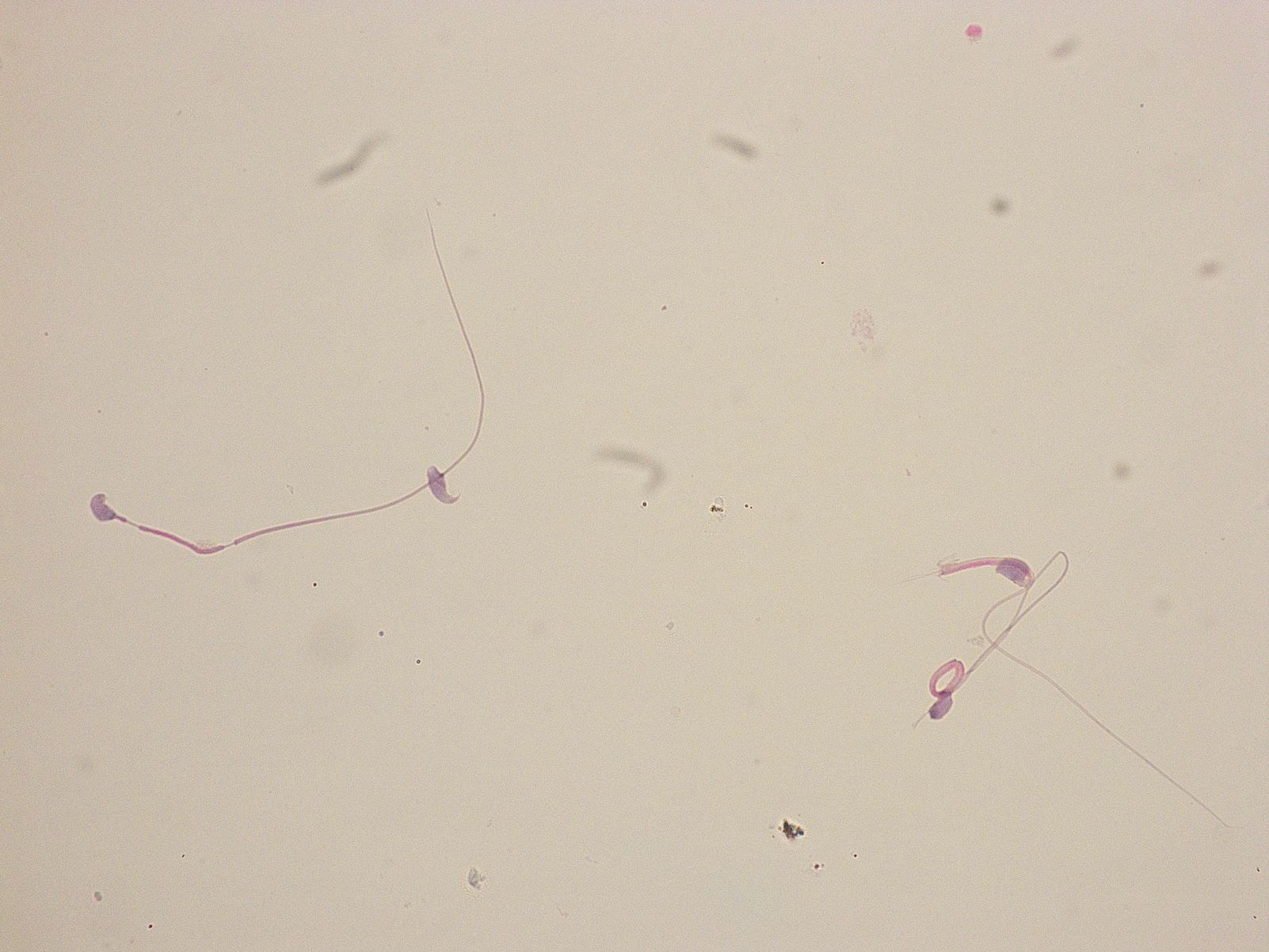

Supplement: Supplementary file 6 — Source Data for Figure 3 [file EMBR-24-e56316-s005.zip › Figure3/3A/3A_KO.tif]

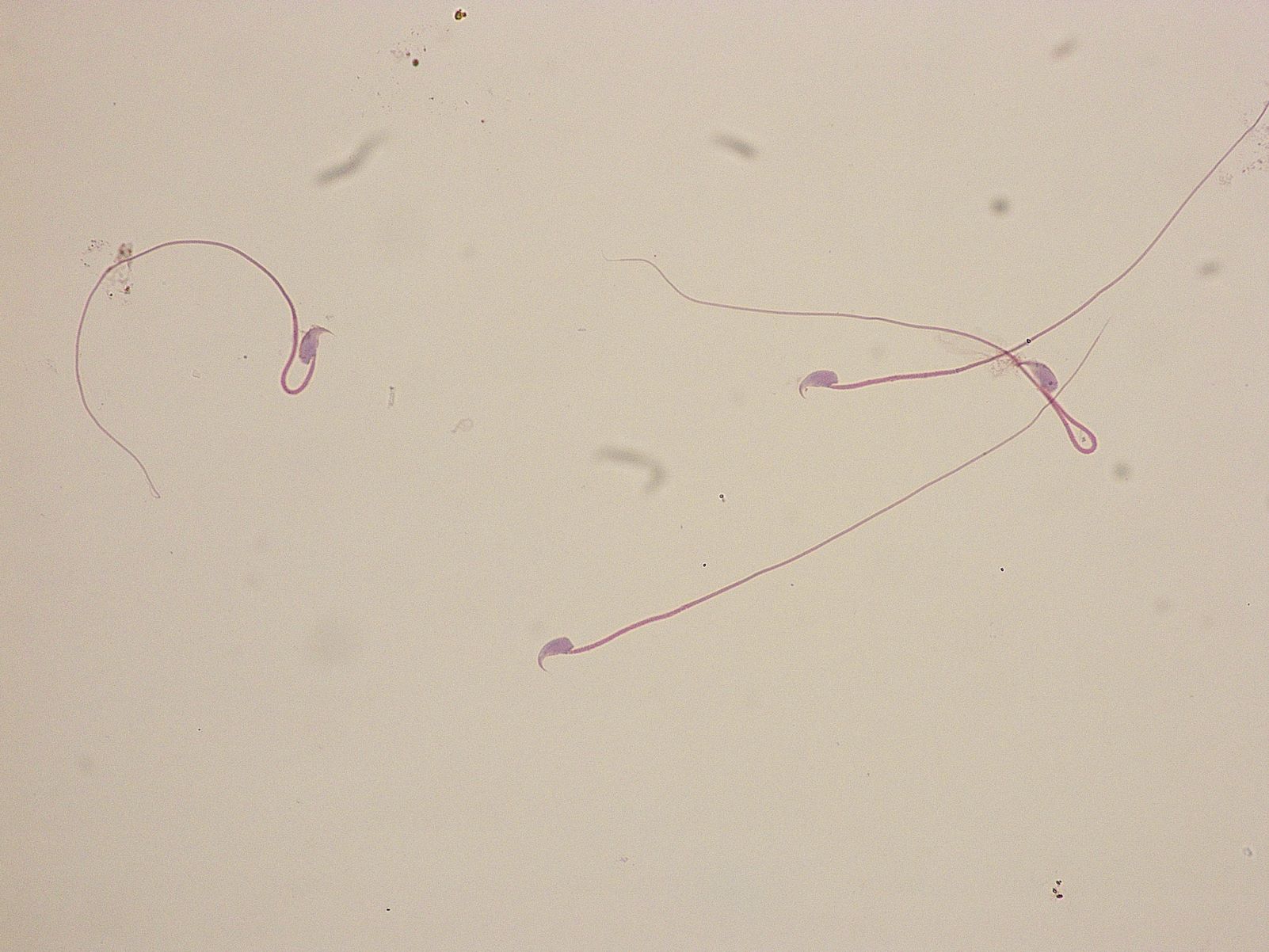

Supplement: Supplementary file 6 — Source Data for Figure 3 [file EMBR-24-e56316-s005.zip › Figure3/3A/3A_CTL.tif]

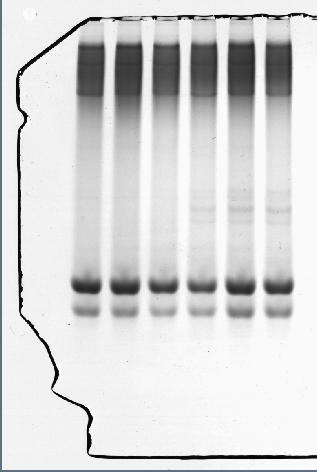

Supplement: Supplementary file 6 — Source Data for Figure 3 [file EMBR-24-e56316-s005.zip › Figure3/3G/3G_left_image.tif]

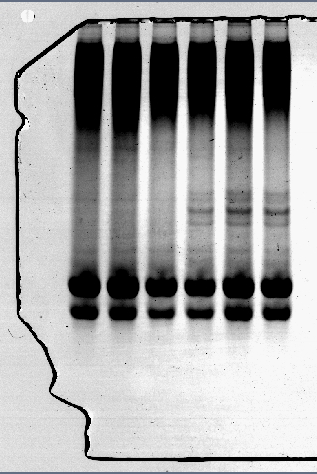

Supplement: Supplementary file 6 — Source Data for Figure 3 [file EMBR-24-e56316-s005.zip › Figure3/3G/3G_left_image_longer_exposure.tif]

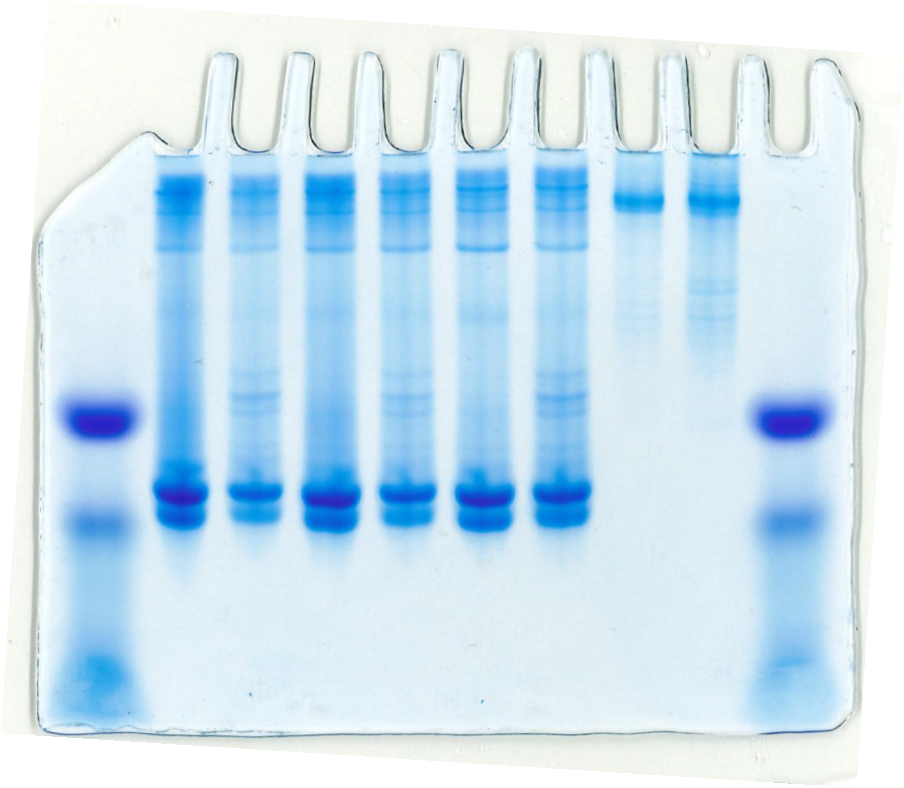

Supplement: Supplementary file 6 — Source Data for Figure 3 [file EMBR-24-e56316-s005.zip › Figure3/3G/3G_right_image.tif]
