# Supplementary material for: DOT1L regulates chromatin reorganization and gene expression during sperm differentiation
Source: EMBO Rep. 2023 Apr 26;24(6):e56316. doi: 10.15252/embr.202256316 (PMC10240200; doi:10.15252/embr.202256316)
Supplement: Supplementary file 8 [file EMBR-24-e56316-s001.pdf]

EMBO reports

DOT1L regulates chromatin reoganization and gene expression during sperm differentiation

Melina Blanco, Laila El Khattabi, Clara Gobe, Marion Crespo, Manon Coulee, Alberto de la Iglesia, Côme Ialy-Radio, Clementine Lapoujade, Maelle Givelet, Marion Delessard, Ivan Seller Corona, Kosuke Yamaguchi, Nadege Vernet, Fred van Leeuwen, Alban Lermine, Yuki Okada, Romain Daveau, Rafael Oliva, Pierre Fouchet, Ahmed Ziyyat, Delphine Pflieger, and Julie COCQUET
DOI: 10.15252/embr.202256316

Corresponding author(s): Julie COCQUET (julie.cocquet@inserm.fr)

Review Timeline:

Submission Date:	17th Oct 22
Editorial Decision:	28th Nov 22
Revision Received:	24th Feb 23
Editorial Decision:	24th Mar 23
Revision Received:	31st Mar 23
Accepted:	13th Apr 23

Editor: Achim Breiling

Transaction Report:

(Note: With the exception of the correction of typographical or spelling errors that could be a source of ambiguity, letters and reports are not edited. Depending on transfer agreements, referee reports obtained elsewhere may or may not be included in this compilation. Referee reports are anonymous unless the Referee chooses to sign their reports.)

Dear Dr. Cocquet,

Thank you for the submission of your research manuscript to EMBO reports. I have now received the reports from the three referees that were asked to evaluate your study, which can be found at the end of this email.

As you will see, the referees think that these findings are of interest. However, they have several comments, concerns, and suggestions, indicating that a major revision of the manuscript is necessary to allow publication of the study in EMBO reports. As the reports are below, and all the referee concerns need to be addressed, I will not detail them here.

Given the constructive referee comments, we would like to invite you to revise your manuscript with the understanding that all referee concerns must be addressed in the revised manuscript and in a detailed point-by-point response. Acceptance of your manuscript will depend on a positive outcome of a second round of review. It is EMBO reports policy to allow a single round of revision only and acceptance of the manuscript will therefore depend on the completeness of your responses included in the next, final version of the manuscript.

Revised manuscripts should be submitted within three months of a request for revision. Please contact me to discuss the revision (also by video chat) if you have questions or comments regarding the revision, or should you need additional time.

When submitting your revised manuscript, please also carefully review the instructions that follow below.

PLEASE NOTE THAT upon resubmission revised manuscripts are subjected to an initial quality control prior to exposition to re-review. Upon failure in the initial quality control, the manuscripts are sent back to the authors, which may lead to delays. Frequent reasons for such a failure are the lack of the data availability section (please see below) and the presence of statistics based on n=2 (the authors are then asked to present scatter plots or provide more data points).

When submitting your revised manuscript, we will require:

1) a .docx formatted version of the final manuscript text (including legends for main figures, EV figures and tables), but without the figures included. Figure legends should be compiled at the end of the manuscript text.

2) individual production quality figure files as .eps, .tif, .jpg (one file per figure), of main figures (up to 8) and EV figures. Please upload these as separate, individual files upon re-submission.

The Expanded View format, which will be displayed in the main HTML of the paper in a collapsible format, has replaced the Supplementary information. You can submit up to 5 images as Expanded View. Please follow the nomenclature Figure EV1, Figure EV2 etc. The figure legend for these should be included in the main manuscript document file in a section called Expanded View Figure Legends after the main Figure Legends section. Additional Supplementary material should be supplied as a single pdf file labeled Appendix. The Appendix should have page numbers and needs to include a table of content on the first page (with page numbers) and legends for all content. Please follow the nomenclature Appendix Figure Sx, Appendix Table Sx etc. throughout the text, and also label the figures and tables according to this nomenclature.

For more details, please refer to our guide to authors:
http://www.embopress.org/page/journal/14693178/authorguide#manuscriptpreparation

Please consult our guide for figure preparation:
http://wol-prod-cdn.literatumonline.com/pb-assets/embo-site/EMBOPress_Figure_Guidelines_061115-1561436025777.pdf

See also the guidelines for figure legend preparation:
https://www.embopress.org/page/journal/14693178/authorguide#figureformat

3) a complete author checklist, which you can download from our author guidelines (https://www.embopress.org/page/journal/14693178/authorguide). Please insert page numbers in the checklist to indicate where the requested information can be found in the manuscript. The completed author checklist will also be part of the RPF.

Please also follow our guidelines for the use of living organisms, and the respective reporting guidelines:
http://www.embopress.org/page/journal/14693178/authorguide#livingorganisms

4) that primary datasets produced in this study (e.g. RNA-seq, ChIP-seq, structural and array data) are deposited in an appropriate public database. If no primary datasets have been deposited, please also state this in a dedicated section (e.g. 'No primary datasets have been generated and deposited'), see below.

See also: http://embor.embopress.org/authorguide#datadeposition

Please remember to provide a reviewer password if the datasets are not yet public.

The accession numbers and database should be listed in a formal "Data Availability" section (placed after Materials & Methods) that follows the model below. This is now mandatory (like the COI statement). Please note that the Data Availability Section is restricted to new primary data that are part of this study. This section is mandatory. As indicated above, if no primary datasets have been deposited, please state this in this section

Data availability

The datasets produced in this study are available in the following databases:

- RNA-Seq data: Gene Expression Omnibus GSE46843 (https://www.ncbi.nlm.nih.gov/geo/query/acc.cgi?acc=GSE46843)
- [data type]: [name of the resource] [accession number/identifier/doi] ([URL or identifiers.org/DATABASE:ACCESSION])

*** Note - All links should resolve to a page where the data can be accessed. ***

Moreover, I have these editorial requests:

6) We now request the publication of original source data with the aim of making primary data more accessible and transparent to the reader. Our source data coordinator will contact you to discuss which figure panels we would need source data for and will also provide you with helpful tips on how to upload and organize the files.

7) Our journal encourages inclusion of *data citations in the reference list* to directly cite datasets that were re-used and obtained from public databases. Data citations in the article text are distinct from normal bibliographical citations and should directly link to the database records from which the data can be accessed. In the main text, data citations are formatted as follows: "Data ref: Smith et al, 2001" or "Data ref: NCBI Sequence Read Archive PRJNA342805, 2017". In the Reference list, data citations must be labeled with "[DATASET]". A data reference must provide the database name, accession number/identifiers and a resolvable link to the landing page from which the data can be accessed at the end of the reference. Further instructions are available at: http://www.embopress.org/page/journal/14693178/authorguide#referencesformat

8) Regarding data quantification and statistics, please make sure that the number "n" for how many independent experiments were performed, their nature (biological versus technical replicates), the bars and error bars (e.g. SEM, SD) and the test used to calculate p-values is indicated in the respective figure legends (also for potential EV figures and all those in the final Appendix). Please also check that all the p-values are explained in the legend, and that these fit to those shown in the figure. Please provide statistical testing where applicable. Please avoid the phrase 'independent experiment', but clearly state if these were biological or technical replicates. Please also indicate (e.g. with n.s.) if testing was performed, but the differences are not significant. In case n=2, please show the data as separate datapoints without error bars and statistics. See also: http://www.embopress.org/page/journal/14693178/authorguide#statisticalanalysis

If n<5, please show single datapoints for diagrams.

9) Please add scale bars of similar style and thickness to all the microscopic images, using clearly visible black or white bars (depending on the background). Please place these in the lower right corner of the images themselves. Please do not write on or near the bars in the image but define the size in the respective figure legend.

10) Please also note our reference format:
http://www.embopress.org/page/journal/14693178/authorguide#referencesformat

11) We updated our journal's competing interests policy in January 2022 and request authors to consider both actual and perceived competing interests. Please review the policy https://www.embopress.org/competing-interests and update your competing interests if necessary. Please name this section 'Disclosure and Competing Interests Statement' and put it after the Acknowledgements section.

12) We now use CRediT to specify the contributions of each author in the journal submission system. CRediT replaces the author contribution section. Please use the free text box to provide more detailed descriptions. See also guide to authors: https://www.embopress.org/page/journal/14693178/authorguide#authorshipguidelines

Finally, please order the manuscript sections like this, using these names:
Title page - Abstract - Keywords - Introduction - Results - Discussion - Materials and Methods - Data availability section - Acknowledgements (including funding information) - Disclosure and Competing Interests Statement - References - Figure legends - Expanded View Figure legends

I look forward to seeing a revised version of your manuscript when it is ready. Please let me know if you have questions or

comments regarding the revision.

Please use this link to submit your revision: https://embor.msubmit.net/cgi-bin/main.plex

Yours sincerely,

Achim Breiling
Senior Editor
EMBO Reports

Referee #1:

In this manuscript, Blanco et al., reported that using conditional knockout mouse model, the histone H3K79 methylansferase DOT1L is essential for gene regulation and chromatin remodeling during spematid differentiation. Authors found that Dot1l-knockout (KO) spermatozoa exhibit several defects such as thinner and distorted flagella, cytoplasmic retention and impaired nuclear compaction, which could lead to less fertility. The chromatin of Dot1l- KO spermatozoa is less compact and characterized by a higher level of retained histones, and thus authors conclude histone-to-protamine transition is impaired in Dot1l- KO. Although the characterization of the KO phenotypes is impressive, the link with molecular basis is not enough at this moment. I have comments below, which should be considered before the acceptance.

1. In Figure 3F, it is not described and thus unclear on 1) the potential cause of and 2) what is the outcome of the accumulation of immature PRM2 and in Dot1l-KO SPZ.

2. In Figure 4, H4 PTMS in ES were overall affected in DOT1L KO. More specifically speaking, the level of H4K20me1/2/3 is severely compromised. Are there any explanation or connection with DOT1L? Authors should discuss the point.

3. Relevant to above point, H4 acethylations were severely compromised in Dot1l-KO ES. Although authors discussed the potential link of Dot1L with H4Ac in the discussion, the molecular basis for the observed phenotypes is not substantial at this moment. In Line 288, authors argued that Kat2a-KO show similar phenotypes to Dot1l KO. Could KAT2a be the HAT for the H4 acethylation? Or any other potential HAT for H4 in this stage. Authors should discuss more this point.

4. The molecular relationship between H3K79me and H2B ubiquitination has been intensively discussed in earlier reports. In the case authors already performed LC-MS/MS experiments, it would be appreciated by readers if authors disclose the quantification results of PTMs of H2B and also H2A.

Referee #2:

Blanco et al. examine the role of the histone methyltransferase (HMT) Dot1l in the development of postnatal mouse male germ cells. They identify a role for this gene in germ line maintenance and post-meiotic differentiation. They show that Dot1l¬-deficient males produce significantly fewer offspring than their control siblings and that the sperm produced by these animals show defects in shape and microtubule organization. They find that during spermiogenesis (some?) Dot1l-deficient germ cells fail to undergo normal histone-to-protamine exchange, retaining higher quantities of histones and (presumably) un-processed pre-protamine, and possess altered gene expression and histone post-translational modification (PTM) levels.

Overall, the data in this paper are interesting and identify a new player in the terminal phase of mouse spermatogenesis. The wet-lab experiments are performed well, yet the lack of a connection between the described phenotypes and the direct function of Dot1l (as a chromatin modifying enzyme) severely limits the value of the findings. Further experiments, connecting the observed changes in gene expression and histone PTMs are required to make this story more complete and provide much stronger insights into the role of this gene in spermatogenesis.

Specific points:
Figure 1 and S1
1B, 1D & S1B: WB for DOT1L
What is the source of the many additional bands observed in the Western blot analysis of DOT1L, shown in Fig. S1B? Given that these lower bands are strongly decreased in the Dot1l-deficient testis, are they processed forms of DOT1L? Hence, is the representation shown in Figure 1B truly representative? The image shown in Figure S1B might be more appropriate for the main text.

If DOT1L protein is subject to degradation, does this happen in a cell type specific manner? Accordingly, please provide the entire blot for the protein analysis shown in Fig1D (as well as for any of the other WBs provided in the paper).

1C & S1D: immunohistochemistry detection of DOT1L in testicular sections
In which cell types is DOT1L protein expressed? The authors are urged to provide a more comprehensive description of the cell specificity and overall dynamics of DOT1L expression during spermatogenesis. Is it also expressed in certain spermatogonia, or Sertoli cells? What is the age of the animals shown in figure 1C? Some tubules seem to have reduced cell numbers.

Data in S1D suggest that around 11% of round spermatids possess DOT1L protein and/or H3K79me2. Did the authors genotype the pups generated after breeding? One possibility would be that the sperm that was capable of producing offspring originates from germ cells in which Stra8-Cre failed to excise the Dot1l conditional allele, leading to 50% of offspring having a paternally deleted or a floxed wildtype allele.

S1D: How many cells were counted per animal? To represent the possible variability between animals, a box plot representation showing variability between animals would be more informative than the current plot. The authors could represent the data as the number of cells per tubule per animal that are positive or negative for DOT1L expression. Please include a description of the age of animals analyzed.

Figure 2 and S2
Fig. 2B, S2C, Lines 120 and following:
please provide a more comprehensive description of the testicular pathology. What is the age of animals? The picture shows a few rather "empty tubules". Is there a spermatogonial defect? Does this phenotype become more severe upon aging?

Breeding performance of the Dot1l-deficient males: The authors show that breedings with Dot1l¬-deficient males produce pups (albeit at reduced number and frequency, Figure 2D). When performing assays for fertilization (either with or without the zona pellucida) they find that Dot1l¬-deficient sperm do not fertilize any of the oocytes (Figure 2E). How do they explain this difference? The difference should be pointed out in the text of the manuscript and discussed accordingly.

The reduction in littersizes points towards a defect either in fertilization, pre- or post-implantation development. In the natural mating experiments, what is the underlying problem? If embryos would die at some point during their development, this would be really an interesting finding and might reveal a link between faulty chromatin status in sperm and embryonic fitness. This is a critical point that needs to be addressed by the authors.

Fig S2A: what is DOT1L-KOeops?

Figure 3 and S3
3E: increased histone levels in DOT1L-KO:
This figure presents interesting and important data. Unfortunately, the current presentation of the data does not allow a straightforward interpretation of the absolute levels of histone proteins in the different samples and the (apparent) variability observed between samples. The current plot suggests that in several KO animals, there is no major change in histone levels. Correct? What is the explanation? Plotting absolute protein levels in a box plot format would be more appropriate. Moreover, a direct comparison of (absolute) H3 and TH2B levels would be informative as well, to assess whether levels of H3 and TH2B co-vary or not. Finally, the authors are requested to provide all primary WB data of all samples analyzed.

Is it possible to provide a loading control, reflecting the number of germ cell analyzed per sample?

3F: Coomassie staining AU gel for protamines:
Is the order of PRM1 and PRM2 on the gel correct?

It is not clear how the quantification and normalization of protein levels was done to measuring protamine ratios. A box plot representation of direct ratios would be easier to interpret.

How sure are the authors that the higher molecular weight proteins observed in knockout sperm are immature forms of PRM2? A Western blot analysis, using a PRM2 antibody would be more informative.

S3D: susceptibility of KO sperm to NPM treatment:
This is an interesting data set that relates to the WB data presented in 3E. How do the total levels of histones in the different samples compare between the protein lysis method used to generate the data shown in Fig 3E versus the method used to generate figure S3D? It seems as if the total amount of H3 is higher upon NPM decondensation? Or is this reflecting variation between animals?

The normalization of the supernatant to pellet ratio in control samples to "1" is not appropriate given the dramatic difference in levels between the two preparations and hence being very sensitive to mistakes in measurements. A simple quantification and representation of total levels would be more suitable.

Did the authors observe similar findings for TH2B and for example histone H4?

Figure 4 and S4
If absolute levels of histones are increased in the elongating spermatids of Dot1l-deficient animals, how is the quantitation of histone PTMs to be interpreted? H3K36me2 and H3K27me2 are relatively highly abundant marks found in inter-genic regions in somatic cells. Hence, do the changes in PTM levels simply reflect a pool of more intergenic retained histones, or do they reflect changes in the histone modification state of histones in the same regions in mutant and control spermatids? Are these PTMs already upregulated in spermatocytes and spermatids of KO animals, or only upon nuclear elongation? WB analyses on protein lysates of sorted germ cells may allow this question to be addressed.

Figure 5 and S5
Fig. 5A and Line 224: it is not clear to the reviewer whether calling differential expression has been corrected for multiple testing (adj p-value)?

The analysis of differentially expressed genes is limited. Are there any sequence features in genes mis-expressed during specific stages of spermatogenesis in the Dot1l¬-deficient testis? What are the patterns of expression of these genes in control testes? What is the chromatin status of mis-expressed genes in each of the stages profiled? While ChIP-Seq in mutant animals would be most informative, existing published data sets in wild type animals could be analyzed to further enhance the classification of these genes and understand why certain genes may be misregulated.

The lack of knowledge of where H3K79me2 and H3K79me3 are localized within the genomes of cell types examined is a particular limitation of the study. How do these marks established by Dot1l in wild type spermatocytes and round spermatids relate to their gene regulatory function?

To obtain a better mechanistic insight in the kinetics of chromatin remodeling in control and mutant spermatids, immunofluorescence analysis of several histone PTMs, as identified by MS analysis, should be quantitatively investigated during the progression of spermatid development as revealed in testicular sections. (Co-)staining for H3K79me2, H3K79me3, H4ac, H3K36me2, H3K27me2 as well as H4K20me1, me2 and me3 should be investigated. Brdt has been shown to read H4ac levels. Is there any change in the localization of the protein in Dot1l ko spermatids? What about TNP1/2 and PRM1/2 expression at the single spermatid level?

Given the increased levels of H3K36me2 and H3K27me2 in mutant elongating spermatids, performing ChIP-Seq analysis of these two marks in control and Dot1l-deficient round and elongating spermatids may be very informative as well (pending on the outcome of the requested WB analyses (see Fig. 4) and IF analysis (see above)). For example, does the increase in H3K36me2 occur because gene body H3K36me3 is decreased, or because more intergenic H3K36me2 is retained in elongating spermatids of Dot1l-deficient animals. Likewise, ChIP-Seq for H3K27me2 could provide similar interesting insights.

Line 313-316:
"The deregulation of genes associated to "chromatin function" could also contribute to the observed chromatin changes. This is exemplified by the increase in H3K27me2 detected in Dot1l-KO elongating/spermatids following the downregulation of the gene encoding the H3K27 demethylase KDM6A in round spermatids". Currently, this is just a correlation. Functional causality has not been shown and hence this sentence should be rephrased.

Line 236-237: Which Slc genes have specifically been implicated in spermatogenesis? Are these genes deregulated upon Dot1l loss of function? Please be more specific.

Referee #3:

The manuscript by Bianco and co-authors, titled "The histone methyltransferase DOT1L regulates chromatin reorganization and gene expression during the postmeiotic differentiation of male germ cells" describes defects in post-meiotic male germ cells following conditional knock-out of the Histone H3K79 methyltransferase, Dot1L, in mice.

The authors demonstrate pathological phenotype of spermatozoa that include defects in histone-protamine exchange manifested by retention of histones, distorted protamine1/protamine2 ratio and impaired nuclear compaction. In addition, the authors observe cytoplasmic retention, abnormal flagellum and structural abnormalities of the axonemes resulting in impairment of sperm motility and inability of Dot1lKO spermatozoa to deliver proper fertilization of the oocyte.

Next, the authors explore the effect of Dot1L knockdown on the epigenetic landscape of post-meiotic spermatids and their gene expression. They show, via elegant masspectrometry studies, that decreased H3K79me levels have a profound effect on overall H4 Acetylation (downregulation) and H3 modifications (upregulation). The gene expression profile of three germ cell types show the deregulation of a large number of genes, including a family of Slc genes, responsible for sperm motility and flagella differentiation, and downregulation of H3K27 demethylase KDM6A.

While this study has a largely descriptive nature, it provides an important and significant Dot1l KO model to investigate Dot1L

function in its non-pathological physiological environment. As a result, a number of novel insights into its function during spermiogenesis have been described. While the study has minimal attempt to investigate the mechanistic aspects, the brief but in-depth discussion offers excellent explanations for the observed phenotypes and convincing interpretations of the results. Another strong side of this manuscript is an abundance of good-quality data that largely supports and justifies the conclusions.

Minor changes:

i. Please include a DAPI-stained or H&E-stained panel in Fig 1C to better highlight the morphology and lack of cell types in the Dot1L KO seminiferous tubules. At present, the panel depicting the KO is not very clear without nuclear staining, making it hard to compare to the wt.
ii. Please include for clarity horizontal bar and * to indicate significant difference in H3 and H4 PTMs in wt and Dot1LKO in Fig 4 and Fig S4A
iii. The increased TUNEL staining for some ES cells of Dot1lKO in Fig 3D may not indicate increased apoptosis of these cell type but rather marks ES with retained cytoplasm. It has been demonstrated earlier that shading of the cytoplasm in the form of Residual Bodies regulated via cytoplasm-restricted apoptotic signaling in the early ES cells, so if the cytoplasm is retained, so are the apoptotic markers. (For references, please see Blanco-Rodriguez, J. & Martinez-Garcia, C. Apoptosis is physiologically restricted to a specialized cytoplasmic compartment in rat spermatids. Biol Reprod 61, 1541-1547 (1999))

Dear editor,

We have completed the revision of our manuscript. As you will see in the point-by-point response below, we have performed additional experiments and have modified our manuscript and figures to address the reviewers' comments. We hope the revised version of our manuscript will be accepted for publication in EMBO Reports.

We want to thank the Reviewers for their constructive evaluation of our manuscript which helped us improving the presentation and interpretation of our data. We have now thoroughly revised the manuscript based on their suggestions and requests. Point-to-point responses to Reviewer comments can be found below (in blue).

Referee #1:

In this manuscript, Blanco et al., reported that using conditional knockout mouse model, the histone H3K79 methylansferase DOT1L is essential for gene regulation and chromatin remodeling during spematid differentiation. Authors found that Dot1l-knockout (KO) spermatozoa exhibit several defects such as thinner and distorted flagella, cytoplasmic retention and impaired nuclear compaction, which could lead to less fertility. The chromatin of Dot1l-KO spermatozoa is less compact and characterized by a higher level of retained histones, and thus authors conclude histone-to-protamine transition is impaired in Dot1l- KO. Although the characterization of the KO phenotypes is impressive, the link with molecular basis is not enough at this moment. I have comments below, which should be considered before the acceptance.

1. In Figure 3F, it is not described and thus unclear on 1) the potential cause of and 2) what is the outcome of the accumulation of immature PRM2 and in Dot1l-KO SPZ.

Even though the exact underlying molecular mechanisms have not been elucidated so far, depletion of some of the proteins driving the histone-to-protamine transition (i.e. histone variants, transition proteins) has been shown to lead to accumulation of unprocessed PRM2 in spermatozoa (Barral *et al*, 2017; Luense *et al*, 2019; Yamauchi *et al*, 2010; Yu *et al*, 2000), suggesting a link between proper histone displacement and a correct protamine processing. Also, depletion of the cleaved domain of the protamine 2 precursor leads to impaired histone variants and transition protein levels (Arevalo *et al*, 2022). The relation between accumulation of pre-PRM2 and increased PRM1/PRM2 has also been described in infertile patients (de Mateo *et al*, 2009; Torregrosa *et al*, 2006). This information has been included in the discussion (page 11, lines 341-346).

2. In Figure 4, H4 PTMS in ES were overall affected in DOT1L KO. More specifically speaking, the level of H4K20me1/2/3 is severely compromised. Are there any explanation or connection with DOT1L? Authors should discuss the point.

Our findings are in agreement with the observation made by Jones et al. in 2008 (Jones *et al*, 2008) since they found that H4K20 tri-methylation level is reduced at centromeres and telomeres of *Dot1l*-KO embryonic stem cells. H3K9 di-methylation (but not tri-methylation) was also found reduced in KO embryonic stem cells, but this histone PTM could not be detected in our analyses. By nano LC-MS/MS, Luense et al. have observed that H4K20me3 was the

most dynamic H4K20 modification during spermatogenesis, with a peak towards the end of spermiogenesis, when histones are replaced by protamines.
Interestingly, Ho et al have found that SET8 H4K20me1 methyltransferase and DOT1L (which is a non-SET domain methyltransferase) bind to the nucleosomal acidic patch by a similar mechanism (Ho *et al*, 2021). Yet, the mechanism by which DOTL1 and/or H3K79 methylation influences H4K20 methylation remains unclear. We have added this information in the discussion (lines 367-383).

3. Relevant to above point, H4 acethylations were severely compromised in Dot1l-KO ES. Although authors discussed the potential link of Dot1L with H4Ac in the discussion, the molecular basis for the observed phenotypes is not substantial at this moment. In Line 288, authors argued that Kat2a-KO show similar phenotypes to Dot1l KO. Could KAT2a be the HAT for the H4 acethylation? Or any other potential HAT for H4 in this stage. Authors should discuss more this point.

Kat2a (*Gcn5*) has been shown to acetylate H4K5, K8, K12, and K16 (Kuo *et al*, 1996) as well as H3K9 and K14 (Bonnet *et al*, 2014; Grant *et al*, 1999). In their article, Luense et al have shown that KAT2A promotes histone hyperacetylation and nucleosome eviction during spermiogenesis (Luense *et al.*, 2019). We have cited this work in our discussion because *Kat2a*-knockout in male germ cells results in incomplete histone-to-protamine transition characterized by higher level of retained histones, as observed in *Dot1l*-KO.
Kat2a expression is not affected in *Dot1l*-KO spermatids. So we do not think that the decrease in H4 acetylation is a consequence of the downregulation of Kat2a (or of any known HAT). Our model is that H4 acetylation is modified as a consequence of the loss of H3K79 methylation, as indicated by several studies showing a cross talk between these 2 marks.
We have tried to clarify this point in the discussion (lines 352-366).

4. The molecular relationship between H3K79me and H2B ubiquitination has been intensively discussed in earlier reports. In the case authors already performed LC-MS/MS experiments, it would be appreciated by readers if authors disclose the quantification results of PTMs of H2B and also H2A.

In the revised version of our manuscript, we have added the quantification of H2A and H2B PTMs performed in round and elongating spermatids (Appendix FigS4B). A few PTMs were found significantly modified in KO vs CTL elongating spermatids, such as H2AR88me1, TH2BK6ac and TH2BK35ac. Some ubiquitinated residues could be identified, namely K35, K44/47 and K109 from TH2B, via the identification of peptides bearing a lysine modified with GlyGly. They are not presented in the histograms, because they were not sufficiently reproducibly quantified. H2BK120ub could not be identified; of note, the tryptic peptide AVTKYTSSK containing Lys120 was identified in a non-modified form, and in acetylated form, yet with a low identification score and with quantification in only 3 samples for the latter peptide form. We also provide all the quantitative data resulting from LC-MS/MS analyses as source data.

Referee #2:

Blanco et al. examine the role of the histone methyltransferase (HMT) Dot1l in the development of postnatal mouse male germ cells. They identify a role for this gene in germ line maintenance and post-meiotic differentiation. They show that Dot1l¬-deficient males produce significantly fewer offspring than their control siblings and that the sperm produced by these animals show defects in shape and microtubule organization. They find that during spermiogenesis (some?) Dot1l-deficient germ cells fail to undergo normal histone-to-protamine exchange, retaining higher quantities of histones and (presumably) un-processed pre-protamine, and possess altered gene expression and histone post-translational modification (PTM) levels.

Overall, the data in this paper are interesting and identify a new player in the terminal phase of mouse spermatogenesis. The wet-lab experiments are performed well, yet the lack of a connection between the described phenotypes and the direct function of Dot1l (as a chromatin modifying enzyme) severely limits the value of the findings. Further experiments, connecting the observed changes in gene expression and histone PTMs are required to make this story more complete and provide much stronger insights into the role of this gene in spermatogenesis.

Specific points:

Figure 1 and S1,1B, 1D & S1B: WB for DOT1L
What is the source of the many additional bands observed in the Western blot analysis of DOT1L, shown in Fig. S1B? Given that these lower bands are strongly decreased in the Dot1l-deficient testis, are they processed forms of DOT1L? Hence, is the representation shown in Figure 1B truly representative? The image shown in Figure S1B might be more appropriate for the main text. If DOT1L protein is subject to degradation, does this happen in a cell type specific manner? Accordingly, please provide the entire blot for the protein analysis shown in Fig1D (as well as for any of the other WBs provided in the paper).

Additional bands are specific (since they decrease in KO samples) and are most likely DOT1L proteoforms since several of them have the same molecular weight as the proteoforms described in UniProt (see lines 114-118 of the revised version of our manuscript, and information added in the legend of Appendix FigS1E). Those smaller size forms are only visible in case of long exposure since they are much less abundant than the canonical (~165 kDa) and the testis-specific (~185 kDa) proteoforms. Except for this latter isoform (characterized in (Dottermusch-Heidel *et al*, 2014)), other proteoforms are not cell-type specific. We have added the entire Fig1D blot detected with anti-DOT1L antibody in Appendix FigS1I. We also provide all source (uncropped) images in separate files, as requested by *EMBO Reports*.

1C & S1D: immunohistochemistry detection of DOT1L in testicular sections
In which cell types is DOT1L protein expressed? The authors are urged to provide a more comprehensive description of the cell specificity and overall dynamics of DOT1L expression during spermatogenesis. Is it also expressed in certain spermatogonia, or Sertoli cells?
In Appendix FigS1B, we have added a figure showing *Dot1l* mRNA expression pattern using single cell RNA-seq data from (Green *et al*, 2018). We also show a schematic diagram recapitulating what is known of DOT1L protein expression in testis based on the literature and from our observations (Appendix FigS1C). We have also added information in the manuscript (lines 97-98).

What is the age of the animals shown in figure 1C? Some tubules seem to have reduced cell numbers.
All males were analyzed at adult age, between 3 and 5 months old. We have added this

information in the material & method section (line 409). “all experiments were performed on (adult) 3 to 5 month-old males.” Indeed, some tubules have reduced cell numbers. This is in line with the reduced number of all germ cell population observed in Figures 2B and 2C, as well as what has been found by Lin et al (Lin *et al*, 2022).

Data in S1D suggest that around 11% of round spermatids possess DOT1L protein and/or H3K79me2. Did the authors genotype the pups generated after breeding? One possibility would be that the sperm that was capable of producing offspring originates from germ cells in which Stra8-Cre failed to excise the Dot1l conditional allele, leading to 50% of offspring having a paternally deleted or a floxed wildtype allele.

Thank you for this question. Yes, we have genotyped the pups born from KO males (mated with WT females) and found transmission of the floxed allele in 35% of babies and of the deleted allele in 65% of them. This indicates that the deleted allele is transmitted but less efficiently than what we could have expected based on our IF quantification (i.e. 65% vs. >85% of spermatids without DOT1L signal).We have added this information in the text (lines 132-136).

S1D: How many cells were counted per animal? To represent the possible variability between animals, a box plot representation showing variability between animals would be more informative than the current plot. The authors could represent the data as the number of cells per tubule per animal that are positive or negative for DOT1L expression. Please include a description of the age of animals analyzed.

A total of 5 CTL, 3 HET and 6 KO males were analyzed. All round spermatids (RS) in 6 to 10 tubules per animal were counted (RS were identified based on DAPI staining).This represents between 200 and 400 RS per animal. To evaluate the efficiency of DOT1L KO, the number of RS with a visible DOT1L signal was recorded. We have modified the corresponding figure (now Appendix FigS1G) to show individual animal values (% of RS with DOT1L signal per animal). The males which were analyzed were all between 2.5 and 4 months old. This information was added in Appendix FigS1G legend.

Figure 2 and S2

Fig. 2B, S2C, Lines 120 and following:

please provide a more comprehensive description of the testicular pathology. What is the age of animals? The picture shows a few rather "empty tubules". Is there a spermatogonial defect? Does this phenotype become more severe upon aging?

All males were analyzed at adult age, between 3 and 5 months old. We have added this information in the material & method section (line 409). The reduction in cell number is in line with the sequential loss of germ cells from spermatogonia stage which has been described by Lin et al and which gets worse with age (Lin *et al.*, 2022). We have clarified this information in our discussion from line 300.

Breeding performance of the Dot1l-deficient males: The authors show that breedings with Dot1l¬-deficient males produce pups (albeit at reduced number and frequency, Figure 2D). When performing assays for fertilization (either with or without the zona pellucida) they find that Dot1l¬-deficient sperm do not fertilize any of the oocytes (Figure 2E). How do they explain this difference? The difference should be pointed out in the text of the manuscript and discussed accordingly.

This difference could be explained by the optimal conditions and the high efficiency of in vivo fertilization compared to in vitro fertilization. Therefore, IVF can better reveal even minor

functional alterations. If evidence was needed, the difference of the number of sperm needed to fertilize oocytes in vivo or in vitro is very large. Indeed, while it is estimated that the 100 to 200 sperm that succeed to reach the ampullae in vivo are sufficient to fertilize all the oocytes, even after superovulation (between 20 and 30 per mouse), one million sperm per ml (10^5 in 100 µL of medium drop) are necessary for a poorer rate of in vitro fertilization of a similar oocyte pool. Such differences have already been observed by us (Barraud-Lange *et al*, 2020; Gadadhar *et al*, 2021) and by others (Fujihara *et al*, 2010; Kawano *et al*, 2010). We are discussing this point in the revised version of our manuscript (lines 150-153).

The reduction in littersizes points towards a defect either in fertilization, pre- or post-implantation development. In the natural mating experiments, what is the underlying problem? If embryos would die at some point during their development, this would be really an interesting finding and might reveal a link between faulty chromatin status in sperm and embryonic fitness. This is a critical point that needs to be addressed by the authors. Look at embryo count just after in vivo fertilization.
Thank you for this very relevant point. We have carried out a series of experiments to differentiate between a fertilization and a developmental defect. In brief, superovulated females were crossed with WT males or with *Dot1l* KO males and their oocytes collected the following day. As now shown in Figure 2E, these experiments confirm a fertilization defect (rather than a pre or post implantation developmental defect) in line with the observed reduction in litter size (see also lines 137-143).

Fig S2A: what is DOT1L-KOeops?
"Eops" means spf in French. We apologize for this oversight which has been corrected in Appendix FigS2A (previously called S2A).

Figure 3 and S3
3E: increased histone levels in DOT1L-KO:
This figure presents interesting and important data. Unfortunately, the current presentation of the data does not allow a straightforward interpretation of the absolute levels of histone proteins in the different samples and the (apparent) variability observed between samples. The current plot suggests that in several KO animals, there is no major change in histone levels. Correct? What is the explanation? Plotting absolute protein levels in a box plot format would be more appropriate. Moreover, a direct comparison of (absolute) H3 and TH2B levels would be informative as well, to assess whether levels of H3 and TH2B co-vary or not. Finally, the authors are requested to provide all primary WB data of all samples analyzed.
Indeed, we can see variability among KO animals which is correlated with the heterogeneity in *Dot1l*-KO other phenotypes (for instance testis weight or *in vivo* fertilization rate, see Fig 2). We also see a variability among CTL which we cannot explain. Quantifications were performed in 3 independent experiments (technical and biological replicates), each including at least 3 CTL samples for normalization, therefore plotting raw data would not allow us to compare between all 11 CTL and 10 KO samples loaded in those 3 experiments. We now provide source images of all our western blots, with sample names which allow to compare H3 and TH2B levels in the same samples. The graphic representation of these paired comparisons is shown in Appendix FigS3D, and indicates that, indeed, H3 and TH2B levels co-vary in CTL and KO samples.
In parallel of these experiments we also quantified the transition protein TNP2 in the same extracts and observed a striking retention of TNP2 in KO spermatozoa, confirming a defective remodeling of spermatozoa (in Figure 3F).

Is it possible to provide a loading control, reflecting the number of germ cell analyzed per sample?
The chromatin of spermatozoa is very compact and more difficult to access than that of other cells. To perform reliable westernblot quantification of sperm chromatin proteins, we used a dedicated extraction protocol which specifically extracts histones (acid extraction protocol, a common practice in the field). This is not compatible with the detection of non-histone proteins. NB. Other studies using the same protocol also do not normalize their western blots with an internal loading control, see for instance (Luense *et al.*, 2019).
Nevertheless, following transfer and staining of the membranes with Ponceau stain, we can see a nonspecific band which allows us to check for comparative loading between samples. We do not want to normalize our histone detection with this band because we do not know what it is. This nonspecific band can be faintly seen in the TH2B image we now show in Fig 3E. Finally, we provide in Appendix FigS3C the uncropped westernblot and Ponceau staining images shown in Fig 3E.

3F: Coomassie staining AU gel for protamines:
Is the order of PRM1 and PRM2 on the gel correct?
Yes, as shown in previous publications by us and others (Arevalo *et al.*, 2022; Oliva, 2006; Zatecka *et al*, 2014). Due to their migration properties, mouse protamine 2 migrates slower than protamine 1 in acid urea polyacrylamide gel electrophoresis (AU-PAGE) and therefore is visualized above PRM1, similarly as in other mammalian species, and contrary to what happens in humans and primates (Corzett *et al*, 2002). We confirmed it by western blot detection using anti-PRM2 antibody (shown in Fig 3G of the revised manuscript).

It is not clear how the quantification and normalization of protein levels was done to measuring protamine ratios. A box plot representation of direct ratios would be easier to interpret.

After optic density quantification of the bands corresponding to PRM1 and PRM2, the ratios were normalized against the mean value of the control group. We have added this information in the material & method section (page 15, lines 540-543) and in the figure legend. We have added in Appendix FigS3F a box plot of direct (raw) ratios which gives the same result.

How sure are the authors that the higher molecular weight proteins observed in knockout sperm are immature forms of PRM2? A Western blot analysis, using a PRM2 antibody would be more informative.
Following this comment, we performed western blot detection using anti-PRM2 antibody, and confirmed that at least one of the higher molecular weight proteins observed in KO sperm is an immature form of PRM2. This novel data is shown in Fig 3G of the revised manuscript. Even though other bands could not be detected (probably because less abundant), we think they also very likely correspond to immature forms of PRM2. This interpretation is based on i) the gel migration pattern, ii) extensive detection of unprocessed PRM2 by others (Arevalo *et al.*; Rezaei-Gazik *et al*, 2022), and iii) our previous experience of protamine proteoforms (de Mateo *et al.*, 2009; Soler-Ventura *et al*, 2020; Torregrosa *et al.*, 2006). We have modified figure 3 legend accordingly.

S3D: susceptibility of KO sperm to NPM treatment:
This is an interesting data set that relates to the WB data presented in 3E. How do the total levels of histones in the different samples compare between the protein lysis method used to generate the data shown in Fig 3E versus the method used to generate figure S3D? It seems as

if the total amount of H3 is higher upon NPM decondensation? Or is this reflecting variation between animals?
The normalization of the supernatant to pellet ratio in control samples to "1" is not appropriate given the dramatic difference in levels between the two preparations and hence being very sensitive to mistakes in measurements. A simple quantification and representation of total levels would be more suitable.
Did the authors observe similar findings for TH2B and for example histone H4?

For the quantification of histones (H3 and TH2B) shown in Figure 3E we have used a dedicated protocol consisting first in incubating spermatozoa in DTT then in H2S04 to fully extract histones from KO and CTL samples. This protocol is expected to ensure a complete extraction of histones; it is therefore more “efficient” than the one used for NPM experiments (previously Figure S3D now Appendix FigS3B) and more appropriate to quantify histone retention in sperm.
With NPM experiments, we illustrate that KO spermatozoa are less compact because easier to de-condense than CTL ones. Indeed, following nucleoplasmin treatment (then fixation and sonication), samples are centrifuged and separated in two fractions: the supernatant contains histones from the de-compacted chromatin while the pellet contains histones from tightly compact chromatin which is “resistant” to the NPM treatment we used. Accordingly, the increased level of histones in the supernatant of *Dot1l*-KO sperm reflects the fact that their chromatin is easier to solubilize than CTL sperm chromatin.
Figure 3E and Appendix FigS3B are therefore complementary, showing histone retention and chromatin decompaction in KO sperm, respectively. We have added more precision regarding NPM interpretation in Appendix FigS3B legend.
We agree with the reviewer, and we now show (in Appendix FigS3B) the ratio obtained from raw values, in the 3 experiments we performed (E1, E2 and E3 which are technical and biological replicates). We did not try to detect with TH2B or H4 but we expect to see similar patterns using different anti histone antibodies as observed for Fig 3E (and shown in Appendix FigS3F).

Figure 4 and S4
If absolute levels of histones are increased in the elongating spermatids of Dot1l-deficient animals, how is the quantitation of histone PTMs to be interpreted?
Histone PTMs were quantified between conditions while normalizing raw MS measurements made on individual modified peptides from a given histone by the sum of MS signals detected on all tryptic peptides from this histone. In other words, comparisons were made at constant amount of the considered histone in the various samples analyzed by LC-MS/MS. Quantification of variants was performed by normalizing to the total amount of histone H4. So in both cases, the retention of histones in KO samples should not affect our results. We have clarified this point in the material & method as well as in the figure legend.

H3K36me2 and H3K27me2 are relatively highly abundant marks found in inter-genic regions in somatic cells. Hence, do the changes in PTM levels simply reflect a pool of more intergenic retained histones, or do they reflect changes in the histone modification state of histones in the same regions in mutant and control spermatids?
Are these PTMs already upregulated in spermatocytes and spermatids of KO animals, or only upon nuclear elongation? WB analyses on protein lysates of sorted germ cells may allow this question to be addressed.

To address this very relevant question, we have quantified histone PTMs and variants in round spermatids (before spermatid elongation) using LC-MS/MS quantification, which is more precise and reliable than western blot quantification. These data are now presented in EV4 and Appendix FigS4.
Importantly, using more appropriate statistical tests than previously (i.e. performing t-tests corrected for multiple analyses), we find that the "trends" observed for H3K36me2 and H3K27me2 are not significant. We have modified our text accordingly. The striking and significant changes observed in ES are decreases in H4 hyperacetylation and in H4K20 methylation levels. Those quantitative changes are not found at an earlier stage, in round spermatids. We now show bar plots and individual values as well as significant differences with a star in Figures 4, EV4, and in Appendix FigS4.

Figure 5 and S5
Fig. 5A and Line 224: it is not clear to the reviewer whether calling differential expression has been corrected for multiple testing (adj p-value)?

Thank you for this comment. The deregulated genes we showed in the initial version of our manuscript were identified based on a p-value <0.05. NB. on this list of deregulated genes, the FDR was calculated *a posteriori* using Benjamini-Hochberg correction (Benjamini & Hochberg, 1995) and was confirmed to be < 5%. This analysis is called "p-value" analysis in the revised version of our manuscript.
Now, we also present a more stringent (unsupervised) differential expression analysis, in which deregulated genes are selected using FDR < 5% (adjusted p-values). This analysis is called "FDR" analysis in the revised version of our manuscript. We think both approaches provide complementary information. Interestingly, deregulated genes obtained with these two methods have the same characteristics: downregulated genes are enriched in H3K79me2 mark, while upregulated genes are devoid of H3K79me2.
We have added all these information in the results section (lines 247-250 and lines 254-264) and in Figs 5B-C and Appendix FigS5A,B. Detailed information can be found in the material & methods, lines 615-618: "Two analyses were performed in parallel: one selecting the deregulated genes with a FDR < 5% (adjusted p-values), and one selecting deregulated genes with a p-value < 5% (non-adjusted p-values). On this second category of genes, the FDR was calculated *a posteriori* using Benjamini-Hochberg correction (Benjamini & Hochberg, 1995) and was confirmed to be < 5%."

The analysis of differentially expressed genes is limited. Are there any sequence features in genes mis-expressed during specific stages of spermatogenesis in the Dot1l¬-deficient testis? What are the patterns of expression of these genes in control testes? What is the chromatin status of mis-expressed genes in each of the stages profiled?
While ChIP-Seq in mutant animals would be most informative, existing published data sets in wild type animals could be analyzed to further enhance the classification of these genes and understand why certain genes may be misregulated.
The lack of knowledge of where H3K79me2 and H3K79me3 are localized within the genomes of cell types examined is a particular limitation of the study. How do these marks established by Dot1l in wild type spermatocytes and round spermatids relate to their gene regulatory function.

Thank you for these interesting questions, we have compared deregulated genes found in Dot1l-KO spermatids with H3K79me2 ChIP-seq data that we generated. In the revised version of our manuscript, we now show that downregulated genes are enriched in H3K79me2 at their gene body, while upregulated genes are not, suggesting that downregulated genes in the KO are direct DOT1L targets while upregulated genes are indirect targets. This is in agreement with the results obtained in *Dot1l*-KO B cells and cardiomyocytes by others (Aslam *et al*, 2021; Cattaneo *et al*, 2022). These novel analyses are presented lines 254-264, in Figs 5C-D and Appendix FigS5B.
To further investigate the sequence features of deregulated genes, we also performed a motif analysis using HOMER and identified an enrichment of BCL6 binding site in some of the upregulated genes. Like other upregulated genes, most of them are devoid of H3K79me2 in wild type spermatids and may not be directly regulated by DOT1L. In contrast, *Bcl6* and many other downregulated genes in *Dot1l*-KO spermatids are marked by H3K79me2 in wild-type cells and are likely directly regulated by DOT1L. We present these novel data in the revised version of our manuscript lines 279-285 and Appendix FigS5D. All these points are also discussed lines 317-324.

To obtain a better mechanistic insight in the kinetics of chromatin remodeling in control and mutant spermatids, immunofluorescence analysis of several histone PTMs, as identified by MS analysis, should be quantitatively investigated during the progression of spermatid development as revealed in testicular sections. (Co-)staining for H3K79me2, H3K79me3, H4ac, H3K36me2, H3K27me2 as well as H4K20me1, me2 and me3 should be investigated. Brdt has been shown to read H4ac levels. Is there any change in the localization of the protein in Dot1l ko spermatids? What about TNP1/2 and PRM1/2 expression at the single spermatid level?
We did not investigate BRDT in our model because a previous study has shown that a dramatic loss of H4 acetylation in elongating spermatids (following *Nut*-KO) did not modify BRDT level (Shiota *et al*, 2018).
To investigate the kinetics of chromatin remodeling we performed LC-MS/MS quantification in round spermatids and observed that the changes in H4 acetylation and H4K20 methylation are specific of the elongating spermatid stage. We also performed immunofluorescence detection of acH4 and TNP2 and observed qualitative changes: a decreased acH4 intensity in elongating spermatids, and a stronger TNP2 signal in some step 16 condensed spermatids. These novel data are presented in Figs EV3, EV4 and in Appendix FigS3E and S4 of the revised version of our manuscript.

Given the increased levels of H3K36me2 and H3K27me2 in mutant elongating spermatids, performing ChIP-Seq analysis of these two marks in control and Dot1l-deficient round and elongating spermatids may be very informative as well (pending on the outcome of the requested WB analyses (see Fig. 4) and IF analysis (see above)). For example, does the increase in H3K36me2 occur because gene body H3K36me3 is decreased, or because more intergenic H3K36me2 is retained in elongating spermatids of Dot1l-deficient animals. Likewise, ChIP-Seq for H3K27me2 could provide similar interesting insights.

We have carefully reviewed and integrated all our PTM quantification data obtained from 3 stages (round spermatids, elongating/condensing spermatids and spermatozoa). Systematic statistical analyses (using t-tests corrected for multiple analyses) confirm the strong decrease in H4 hyperacetylation and in H4K20 methylation in ES but H3K36 methylation and H3K27 methylation are not significantly changed at this stage nor at any other. We therefore do not think it is relevant to further investigate these marks. We have modified our text and removed the parts discussing H3K36me and H3K27me.

Line 313-316:
"The deregulation of genes associated to "chromatin function" could also contribute to the observed chromatin changes. This is exemplified by the increase in H3K27me2 detected in Dot1l-KO elongating/spermatids following the downregulation of the gene encoding the H3K27 demethylase KDM6A in round spermatids". Currently, this is just a correlation. Functional causality has not been shown and hence this sentence should be rephrased.

As said above we have removed the parts in which H3K36 methylation and H3K27 methylation were discussed.

Line 236-237: Which Slc genes have specifically been implicated in spermatogenesis? Are these genes deregulated upon Dot1l loss of function? Please be more specific.
We have added more information in the discussion line 326. “. Several members of this family such as *Slc22a14* and *Slc26a8* have previously been implicated in sperm motility *via* their effects on flagellar differentiation and/or sperm energy production …”

Referee #3:

The manuscript by Bianco and co-authors, titled "The histone methyltransferase DOT1L regulates chromatin reorganization and gene expression during the postmeiotic differentiation of male germ cells" describes defects in post-meiotic male germ cells following conditional knock-out of the Histone H3K79 methyltransferase, Dot1L, in mice.

The authors demonstrate pathological phenotype of spermatozoa that include defects in histone-protamine exchange manifested by retention of histones, distorted protamine1/protamine2 ratio and impaired nuclear compaction. In addition, the authors observe cytoplasmic retention, abnormal flagellum and structural abnormalities of the axonemes resulting in impairment of sperm motility and inability of Dot1lKO spermatozoa to deliver proper fertilization of the oocyte.

Next, the authors explore the effect of Dot1L knockdown on the epigenetic landscape of post-meiotic spermatids and their gene expression. They show, via elegant masspectrometry studies, that decreased H3K79me levels have a profound effect on overall H4 Acetylation (downregulation) and H3 modifications (upregulation). The gene expression profile of three germ cell types show the deregulation of a large number of genes, including a family of Slc genes, responsible for sperm motility and flagella differentiation, and downregulation of H3K27 demethylase KDM6A.

While this study has a largely descriptive nature, it provides an important and significant Dot1l KO model to investigate Dot1L function in its non-pathological physiological environment. As a result, a number of novel insights into its function during spermiogenesis have been described. While the study has minimal attempt to investigate the mechanistic aspects, the brief but in-depth discussion offers excellent explanations for the observed phenotypes and convincing interpretations of the results. Another strong side of this manuscript is an abundance of good-quality data that largely supports and justifies the conclusions.

Minor changes:

i. Please include a DAPI-stained or H&E-stained panel in Fig 1C to better highlight the morphology and lack of cell types in the Dot1L KO seminiferous tubules. At present, the panel depicting the KO is not very clear without nuclear staining, making it hard to compare to the wt.
We have added a counterstained IHC image in Appendix FigS1F and annotated the figure to help the readers recognize the different cell types. We have added images of immunofluorescence detection of H3K79me2 in CTL and KO testis sections (Fig EV1). We are also showing an image of testicular tubules stained with hematoxylin & Eosin in Fig2B to show which cell types are missing.

ii. Please include for clarity horizontal bar and * to indicate significant difference in H3 and H4 PTMs in wt and Dot1LKO in Fig 4 and Fig S4A
We now show bar plots and individual values as well as significant differences with a star in Figures 4, EV4 and Appendix FigS4.

iii. The increased TUNEL staining for some ES cells of Dot1lKO in Fig 3D may not indicate increased apoptosis of these cell type but rather marks ES with retained cytoplasm. It has been demonstrated earlier that shading of the cytoplasm in the form of Residual Bodies regulated via cytoplasm-restricted apoptotic signaling in the early ES cells, so if the cytoplasm is retained, so are the apoptotic markers. (For references, please see Blanco-Rodriguez, J. & Martinez-Garcia, C. Apoptosis is physiologically restricted to a specialized cytoplasmic compartment in rat spermatids. Biol Reprod 61, 1541-1547 (1999))
Thank you for this interesting information. TUNEL detects DNA fragmentation and indeed the green fluorescent staining we detect co-localizes with the nucleus of elongating spermatids (visualized in blue by DAPI). We therefore do not think that the increased TUNEL signal we see is due to cytoplasmic retention.

+++

References

Arevalo L, Merges GE, Schneider S, Oben FE, Neumann IS, Schorle H (2022) Loss of the cleaved-protamine 2 domain leads to incomplete histone-to-protamine exchange and infertility in mice. *PLoS Genet* 18: e1010272

Aslam MA, Alemdehy MF, Kwesi-Maliepaard EM, Muhaimin FI, Caganova M, Pardieck IN, van den Brand T, van Welsem T, de Rink I, Song JY *et al* (2021) Histone methyltransferase DOT1L controls state-specific identity during B cell differentiation. *EMBO Rep* 22: e51184

Barral S, Morozumi Y, Tanaka H, Montellier E, Govin J, de Dieuleveult M, Charbonnier G, Coute Y, Puthier D, Buchou T *et al* (2017) Histone Variant H2A.L.2 Guides Transition Protein-Dependent Protamine Assembly in Male Germ Cells. *Mol Cell* 66: 89-101 e108

Barraud-Lange V, Ialy-Radio C, Chalas C, Holtzmann I, Wolf JP, Barbaux S, Ziyyat A (2020) Partial Sperm beta1 Integrin Subunit Deletion Proves its Involvement in Mouse Gamete Adhesion/Fusion. *Int J Mol Sci* 21

Benjamini Y, Hochberg Y (1995) Controlling the False Discovery Rate: A Practical and Powerful Approach to Multiple Testing. *Journal of the Royal Statistical Society: Series B (Methodological)* 57: 289-300

Bonnet J, Wang CY, Baptista T, Vincent SD, Hsiao WC, Stierle M, Kao CF, Tora L, Devys D (2014) The SAGA coactivator complex acts on the whole transcribed genome and is required for RNA polymerase II transcription. *Genes & development* 28: 1999-2012

Cattaneo P, Hayes MGB, Baumgarten N, Hecker D, Peruzzo S, Aslan GS, Kunderfranco P, Larcher V, Zhang L, Contu R *et al* (2022) DOT1L regulates chamber-specific transcriptional networks during cardiogenesis and mediates postnatal cell cycle withdrawal. *Nature communications* 13: 7444

Corzett M, Mazrimas J, Balhorn R (2002) Protamine 1: protamine 2 stoichiometry in the sperm of eutherian mammals. *Molecular reproduction and development* 61: 519-527

de Mateo S, Gazquez C, Guimera M, Balasch J, Meistrich ML, Ballesca JL, Oliva R (2009) Protamine 2 precursors (Pre-P2), protamine 1 to protamine 2 ratio (P1/P2), and assisted reproduction outcome. *Fertil Steril* 91: 715-722

Dottermusch-Heidel C, Klaus ES, Gonzalez NH, Bhushan S, Meinhardt A, Bergmann M, Renkawitz-Pohl R, Rathke C, Steger K (2014) H3K79 methylation directly precedes the histone-to-protamine transition in mammalian spermatids and is sensitive to bacterial infections. *Andrology* 2: 655-665

Fujihara Y, Murakami M, Inoue N, Satouh Y, Kaseda K, Ikawa M, Okabe M (2010) Sperm equatorial segment protein 1, SPESP1, is required for fully fertile sperm in mouse. *J Cell Sci* 123: 1531-1536

Gadadhar S, Alvarez Viar G, Hansen JN, Gong A, Kostarev A, Ialy-Radio C, Leboucher S, Whitfield M, Ziyyat A, Toure A *et al* (2021) Tubulin glycylation controls axonemal dynein activity, flagellar beat, and male fertility. *Science* 371

Grant PA, Eberharter A, John S, Cook RG, Turner BM, Workman JL (1999) Expanded lysine acetylation specificity of Gcn5 in native complexes. *The Journal of biological chemistry* 274: 5895-5900

Green CD, Ma Q, Manske GL, Shami AN, Zheng X, Marini S, Moritz L, Sultan C, Gurczynski SJ, Moore BB *et al* (2018) A Comprehensive Roadmap of Murine Spermatogenesis Defined by Single-Cell RNA-Seq. *Developmental cell* 46: 651-667 e610

Ho CH, Takizawa Y, Kobayashi W, Arimura Y, Kimura H, Kurumizaka H (2021) Structural basis of nucleosomal histone H4 lysine 20 methylation by SET8 methyltransferase. *Life Sci Alliance* 4

Jones B, Su H, Bhat A, Lei H, Bajko J, Hevi S, Baltus GA, Kadam S, Zhai H, Valdez R *et al* (2008) The histone H3K79 methyltransferase Dot1L is essential for mammalian development and heterochromatin structure. *PLoS Genet* 4: e1000190

Kawano N, Kang W, Yamashita M, Koga Y, Yamazaki T, Hata T, Miyado K, Baba T (2010) Mice lacking two sperm serine proteases, ACR and PRSS21, are subfertile, but the mutant sperm are infertile in vitro. *Biology of reproduction* 83: 359-369

Kuo MH, Brownell JE, Sobel RE, Ranalli TA, Cook RG, Edmondson DG, Roth SY, Allis CD (1996) Transcription-linked acetylation by Gcn5p of histones H3 and H4 at specific lysines. *Nature* 383: 269-272

Lin H, Cheng K, Kubota H, Lan Y, Riedel SS, Kakiuchi K, Sasaki K, Bernt KM, Bartolomei MS, Luo M *et al* (2022) Histone methyltransferase DOT1L is essential for self-renewal of germline stem cells. *Genes & development* 36: 752-763

Luense LJ, Donahue G, Lin-Shiao E, Rangel R, Weller AH, Bartolomei MS, Berger SL (2019) Gcn5-Mediated Histone Acetylation Governs Nucleosome Dynamics in Spermiogenesis. *Developmental cell*

Oliva R (2006) Protamines and male infertility. *Human reproduction update* 12: 417-435

Rezaei-Gazik M, Vargas A, Amiri-Yekta A, Vitte AL, Akbari A, Barral S, Esmaeili V, Chuffart F, Sadighi-Gilani MA, Coute Y *et al* (2022) Direct visualization of pre-protamine 2

detects protamine assembly failures and predicts ICSI success. *Molecular human reproduction* 28
Shiota H, Barral S, Buchou T, Tan M, Coute Y, Charbonnier G, Reynoird N, Boussouar F, Gerard M, Zhu M *et al* (2018) Nut Directs p300-Dependent, Genome-Wide H4 Hyperacetylation in Male Germ Cells. *Cell reports* 24: 3477-3487 e3476
Soler-Ventura A, Gay M, Jodar M, Vilanova M, Castillo J, Arauz-Garofalo G, Villarreal L, Ballesca JL, Vilaseca M, Oliva R (2020) Characterization of Human Sperm Protamine Proteoforms through a Combination of Top-Down and Bottom-Up Mass Spectrometry Approaches. *J Proteome Res* 19: 221-237
Torregrosa N, Dominguez-Fandos D, Camejo MI, Shirley CR, Meistrich ML, Ballesca JL, Oliva R (2006) Protamine 2 precursors, protamine 1/protamine 2 ratio, DNA integrity and other sperm parameters in infertile patients. *Human reproduction (Oxford, England)* 21: 2084-2089
Yamauchi Y, Riel JM, Stoytcheva Z, Burgoyne PS, Ward MA (2010) Deficiency in mouse Y chromosome long arm gene complement is associated with sperm DNA damage. *Genome biology* 11: R66
Yu YE, Zhang Y, Unni E, Shirley CR, Deng JM, Russell LD, Weil MM, Behringer RR, Meistrich ML (2000) Abnormal spermatogenesis and reduced fertility in transition nuclear protein 1-deficient mice. *Proceedings of the National Academy of Sciences of the United States of America* 97: 4683-4688
Zatecka E, Castillo J, Elzeinova F, Kubatova A, Ded L, Peknicova J, Oliva R (2014) The effect of tetrabromobisphenol A on protamine content and DNA integrity in mouse spermatozoa. *Andrology* 2: 910-917

1st Revision - Editorial Decision **24th Mar 2023**

Dear Dr. Cocquet,

Thank you for the submission of your revised manuscript to our editorial offices. I have now received the reports from the three referees that were asked to re-evaluate your study, you will find below. As you will see, all three referees now fully support the publication of your study in EMBO reports. Referee #2 has some remaining questions and suggestions to improve the study, I ask you to address in a final revised manuscript. Please also provide a final p-b-p-response to these points.

Moreover, I have these further editorial requests I ask you to address:

- Please reduce the number of keywords to 5.

- Please finalize the data availability section, provide all direct links and accession codes, and remove referee tokens. Please make sure all datasets are public lates upon online publication of the study.

- Regarding data quantification and statistics, please make sure that the number "n" for how many independent experiments were performed, their nature (biological versus technical replicates), the bars and error bars (e.g. SEM, SD) and the test used to calculate p-values is indicated in the respective figure legends (main, EV and Appendix figures). Please also check that all the p-values are explained in the legend, and that these fit to those shown in the figure. Please provide statistical testing where applicable. Please avoid the phrase 'independent experiment', but clearly state if these were biological or technical replicates. Please also indicate (e.g. with n.s.) if testing was performed, but the differences are not significant.

In case n=2, please show the data as separate datapoints without error bars and statistics.

See also:
http://www.embopress.org/page/journal/14693178/authorguide#statisticalanalysis

If n<5, please show single datapoints for diagrams. Could statistical testing also be provided for Fig. 2E (right panel) and Appendix Fig. S2F?

- The requested source data for Fig. 3E (blot) seems missing. Please check and add this upon re-submission.

- The resolution for the Appendix Figures is too low. Please re-supply the appendix document with figures provided at a higher resolution as per our guidelines. https://www.embopress.org/pb-assets/embo-site/EMBOPress_Figure_Guidelines_061115-1561436025777.pdf

- In the Appendix, please name the figures Appendix Figure Sx (the S is missing). The panel labelling should just be 'A', 'B, 'C' ... etc., not "S1A", "S1B", "S1C". Please fix this. Moreover, please move the legends below the respective figures. This is more comprehensible for the readers.

- Finally, please find attached a word file of the manuscript text (provided by our publisher) with changes we ask you to include in your final manuscript text and comments. Please use the attached file as basis for further revisions and provide your final manuscript file with track changes, in order that we can see any modifications done.

In addition, I would need from you:
- a short, two-sentence summary of the manuscript (not more than 35 words).
- two to four short bullet points highlighting the key findings of your study (two lines each).
- a schematic summary figure that provides a sketch of the major findings (not a data image) in jpeg or tiff format (with the exact width of 550 pixels and a height of not more than 400 pixels) that can be used as a visual synopsis on our website.

I look forward to seeing the final revised version of your manuscript when it is ready. Please let me know if you have questions regarding the revision.

Please use this link to submit your revision: https://embor.msubmit.net/cgi-bin/main.plex

Best,

Achim Breiling
Senior Editor
EMBO Reports

Referee #1:

The authors have addressed previous concerns.

Referee #2:

The authors have incorporated several new data sets and edited the manuscript in a way such that it is (almost) suitable for publication.
Two suggestions for further improvements:
1. FigS3B: following the original comments, it is still not clear to this reviewer how the quantification of the WB shown on the right can result in the data presented in the barplot on the left. Please clarify.

2. Thank you for incorporating H3K79me2 ChIP-data into the manuscript (Fig. 5C, 5D). To enhance the understanding of the possible relevance of DOT1L and H3K79me2 in transcriptional regulation, it is strongly advised to relate H3K79me2 occupancy levels along genes in relation to their transcriptional status. Are highly expressed genes marked while non-expressed genes are not? Is there any difference in occupancy along the length of the genes, possibly pending on their expression levels?
Once these data have been presented, the data in the current Figure 5C will be better understandable.
Moreover, is there any logic from the H3K79me2 perspective (occupancy levels, distribution along gene bodies) underlying why some genes get mis-expressed in the Dot1l mutant cells, and others not?

3. The overall resolution of the panels in the supplementary figures is too low.

Referee #3:

My initial review was positive and I have identified three minor points that required improvement. The authors have fully addressed all the points and I would recommend the manuscript for publication.

Point by Point response to Referee's comments.

Thank you for your positive feedback. Our responses are inserted in blue below.

Referee #1:

The authors have addressed previous concerns.

Referee #2:

The authors have incorporated several new data sets and edited the manuscript in a way such that it is (almost) suitable for publication.
Two suggestions for further improvements:
1. FigS3B: following the original comments, it is still not clear to this reviewer how the quantification of the WB shown on the right can result in the data presented in the barplot on the left. Please clarify.

The western blot image shown in FigS3B corresponds to E2 experiment. Quantification was performed on a higher exposure image, which we now show in the revised version of FigS3B. The legend was modified accordingly.

2. Thank you for incorporating H3K79me2 ChIP-data into the manuscript (Fig. 5C, 5D). To enhance the understanding of the possible relevance of DOT1L and H3K79me2 in transcriptional regulation, it is strongly advised to relate H3K79me2 occupancy levels along genes in relation to their transcriptional status. Are highly expressed genes marked while non-expressed genes are not? Is there any difference in occupancy along the length of the genes, possibly pending on their expression levels? Once these data have been presented, the data in the current Figure 5C will be better understandable. Moreover, is there any logic from the H3K79me2 perspective (occupancy levels, distribution along gene bodies) underlying why some genes get mis-expressed in the Dot1l mutant cells, and others not?

In our manuscript, we correlated H3K79me2 mark with the gene deregulation observed in *Dot1l*-KO male germ cells (Fig 5C and 5D). We agree with the referee that the link between H3K79me2 gene occupancy and transcriptional status (level of expression, expressed vs. non-expressed genes) deserves further interest but we think it is beyond the scope of the present study. We hope to address this question in the context of gene expression dynamic throughout the various stages of spermatogenesis, in another study.

3. The overall resolution of the panels in the supplementary figures is too low.
Thank you for this comment, we will upload supplementary figures with a better resolution.

Referee #3:

My initial review was positive and I have identified three minor points that required improvement. The authors have fully addressed all the points and I would recommend the manuscript for publication.

2nd Revision - Editorial Decision 13th Apr 2023

Dr. Julie COCQUET
Institut Cochin - Inserm - CNRS - Université Paris Cité
24 rue du Faubourg St Jacques
PARIS 75014
France

Dear Dr. COCQUET,

I am very pleased to accept your manuscript for publication in the next available issue of EMBO reports. Thank you for your contribution to our journal.

At the end of this email I include important information about how to proceed. Please ensure that you take the time to read the information and complete and return the necessary forms to allow us to publish your manuscript as quickly as possible.

As part of the EMBO publication's Transparent Editorial Process, EMBO reports publishes online a Review Process File to accompany accepted manuscripts. As you are aware, this File will be published in conjunction with your paper and will include the referee reports, your point-by-point response and all pertinent correspondence relating to the manuscript.

If you do NOT want this File to be published, please inform the editorial office within 2 days, if you have not done so already, otherwise the File will be published by default [contact: emboreports@embo.org]. If you do opt out, the Review Process File link will point to the following statement: "No Review Process File is available with this article, as the authors have chosen not to make the review process public in this case."

Thank you again for your contribution to EMBO reports and congratulations on a successful publication. Please consider us again in the future for your most exciting work.

Yours sincerely,

Achim Breiling
Senior Editor
EMBO Reports

****

THINGS TO DO NOW:

Please note that you will be contacted by Wiley Author Services to complete licensing and payment information. The required 'Page Charges Authorization Form' is available here: https://www.embopress.org/pb-assets/embo-site/er_apc.pdf - please download and complete the form and return to embopressproduction@wiley.com

EMBO Press participates in many Publish and Read agreements that allow authors to publish Open Access with reduced/no publication charges. Check your eligibility: https://authorservices.wiley.com/author-resources/Journal-Authors/open-access/affiliation-policies-payments/index.html

You will receive proofs by e-mail approximately 2-3 weeks after all relevant files have been sent to our Production Office; you should return your corrections within 2 days of receiving the proofs.

Please inform us if there is likely to be any difficulty in reaching you at the above address at that time. Failure to meet our deadlines may result in a delay of publication, or publication without your corrections.

All further communications concerning your paper should quote reference number EMBOR-2022-56316V3 and be addressed to emboreports@wiley.com.

Should you be planning a Press Release on your article, please get in contact with emboreports@wiley.com as early as possible, in order to coordinate publication and release dates.

EMBO Press Author Checklist

Corresponding Author Name: Julie COCQUET	
Journal Submitted to: EMBO REPORTS	
Manuscript Number: EMBOR-2022-56316V2	

USEFUL LINKS FOR COMPLETING THIS FORM
[The EMBO Journal - Author Guidelines]
[EMBO Reports - Author Guidelines]
[Molecular Systems Biology - Author Guidelines]
[EMBO Molecular Medicine - Author Guidelines]

Reporting Checklist for Life Science Articles (updated January 2022)
This checklist is adapted from Materials Design Analysis Reporting (MDAR) Checklist for Authors. MDAR establishes a minimum set of requirements in transparent reporting in the life sciences (see Statement of Task: 10.31222/osf.io/9sm4x). Please follow the journal's guidelines in preparing your manuscript.
Please note that a copy of this checklist will be published alongside your article.

Abridged guidelines for figures
1. Data
The data shown in figures should satisfy the following conditions:
- the data were obtained and processed according to the field's best practice and are presented to reflect the results of the experiments in an accurate and unbiased manner.
- ideally, figure panels should include only measurements that are directly comparable to each other and obtained with the same assay.
- plots include clearly labeled error bars for independent experiments and sample sizes. Unless justified, error bars should not be shown for technical replicates.
- if n<5, the individual data points from each experiment should be plotted. Any statistical test employed should be justified.
- Source Data should be included to report the data underlying figures according to the guidelines set out in the authorship guidelines on Data Presentation.

2. Captions
Each figure caption should contain the following information, for each panel where they are relevant:
- a specification of the experimental system investigated (eg cell line, species name).
- the assay(s) and method(s) used to carry out the reported observations and measurements.
- an explicit mention of the biological and chemical entity(ies) that are being measured.
- an explicit mention of the biological and chemical entity(ies) that are altered/varied/perturbed in a controlled manner.
- the exact sample size (n) for each experimental group/condition, given as a number, not a range;
- a description of the sample collection allowing the reader to understand whether the samples represent technical or biological replicates (including how many animals, litters, cultures, etc.).
- a statement of how many times the experiment shown was independently replicated in the laboratory.
- definitions of statistical methods and measures:
  - common tests, such as t-test (please specify whether paired vs. unpaired), simple $\chi 2$ tests, Wilcoxon and Mann-Whitney tests, can be unambiguously identified by name only, but more complex techniques should be described in the methods section;
  - are tests one-sided or two-sided?
  - are there adjustments for multiple comparisons?
  - exact statistical test results, e.g., P values = x but not P values < x;
  - definition of 'center values' as median or average;
  - definition of error bars as s.d. or s.e.m.

Please complete ALL of the questions below.
Select "Not Applicable" only when the requested information is not relevant for your study.

Materials

Newly Created Materials	Information included in the manuscript?	In which section is the information available? (Reagents and Tools Table, Materials and Methods, Figures, Data Availability Section)
New materials and reagents need to be available; do any restrictions apply?	Not Applicable	

Antibodies	Information included in the manuscript?	In which section is the information available? (Reagents and Tools Table, Materials and Methods, Figures, Data Availability Section)
For **antibodies** provide the following information:<br>- Commercial antibodies: RRID (if possible) or supplier name, catalogue number and or/clone number<br>- Non-commercial: RRID or citation	Yes	Appendix Table S2

DNA and RNA sequences	Information included in the manuscript?	In which section is the information available? (Reagents and Tools Table, Materials and Methods, Figures, Data Availability Section)
**Short novel DNA or RNA including primers, probes**: provide the sequences.	Yes	Appendix Table S1

Cell materials	Information included in the manuscript?	In which section is the information available? (Reagents and Tools Table, Materials and Methods, Figures, Data Availability Section)
**Cell lines:** Provide species information, strain. Provide accession number in repository **OR** supplier name, catalog number, clone number, and/**OR** RRID.	Not Applicable	
**Primary cultures:** Provide species, strain, sex of origin, genetic modification status.	Not Applicable	
Report if the cell lines were recently **authenticated** (e.g., by STR profiling) and tested for mycoplasma contamination.	Not Applicable	

Experimental animals	Information included in the manuscript?	In which section is the information available? (Reagents and Tools Table, Materials and Methods, Figures, Data Availability Section)
**Laboratory animals or Model organisms:** Provide species, strain, sex, age, genetic modification status. Provide accession number in repository **OR** supplier name, catalog number, clone number, **OR** RRID.	Yes	Materials and Methods
**Animal observed in or captured from the field:** Provide species, sex, and age where possible.	Not Applicable	Materials and Methods
Please detail **housing and husbandry conditions**.	Yes	

Plants and microbes	Information included in the manuscript?	In which section is the information available? (Reagents and Tools Table, Materials and Methods, Figures, Data Availability Section)
**Plants:** provide species and strain, ecotype and cultivar where relevant, unique accession number if available, and source (including location for collected wild specimens).	Not Applicable	
**Microbes:** provide species and strain, unique accession number if available, and source.	Not Applicable	

Human research participants	Information included in the manuscript?	In which section is the information available? (Reagents and Tools Table, Materials and Methods, Figures, Data Availability Section)
If collected and within the bounds of privacy constraints report on age, sex and gender or ethnicity for all study participants.	Not Applicable	

Core facilities	Information included in the manuscript?	In which section is the information available? (Reagents and Tools Table, Materials and Methods, Figures, Data Availability Section)
If your work benefited from core facilities, was their service mentioned in the acknowledgments section?	Yes	Acknowledgments

Design

Study protocol	Information included in the manuscript?	In which section is the information available? (Reagents and Tools Table, Materials and Methods, Figures, Data Availability Section)
If study protocol has been **pre-registered, provide DOI in the manuscript**. For clinical trials, provide the trial registration number **OR** cite DOI.	Not Applicable	
Report the **clinical trial registration number** (at ClinicalTrials.gov or equivalent), where applicable.	Not Applicable	

Laboratory protocol	Information included in the manuscript?	In which section is the information available? (Reagents and Tools Table, Materials and Methods, Figures, Data Availability Section)
Provide DOI OR other citation details if **external detailed step-by-step protocols** are available.	Not Applicable	

Experimental study design and statistics	Information included in the manuscript?	In which section is the information available? (Reagents and Tools Table, Materials and Methods, Figures, Data Availability Section)
Include a statement about **sample size** estimate even if no statistical methods were used.	Yes	Materials and Methods, Figures
Were any steps taken to minimize the effects of subjective bias when allocating animals/samples to treatment (e.g. **randomization procedure**)? If yes, have they been described?	Not Applicable	
Include a statement about **blinding** even if no blinding was done.	Not Applicable	
Describe **inclusion/exclusion criteria** if samples or animals were excluded from the analysis. Were the criteria pre-established?<br><br>If sample or data points were omitted from analysis, report if this was due to attrition or intentional exclusion and provide justification.	Not Applicable	
For every figure, are **statistical tests** justified as appropriate? Do the data meet the assumptions of the tests (e.g., normal distribution)? Describe any methods used to assess it. Is there an estimate of variation within each group of data? Is the variance similar between the groups that are being statistically compared?	Yes	Materials and Methods, Figures

Sample definition and in-laboratory replication	Information included in the manuscript?	In which section is the information available? (Reagents and Tools Table, Materials and Methods, Figures, Data Availability Section)
In the figure legends: state number of times the experiment was **replicated** in laboratory.	Yes	Figures
In the figure legends: define whether data describe **technical or biological replicates**.	Yes	Figures

Ethics

Ethics	Information included in the manuscript?	In which section is the information available? (Reagents and Tools Table, Materials and Methods, Figures, Data Availability Section)
Studies involving **human participants**: State details of **authority granting ethics approval** (IRB or equivalent committee(s), provide reference number for approval.	Not Applicable	
Studies involving **human participants**: Include a statement confirming that **informed consent** was obtained from all subjects and that the experiments conformed to the principles set out in the WMA Declaration of Helsinki and the Department of Health and Human Services Belmont Report.	Not Applicable	
Studies involving **human participants:** For publication of **patient photos**, include a statement confirming that consent to publish was obtained.	Not Applicable	
Studies involving experimental **animals**: State details of **authority granting ethics approval** (IRB or equivalent committee(s), provide reference number for approval. Include a statement of compliance with ethical regulations.	Yes	Materials and Methods
Studies involving **specimen and field samples:** State if relevant **permits** obtained, provide details of authority approving study; if none were required, explain why.	Not Applicable	

Dual Use Research of Concern (DURC)	Information included in the manuscript?	In which section is the information available? (Reagents and Tools Table, Materials and Methods, Figures, Data Availability Section)
Could your study fall under dual use research restrictions? Please check biosecurity documents and list of **select agents and toxins** (CDC): https://www.selectagents.gov/sat/list.htm	Not Applicable	
If you used a select agent, is the security level of the lab appropriate and reported in the manuscript?	Not Applicable	
If a study is subject to dual use research of concern regulations, is the name of the **authority granting approval and reference number** for the regulatory approval provided in the manuscript?	Not Applicable	

Reporting

The MDAR framework recommends adoption of discipline-specific guidelines, established and endorsed through community initiatives. Journals have their own policy about requiring specific guidelines and recommendations to complement MDAR.

Adherence to community standards	Information included in the manuscript?	In which section is the information available? (Reagents and Tools Table, Materials and Methods, Figures, Data Availability Section)
State if relevant guidelines or checklists (e.g., **ICMJE, MIBBI, ARRIVE, PRISMA**) have been followed or provided.	Not Applicable	
For **tumor marker prognostic studies,** we recommend that you follow the **REMARK** reporting guidelines (see link list at top right). See author guidelines, under 'Reporting Guidelines'. Please confirm you have followed these guidelines.	Not Applicable	
For **phase II and III randomized controlled trials**, please refer to the **CONSORT** flow diagram (see link list at top right) and submit the CONSORT checklist (see link list at top right) with your submission. See author guidelines, under 'Reporting Guidelines'. Please confirm you have submitted this list.	Not Applicable	

Data Availability

Data availability	Information included in the manuscript?	In which section is the information available? (Reagents and Tools Table, Materials and Methods, Figures, Data Availability Section)
Have **primary datasets** been deposited according to the journal's guidelines (see 'Data Deposition' section) and the respective accession numbers provided in the Data Availability Section?	Yes	Data availability
Were **human clinical and genomic datasets** deposited in a public access-controlled repository in accordance to ethical obligations to the patients and to the applicable consent agreement?	Not Applicable	
Are **computational models** that are central and integral to a study available without restrictions in a machine-readable form? Were the relevant accession numbers or links provided?	Yes	Materials and Methods (Code availability)
If publicly available data were reused, provide the respective **data citations in the reference list**.	Yes	References
